# Supplementary material for: Estimating the impact of school closures on the COVID-19 dynamics in 74 countries: A modelling analysis
Source: PLoS Med. 2025 Jan 21;22(1):e1004512. doi: 10.1371/journal.pmed.1004512 (PMC11793732; doi:10.1371/journal.pmed.1004512)
Supplement: S1 Appendix — (PDF) [file pmed.1004512.s001.pdf]

Estimating the impact of school closures on the COVID-19 epidemics in 74 countries
Supplementary Appendix

Ragonnet R, Hughes AE, Shipman DS, Meehan MT, Henderson AS, Briffoteaux G, Melab N, Tuyttens D, McBryde ES, Trauer JM

Contents

A Methododological details **2**
A.1 Model description . . . 2
A.1.1 General approach . . . 2
A.1.2 Transmission model . . . 3
A.1.3 Estimation of COVID-19-related hospital pressure and deaths . . . 8
A.1.4 Model parameters . . . 9
A.2 Software and code used to conduct the analyses . . . 15
A.2.1 Code . . . 15
A.2.2 Software implementation . . . 15
A.3 Model calibration and uncertainty propagation . . . 15
A.3.1 Parameters varied during calibration . . . 16
A.3.2 Calibration targets . . . 16
A.3.3 Likelihood definition . . . 17

B Additional results **26**
B.1 Exploring the findings heterogeneity . . . 26
B.1.1 Proportion of individuals aged under 15 years old . . . 27
B.1.2 Proportion of individuals aged 70 years old and above . . . 27
B.1.3 Proportion of enrolled students . . . 27
B.1.4 Total duration of school closures . . . 28
B.1.5 Average PHSM stringency index . . . 28
B.1.6 Multivariate analysis . . . 29
B.2 Sensitivity analyses . . . 30
B.2.1 SA1: Increased household transmission during school closures . . . 30
B.2.2 SA2: Google Mobility data not included . . . 30
B.2.3 SA3: Using alternative mixing matrices . . . 30
B.2.4 Output comparisons between analyses . . . 31
B.2.5 Likelihood comparisons between analyses . . . 32
B.2.6 Testing alternative model structures to capture incubation and active disease periods 32
B.2.7 Exploring more stringent school closures during the Delta wave in Indonesia . . . 32
B.3 Posterior samples exploration . . . 33
B.4 Detailed country-specific results . . . 49

Part A

Methododological details

A.1 Model description

A.1.1 General approach

We use a semi-mechanistic compartmental model of COVID-19 transmission governed by ordinary differential equations (ODEs) to simulate country-specific COVID-19 epidemics during the first three years of the pandemic (2020-2022). Our model captures important factors relevant to COVID-19 dynamics such as age-specific characteristics, heterogeneous mixing, vaccination and the emergence of different variants of concern. The ODE-based model is used to capture only states relevant to transmission, whereas hospitalisations and deaths are estimated through a convolution process applied to the ODE-based model's outputs. This process combines the model-estimated disease incidence with statistical distributions modelling the time to hospitalisation, the hospital stay duration and the time to death. This approach presents two main advantages. First it reduces the complexity of the dynamic system relying on numerical solving of ODEs, which is computationally expensive. Second, the convolution approach allows for more flexibility and produces more realistic assumptions regarding the timing of hospitalisation and death, compared to what could be achieved with a simple compartmental approach. The following sections describe the model in details and Figure A summarises the overall approach used in our analysis.

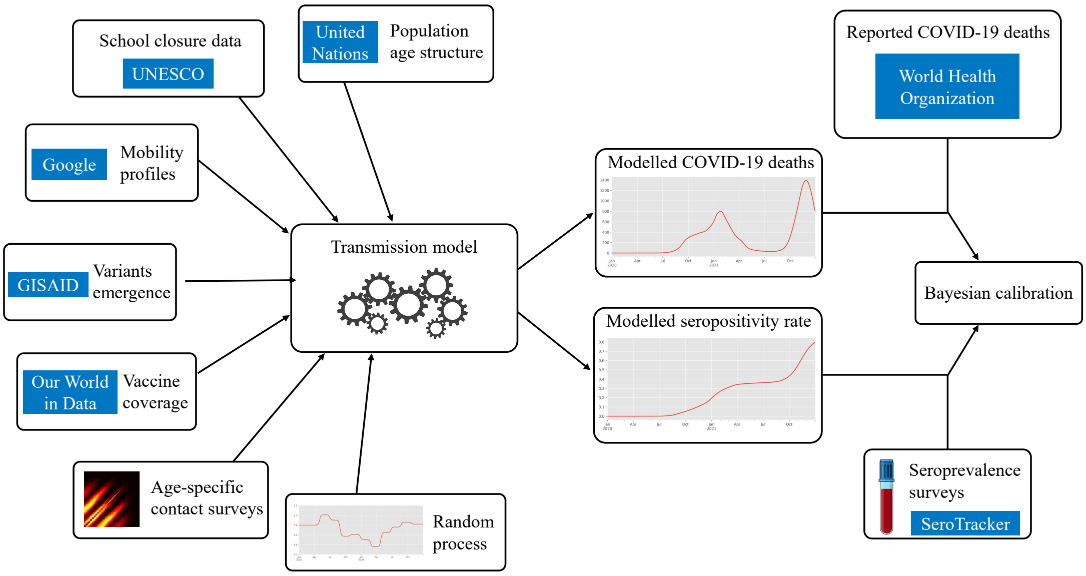

Fig A: Conceptual approach used to build and calibrate the models

A.1.2 Transmission model

A.1.2.1 Compartment types and sequence

Model compartments represent sequential progressions through the processes of infection with, progression through, and recovery from the phases of SARS-CoV-2 infection and COVID-19 disease. The following types of compartments are implemented:

- Susceptible
  - Persons never previously infected with SARS-CoV-2.
- Latent
  - Persons recently infected with SARS-CoV-2, but not yet in the active phase of the disease.
  - These individuals may still be infectious (see details in the next paragraph).
- Active
  - Persons with active COVID-19 who are currently infectious.
- Recovered
  - Persons recovered from COVID-19 during the model simulation period
  - Reinfection from these compartments is permitted through exposure to a different strain than the one that most recently infected the individual (see strain stratification section for details).

The base model structure consists of a sequence of one susceptible compartment (S), four exposed compartments (E_1, ..., E_4), four active disease compartments (I^1, ..., I^4) and one recovered compartment (R) (Figure B).

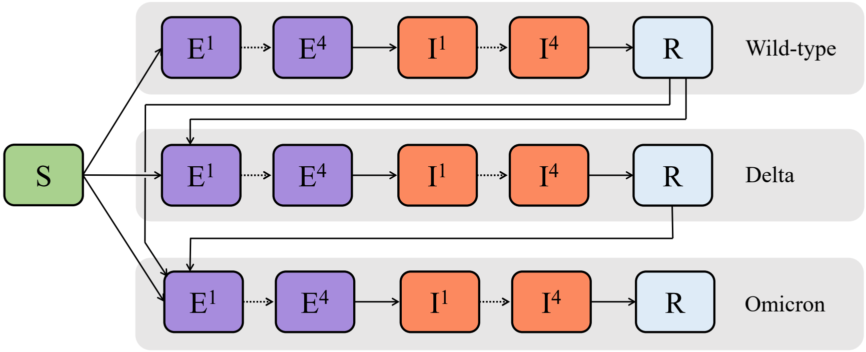

Fig B: Compartmental model structure. S = Susceptible, E = Exposed, I = Active disease, R = Recovered. Stratification by age and vaccination status not shown.

The main rationale for using multiple serial compartments for both the exposed and active states is to achieve an Erlang distribution for the time spent in each of these states. This distribution is more realistic than the exponential distribution which is the consequence of a single compartment assumption, because the Erlang distribution does not have a large density mass around 0 and is not heavy-tailed. Figure C illustrates the modelled distributions of the incubation active disease periods.

The four active disease compartments all have identical characteristics. However, the last two exposed compartments (E^3 and E^4) are infectious whereas the first two (E^1 and E^2) are not. In other words, compartments E^1 and E^2 represent the latent period (i.e. before infectiousness), while the full incubation period is modelled with the four compartments E^1-E^4. We further assume that the infectious exposed compartments (E^3 and E^4) are half as infectious as the active disease compartments.

In a Sensitivity Analysis, we explored the impact of using alternative model structures on our main findings. Namely, we repeated the analyses for the three countries highlighted in the main text (Morocco, the UK and Indonesia), this time considering different numbers of serial compartments to capture both incubation and active disease periods. See results in Section B.2.6.

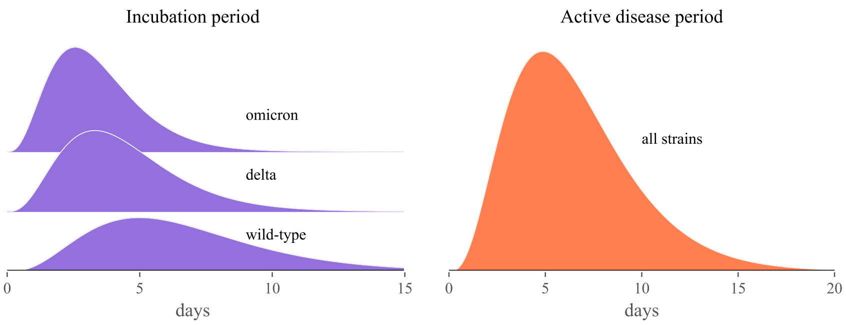

Fig C: Modelled distributions of the incubation and active disease periods. Active disease period shown for an average duration of 8 days, but this parameter is varied during calibration.

A.1.2.2 Model stratification by age

All compartments of the base compartmental structure were stratified by age into the following bands: zero to 14 years / 15 to 24 years / 25 to 49 years / 50 to 69 years / 70 years and above.

The initial population was distributed between the different age bands to reflect the age distributions reported by the United Nations Population Division. Demographic processes, including births, ageing and non-infection-related deaths are not simulated, given the timeframes considered in this simulation.

We assumed heterogeneous mixing between the different age groups to account for the assortative nature of social interactions by age (see Section A.1.2.5). Age was assumed to affect:

- susceptibility to infection
- risk of COVID-19 hospitalisation
- risk of COVID-19 death
- vaccination rate (see Section A.1.2.3)

A.1.2.3 Capturing the effects of vaccination

History of vaccination was captured by stratifying all model compartments by vaccination status. Two vaccination strata were included to represent those who have received at least two doses of a COVID-19 vaccine, and those who have not.

We used data from *Our World in Data* to inform the modelled dynamic vaccination coverage [28]. In particular, we specified the time-variant proportion of vaccinated people in our model using the reported proportion of people "fully vaccinated", defined as individuals who have received all doses for a prescribed vaccination protocol for a general population. We assumed that older individuals are vaccinated first by prioritising the modelled age groups in descending order, to align with WHO policy to prioritise the vaccination of older adults within the general population [46]. That is, the oldest age group receives all available vaccines until a saturation coverage of 80% is reached for this group. Then the next oldest category starts receiving vaccines and we repeat this process until all available vaccines are allocated. Note that in the event that the population-level vaccine coverage exceeds 80%, the saturation coverage was set equal to the population-level coverage.

Let us consider two successive time points t_i and t_{i+1} for which vaccination data are available. Let us denote $r_{a,i}$ and $r_{a,i+1}$ the associated vaccine coverage for age group a. The time-variant and age-specific vaccination rate per capita $\omega_a(t)$ verifies:

$$1 - r_{a,i+1} = (1 - r_{a,i})e^{-w_a(t)(t_{i+1}-t_i)} \quad ,\forall t \in [t_i, t_{i+1}). \tag{A.1}$$

Then,

$$\omega_a(t) = \frac{\ln(1 - r_{a,i}) - \ln(1 - r_{a,i+1})}{t_{i+1} - t_i} \quad ,\forall t \in [t_i, t_{i+1}), \tag{A.2}$$

where $\ln(x)$ represents the natural logarithm of x.

Figure D shows the modelled vaccination coverage over time against the reported data for the analysed countries.

The effect of vaccination on transmission is to reduce the rate of infection partially for all persons at risk of infection in the vaccinated stratum. This includes both fully susceptible (never previously infected) persons, as well as recovered persons who are at risk of reinfection. The model allows for hybrid immunity in the sense that the vaccination-induced relative reduction of transmission risk is multiplied with that induced by previous infection. Vaccination is also assumed to reduce the risk of hospitalisation and death.

Emerging variants of concern (VoCs) may partially escape vaccine-induced (as well as infection-induced) immunity, as described further below (Table B and Section A.1.2.4).

A.1.2.4 Modelling multiple viral strains

The model was stratified by "strain" to simulate the emergence of multiple variants of concern (VoC). This approach explicitly represents multiple competing strains, each with an independent force of infection calculation. We assumed that VoCs can have different levels of transmissibility, incubation period and disease severity (hospitalisation and death risks) compared to the ancestral COVID-19 strain. In addition, VoCs were assumed to escape immunity partially for both vaccination- and infection-related immunity.

We assumed that individuals previously infected with the wild-type strain could only be reinfected with the delta or omicron strains. However, such individuals have a reduced risk of infection with these variants compared to infection-naive individuals (82% and 45% reduction for delta and omicron, respectively) [41]. We assumed that individuals previously infected with the delta variant could only be reinfected with the omicron variant, with an infection risk reduced by 45% compared to infection-naive individuals [41]. The other parameters used to represent strain-specific characteristics are presented in Table B.

Seeding of each new strain into the model was achieved through the importation of a small number (10 per million population) of new infectious persons with the relevant strain into the model. The seeding process was implemented over a ten-day period, with the start of this period extracted from the GISAID database for each country [11]. We considered a strain (either Delta or Omicron) to have emerged when, during a single week in GISAID, at least two cases of that strain were reported, and these cases accounted for at least 1% of all reported cases during that week.

A.1.2.5 Dynamic social mixing

The model captures changes in social interactions over time through a dynamic age-specific mixing matrix. The following sections describe how this matrix was defined and how it captures the different non-pharmaceutical interventions implemented in the analysed countries, including school closures. The overall approach is also illustrated by Figure F.

A.1.2.5.1 Reference mixing matrices

We extracted country-specific contact matrices using the *conmat* R package which derives social mixing matrices from contact survey data [12, 31]. These matrices provide the average numbers of contacts per day between different age groups, disaggregated by the following locations: home, school, work, other locations. In a Sensitivity Analysis, we repeated our estimations using alternative contact matrices provided by Mistry et al. for the 25 countries in our study where these matrices were available [30]. The results associated with these additional analyses are presented in Section B.2.3.

The pre-COVID-19 contact matrix (before adjustments for mobility changes) is calculated as the summation of the four location-specific contact matrices: $C_0 = C_H + m_S C_S + C_W + C_L$, where C_H, C_S, C_W and C_L are the age-specific contact matrices associated with households, schools, workplaces and other locations, respectively. Note that the school contribution C_S is multiplied by the factor m_S that was varied during model calibration (see Section A.3), in order to account for uncertainty around the relative contribution of school contacts to COVID-19 transmission.

A.1.2.5.2 Modifications of contact rates over time

To capture mobility changes over time, the contributions of the matrices C_S, C_W and C_L vary with time, such that the input contact matrix can be written as:

$$C(t) = h(t)^2 C_H + s(t)^2 m_S C_S + w(t)^2 C_W + l(t)^2 C_L \tag{A.3}$$

The modifying functions h (for households), s (for schools), w (for work) and l (for other-locations) were each squared to capture the effect of the mobility changes on both the infector and the infectee in any given interaction that could potentially result in transmission.

School closure/re-opening

Reduced attendance at schools was represented through the function s, which represents the proportion of all school students currently attending on-site teaching. If schools are fully closed at time t, $s(t) = 0$ and C_S does not contribute to the overall mixing matrix $C(t)$. The function s was derived from the UNESCO database on school closures from the start of the COVID-19 pandemic [44]. This database provides school opening status over time as a categorical variable taking the following values: "Fully open", "Partially open", "Academic break", "Closed due to COVID-19". Table A indicates how the different categorical values were converted into the numerical function s.

Table A: Assumed percentage of students on-site for the different UNESCO school closure categories.

**UNESCO category**	**Assumed proportion of students on-site at national level ($s(t)$)**
Fully open	100%
Partially open	10-50%
Academic break	0%
Closed due to COVID-19	0%

We included uncertainty around the value associated with the partial closure category, as there were no quantitative data available to inform this parameter [44]. The partial closure periods are likely to be periods where only a small fraction of students such as children of "essential workers" were attending school. We assumed that between 10 and 50% of students attended on-site learning during these periods.

To model the counterfactual "no school closure" scenario, we assumed that the schools were "Fully open" during the periods reported as "Partially open" or "Closed due to COVID-19".

Figure E summarises UNESCO data on school closure for the analysed countries.

Dynamic mobility outside of schools and homes

Changes to people's mobility in places other than schools and homes were modelled using Google Mobility data [23], after applying a seven-day moving average smoothing. We used the "Workplace" category of the Google data to scale the work-related matrix contribution C_W to overall mixing over time, using the adjusting function w. The "other locations" matrix C_L was scaled through the adjusting function l which was defined as the average of the Google mobility indicators across the following Google categories: "Retail and recreation", "Grocery and pharmacy" and "Transit stations".

Household contacts

In the base case analysis, the contribution of household contacts to the overall mixing matrix was fixed over time (i.e. $h(t) = 1$ in Equation A.3). Although Google provides mobility estimates for residential contacts, the nature of these data is different from that of each of the other Google mobility types. They represent the time spent in that location, as opposed to other categories, which measure a change in total visits rather than duration. The daily frequency with which people attend their residence is likely to be close to one, and we considered that household members likely have a daily opportunity for infection with each other household member regardless of the background level of mobility. Therefore, we did not implement a function to scale the contribution of household contacts to the mixing matrix with time.

A.1.2.5.3 Sensitivity analyses around dynamic social mixing

In addition to the Base Case analysis described above, we performed two sensitivity analyses considering different assumptions for the modelled social mixing.

SA1: School closures increase household contact rates In a first sensitivity analysis (SA1), we considered an alternative assumption under which the effective contact rates within households were increased during periods of school closure. In that case, the household component of the mixing matrix is modified by the following function:

$$h(t) = 1 + 0.20(1 - s(t)) \quad ,$$

where s is the function modifying school contacts as introduced in Section A.1.2.5.2. This is equivalent to assuming that each individual has 20% more household effective contact potential when schools are fully closed.

SA2: No Google mobility data In another sensitivity analysis (SA2), we removed the contribution of the Google mobility data to the modelled social mixing (see Figure F). In this configuration, the calibrated random process ($W(t)$) is implicitly responsible for capturing mobility changes.

A.1.2.6 Random transmission adjustment

The risk of SARS-CoV-2 transmission per contact was adjusted by a time-variant random process, making the model semi-mechanistic. This random process reflects the fact that all the variations observed in the transmission risk in the real world cannot be explained solely by the factors that are explicitly captured through our model inputs (such as vaccination, dynamic mobility or new variants' emergence). We therefore allowed for random perturbations to the risk of transmission over time, although the random process was highly auto-correlated to avoid unrealistic changes over a short period of time.

We used a random walk with Gaussian update defined by:

$$\begin{aligned} W(0) &= 0 \\ W(t+1) &\sim \mathcal{N}(W(t), 0.5) \quad , \end{aligned} \tag{A.4}$$

where $\mathcal{N}$ denotes the normal distribution. The random process W was updated every two months and was transformed using the exponential function before being applied to the risk of transmission per contact (see Equation A.6). Finally, the contribution of the random process to the risk of transmission was squared in order to capture its effect on both the susceptible and the infectious individuals (see Equation A.6). The decision to update the random process every two months represents a compromise between achieving high resolution - benefiting from better model fits with more frequent updates - and keeping the number of calibrated parameters within a reasonable range. Notably, each update to the random process necessitates the calibration of an additional parameter using Bayesian inference.

A.1.2.7 Ordinary differential equations

We now introduce some new notation. Modelled age groups are indicated by the subscript a, and $\mathcal{A}$ represents the set of all modelled age groups (see Section A.1.2.2). Vaccination status is represented by the subscript v, and $\mathcal{V}$ is the set of vaccination statuses (i.e. $\mathcal{V} = \{$"0", "1"$\}$, where "0" represents unvaccinated people and "1" represents vaccinated people). The subscript s is used to represent the different viral strains, and $\mathcal{S}$ is the set of all strains (i.e. $\mathcal{S} = \{$"wild-type", "delta", "omicron"$\}$). The average incubation period duration associated with strain s is denoted q_s and the average duration of active disease is denoted w. The relative susceptibility to infection with strain s of individuals aged a with vaccination status v is denoted $\rho_{a,v,s}$. The term $b_{a,v,s}(t)$ designates the introduction of individuals of age a and with vaccination status v that are infected with strain s (infection seeding).

Vaccination is characterised by the age-specific and time-variant per-capita vaccination rate ω_a. For each type of compartment, the rate ω_a is multiplied by the size of the unvaccinated population associated with the relevant compartment to determine the number of individuals transitioning to the vaccinated strata at each time step. In the ordinary differential equations that follow, the terms describing the vaccination flows must be positive when pertaining to destination (vaccinated) strata, and negative when pertaining to source (unvaccinated) strata. To capture this, we use the binary operator Φ_v to switch between a positive and negative multiplier depending on vaccination status:

$$\Phi_v = \begin{cases} 1 & \text{if } v = 1 \\ -1 & \text{if } v = 0 \end{cases}$$

Finally, $\chi_{s,\sigma}$ represents the relative susceptibility to infection with strain σ for individuals whose most recent infection episode was with strain s. Using this new notation combined with those previously introduced, we can describe the transmission model with the following set of ordinary differential equations:

$$\begin{aligned}
\frac{dS_{a,v}}{dt} &= -\sum_{s\in\mathcal{S}} \lambda_{a,s}(t)\rho_{a,v,s}S_{a,v} + \Phi_v\omega_a(t)S_{a,v=0} \quad ,\\
\frac{dE^1_{a,v,s}}{dt} &= \lambda_{a,s}(t)\rho_{a,v,s}\Big(S_{a,v} + \sum_{\sigma\in\mathcal{S}} \chi_{\sigma,s}R_{a,v,\sigma}\Big) - \frac{4}{q_s}E^1_{a,v,s} + \Phi_v\omega_a(t)E^1_{a,v=0,s} \quad ,\\
\frac{dE^k_{a,v,s}}{dt} &= \frac{4}{q_s}E^{k-1}_{a,v,s} - \frac{4}{q_s}E^k_{a,v,s} + \Phi_v\omega_a(t)E^k_{a,v=0,s} \quad ,\forall k\in\{2,3,4\},\\
\frac{dI^1_{a,v,s}}{dt} &= \frac{4}{q_s}E^4_{a,v,s} - \frac{4}{w}I^1_{a,v,s} + b_{a,v,s}(t) + \Phi_v\omega_a(t)I^1_{a,v=0,s} \quad ,\\
\frac{dI^k_{a,v,s}}{dt} &= \frac{4}{w}I^{k-1}_{a,v,s} - \frac{4}{w}I^k_{a,v,s} + \Phi_v\omega_a(t)I^k_{a,v=0,s} \quad ,\forall k\in\{2,3,4\},\\
\frac{dR_{a,v,s}}{dt} &= \frac{4}{w}I^4_{a,v,s} - \sum_{\sigma\in\mathcal{S}} \lambda_{a,\sigma}(t)\rho_{a,v,\sigma}\chi_{s,\sigma}R_{a,v,s} + \Phi_v\omega_a(t)R_{a,v=0,s} \quad ,
\end{aligned} \tag{A.5}$$

where $\lambda_{a,s}$ represents the force of infection of strain s affecting individuals of age a.
The force of infection was calculated as:

$$\lambda_{a,s}(t) = \beta e^{2W(t)}\psi_s \sum_{\alpha\in\mathcal{A}}\sum_{v\in\mathcal{V}} \frac{c_{a,\alpha}(t)}{N_{\alpha,v}}\Big(0.5\sum_{k=3}^{4}E^k_{\alpha,v,s} + \sum_{k=1}^{4}I^k_{\alpha,v,s}\Big) \quad . \tag{A.6}$$

In the previous equation, β represents the unadjusted risk of transmission per contact, ψ_s is the relative infectiousness of strain s, and $W(t)$ is the random process introduced in Section A.1.2.6. The size of the population of age α with vaccination status v is denoted $N_{\alpha,v}$. The term $c_{a,\alpha}(t)$ is a single element of the contact matrix $C(t)$ introduced in Equation A.3. It represents the average numbers of contacts per day that a individual of age a has with individuals of age α.

A.1.3 Estimation of COVID-19-related hospital pressure and deaths

The transmission model described in Section A.1.2 provides estimates of COVID-19 incidence over time, disaggregated by age, vaccination status and strain. We combine these incidence estimates with the age-, vaccination- and strain-specific risks of hospitalisation and death, as well as statistical distributions of time to events to compute COVID-19-related hospital pressure and deaths over time.

A.1.3.1 COVID-19-related hospital pressure

The risk of hospitalisation given infection was expected to vary markedly by setting. For example, different countries may have different criteria for whether or not a COVID-19 patient should be admitted to a hospital. This makes it difficult to provide accurate estimates of hospitalisation rates for multiple countries.

For this reason we introduced a universal indicator named "hospital occupancy pressure" in our analysis. This indicator was obtained by considering the age-specific risk of hospitalisation given infection observed in the first year of the pandemic in the Netherlands, adjusted for vaccination status and for the infecting strain (Tables B and C). The "hospital occupancy pressure" indicator can therefore be interpreted as the level of hospital occupancy that would be observed in the analysed country if the rates of hospitalisation given infection in this country were the same as for the Netherlands. This quantity is expected to vary proportionately with occupancy over time, providing an indicator of hospital pressure. Note that this indicator was used in order to make comparisons between scenarios, such that one should interpret the relative differences between scenarios rather than the absolute values of the indicator.

Let us denote $i_{a,v,s}(t)$ the number of new disease episodes estimated to start at time t for people aged a with vaccination status v and infected with strain s. The number of new hospital admissions occurring at time t was calculated using the following convolution product:

$$\eta(t) = \sum_{a,v,s} \kappa_{a,v,s} \int_{u \geq 0} i_{a,v,s}(t-u) g_h(u) du \quad , \tag{A.7}$$

where $\kappa_{a,v,s}$ is the risk of hospitalisation given infection for age a, vaccination status v and strain s based on the Netherlands data, and g_h is the probability density function of the statistical distribution chosen to represent the time from symptom onset to hospitalisation (Table B).

We then computed the "hospital occupancy pressure" quantity h, which is an indicator of hospital occupancy level, by combining the number of new hospital admissions η with the statistical distribution used to model hospital stay duration:

$$h(t) = \int_{u \geq 0} \eta(t-u)(1-\tau(u)) du \quad , \tag{A.8}$$

where τ is the cumulative density function of the statistical distribution chosen to represent the hospital stay duration (Table B).

A.1.3.2 COVID-19 deaths

We estimated the number of COVID-19 deaths over time using a similar approach as for the hospital pressure indicator. We used the age-specific infection fatality rates reported in O'Driscoll et al. [33], adjusted for vaccination status and for the infecting strain to estimate COVID-19 mortality. Using the same notation as in Section A.1.3.1, the number of COVID-19 deaths observed at time t was obtained by:

$$\mu(t) = m_C \sum_{a,v,s} ifr_{a,v,s} \int_{u \geq 0} i_{a,v,s}(t-u) g_d(u) du \quad , \tag{A.9}$$

where $ifr_{a,v,s}$ is the risk of death given infection for age a, vaccination status v and strain s, and g_d is the probability density function of the statistical distribution chosen to represent the time from symptom onset to death (Table B). We used the country-specific adjuster m_C to capture the fact that the infection fatality ratio is expected to vary by country, in part due to differences in COVID-19 death definition and reporting standards. This adjustment was automatically calibrated by the MCMC (Section A.3).

A.1.4 Model parameters

A.1.4.1 Parameter tables

The model parameters and their values (or associated prior distributions) are listed in Table B and Table C. The following sections include discussion of the evidence and reasoning used to inform the parameters.

A.1.4.2 Incubation period and active disease duration

A systematic review and meta-analysis was conducted in 2022 attempting to estimate the mean incubation period for different SARS-CoV-2 variants [47]. Their pooled estimate for the mean incubation period for the wild-type variant was 6.65 days (95% CI 6.30-6.99), leveraging estimates from 119 individual studies [47]. The mean incubation period for the Delta variant was found to be 4.41 days (3.76-5.05). The pooled estimate for the mean incubation period for the Omicron variant was 3.42 days (2.88-3.96). These estimates are consistent with evidence from a rapid review that identified shortening of the serial interval of the Delta variant compared to wild-type, and further shortening of the serial interval for the Omicron variant compared to the Delta and wild-type variants [27].

Active disease duration is difficult to estimate for a number of reasons. Observing the period of time over which transmission events occur from a primary case to their secondary cases can be biased towards shorter time periods due to interventions. In particular, case isolation truncates the period at which secondary infections can occur. Proxies of infectiousness such as rapid antigen positivity, PCR or viral culture may not perfectly reflect true infectiousness. Duration of live virus shedding by viable

Table B: Model parameters. Calibrated parameters indicated with ^{C}.

Parameter	Value/Distribution	Evidence
Transmission probability per contact ^{C}	Uniform (0.01, 0.06)	Calibrated
Mean active disease period (days)	4.5	[39, 14, 21]
Country-specific IFR multiplier (m_C) ^{C}	Uniform (0.5, 1.5)	Calibrated
Uncertainty multiplier for school contacts (m_S) ^{C}	Uniform (0.8, 1.2)	Calibrated
Prop. students on-site during "Partially open" periods ^{C}	Uniform (0.1, 0.5)	Calibrated
VE against infection	0.7	[3, 35, 15, 4, 19]
VE against hospitalisation	0.9	[7, 4, 19]
VE against death	0.9	[7, 19]
Time from symptom onset to hospitalisation (days)	Gamma (shape=5, mean=3)	[13]
Hospital stay duration (days)	Gamma (shape=5, mean=9)	[13]
Time from symptom onset to death (days)	Gamma (shape=10, mean=15.93)	[20]
**Strain-specific parameters**		
Wild-type strain		
Mean incubation period (days)	6.65	[47]
Delta variant		
Mean incubation period (days)	4.41	[47]
Relative intrinsic transmissibility (ref. wild-type)	1.5	[22, 29]
Relative risk of hospitalisation (ref. wild-type)	2.0	[9]
Relative risk of death (ref. wild-type)	2.3	[9]
Prop. escaping vaccine immunity against infection	0.3	[17, 26]
Omicron variant		
Mean incubation period (days)	3.42	[47]
Relative intrinsic transmissibility (ref. wild-type)	2.0	[17, 26]
Relative risk of hospitalisation (ref. wild-type)	0.82	[9, 32]
Relative risk of death (ref. wild-type)	0.71	[9, 32]
Prop. escaping vaccine immunity against infection	0.6	[17, 26]

Table C: Age-specific parameters for wild-type COVID-19

Age group	Rel. suscepitbility to infection (ref. 15-69 y.o.) [48]	Proportion symptomatic [37]	Proportion of symptomatic patients hospitalised [36]	Infection fatality rate [33]
0-4	0.36	0.533	0.0777	0.00003
5-9	0.36	0.533	0.0069	0.00001
10-14	0.36	0.533	0.0034	0.00001
15-19	1.00	0.533	0.0051	0.00003
20-24	1.00	0.679	0.0068	0.00006
25-29	1.00	0.679	0.0080	0.00013
30-34	1.00	0.679	0.0124	0.00024
35-39	1.00	0.679	0.0129	0.00040
40-44	1.00	0.679	0.0190	0.00075
45-49	1.00	0.679	0.0331	0.00121
50-54	1.00	0.679	0.0383	0.00207
55-59	1.00	0.679	0.0579	0.00323
60-64	1.00	0.803	0.0617	0.00456
65-69	1.00	0.803	0.1030	0.01075
70-74	1.41	0.803	0.1072	0.01674
75-79	1.41	0.803	0.0703	0.03203
80 and above	1.41	0.803	0.0703	0.08292

viral culture is a better proxy indicator than PCR positivity and duration of live virus shedding is likely the best proxy coupled with epidemiological evidence to indicate the upper limit for duration of infection based on observed secondary cases. In studies that recruited people infected in the community, the median duration of viable viral shedding has been shown to be 4-5 days from symptom onset for the ancestral/wild-type strain [39, 14]. This is coupled with evidence of the possibility of transmission occurring 1-3 days prior to the onset of symptoms places the duration of infection at around 6-7 days on average for the ancestral/wild-type strain [16]. This is supported by evidence of the duration of viable virus detectable in a small number of healthy young adults in a human challenge study being 6.5 days [21].

In our model, the active disease duration parameter defines the time spent in the compartments $I_1 ... I_4$ (Figure B), so we assumed a mean value of 4.5 days for this duration. Moreover, our assumption that infected individuals are infectious during the second half of the incubation period (compartments E_3 and E_4) is consistent with the evidence above.

A.1.4.3 Relative infectiousness of pre-symptomatic period

Evidence indicates that while pre-symptomatic transmission does occur, its contribution is generally smaller compared to symptomatic transmission [10]. Quantifying its exact role remains challenging. A systematic review identified two contact tracing studies, estimating a relative risk of infection from pre-symptomatic individuals at 0.63, though with considerable uncertainty (0.18-2.26) [10]. Similarly, the review included modelling studies estimating that 0-80% of transmission could be attributed to pre-symptomatic individuals. Based on this evidence, we assume a relative infectiousness of 50% for pre-symptomatic cases.

A.1.4.4 Times to hospitalisation and death / hospital stay duration

This section describes the evidence used to inform the statistical distributions listed in Table B regarding times to disease outcomes (hospitalisation and death) and hospital stay duration.

Time from symptom onset to hospitalisation

To inform the mean time from symptom onset to hospitalisation we used the estimate published in the March 2022 International Severe Acute Respiratory and emerging Infections Consortium (ISARIC) Clinical Data Report [13]. ISARIC developed a standardised reporting system to systematically collect, analyse and report on COVID-19 clinical data over the pandemic. Since the start of the pandemic, they have collected clinical data on over 800,000 individuals across 60 countries. The published estimate provided in this latest report and used in our model represents the mean time across all COVID-19 variants and so we believe it is the best representative estimate to use in this analysis, as we assumed this distribution is the same across all SARS-CoV-2 variants modelled.

Hospital stay duration

To inform the mean duration of hospital stay for COVID-19 cases we use the estimate published in the ISARIC Clinical Data Report published March 2022 [13], briefly described above. The published estimate provided in this latest report and used in our model represents the mean time across all COVID-19 variants.

Time from symptom onset to death

The mean time from symptom onset to death was informed by a systematic review and meta-analysis published in 2020 [20]. This pooled estimation was based on three studies that examined the clinical characteristics and outcomes of cases from early outbreaks in China, in 2020.

A.1.4.5 Vaccine efficacy (against wild-type virus)

We aimed to generate an approximate average estimate for vaccine efficacy (VE) against the outcomes below that best represents the protection provided by two doses of the most common vaccine types used globally. First, we were interested in the protection of vaccines against the wild-type virus, and we then considered the different strains' characteristics to determine how vaccine protection should be adjusted for the different viral strains (Section A.1.4.6). We considered studies evaluating the efficacy of adenoviral vector vaccines (namely AZD1222/ChAdOx1 nCoV-19), the mRNA vaccines (BNT162b2 and mRNA-1273) and the inactivated whole-virion vaccines (namely Coronavac, BIBP-CorV and BBV152).

Vaccine efficacy against infection

The results of ChAdOx1 nCoV-19 trial (adenoviral vector vaccine) demonstrated an efficacy against infection of 64.3% (95% CI, 56.1 to 71.0) [8], while data from the mRNA-1273 (mRNA vaccine) trial suggested a VE against infection of 82% (79.5-84.2) against any infection [5]. The BNT162b2 (mRNA vaccine) trial only evaluated the efficacy against symptomatic infection, with a vaccine efficacy of 91.3% (89.0-93.2) [43]. This is similar to the VE of mRNA-1273 against symptomatic infection and so we assume BNT162b2 likely has a similar VE against infection as mRNA-1273 [5, 43]. Inactivated vaccine trials estimated a two-dose VE against infection of 68.8% (46.7–82.5) (BBV152), 64.0% (48.8-74.7) (BIBP-CorV, WIV04 strain) and 73.5% (60.6-82.2) (BIBP-CorV, HB02 strain) [6, 1]. For Coronavac, clinical trials focused on VE against symptomatic infection, with a VE of 83.5% (65.4-92.1), which is similar to the VE against symptomatic infection seen for BBV152 and BIBP-CorV and so we assume that Coronavac has a similar VE against infection [42, 6, 1]. Combining this evidence suggests that 2-dose protection against infection ranges from approximately 60-80% such that we assume that pooled effectiveness falls in the mid-point 70%.

Vaccine efficacy against hospitalisation

This parameter represents the VE against hospitalisation once infected with the wild-type virus. The early trials for two doses (and one dose) of COVID-19 vaccines conducted in 2020 during wild-type circulation were either: 1. Underpowered to precisely estimate the efficacy on the outcome of hospitalisation [8, 42]. Or 2. Evaluated efficacy against severe COVID-19 as a secondary end-point and not explicitly hospitalisation [5, 43, 6, 1]. However, these trials still suggested that the vaccines

significantly protect against hospitalisation and severe disease (a possible proxy of hospitalisation, with point-estimates ranging from 93-100% efficacy [8, 42, 5, 43, 6, 1].

Observational studies conducted in late 2020 and early 2021, prior to the emergence of the Delta variant, estimated VE against hospitalisation of 87% (55-100) for BNT162b2 and 87.5% (86.7–88.2) for Coronavac [4, 19]. We assumed protection against hospitalisation is higher against the wild-type virus than these estimates against variants of concern and so we estimated the VE of 2-doses against hospitalisation at 90% based on the combined evidence.

Vaccine efficacy against death

This parameter represents the VE against death once infected with wild-type virus. The early vaccine trials were underpowered to precisely evaluate the outcome of death; however, as discussed earlier, some demonstrated an effect on severe disease, which precedes COVID-19 death [5, 43, 6, 1].

An observational study in older adults (who have a greater risk death compared to younger ages) in the UK estimated a VE against death for two doses of BNT162b2 of 69% (31-86) following infection [18]. Broader population studies estimated a VE of 84% (44-100) following one dose of BNT162b2 and 86.3% (84.5 to 87.9) following two doses of Coronavac [4, 19]. Furthermore, evaluation of VE against death in Scotland across a broad population following infection with the more severe delta variant estimated a 2-dose VE of 90% (83-94) for BNT162b2 and 91% (86-94) for AZD1222 [38]. Therefore we assume that VE against death following infection with the wild-type virus as modelled in this analysis to be 90% based on the combined evidence.

A.1.4.6 Variant-specific adjustments

A.1.4.6.1 Delta variant

Relative intrinsic transmissibility

Part of the Delta variant's transmission advantage is likely conferred by it being inherently more transmissible than prior circulating variants. Quantifying the extent of this increased transmissibility is difficult in practice because of additional properties around immune evasion of both vaccination and prior infection derived immunity that also give the Delta variant a transmission advantage.

We assumed that the relative intrinsic transmissibility as implemented in the model can be approximated by taking the ratio of the estimates of R_0 for each variant. R_0 is increasingly difficult to estimate for new emerging variants such as the Delta variant due to accumulation of population immunity through vaccination and infection. One study from an outbreak of the Delta variant in a relatively immunologically naive population in Guangdong Province, China, estimated an R_0 of 3.2 [29]. This is approximately 1.5 times that of wild-type estimates from early in the pandemic, $R_0 = 2.2$ (95% CI, 1.4 to 3.9) [22].

Other estimates of the relative effective reproduction number for the Delta variant compared to wild-type report a two-fold increase in transmissibility [2]. However, this did not account for the immune escape advantage that Delta may have, so is still consistent with our assumptions.

Relative intrinsic risk of hospitalisation

A large population-level cohort study in Ontario, Canada found that the adjusted odds of hospitalisation following infection with the Delta variant were 2.08 (1.78-2.40) that of wild-type virus infections [9]. We used the approach set out in [49] to convert the point-estimate of the adjusted odds ratio to an approximate estimate of the relative risk of 2 in the model.

Relative intrinsic risk of death

The same cohort study conducted in Ontario found that the adjusted odds of death following infection with the Delta variant were 2.33 (1.54-3.31) that of wild-type virus infections [9]. Employing the same conversion approach as mentioned in the previous paragraph, we used a relative risk of 2.3 in the model.

Immune escape property

Part of the Delta variant's transmission advantage is likely conferred by its ability to evade vaccine-induced immunity. In particular there is evidence for reduced activity in immune correlates for protection against infection, such as neutralising antibody titres [34]. However, it is less clear how this translates into VE against infection. One population-level observational study in the UK estimated two doses of either an mRNA or adenoviral vector vaccine as being approximately 10% less effective at preventing

symptomatic COVID-19 infection for the Delta variant compared to the Alpha variant [24]. However in highly-vaccinated populations this is likely an estimate of the combined effect that includes the protection of the vaccine against onward transmission in infected, which we do not capture in this model. The best estimate we have found for VE against Delta variant infection from unvaccinated source comes from a Danish household transmission study and estimates a VE of 46% (40-52) for two doses of an mRNA or adenoviral vector vaccine [25]. Similarly, a household transmission study in Norway estimated the VE against infection for 2-dose-mRNA-vaccinated individuals following contact with a Delta household contact was 42% (23-55) [17]. These estimates are about 60-66% of our assumed modelled VE of 70% based on prior evidence from vaccine trials (Section A.1.4.5). Given the uncertainty, we assumed a conservative estimate that the Delta variant is able to escape 30% of prior vaccine immunity against infection.

A.1.4.6.2 Omicron variant

Relative intrinsic transmissibility
As described above for the Delta variant, the Omicron variant likely has a combined transmission advantage due to increased intrinsic transmissibility and its ability to evade prior immunity from infection and vaccination. R_0 estimates for the Omicron variant are very difficult due to the high levels of population immunity from infection and vaccination in nearly every setting at the time of its emergence. However, estimates of the risk of infection in unvaccinated close contacts of individuals infected with the Omicron variant compared to other variants should also allow us to estimate the change in relative intrinsic transmissibility. These estimates largely only exist comparing the first Omicron subvariant BA.1 against the Delta variant and do not exist comparing Omicron directly to wild-type. We can, however, consider the product of our relative intrinsic transmissibility for the Delta variant compared to wild-type and our estimate of the relative intrinsic transmissibility for the Omicron variant compared to Delta.

A household transmission study in Norway found that unvaccinated household contacts of primary cases infected with Omicron BA.1 had a risk of being infected that was 1.27 times that of unvaccinated household contacts of a Delta infected primary case [17]. However, susceptibility is just one component of transmissibility as infectiousness also plays a role. The same study estimated that household close contacts of unvaccinated Omicron BA.1 primary cases had a relative risk of being infected 1.5 times that of households close contacts of unvaccinated Delta primary cases [17]. This suggests that the Omicron BA.1 variant was between 1.2-1.5 times as transmissible as the Delta variant.

A household transmission study in Norway found evidence that suggest people are more susceptible and more infectious with Omicron variant compared to the Delta variant [17]. Unvaccinated household contacts of primary cases infected with Omicron BA.1 had a relative risk 1.27 times that of unvaccinated household contacts of a Delta primary infected case [17]. Similarly, they also found that household contacts of unvaccinated Omicron BA.1 primary cases had a relative risk 1.5 times that of household close contacts of unvaccinated Delta primary cases [17]. This suggests that the Omicron BA.1 variant is between 1.2-1.5 times as transmissible as the Delta variant.

Therefore, in this model we assumed the modelled Omicron variant is 2 times (1.3, (mid-point of 1.2-1.5) multiplied by 1.5 (the relative intrinsic transmissibility of the Delta variant)) as intrinsically transmissible as the wild-type virus.

Relative intrinsic risk of hospitalisation
We could not find clear estimates of the relative intrinsic risk of hospitalisation for Omicron variant infection compared to the wild-type virus because of the issues raised in the previous paragraph. However, we could find estimates for the relative risk of Omicron compared to Delta and thus can use the product of this estimate with our estimate of the relative risk of hospitalisation with the Delta variant compared to the wild-type virus. A large population-level study in England found an adjusted hazard ratio for hospitalisation of 0.41 (0.39-0.43) for infection with the Omicron variant compared to the Delta variant [32]. We assumed that this hazard ratio can be used to estimate the relative risk and thus our estimate for the relative intrinsic risk of hospitalisation for Omicron variant infection compared to the wild-type was 0.82 (0.41×2).

Relative intrinsic risk of death
To estimate the relative risk of death for Omicron compared to wild-type virus, we used the same ap-

proach as described above for relative risk of hospitalisation. We leveraged estimates relevant to the Delta variant to find the approximate estimate for this parameter in the model. A large population-level study in England found an adjusted hazard ratio for hospitalisation of 0.31 (0.26-0.37) for infection with the Omicron variant compared to the Delta variant [32]. We assumed that this hazard ratio can be used to estimate the relative risk and thus our estimate for the relative intrinsic risk of hospitalisation for Omicron variant infection compared to the wild-type is 0.71 (0.31×2.3).

Immune escape property
In vitro neutralisation studies leveraging sera from vaccinated individuals demonstrated substantial immune escape of Omicron BA.1 compared to the Delta variant [45]. The best estimate of VE against infection we could find was from a household transmission study in Norway which estimated a VE against infection with the Omicron variant of 27% (6-49) for 2-dose-vaccinated individuals [17]. This is about 65% of their estimated VE against the Delta variant and about 38% of our modelled VE for the wild-type virus of 70% [17]. We therefore assumed that the Omicron variant in the model is able to escape 60% of vaccine immunity.

A.2 Software and code used to conduct the analyses

A.2.1 Code

The Python code and data used to perform the analyses is fully available on Github at the following link: `https://github.com/monash-emu/covid19_school_closures`. In particular, the code associated with the implementation of the model is available here. The suite of functions used to run the model, perform calibration and analyse the outputs is available here. Finally, the model outputs can be explored in detail for all analyses via an online interactive interface, without any local software installation requirement.

We also provide detailed instructions on how to install and run our model locally on the project's GitHub webpage.

A.2.2 Software implementation

We used the *summer* Python package (v 1.2.5) to implement the model [40]. This is a domain-specific library for compartmental epidemiological modelling enabling highly expressive programming and accelerated computation through Google's *jax* library

A.2.2.1 Application Programming Interface (API)

The model specification was done via a simple yet expressive Python API, while the numerical implementation was autogenerated by the *summer* package at runtime. This specification is composable via stratification classes and other reusable components, such that the complexity of the software is kept to a minimum, reducing cognitive overhead and greatly reducing the possibility for error.

A.2.2.2 Optimising compiler

The *summer* package uses the jax library as its computational backend, meaning that while the specification of models is done largely in Python, the model execution itself is transformed via an optimising compiler into fast native code. This brings the model runtime from several seconds (for a naive implementation) to under 50ms per iteration, which was necessary to perform the computationally-intensive calibration tasks described in Section A.3.

A.3 Model calibration and uncertainty propagation

The model was calibrated using a Bayesian approach. In particular, we used the Adaptive Differential Evolution Metropolis (DEMetropolisZ) algorithm implemented with the *PyMC* Python package (v.5.2.0) to sample parameters from their posterior distributions. For each country, we ran 8 independent DEMetropolisZ chains of 35,000 iterations, each starting from a different starting point and using the first 5,000 draws for algorithm tuning. Sampling convergence was assessed by analysing all parameter

traces visually and by requiring that the Gelman-Rubin statistic was below 1.05. To determine the 8 starting points of the DEMetropolisZ chains, we conducted 8 independent optimisation searches using the Covariance Matrix Adaptation Evolution Strategy (CMA-ES) method, implemented with the *nevergrad* Python package (v.0.6.0) and with a budget of 10,000 model evaluations per search. The 8 optimisation searches' initial points were randomly drawn from Latin Hypercube Sampling (LHS) based on the parameter priors shown in Table B. The parameters sampled with LHS were the transmission probability per contact, the IFR multiplier, the uncertainty multiplier for school contacts, and the proportion of students on-site during "Partially open" periods. The random process variables $W(t)$ (Section A.1.2.6) were all set to 0 for each optimisation starting point.

Our calibration approach required a total of 360,000 ($8 \times (10000 + 35000)$) model evaluations per country analysis, which were completed in about two hours on a machine with 8 cpus and 32-GiB memory.

For each country, the results presented in the manuscript are associated with 1000 parameter sets randomly sampled from the posterior distributions obtained from DEMetropolisZ sampling (after discarding the first 25,000 iterations for each chain). The definitions of the prior distributions and the likelihood are detailed in the following sections.

A.3.1 Parameters varied during calibration

The parameters varied during calibration along with their associated prior distributions are listed in Table B and indicated with the superscript ^{C}. We used uniform prior distributions for all calibrated parameters. The primary parameters varied during calibration are the unadjusted risk of transmission per contact (β), the IFR multiplier (m_C), the original infection seeding time, the proportion of students on-site during "Partially open" periods, and the uncertainty multiplier modifying the school contacts' contribution (m_S).

Note that the values of the random process W_t described in Section A.1.2.6 are also treated as calibrated parameters by the MCMC. The Gaussian auto-regressive component described in Equation A.4 is incorporated in the posterior likelihood computation (Section A.3.3).

A.3.2 Calibration targets

Model calibration was performed independently for each country. All models were fitted to the reported number of COVID-19 deaths over time. We used the daily number of COVID-19 deaths reported by WHO and applied a 7-day moving average to the observed data.

In addition, for countries where a nationally representative seroprevalence survey had been conducted (n=36), we include seroprevalence data in the calibration likelihood. We used the online platform SeroTracker to extract country-specific seroprevalence estimates. Seroprevalence estimates had to verify the following conditions to be included in the analysis as calibration targets:

- Be aligned with the World Health Organization's Unity protocol (WHO Unity) for general population seroepidemiological studies.
- Have a sample size greater than 599 (minimum sample size recommended in WHO sero-surveys protocol)
- Have a sampling start date later than 1 May 2020 (to avoid very early surveys that may be less accurate)
- Be nationally representative (as classified by SeroTracker)

We further excluded studies that focused on specific population subgroups presenting a risk of selection bias for seroprevalence (e.g. pregnant women, slum population, healthcare workers, quarantine workers). Finally, to minimise the risk of interference with vaccination, we only included studies for which the vaccination coverage at the time of the survey (midpoint date) was lower than 10% of the measured seroprevalence.

All the criteria listed above were first verified systematically using the SeroTracker database, and the extracted studies were then analysed individually by one author (RR) to check that all inclusion criteria were verified.

Where a seroprevalence study was restricted to a specific age-group, we matched the survey estimate to the modelled seroprevalence measured in the closest modelled age-group. For example, as the age-group reported in the Kenya survey was 16-64 years old, we used the modelled seroprevalence in the age-group 15-69 years-old to inform model calibration.

When multiple estimates were available for a country, we selected the highest ranked estimate after ordering by the following preference criteria (applied in the presented order):

1. Lowest risk of bias (according to SeroTracker),
2. Latest sampling start date (when a greater number of infections have occurred, and to avoid bias due to early geographic heterogeneity),

The estimates used to inform the models are summarised in Table D, with the associated original reports accessible by clicking on the countries' names.

A.3.3 Likelihood definition

Let d_w denote the rounded average daily number of COVID-19 deaths during week w, and $\hat{d_w}^\theta$ the associated predicted number of deaths according to the model with parameter set θ.

For countries with seroprevalence data, let us denote π the measured seroprevalence proportion extracted from SeroTracker (Section A.3.2). Let $\hat{\pi}^\theta$ denote the modelled age-matched proportion ever infected by the time the survey was conducted (using the midpoint date) associated with the parameter set θ. The likelihood was defined as follows for countries with seroprevalence data:

$$\mathcal{L}(\theta) := f_\sigma(\pi|\hat{\pi}^\theta) \times \prod_w g_r(d_w|\,\hat{d_w}^\theta) \quad , \tag{A.10}$$

where $f_\sigma(.|\mu)$ is the probability density function of a $[0,1]$-truncated normal distribution with mean μ and standard deviation σ; and $g_r(.|\mu)$ is the probability mass function of a negative binomial distribution with mean μ and overdispersion parameter r. The overdispersion parameter r was automatically estimated by the MCMC algorithm, while the standard deviation σ was set to different values depending on the SeroTracker-reported risk of bias associated with the seroprevalence estimate ($\sigma = 0.05$ if "Low", $\sigma = 0.1$ if "Moderate", $\sigma = 0.2$ if "High").

For countries without seroprevalence data, the likelihood equation reduces to:

$$\mathcal{L}(\theta) := \prod_w g_r(d_w|\,\hat{d_w}^\theta) \quad . \tag{A.11}$$

The likelihood functions described above represent the goodness of fit of a particular model parameterisation with regards to the targeted data. This quantity needs to be adjusted for the prior likelihood of the parameter set in order to compute the MCMC acceptance quantity $\mathcal{Q}(\theta)$. As we used uniform priors for all the parameters, the inclusion of the individual parameters' priors in the acceptance quantity is not necessary. Indeed, their respective contributions would cancel out as the same quantity would appear in the numerator and the denominator of the MCMC acceptance quantity ratio. However, the auto-regressive relationship described in Equation A.4 must be accounted for as part of the combined prior likelihood of a parameter set. This favours smaller fluctuations of the random process. If W^θ represents the random process associated with the parameter set θ, the overall MCMC acceptance quantity is obtained by:

$$\mathcal{Q}(\theta) = \mathcal{L}(\theta) \times \prod_{i=1}^{n} z_{W^\theta_{i-1},\epsilon}(W^\theta_i) \quad , \tag{A.12}$$

where $z_{\mu,\epsilon}(.)$ represents the probability density function of the normal distribution $\mathcal{N}(\mu,\epsilon)$, and n is the number of random process updates.

Table D: Seroprevalence data extracted from SeroTracker (national surveys).

**country**	**sampling start date**	**sampling end date**	**age min**	**age max**	**denom. value**	**serum pos preva-lence**	**estimate grade**	**overall risk of bias**
Australia	2020-11-03	2021-03-12		19	1685	0.23%	National	High
Austria	2020-06-05	2020-12-04	18	72	20228	2.5%	National	High
Belgium	2020-10-12	2020-10-17		101	2966	4.18%	National	Low
Brazil	2020-05-14	2020-06-23			89362	2.3%	National	Moderate
Canada	2021-04-13	2021-04-30	17		16931	26.92%	National	Moderate
Chile	2020-09-25	2020-11-25	7	94	2493	10.4%	National	Low
Colombia	2020-09-21	2020-12-11	5	80	17863	32.53%	National	Moderate
Croatia	2020-12-15	2021-02-15			1436	25.1%	National	High
Czechia	2021-02-01	2021-03-31	18		19548	51.0%	National	High
Denmark	2020-12-01	2020-12-31	12		4044	4.3%	National	Low
Ecuador	2020-10-12	2020-10-19			1250	11.68%	National	Moderate
Egypt	2021-01-15	2021-06-15			2360	46.3%	National	Moderate
France	2020-05-11	2020-05-17			3592	4.93%	National	Low
Germany	2020-10-26	2020-11-18	18		9929	1.1%	National	Moderate
Honduras	2020-06-16	2020-06-23	5		792	6.2%	National	Moderate
Hungary	2020-05-01	2020-05-16	14		10474	0.68%	National	Low
India	2020-12-18	2021-01-06	10		28598	24.1%	National	Low
Israel	2020-06-28	2020-09-14			54357	4.6%	National	Moderate
Italy	2020-05-25	2020-07-15			64660	2.5%	National	Moderate
Japan	2020-06-01	2020-06-07	20		7950	0.1%	National	Moderate
Jordan	2020-12-27	2021-01-06			5044	34.2%	National	Moderate
Kazakhstan	2020-07-16	2021-07-07			85346	63.0%	National	High
Kenya	2021-01-03	2021-03-15	16	64	3018	48.5%	National	Moderate
Lebanon	2020-12-07	2021-01-15			2058	18.5%	National	Low
Lithuania	2020-08-10	2020-09-10	18	92	3089	1.4%	National	Moderate
Mexico	2020-08-15	2020-11-15	3	12	944	18.7%	National	Low
Nepal	2020-10-09	2020-10-22			3040	14.4%	National	Low
Pakistan	2020-10-21	2020-11-08			4998	7.02%	National	Moderate
Portugal	2020-09-08	2020-10-14			13398	2.2%	National	Moderate
Slovenia	2020-10-17	2020-11-10		99	1211	4.29%	National	Low
South Africa	2021-01-15	2021-05-15	15	69	16762	47.4%	National	Moderate
Rep. of Korea	2020-09-24	2020-12-09	18	86	4085	0.39%	National	Moderate
Spain	2020-06-08	2020-06-22			62167	5.2%	National	Low
Sweden	2020-11-23	2020-12-04			3183	7.0%	National	Moderate
USA	2020-08-09	2020-12-08	18		4654	4.71%	National	Low
UK	2020-08-24	2020-09-18	17		8230	6.1%	National	Low

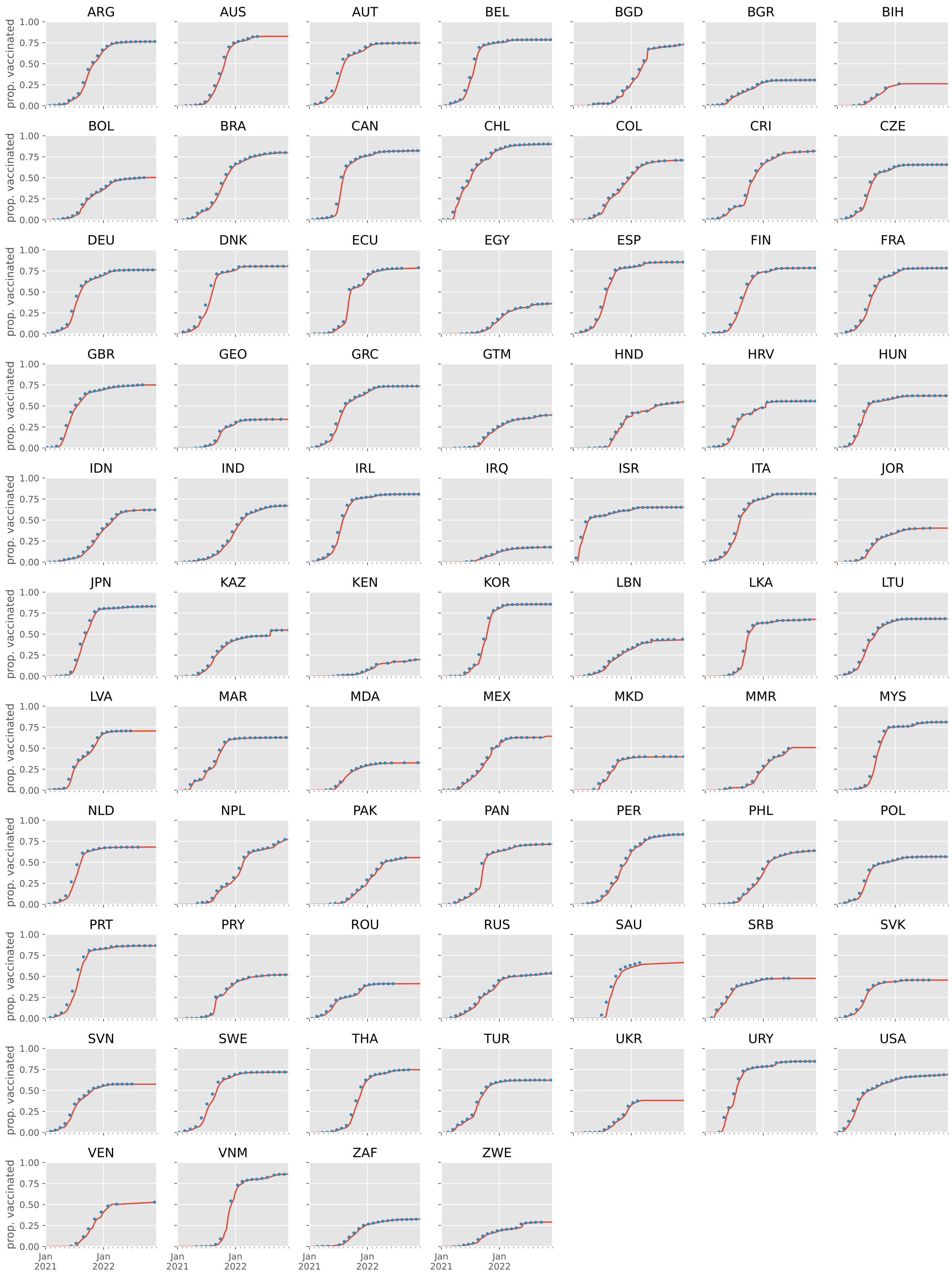

Fig D: Modelled vaccine coverage (lines) against data (dots).

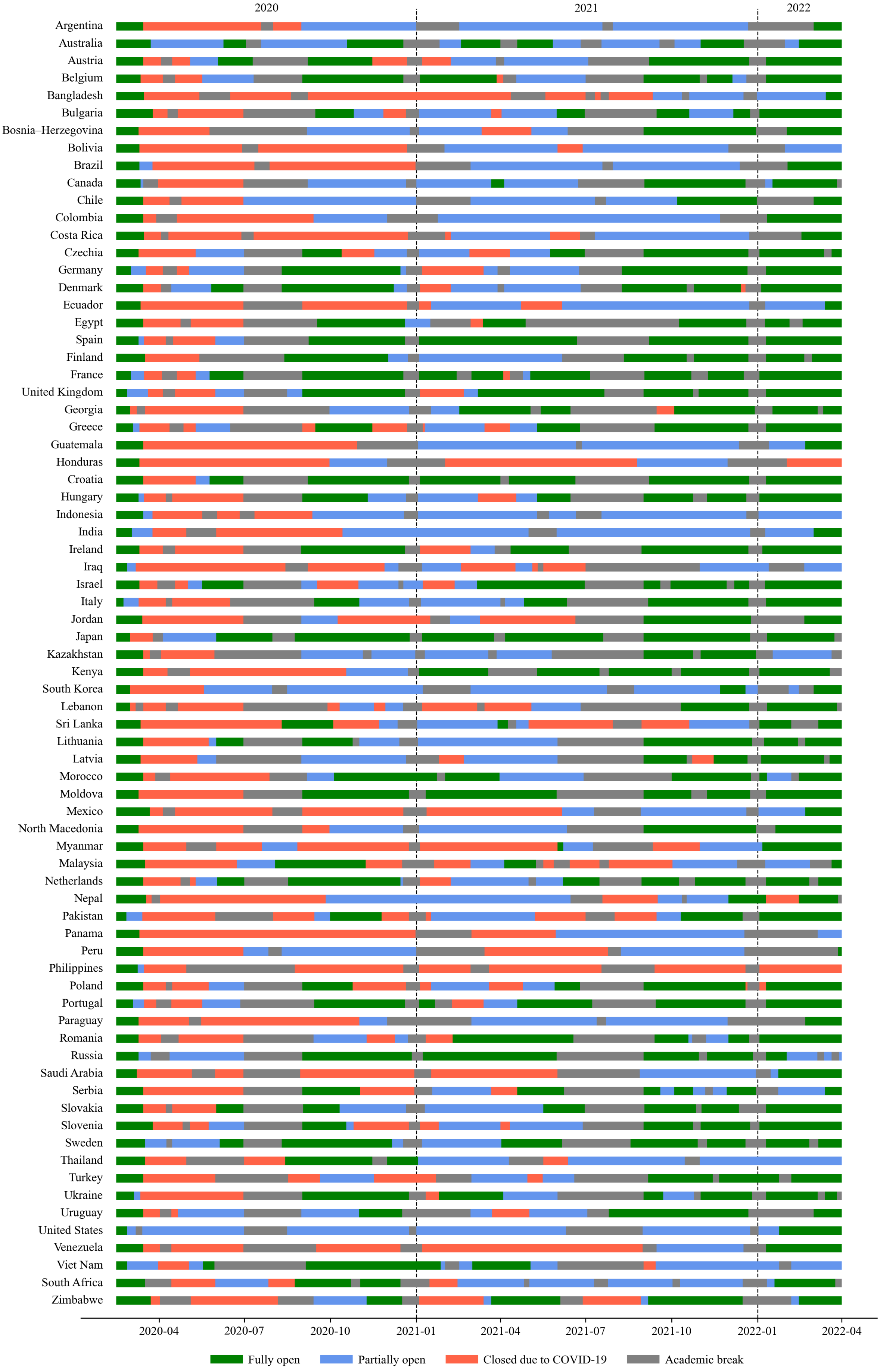

Fig E: UNESCO data on school closures during the COVID-19 pandemic.

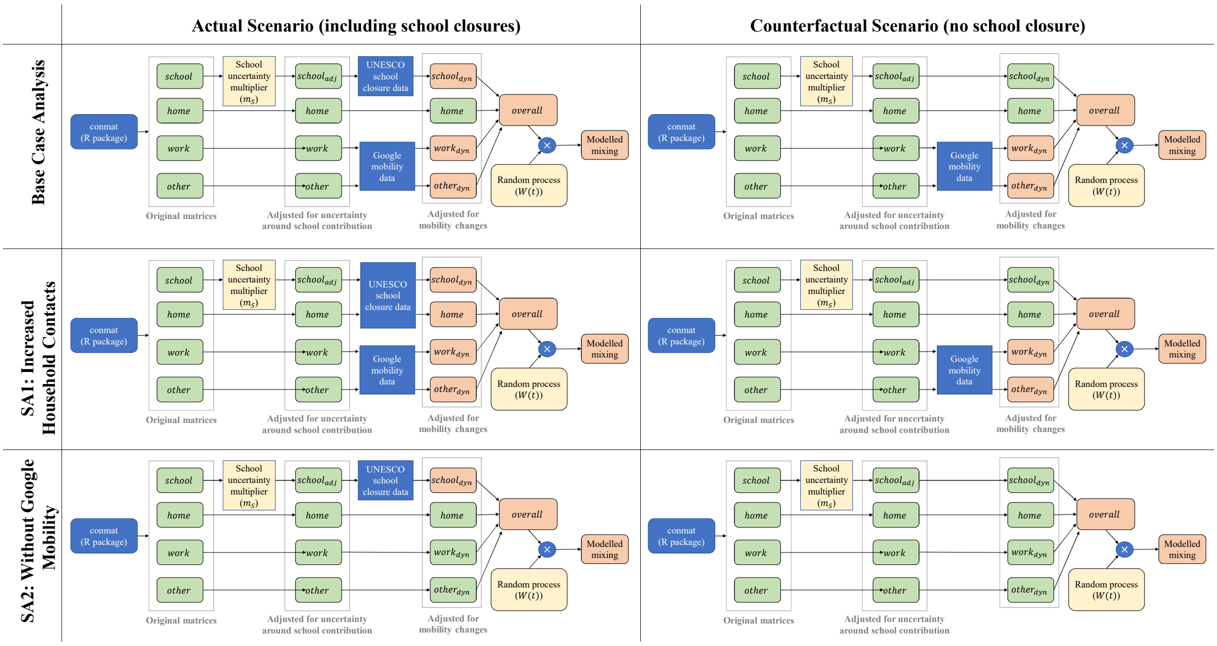

Fig F: Modelled social mixing under Base Case and Sensitivity Analyses.

Bibliography

[1] Nawal Al Kaabi et al. "Effect of 2 Inactivated SARS-CoV-2 Vaccines on Symptomatic COVID-19 Infection in Adults: A Randomized Clinical Trial". In: *JAMA* 326.1 (2021), pp. 35–45. DOI: 10.1001/jama.2021.8565. URL: https://doi.org/10.1001/jama.2021.8565.

[2] Finlay Campbell et al. "Increased transmissibility and global spread of SARS-CoV-2 variants of concern as at June 2021". In: *Eurosurveillance* 26.24 (2021), p. 2100509. DOI: doi:https://doi.org/10.2807/1560-7917.ES.2021.26.24.2100509. URL: https://www.eurosurveillance.org/content/10.2807/1560-7917.ES.2021.26.24.2100509.

[3] Tiffany Charmet et al. "Impact of original, B.1.1.7, and B.1.351/P.1 SARS-CoV-2 lineages on vaccine effectiveness of two doses of COVID-19 mRNA vaccines: Results from a nationwide case-control study in France". In: *The Lancet Regional Health – Europe* 8 (2021). DOI: 10.1016/j.lanepe.2021.100171. URL: https://doi.org/10.1016/j.lanepe.2021.100171.

[4] Noa Dagan et al. "BNT162b2 mRNA Covid-19 Vaccine in a Nationwide Mass Vaccination Setting". In: *New England Journal of Medicine* 384.15 (2021), pp. 1412–1423. DOI: 10.1056/NEJMoa2101765. URL: https://doi.org/10.1056/NEJMoa2101765.

[5] Hana M. El Sahly et al. "Efficacy of the mRNA-1273 SARS-CoV-2 Vaccine at Completion of Blinded Phase". In: *New England Journal of Medicine* 385.19 (2021), pp. 1774–1785. DOI: 10.1056/NEJMoa2113017. URL: https://doi.org/10.1056/NEJMoa2113017.

[6] Raches Ella et al. "Efficacy, safety, and lot-to-lot immunogenicity of an inactivated SARS-CoV-2 vaccine (BBV152): interim results of a randomised, double-blind, controlled, phase 3 trial". In: *The Lancet* 398.10317 (2021), pp. 2173–2184. DOI: 10.1016/S0140-6736(21)02000-6. URL: https://doi.org/10.1016/S0140-6736(21)02000-6.

[7] Public Health England. *COVID-19 vaccine surveillance report - Week 26*. Report. Jan. 2021. URL: https://www.gov.uk/government/publications/covid-19-vaccine-surveillance-report.

[8] Ann R. Falsey et al. "Phase 3 Safety and Efficacy of AZD1222 (ChAdOx1 nCoV-19) Covid-19 Vaccine". In: *New England Journal of Medicine* 385.25 (2021), pp. 2348–2360. DOI: 10.1056/NEJMoa2105290. URL: https://doi.org/10.1056/NEJMoa2105290.

[9] David N. Fisman and Ashleigh R. Tuite. "Evaluation of the relative virulence of novel SARS-CoV-2 variants: a retrospective cohort study in Ontario, Canada". In: *Canadian Medical Association Journal* 193.42 (2021), E1619. DOI: 10.1503/cmaj.211248. URL: http://www.cmaj.ca/content/193/42/E1619.abstract.

[10] W. Gao et al. "Role of asymptomatic and pre-symptomatic infections in covid-19 pandemic". In: *BMJ* 375 (2021), n2342. DOI: 10.1136/bmj.n2342. URL: https://www.ncbi.nlm.nih.gov/pubmed/34852994.

[11] GISAID. *GISAID database*. Web Page. 2023. URL: https://gisaid.org/.

[12] *GitHub repository of the conmat R package to generate synthetic contact matrices*. Web Page. URL: https://github.com/idem-lab/conmat.

[13] Isaric Clinical Characterisation Group et al. "ISARIC COVID-19 Clinical Data Report issued: 27 March 2022". In: *medRxiv* (2022), p. 2020.07.17.20155218. DOI: 10.1101/2020.07.17.20155218. URL: http://medrxiv.org/content/early/2022/04/13/2020.07.17.20155218.abstract.

[14] Seran Hakki et al. "Onset and window of SARS-CoV-2 infectiousness and temporal correlation with symptom onset: a prospective, longitudinal, community cohort study". In: *The Lancet Respiratory Medicine* 10.11 (2022), pp. 1061–1073. DOI: 10.1016/S2213-2600(22)00226-0. URL: https://doi.org/10.1016/S2213-2600(22)00226-0.

[15] Victoria Jane Hall et al. "COVID-19 vaccine coverage in health-care workers in England and effectiveness of BNT162b2 mRNA vaccine against infection (SIREN): a prospective, multicentre, cohort study". In: *The Lancet* 397.10286 (2021), pp. 1725–1735. DOI: 10.1016/S0140-6736(21)00790-X. URL: https://doi.org/10.1016/S0140-6736(21)00790-X.

[16] X. He et al. "Temporal dynamics in viral shedding and transmissibility of COVID-19". In: *Nat Med* 26.5 (2020), pp. 672–675. DOI: 10.1038/s41591-020-0869-5. URL: https://www.ncbi.nlm.nih.gov/pubmed/32296168.

[17] Neda Jalali et al. "Increased household transmission and immune escape of the SARS-CoV-2 Omicron compared to Delta variants". In: *Nature Communications* 13.1 (2022), p. 5706. DOI: 10.1038/s41467-022-33233-9. URL: https://doi.org/10.1038/s41467-022-33233-9.

[18] Bernal Jamie Lopez et al. "Effectiveness of BNT162b2 mRNA vaccine and ChAdOx1 adenovirus vector vaccine on mortality following COVID-19". In: *medRxiv* (2021), p. 2021.05.14.21257218. DOI: 10.1101/2021.05.14.21257218. URL: http://medrxiv.org/content/early/2021/05/18/2021.05.14.21257218.abstract.

[19] Alejandro Jara et al. "Effectiveness of an Inactivated SARS-CoV-2 Vaccine in Chile". In: *New England Journal of Medicine* 385.10 (2021), pp. 875–884. DOI: 10.1056/NEJMoa2107715. URL: https://doi.org/10.1056/NEJMoa2107715.

[20] Malahat Khalili et al. "Epidemiological characteristics of COVID-19: a systematic review and meta-analysis". In: *Epidemiology and Infection* 148 (2020), e130. DOI: 10.1017/S0950268820001430. URL: https://www.cambridge.org/core/article/epidemiological-characteristics-of-covid19-a-systematic-review-and-metaanalysis/8B565B2FE5A97054E8B2564FB2CE6D3E.

[21] Ben Killingley et al. "Safety, tolerability and viral kinetics during SARS-CoV-2 human challenge in young adults". In: *Nature Medicine* 28.5 (2022), pp. 1031–1041. DOI: 10.1038/s41591-022-01780-9. URL: https://doi.org/10.1038/s41591-022-01780-9.

[22] Q. Li et al. "Early Transmission Dynamics in Wuhan, China, of Novel Coronavirus-Infected Pneumonia". In: *N Engl J Med* 382.13 (2020), pp. 1199–1207. DOI: 10.1056/NEJMoa2001316. URL: https://www.ncbi.nlm.nih.gov/pubmed/31995857.

[23] Google LLC. *Google COVID-19 Community Mobility Reports.* Web Page. URL: https://www.google.com/covid19/mobility/.

[24] Jamie Lopez Bernal et al. "Effectiveness of Covid-19 Vaccines against the B.1.617.2 (Delta) Variant". In: *New England Journal of Medicine* 385.7 (2021), pp. 585–594. DOI: 10.1056/NEJMoa2108891. URL: https://doi.org/10.1056/NEJMoa2108891.

[25] Frederik Plesner Lyngse et al. "Effect of vaccination on household transmission of SARS-CoV-2 Delta variant of concern". In: *Nature Communications* 13.1 (2022), p. 3764. DOI: 10.1038/s41467-022-31494-y. URL: https://doi.org/10.1038/s41467-022-31494-y.

[26] Frederik Plesner Lyngse et al. "Household transmission of the SARS-CoV-2 Omicron variant in Denmark". In: *Nature Communications* 13.1 (2022), p. 5573. DOI: 10.1038/s41467-022-33328-3. URL: https://doi.org/10.1038/s41467-022-33328-3.

[27] Zachary J. Madewell et al. "Rapid review and meta-analysis of serial intervals for SARS-CoV-2 Delta and Omicron variants". In: *BMC Infectious Diseases* 23.1 (2023), p. 429. DOI: 10.1186/s12879-023-08407-5. URL: https://doi.org/10.1186/s12879-023-08407-5.

[28] Edouard Mathieu et al. "A global database of COVID-19 vaccinations". In: *Nature Human Behaviour* 5.7 (2021), pp. 947–953. DOI: 10.1038/s41562-021-01122-8. URL: https://doi.org/10.1038/s41562-021-01122-8.

[29] Zhang Meng et al. "Transmission Dynamics of an Outbreak of the COVID-19 Delta Variant B.1.617.2 — Guangdong Province, China, May–June 2021". In: *China CDC Weekly* 3.27 (2021), pp. 584–586. DOI: 10.46234/ccdcw2021.148. URL: https://weekly.chinacdc.cn//article/id/eb772589-1584-4ef9-beac-cac3ab2fbb12.

[30] D. Mistry et al. “Inferring high-resolution human mixing patterns for disease modeling”. In: *Nat Commun* 12.1 (2021), p. 323. DOI: 10.1038/s41467-020-20544-y. URL: https://www.ncbi.nlm.nih.gov/pubmed/33436609.

[31] Joël Mossong et al. “Social Contacts and Mixing Patterns Relevant to the Spread of Infectious Diseases”. In: *PLOS Medicine* 5.3 (2008), e74. DOI: 10.1371/journal.pmed.0050074. URL: https://doi.org/10.1371/journal.pmed.0050074.

[32] T. Nyberg et al. “Comparative analysis of the risks of hospitalisation and death associated with SARS-CoV-2 omicron (B.1.1.529) and delta (B.1.617.2) variants in England: a cohort study”. In: *Lancet* 399.10332 (2022), pp. 1303–1312. DOI: 10.1016/s0140-6736(22)00462-7.

[33] M. O’Driscoll et al. “Age-specific mortality and immunity patterns of SARS-CoV-2”. In: *Nature* 590.7844 (2021), pp. 140–145. DOI: 10.1038/s41586-020-2918-0. URL: https://www.ncbi.nlm.nih.gov/pubmed/33137809.

[34] Eddy Pérez-Then et al. “Neutralizing antibodies against the SARS-CoV-2 Delta and Omicron variants following heterologous CoronaVac plus BNT162b2 booster vaccination”. In: *Nature Medicine* 28.3 (2022), pp. 481–485. DOI: 10.1038/s41591-022-01705-6. URL: https://doi.org/10.1038/s41591-022-01705-6.

[35] Emma Pritchard et al. “Impact of vaccination on new SARS-CoV-2 infections in the United Kingdom”. In: *Nature Medicine* 27.8 (2021), pp. 1370–1378. DOI: 10.1038/s41591-021-01410-w. URL: https://doi.org/10.1038/s41591-021-01410-w.

[36] Netherlands RIVM. *Epidemiologische situatie COVID-19 in Nederland.* Government Document. 2020. URL: https://www.rivm.nl/sites/default/files/2020-08/COVID-19_WebSite_rapport_wekelijks_20200804_1306.pdf.

[37] Pratha Sah et al. “Asymptomatic SARS-CoV-2 infection: A systematic review and meta-analysis”. In: *Proceedings of the National Academy of Sciences* 118.34 (2021), e2109229118. DOI: 10.1073/pnas.2109229118. URL: http://www.pnas.org/content/118/34/e2109229118.abstract.

[38] Aziz Sheikh, Chris Robertson, and Bob Taylor. “BNT162b2 and ChAdOx1 nCoV-19 Vaccine Effectiveness against Death from the Delta Variant”. In: *New England Journal of Medicine* 385.23 (2021), pp. 2195–2197. DOI: 10.1056/NEJMc2113864. URL: https://doi.org/10.1056/NEJMc2113864.

[39] Anika Singanayagam et al. “Duration of infectiousness and correlation with RT-PCR cycle threshold values in cases of COVID-19, England, January to May 2020”. In: *Eurosurveillance* 25.32 (2020), p. 2001483. DOI: doi:https://doi.org/10.2807/1560-7917.ES.2020.25.32.2001483. URL: https://www.eurosurveillance.org/content/10.2807/1560-7917.ES.2020.25.32.2001483.

[40] *Software packages and related resources produced by the Monash Epidemiological Modelling Unit.* Web Page. URL: https://monash-emu.github.io/.

[41] Caroline Stein et al. “Past SARS-CoV-2 infection protection against re-infection: a systematic review and meta-analysis”. In: *The Lancet* 401.10379 (2023), pp. 833–842. DOI: 10.1016/S0140-6736(22)02465-5. URL: https://doi.org/10.1016/S0140-6736(22)02465-5.

[42] Mine Durusu Tanriover et al. “Efficacy and safety of an inactivated whole-virion SARS-CoV-2 vaccine (CoronaVac): interim results of a double-blind, randomised, placebo-controlled, phase 3 trial in Turkey”. In: *The Lancet* 398.10296 (2021), pp. 213–222. DOI: 10.1016/S0140-6736(21)01429-X. URL: https://doi.org/10.1016/S0140-6736(21)01429-X.

[43] Stephen J. Thomas et al. “Safety and Efficacy of the BNT162b2 mRNA Covid-19 Vaccine through 6 Months”. In: *New England Journal of Medicine* 385.19 (2021), pp. 1761–1773. DOI: 10.1056/NEJMoa2110345. URL: https://doi.org/10.1056/NEJMoa2110345.

[44] UNESCO. *UNESCO Global Monitoring of School Closures Caused by the COVID-19 Pandemic.* Web Page. 2023. URL: https://covid19.uis.unesco.org/global-monitoring-school-closures-covid19/.

[45] Brian J. Willett et al. “SARS-CoV-2 Omicron is an immune escape variant with an altered cell entry pathway”. In: *Nature Microbiology* 7.8 (2022), pp. 1161–1179. DOI: 10.1038/s41564-022-01143-7. URL: https://doi.org/10.1038/s41564-022-01143-7.

[46] Organization World Health. *WHO SAGE roadmap for prioritizing the use of COVID-19 vaccines in the context of limited supply: an approach to inform planning and subsequent recommendations based upon epidemiologic setting and vaccine supply scenarios, 13 November 2020.* Report. World Health Organization, 2020 2020. URL: https://iris.who.int/handle/10665/341448.

[47] Yu Wu et al. "Incubation Period of COVID-19 Caused by Unique SARS-CoV-2 Strains: A Systematic Review and Meta-analysis". In: *JAMA Network Open* 5.8 (2022), e2228008–e2228008. DOI: 10.1001/jamanetworkopen.2022.28008. URL: https://doi.org/10.1001/jamanetworkopen.2022.28008.

[48] J. Zhang et al. "Changes in contact patterns shape the dynamics of the COVID-19 outbreak in China". In: *Science* 368.6498 (2020), pp. 1481–1486. DOI: 10.1126/science.abb8001. URL: https://www.ncbi.nlm.nih.gov/pubmed/32350060.

[49] Jun Zhang and Kai F. Yu. "What's the Relative Risk?A Method of Correcting the Odds Ratio in Cohort Studies of Common Outcomes". In: *JAMA* 280.19 (1998), pp. 1690–1691. DOI: 10.1001/jama.280.19.1690. URL: https://doi.org/10.1001/jama.280.19.1690.

Part B

Additional results

B.1 Exploring the findings heterogeneity

We observed significant heterogeneity in the estimated effects of school closures on SARS-CoV-2 infections and COVID-19 deaths. In this section, we explore potential sources of this heterogeneity by examining the relationships between the estimated effects of school closures and key country-specific factors, including demographics and the non-pharmaceutical interventions in place during the study period.

First, we evaluated the correlation between the estimated effects of school closures on infections and deaths, and the following factors:

- proportion of individuals aged under 15 years old,
- proportion of individuals aged 70 years old and above,
- proportion of enrolled students in the population,
- total duration of school closure,
- average stringency of PHSMs other than school closures implemented during school closures.

Table A presents the correlation between each of these factors and the estimated effect of school closures on infections and deaths.

**Correlation**	**Intervention effect on SARS-CoV-2 infections**	**Intervention effect on COVID-19 deaths**
N weeks of schools closures	0.43	0.33
Average Oxford Stringency index during school closures	-0.18	-0.20
% of population under 15 years old (year 2020)	0.46	0.31
% of population enrolled in schools (year 2020)	0.44	0.34
% of population above 70 years old (year 2020)	-0.54	-0.39

Table A: Correlation between various factors and the estimated intervention effect on SARS-CoV-2 infections and COVID-19 deaths

Based on these univariate correlation coefficients, we observe that school closures may have more beneficial effects on infections and deaths in settings that exhibit one or more of the following characteristics:

1. a large proportion of individuals aged under 15,
2. a large proportion of enrolled students,
3. less stringent PHSMs (other than school closures) during school periods
4. a small proportion of individuals aged 70 and above

5. a longer total duration of school closures

It is important to note that calculating the correlation coefficients constitute a crude approach that was only used as a first step in our explorations. The following sections present how we calculated the variables associated with each of these factors and their detailed relationships with the estimated effect of school closures on infections and deaths, respectively.

B.1.1 Proportion of individuals aged under 15 years old

The proportion of individuals aged under 15 years old was calculated from the population age distributions reported by the United Nations' Population Division for year 2020.

Figure G presents how the estimated effects of school closures on infections and deaths varied with the proportion of individuals aged under 15.

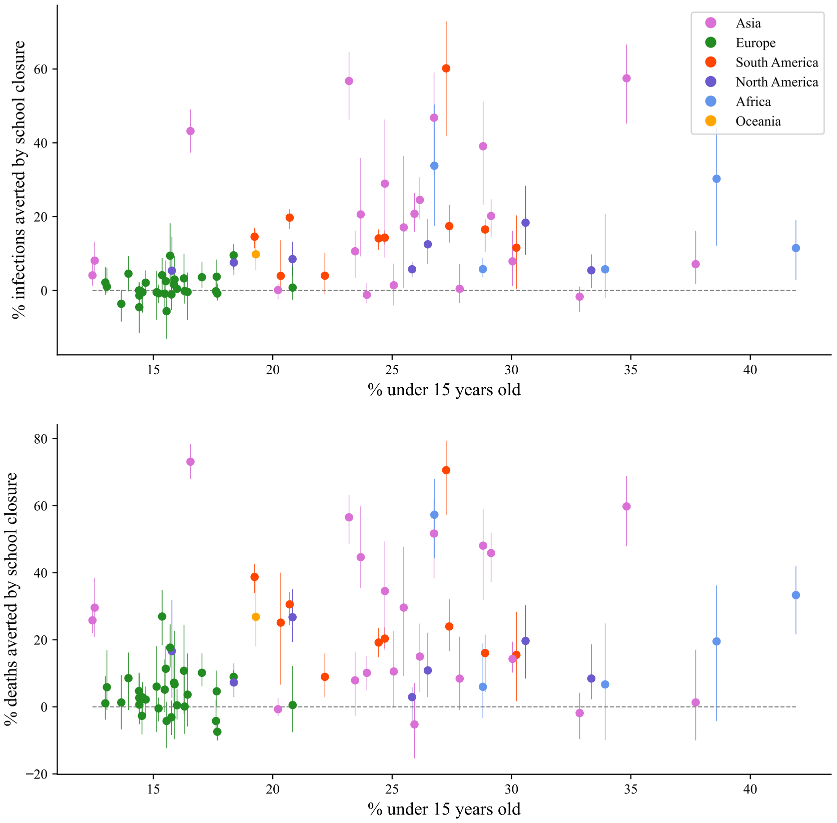

Fig G: Relationship between school closure impact on infections (top panel) and deaths (bottom panel), and proportion of individuals aged under 15 years old. Intervention effects are presented as median (dots) and ranges (bars), with colours indicating the continents to which the countries belong.

B.1.2 Proportion of individuals aged 70 years old and above

The proportion of individuals aged 70 years old and above was calculated from the population age distributions reported by the United Nations' Population Division for year 2020.

Figure H presents how the estimated effects of school closures on infections and deaths varied with the proportion of individuals aged 70 and above.

B.1.3 Proportion of enrolled students

For each country, the total number of enrolled students was obtained from the UNESCO database on school closures for year 2020. The proportion of enrolled students was then obtained by dividing the

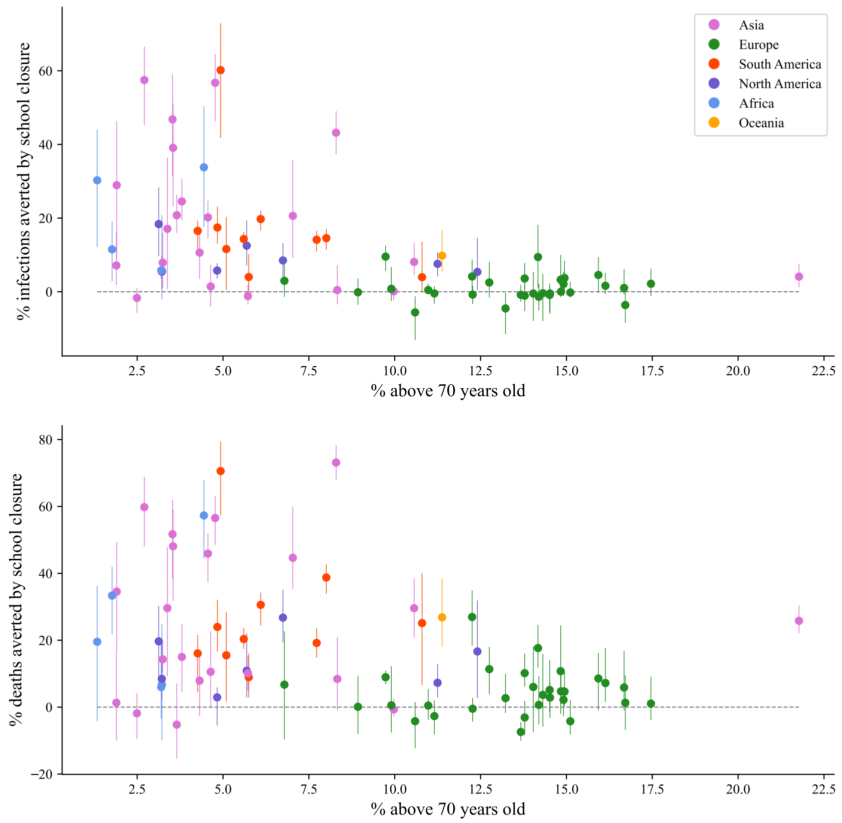

Fig H: Relationship between school closure impact on infections (top panel) and deaths (bottom panel), and proportion of individuals aged 70 years old and over. Intervention effects are presented as median (dots) and ranges (bars), with colours indicating the continents to which the countries belong.

number of enrolled students by the total population size reported by the United Nations' Population Division for year 2020.

Figure I presents how the estimated effects of school closures on infections and deaths varied with the proportion of enrolled students in the population.

B.1.4 Total duration of school closures

The total duration of school closures was calculated using the UNESCO school closure database. The total number of school closure weeks was calculated by adding the number of weeks fully closed, and the number of weeks partially closed multiplied by 0.3 (our midpoint estimate for assumed attendance fraction during partial closures).

Figure J presents how the estimated effects of school closures on infections and deaths varied with the total duration of school closures. Note that the bottom panel of Figure J presents the same data as Figure 2 in the main text.

B.1.5 Average PHSM stringency index

We used stringency index data from The Oxford Covid-19 Government Response Tracker (OxCGRT) to inform our calculations of the average stringency index. Namely, we used the same approach as that used by OxCGRT to compute their *StringencyIndex_Average* index, but we excluded the school closure component from the calculations. Thus, the resulting average stringency index reflects the total extent to which PHSMs other than school closures were implemented in each country. A higher value of the index indicates a higher PHSM stringency level.

Figure K presents how the estimated effects of school closures on infections and deaths varied with the average stringency index for PHSMs other than school closures.

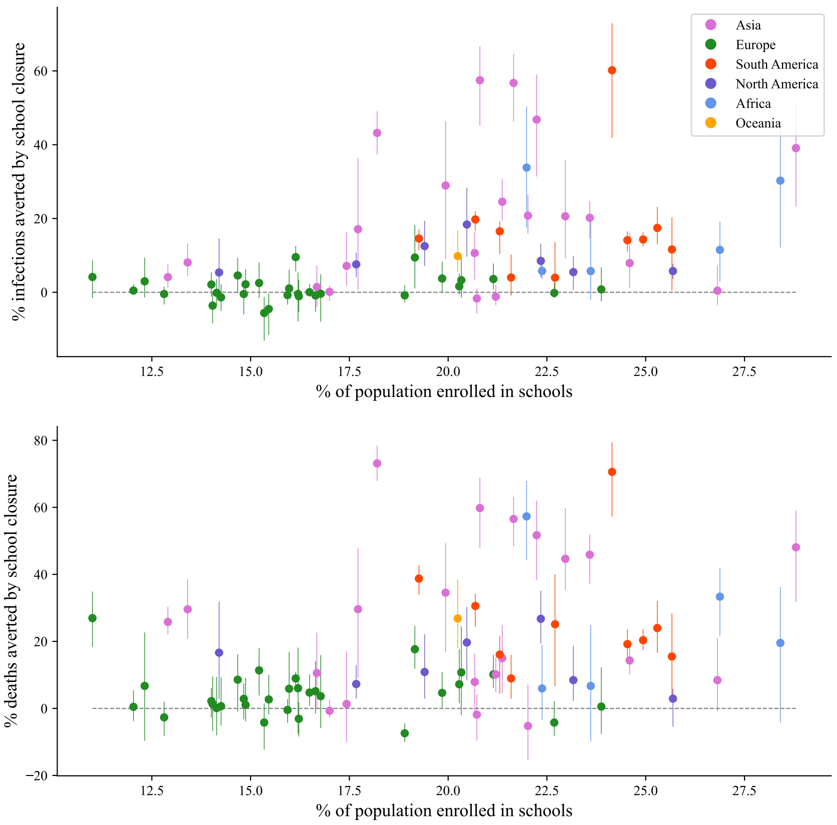

Fig I: Relationship between school closure impact on infections (top panel) and deaths (bottom panel), and proportion of students in the population. Intervention effects are presented as median (dots) and ranges (bars), with colours indicating the continents to which the countries belong.

B.1.6 Multivariate analysis

In order to account for potential confounding, we performed a multivariate analysis to further explore the heterogeneity observed in the estimated effects of school closures on infections and deaths.

First, we noted that some of the factors investigated in the previous sections were dependent. In particular, three factors were directly relevant to the age-structure of the population and therefore highly dependent: proportion under 15 years old, proportion 70 years and above, and proportion of students. We used a causal directed acyclic graph (cDAG) to better represent these dependencies in the context of our intervention effects estimations (Figure L).

The cDAG supports including three factors in a multilinear model: the population age structure, the total duration of school closures, and the stringency of PHSMs other than school closures. Among the factors related to population age structure, the correlation coefficients suggest that the proportion of individuals aged 70 and above may have the strongest relationship with the estimated effects of school closures. Consequently, we incorporated the following three variables into our multilinear regression analysis:

- proportion of individuals aged 70 years old and above,
- total duration of school closure,
- average stringency of PHSMs other than school closures implemented during school closures.

Figure M presents the results of the multilinear regression.

The proportion of elderly was found to be the only statistically significant predictor (p-value ≤ 0.05) of the effect of school closures on infections and deaths. This suggests that settings exhibiting a higher proportion of elderly individuals are less likely to benefit from school closures in terms of both number of infections and deaths. The other two factors were not found to be significantly associated with the outcome variables.

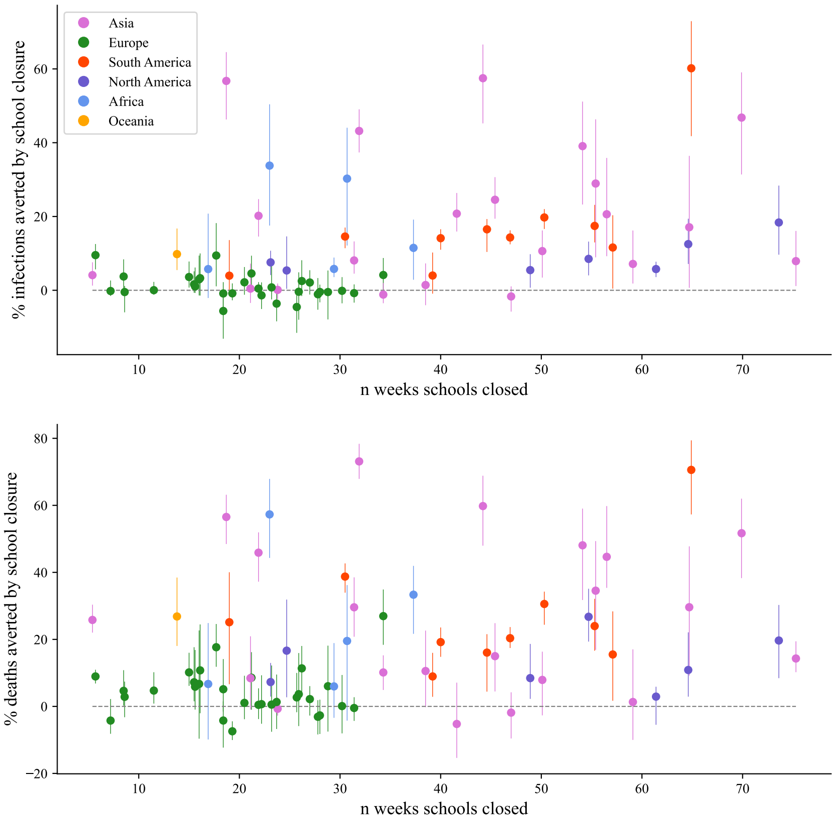

Fig J: Relationship between school closure impact on infections (top panel) and deaths (bottom panel), and total duration of school closures. Intervention effects are presented as median (dots) and ranges (bars), with colours indicating the continents to which the countries belong.

B.2 Sensitivity analyses

B.2.1 SA1: Increased household transmission during school closures

In this sensitivity analysis, we assumed that effective contact rates within households were increased during school closure periods. Specifically, we assumed that each individual had 20% more household contact potential when schools were fully closed.
The results of this sensitivity analysis are presented in Figure N and Figure O.

B.2.2 SA2: Google Mobility data not included

In this sensitivity analysis, we removed the contribution of Google Mobility data from our model. Under this configuration, we exclusively rely on the non-mechanistic component (i.e., random process) to capture mobility changes in locations other than households and schools.

The results of this sensitivity analysis are presented in Figure P and Figure Q.

B.2.3 SA3: Using alternative mixing matrices

We described our base-case approach to capturing heterogeneous mixing by age using contact matrices in Section A.1.2.5. Here we used an alternative source of age-specific contact matrices using Mistry et al. [30], which provides estimates for 25 of our included countries.

Figure R presents the results of this sensitivity analysis.

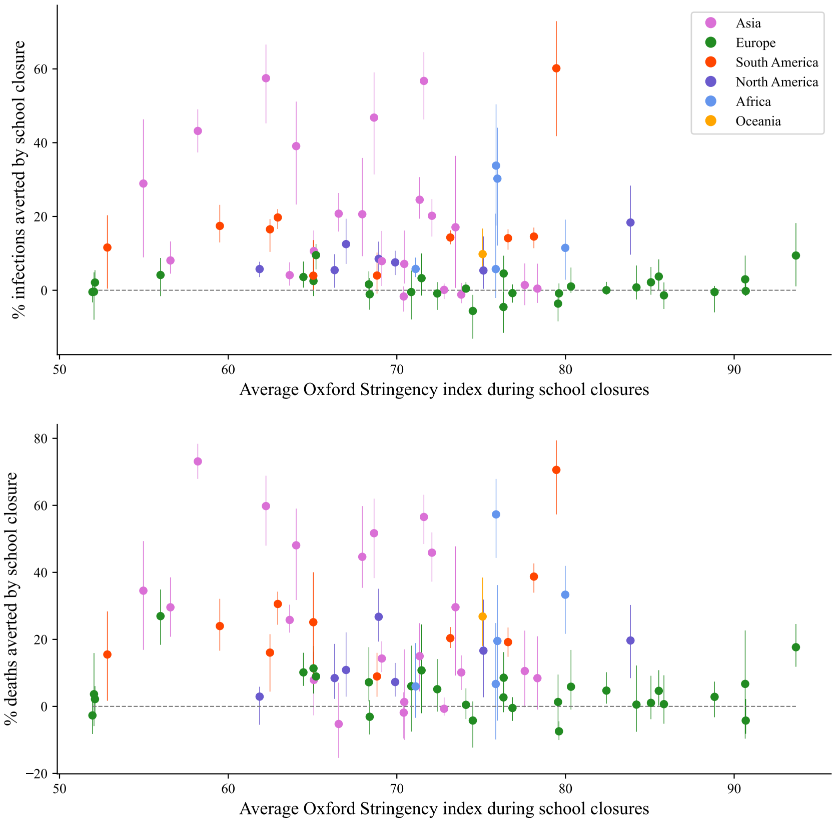

Fig K: Relationship between school closure impact on infections (top panel) and deaths (bottom panel), and average stringency of PHSMs other than school closures. Intervention effects are presented as median (dots) and ranges (bars), with colours indicating the continents to which the countries belong.

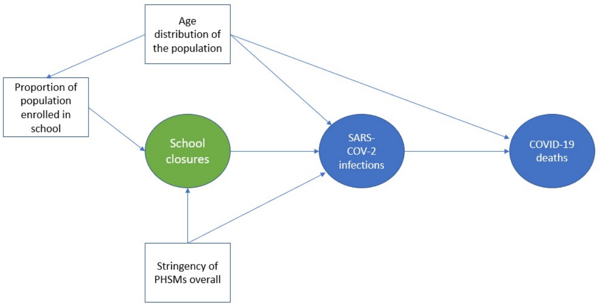

Fig L: Simple causal directed acyclic graph (cDAG) for the effect of school closures on SARS-Cov-2 infections and distal outcome COVID-19 deaths. This cDAG informed the construction of a simple linear model examining this effect. Green circle=main exposure of interest, Blue circles=outcomes of interest, Boxes=adjusted confounding covariates. PHSMs = public health and social measures.

B.2.4 Output comparisons between analyses

In this section, we present side-by-side comparisons of the main outputs between the four analyses (Base-case, SA1, SA2 and SA3). We present comparisons for the effect of school closures on infections (Figure S), deaths (Figure T) and peak hospital occupancy (Figure U).
Finally, Figure V provides overall comparisons across all countries analysed, considering the estimated median effects alone for the analyses covering all included countries (Base-case, SA1 and SA2).

	coef	std err	t	P>|t|	[0.025	0.975]
const	29.4147	15.063	1.953	0.055	-0.635	59.465
prop_elderly	-1.4392	0.420	-3.423	0.001	-2.278	-0.600
stringency	-0.1063	0.165	-0.642	0.523	-0.436	0.224
n_weeks_closed	0.0509	0.123	0.414	0.680	-0.194	0.296

(a)

	coef	std err	t	P>|t|	[0.025	0.975]
const	43.2774	20.638	2.097	0.040	2.105	84.450
prop_elderly	-1.2719	0.576	-2.208	0.031	-2.421	-0.123
stringency	-0.2375	0.227	-1.047	0.299	-0.690	0.215
n_weeks_closed	0.0435	0.169	0.258	0.797	-0.293	0.380

(b)

Fig M: Results of the multilinear regression exploring the relationship between different population factors and the effects of school closures on infections (panel a) and deaths (panel b). The factors are: the proportion of individuals aged 70 years old and above (*prop_elderly*), the average stringency of PHSMs other than school closures implemented during the school closures (*stringency*), and the total duration of school closures (*n_weeks_closed*).

B.2.5 Likelihood comparisons between analyses

In this section, we aimed to compare the ability of the different models to fit the data. For this, we present the values of the a-posteriori log-likelihood function (see Section A.3 for definition and Equation A.12) obtained under the different configurations in Figure W.

To better understand the differences observed in the a-posteriori log-likelihood values, we also presented the likelihood component that is relevant to the time-variant random-process in Figure X (see Section A.4 for definition).

B.2.6 Testing alternative model structures to capture incubation and active disease periods

In the base-case analysis we used four serial compartments ($k = 4$) to capture each of the incubation and active disease periods (see Section A.1.2.1). To test the sensitivity of our results to changes in model structure, we considered alternative assumptions for the number of serial compartments used to model these two periods ($k = 2$ and $k = 6$). Figure Y summarises the three configurations considered in terms of assumed time distributions for the incubation and active disease periods.

Figures Z, AA and AB present output comparisons for Morocco, Indonesia and the UK, respectively. We found no noticeable differences in the results obtained between the different approaches.

B.2.7 Exploring more stringent school closures during the Delta wave in Indonesia

Indonesia experienced its largest COVID-19 wave in the second half of 2021, following the emergence of the Delta variant earlier that year. During 2021, schools in the country were partially closed outside of regular academic breaks. In this additional analysis, we explore a counterfactual scenario in which schools would have been fully closed throughout 2021, to assess whether a more stringent closure policy might have resulted in a lower COVID-19 burden.

Figures AC and AD display the results of this analysis, comparing COVID-19 mortality rates and cumulative infection proportions under both the historical scenario and the counterfactual scenario with more stringent school closures.

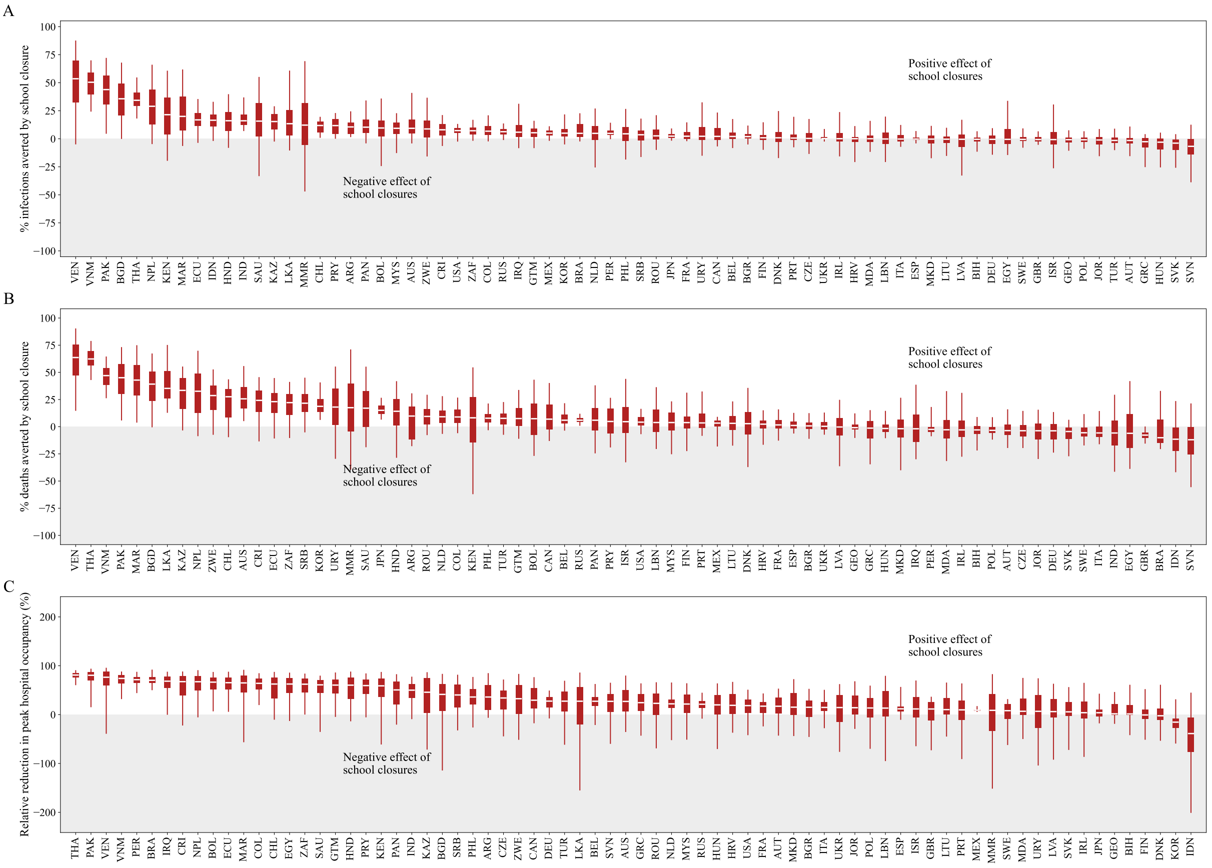

Fig N: Relative impact of school closures on COVID-19 infections, deaths and peak hospital occupancy (SA1: increased household transmission during closures)
Results are presented as relative percentage reductions in COVID-19 infections (A), COVID-19 deaths (B), and peak hospital occupancy (C). The counterfactual "schools open" scenario was used as reference. Estimates are presented as medians (horizontal lines), interquartile ranges (boxes), and 95% central credible intervals (vertical lines). Countries are listed in descending order from left to right, based on the estimated median effect for each disease indicator. See Table 1 to find the country associated with each ISO3 code.

B.3 Posterior samples exploration

In this section we present the parameter posterior distributions alongside prior distribution density plots, as well as pairwise plots of posterior samples for the three analyses highlighted in the main text (Morocco, Indonesia, and the United Kingdom). These visualisations provide a detailed overview of the parameter estimates and enable a comparison between prior beliefs and posterior results for each country.

The combined prior and posterior distribution plots allow for a direct comparison of the parameter estimates with the assumed priors, revealing how the data have influenced the posterior beliefs in each analysis (Figures AE–AG). The pairwise plots of posterior samples display the interactions between pairs of parameters, highlighting potential correlations or dependencies that may exist within the parameters across each regional dataset.

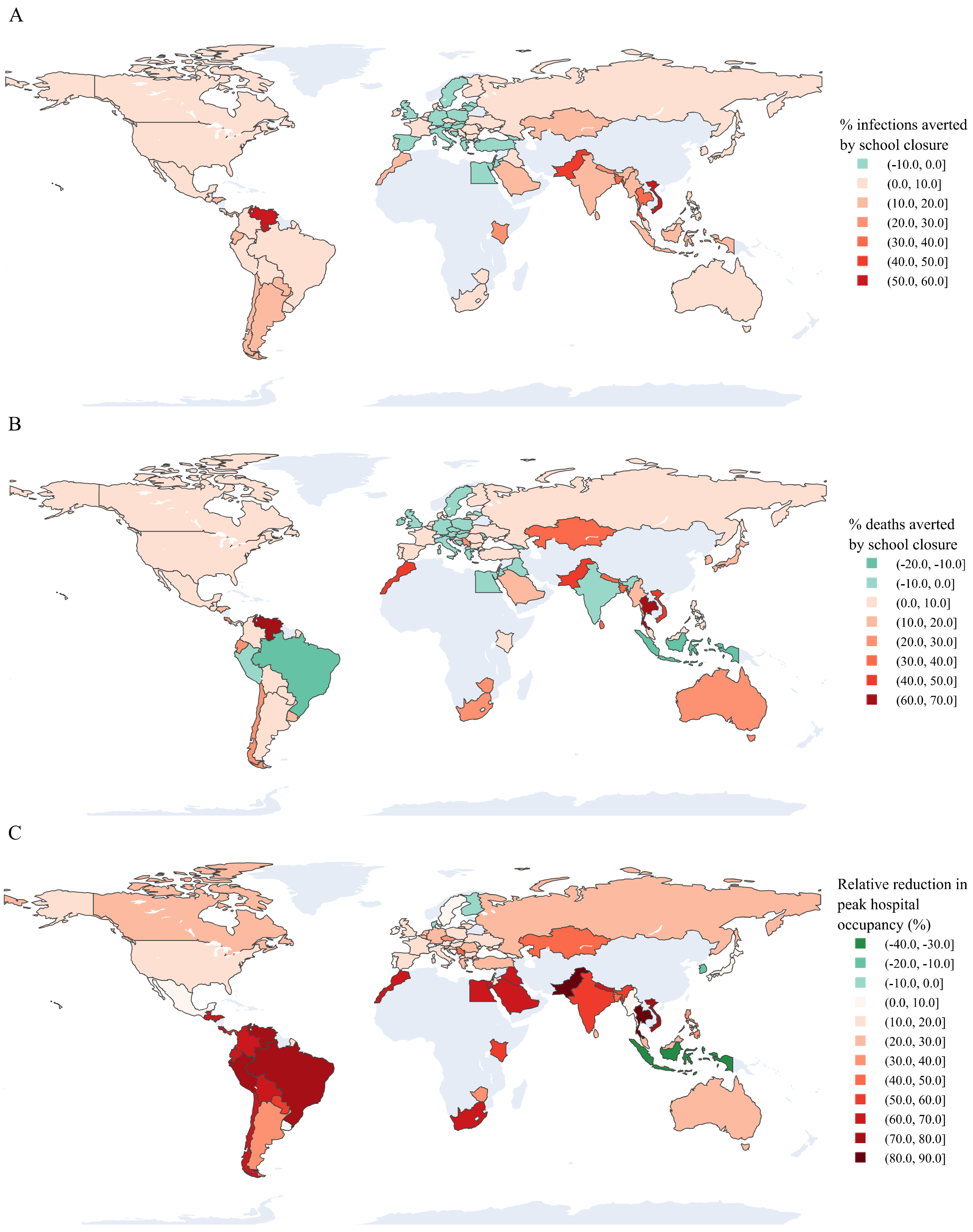

Fig O: Geographic representation of the effects of school closures on COVID-19 (SA1: increased household transmission during closures)
Results presented as median relative percentage reductions in SARS-CoV-2 infections (A), COVID-19 deaths (B), and peak hospital occupancy (C), due to school closures. The counterfactual "schools open" scenario was used as reference. Countries in light grey were not included in the analysis. Negative percentages indicate configurations where school closures are estimated to have had a negative impact on the considered indicator. The maps were generated with *plotly* (v5.14.1) using embedded geometric data derived from the Natural Earth dataset.

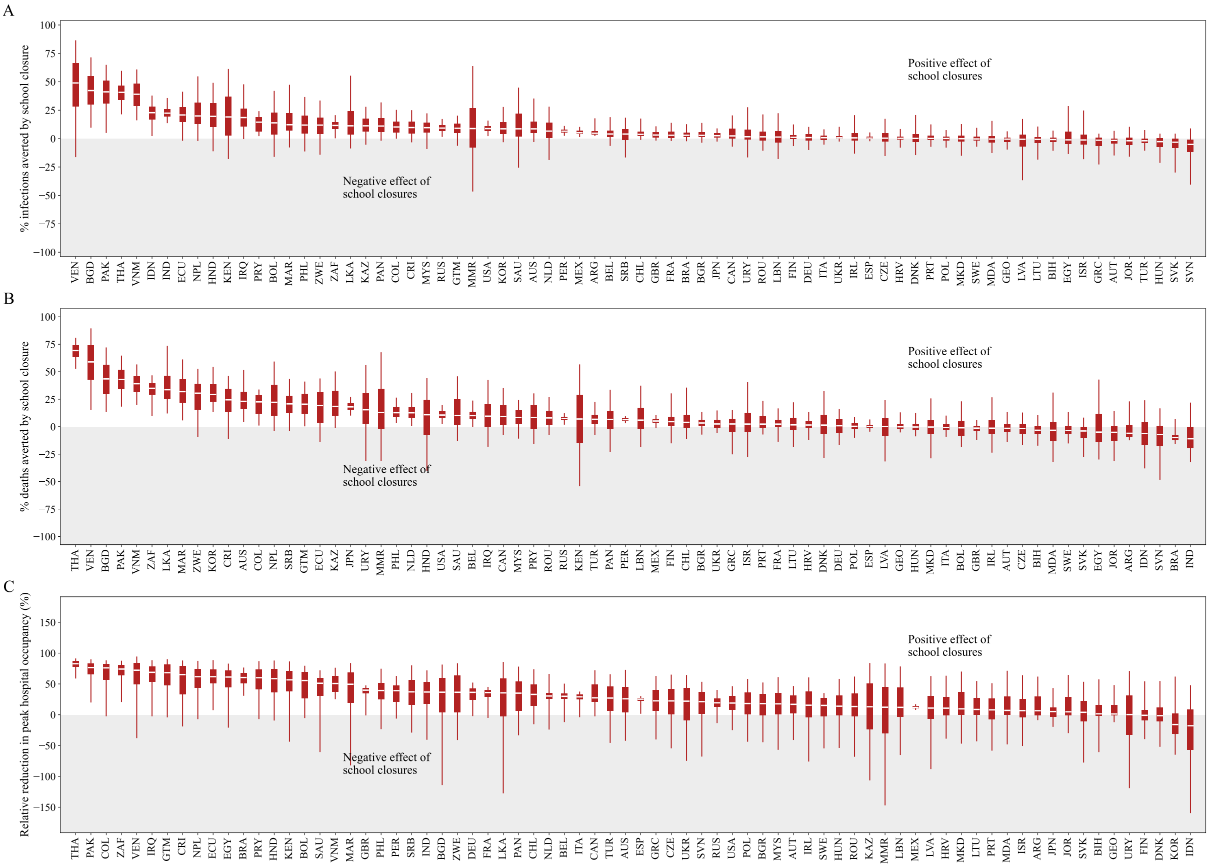

Fig P: Relative impact of school closures on COVID-19 infections, deaths and peak hospital occupancy (SA2: no Google Mobility data)

Results are presented as relative percentage reductions in COVID-19 infections (A), COVID-19 deaths (B), and peak hospital occupancy (C). The counterfactual "schools open" scenario was used as reference. Estimates are presented as medians (horizontal lines), interquartile ranges (boxes), and 95% central credible intervals (vertical lines). Countries are listed in descending order from left to right, based on the estimated median effect for each disease indicator. See Table 1 to find the country associated with each ISO3 code.

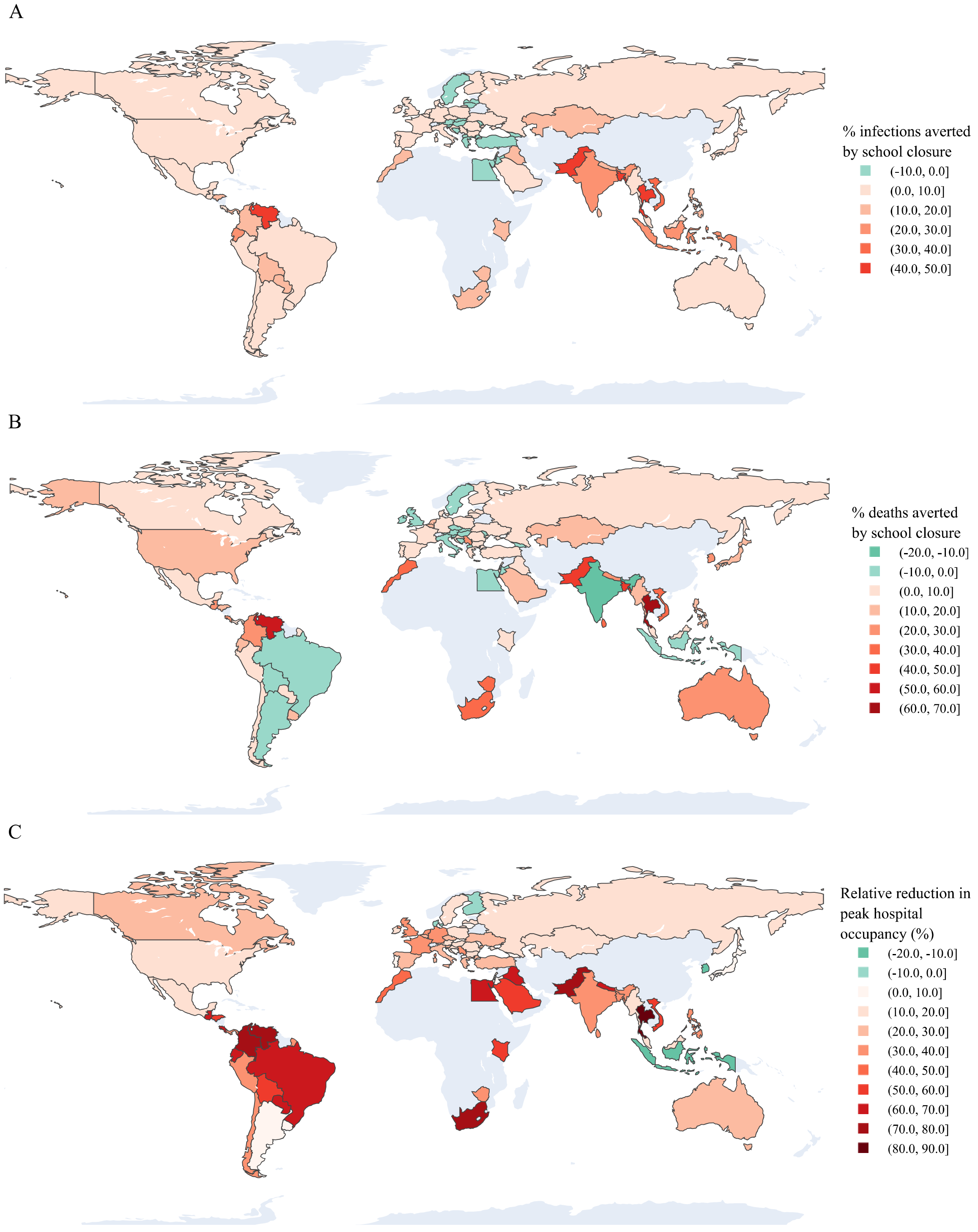

Fig Q: Geographic representation of the effects of school closures on COVID-19 (SA2: no Google Mobility data)

Results presented as median relative percentage reductions in SARS-CoV-2 infections (A), COVID-19 deaths (B), and peak hospital occupancy (C), due to school closures. The counterfactual "schools open" scenario was used as reference. Countries in light grey were not included in the analysis. Negative percentages indicate configurations where school closures are estimated to have had a negative impact on the considered indicator. The maps were generated with *plotly* (v5.14.1) using embedded geometric data derived from the Natural Earth dataset.

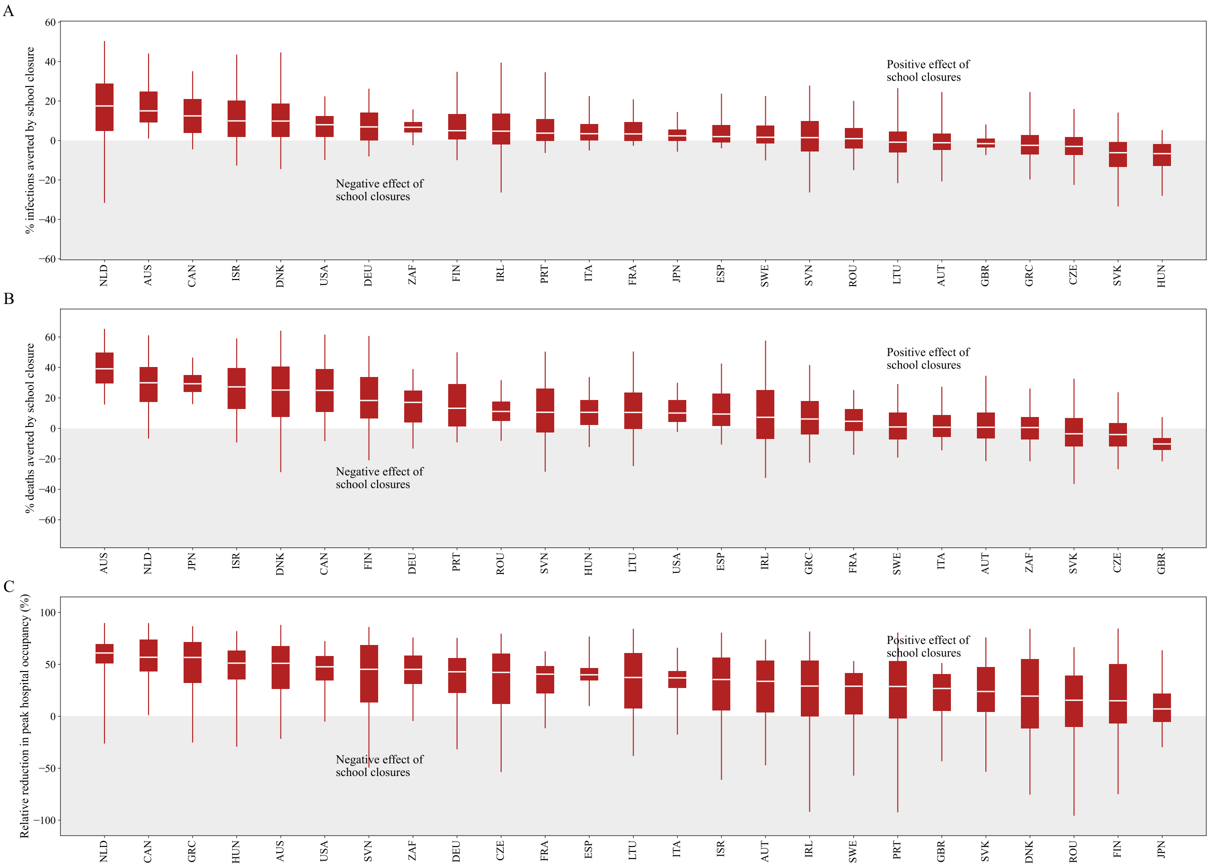

Fig R: Relative impact of school closures on COVID-19 infections, deaths and peak hospital occupancy (SA3: using alternative contact matrices)

Results are presented as relative percentage reductions in COVID-19 infections (A), COVID-19 deaths (B), and peak hospital occupancy (C). The counterfactual "schools open" scenario was used as reference. Estimates are presented as medians (horizontal lines), interquartile ranges (boxes), and 95% central credible intervals (vertical lines). Countries are listed in descending order from left to right, based on the estimated median effect for each disease indicator. See Table 1 to find the country associated with each ISO3 code.

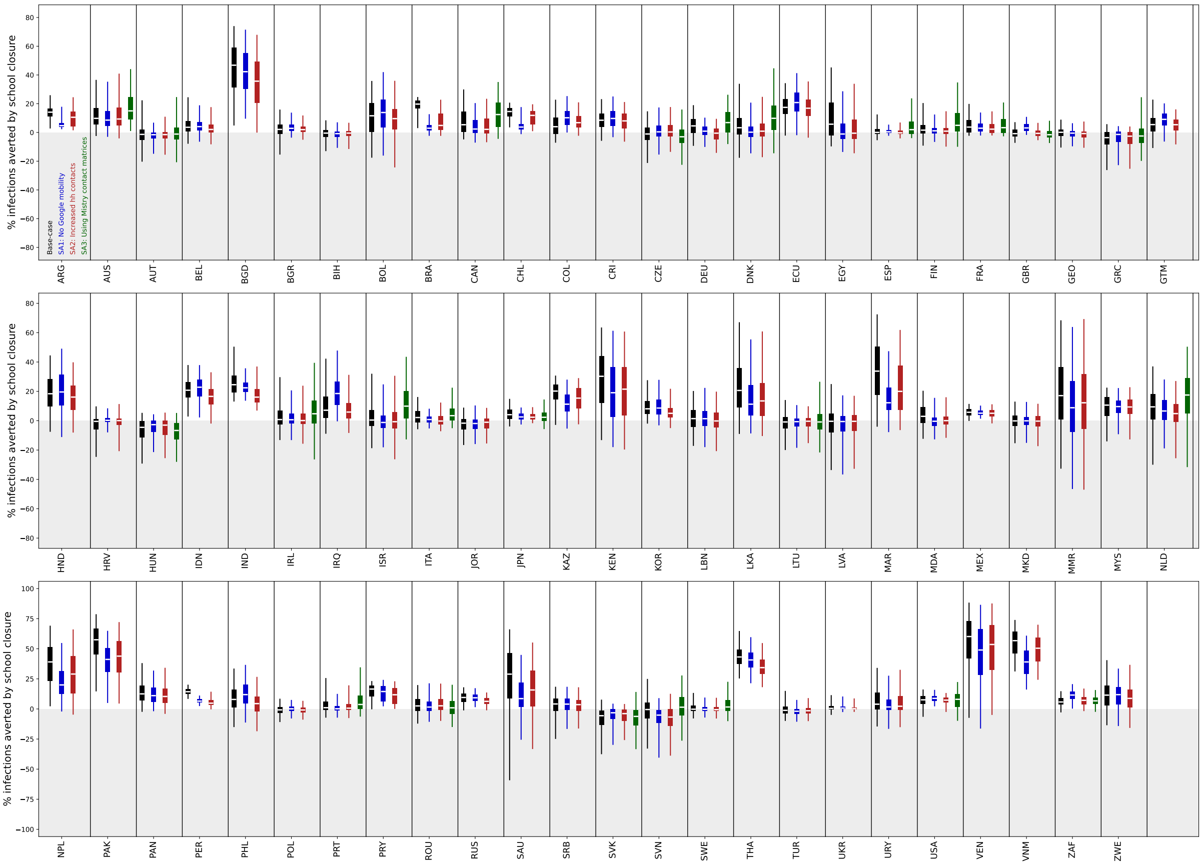

Fig S: Comparison of school closure effects on SARS-CoV-2 infections

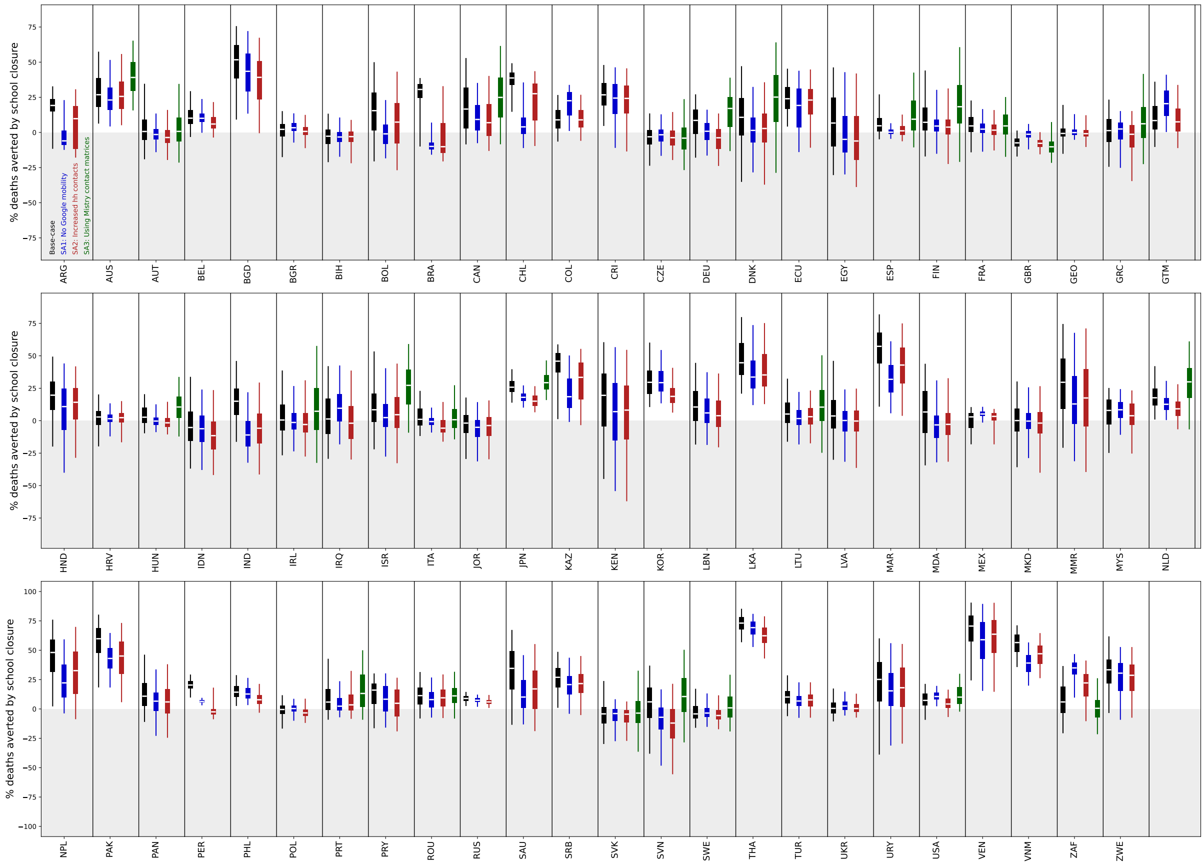

Fig T: Comparison of school closure effects on COVID-19 deaths

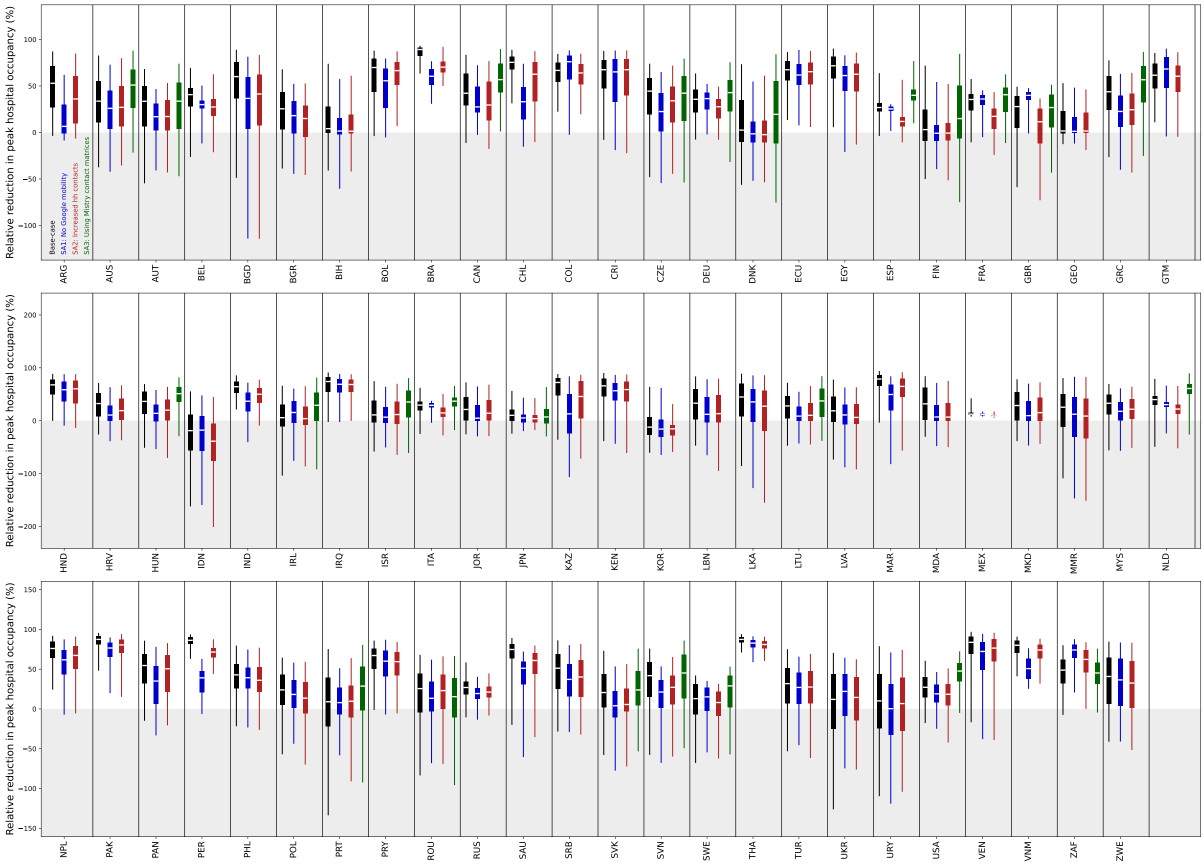

Fig U: Comparison of school closure effects on peak hospital occupancy

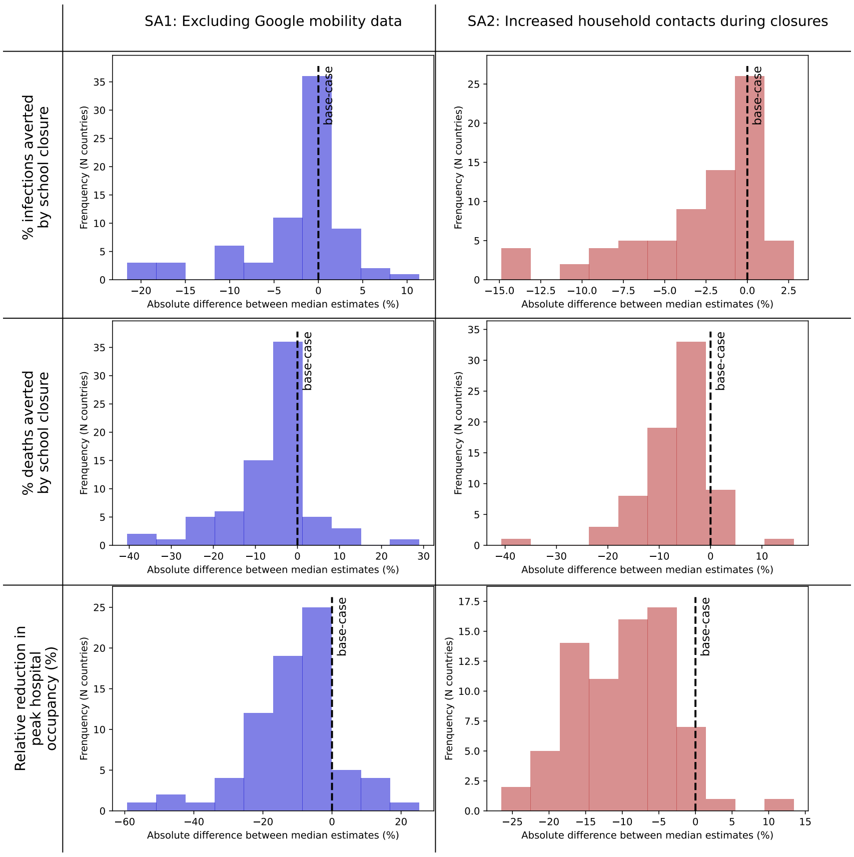

Fig V: Relative difference between median estimated effects of school closures between the different analyses

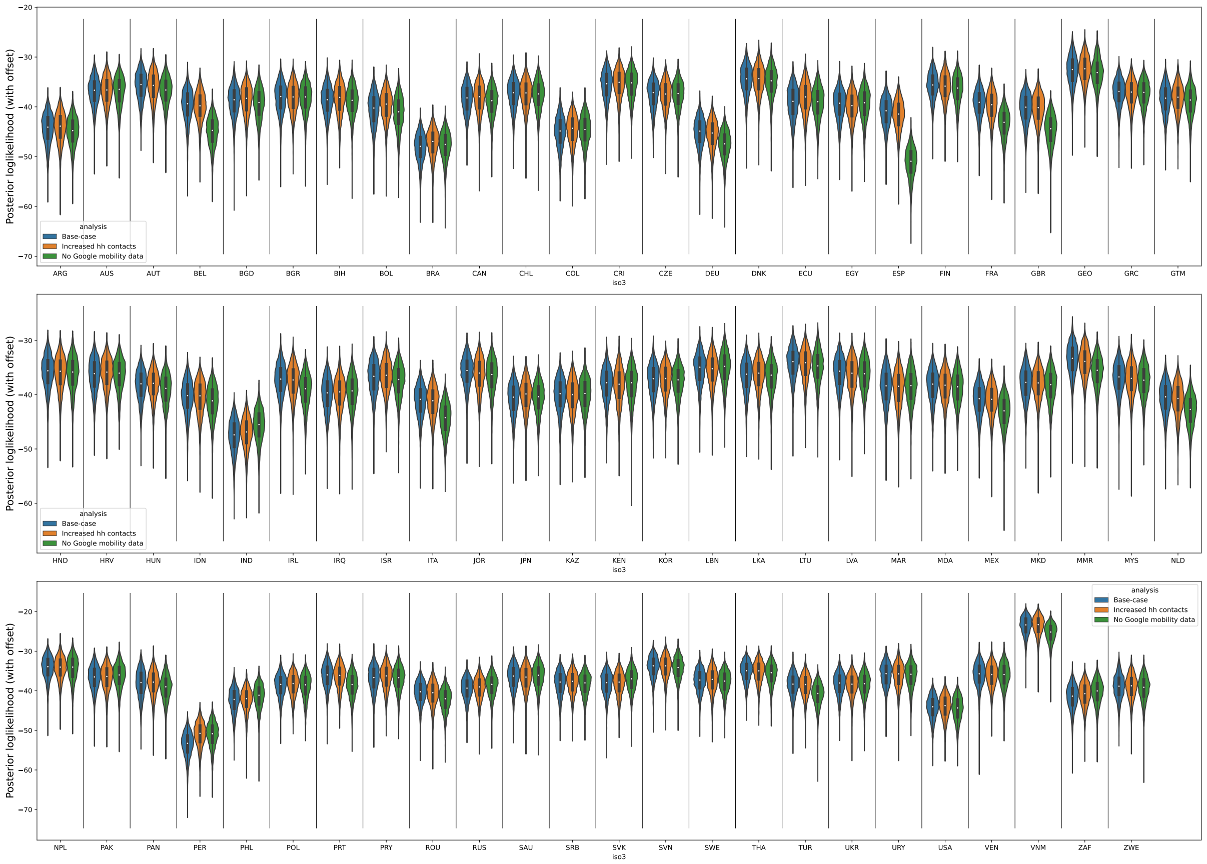

Fig W: Comparison of a-posteriori log-likelihood values between analyses
The a-posteriori log-likelihood combines the model likelihood, the parameter priors and the random process likelihood. Higher values indicate more realistic model fits. Note that the quantity presented in Equation A.12 is not exactly the a-posteriori likelihood but a quantity that is proportional to the latter. This is why the log-likelihood quantity presented here includes an offset.

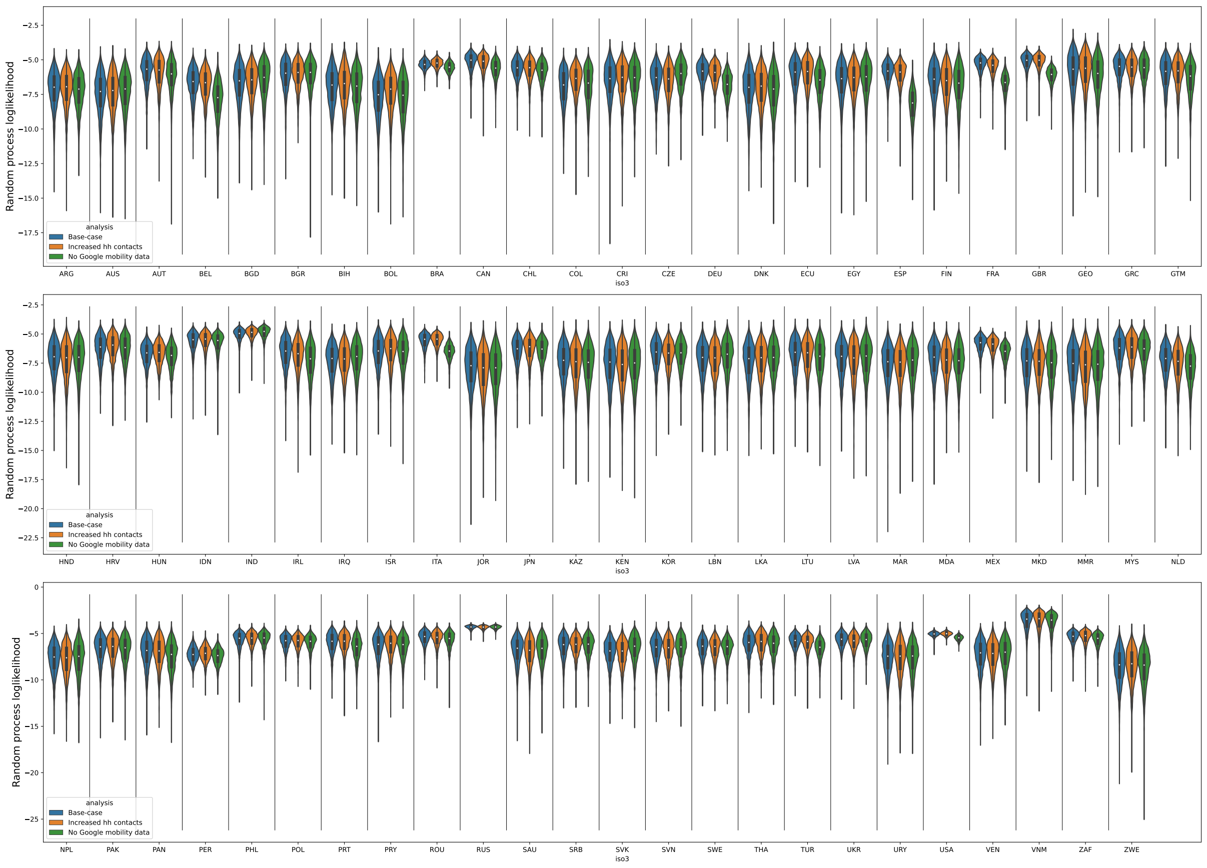

Fig X: Comparison of likelihood-components relevant to the time-variant random process Higher values indicate less overall variability in the random-process.

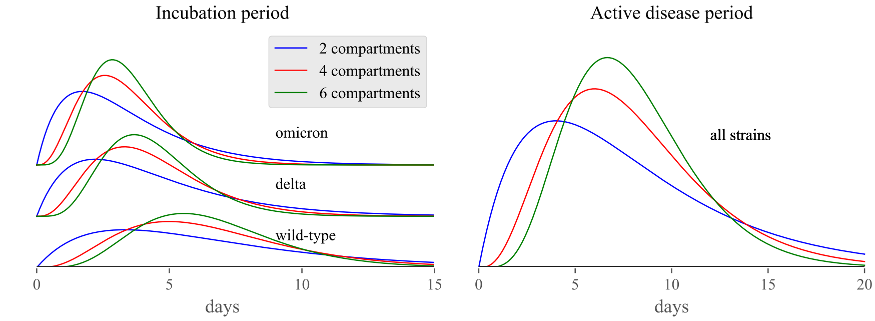

Fig Y: Alternative distributions of the incubation and active disease periods.

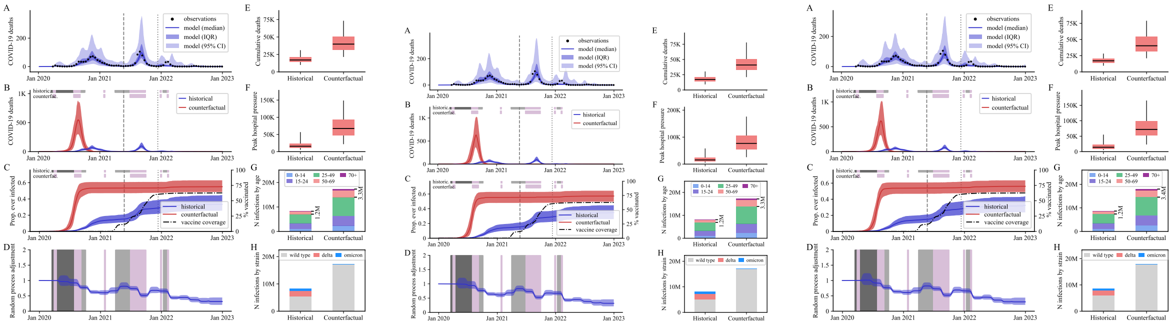

(a) 2 incubations + 2 active disease compartments (b) 4 incubations + 4 active disease compartments (base-case) (c) 6 incubations + 6 active disease compartments

Fig Z: Comparison of Morocco model outputs for different numbers of incubation and active disease compartments.

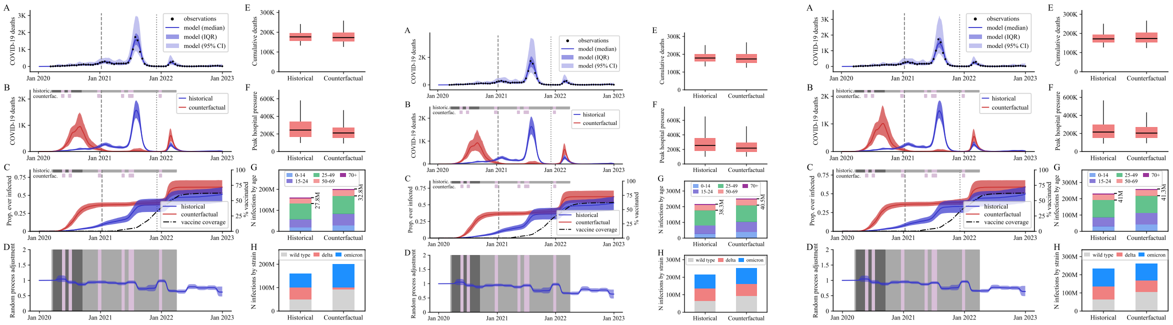

(a) 2 incubations + 2 active disease compartments (b) 4 incubations + 4 active disease compartments (base-case) (c) 6 incubations + 6 active disease compartments

Fig AA: Comparison of Indonesia model outputs for different numbers of incubation and active disease compartments.

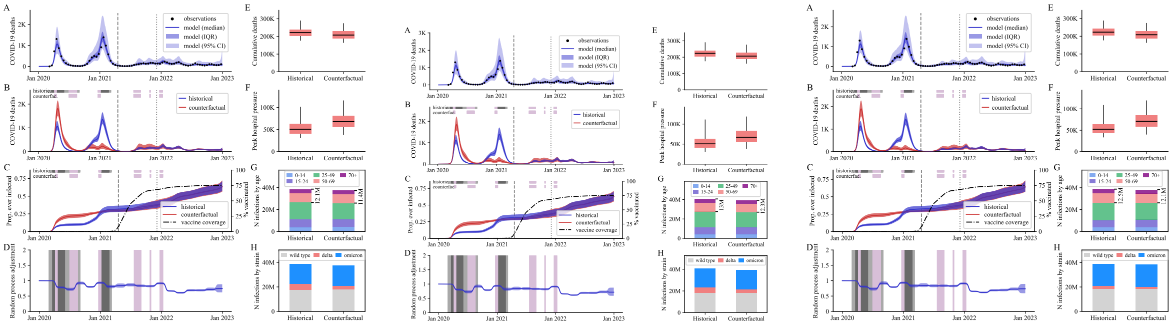

(a) 2 incubations + 2 active disease compartments (b) 4 incubations + 4 active disease compartments (base-case) (c) 6 incubations + 6 active disease compartments

Fig AB: Comparison of UK model outputs for different numbers of incubation and active disease compartments.

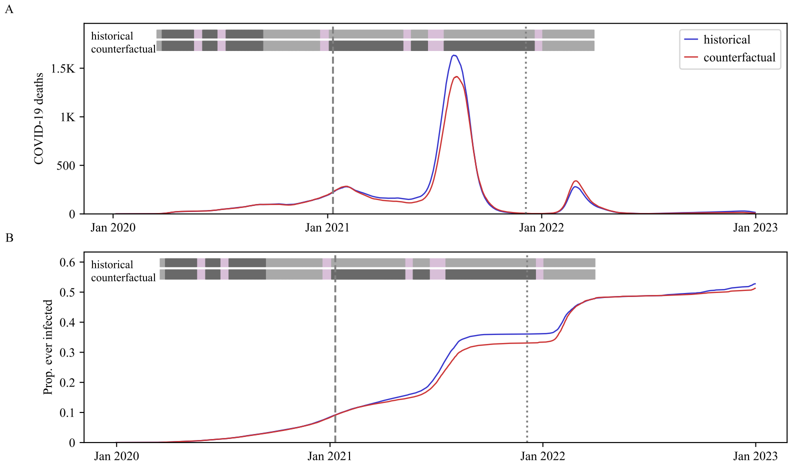

Fig AC: Median estimated COVID-19 mortality (panel A) and cumulative infection proportion (panel B) in Indonesia, under both the historical scenario and the counterfactual scenario with more stringent school closures. School closure statuses are illustrated with the horizontal coloured bands (dark grey: fully closed for COVID-19, light grey: partially closed due to COVID-19, purple: academic break)

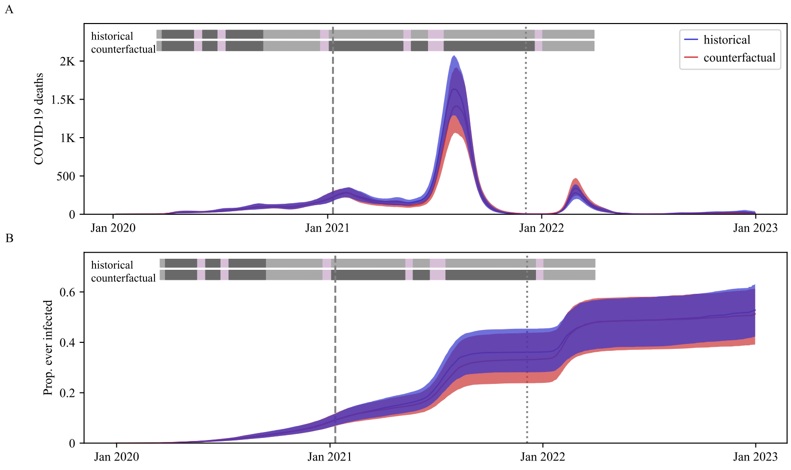

Fig AD: Estimated COVID-19 mortality (panel A) and cumulative infection proportion (panel B) in Indonesia, under both the historical scenario and the counterfactual scenario with more stringent school closures, including uncertainty. Shaded areas represent interquartile ranges and solid lines represent median estimates. School closure statuses are illustrated with the horizontal coloured bands (dark grey: fully closed for COVID-19, light grey: partially closed due to COVID-19, purple: academic break)

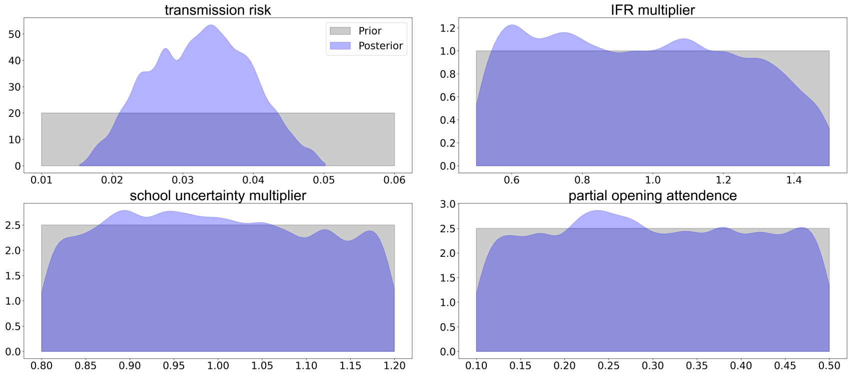

(a) Prior and posterior distributions of main epidemiological parameters

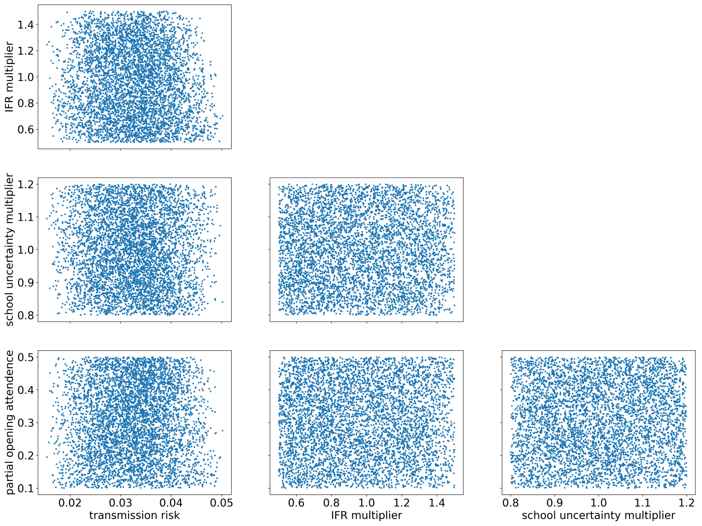

(b) Pairwise comparison

Fig AE: Parameter posterior distributions and pairwise comparison for Morocco.

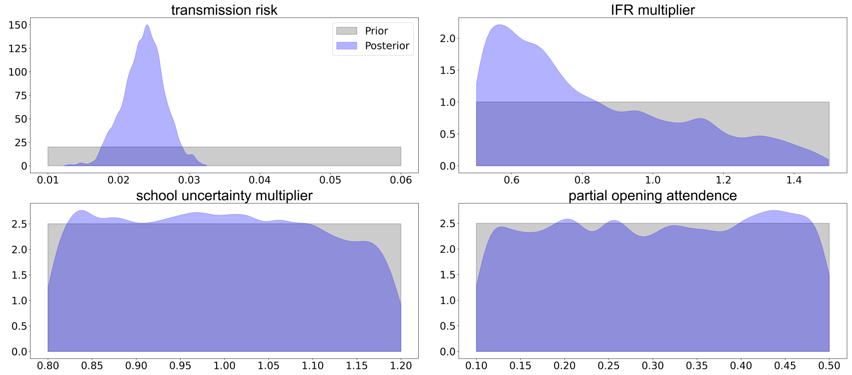

(a) Prior and posterior distributions of main epidemiological parameters

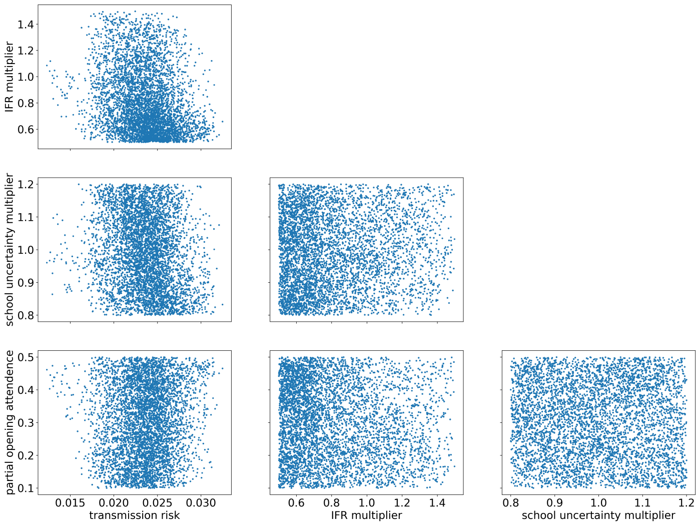

(b) Pairwise comparison

Fig AF: Parameter posterior distributions and pairwise comparison for Indonesia.

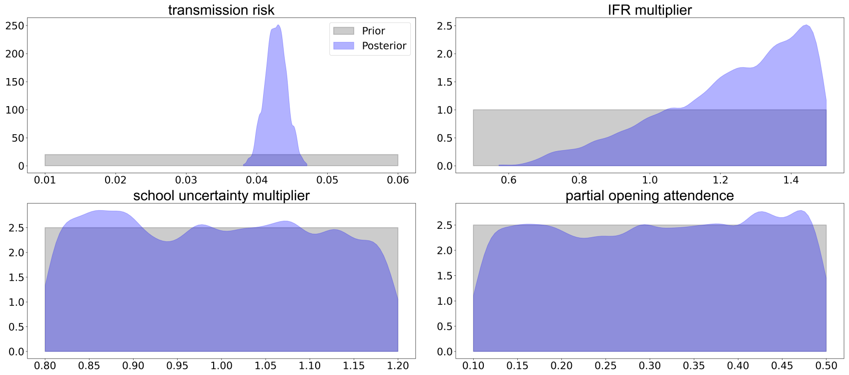

(a) Prior and posterior distributions of main epidemiological parameters

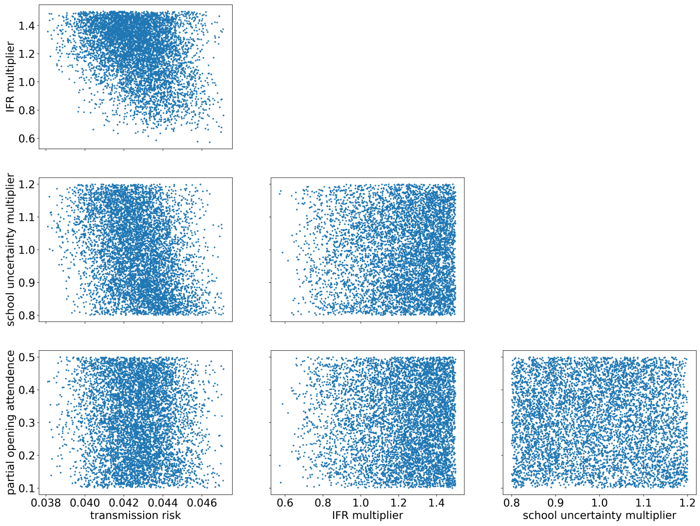

(b) Pairwise comparison

Fig AG: Parameter posterior distributions and pairwise comparison for the UK.

B.4 Detailed country-specific results

The next pages include detailed country-specific results for the 74 countries included in our analysis, under the base-case configuration. Detailed country-specific results associated with Sensitivity Analyses SA1, SA2 and SA3 are **available online**.

You can access any country's outputs under the base-case configuration by clicking on the relevant country's name below:

Argentina	Australia	Austria	Belgium
Bangladesh	Bulgaria	Bosnia and Herzegovina	
Bolivia	Brazil	Canada	Chile
Colombia	Costa Rica	Czechia	Germany
Denmark	Ecuador	Egypt	Spain
Finland	France	United Kingdom	Georgia
Greece	Guatemala	Honduras	Croatia
Hungary	Indonesia	India	Ireland
Iraq	Israel	Italy	Jordan
Japan	Kazakhstan	Kenya	Korea, Republic of
Lebanon	Sri Lanka	Lithuania	Latvia
Morocco	Moldova, Republic of	Mexico	North Macedonia
Myanmar	Malaysia	Netherlands	Nepal
Pakistan	Panama	Peru	Philippines
Poland	Portugal	Paraguay	Romania
Russian Federation	Saudi Arabia	Serbia	Slovakia
Slovenia	Sweden	Thailand	Turkey
Ukraine	Uruguay	United States	Venezuela
Viet Nam	South Africa	Zimbabwe	

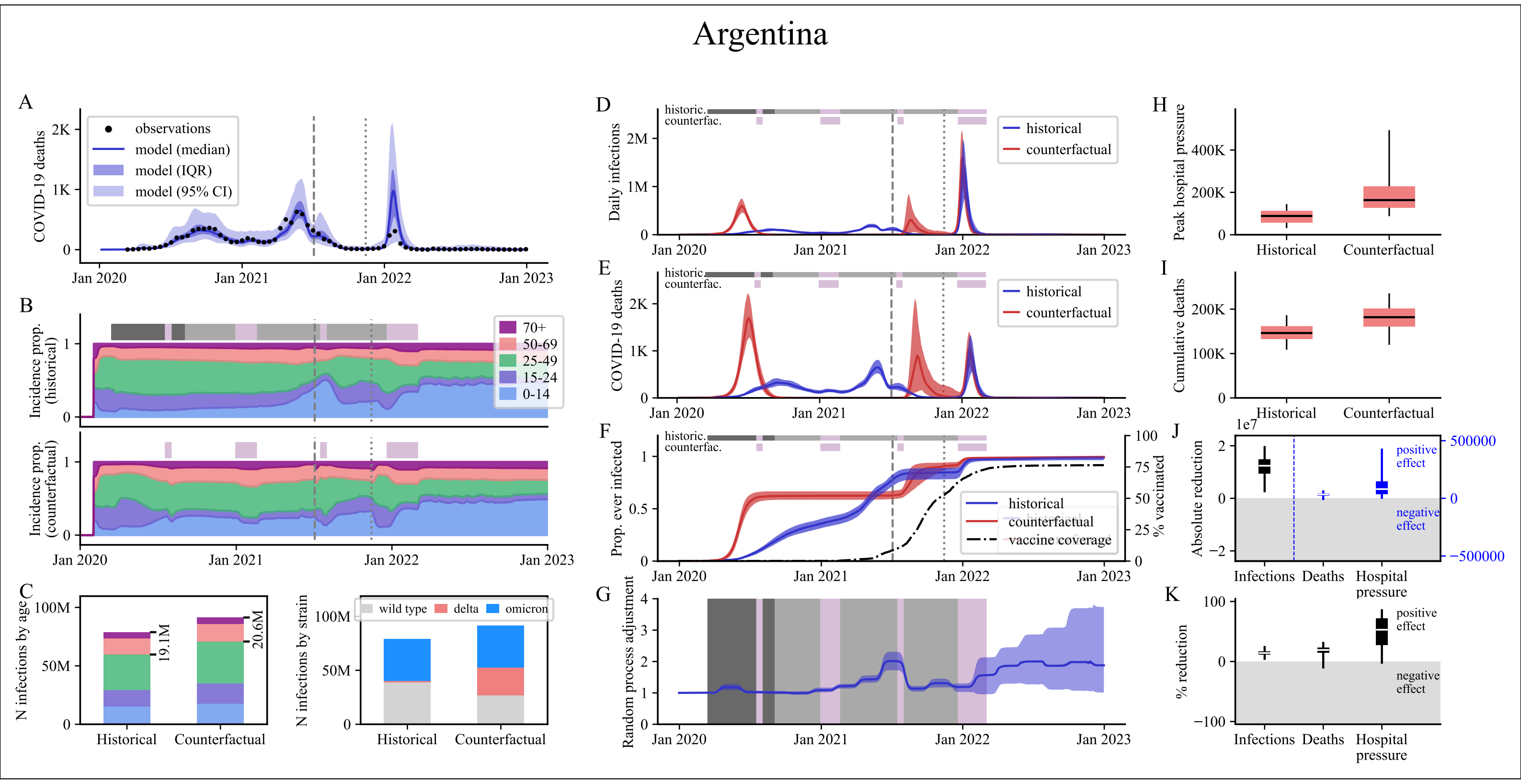

Argentina
A
COVID-19 deaths
observations
model (median)
model (IQR)
model (95% CI)
B
Incidence prop. (historical)
Incidence prop. (counterfactual)
70+
50-69
25-49
15-24
0-14
C
N infections by age
19.1M
20.6M
N infections by strain
wild type
delta
omicron
Historical
Counterfactual
D
Daily infections
historic.
counterfac.
historical
counterfactual
E
COVID-19 deaths
F
Prop. ever infected
% vaccinated
vaccine coverage
G
Random process adjustment
H
Peak hospital pressure
I
Cumulative deaths
J
Absolute reduction
1e7
positive effect
negative effect
Infections
Deaths
Hospital pressure
K
% reduction

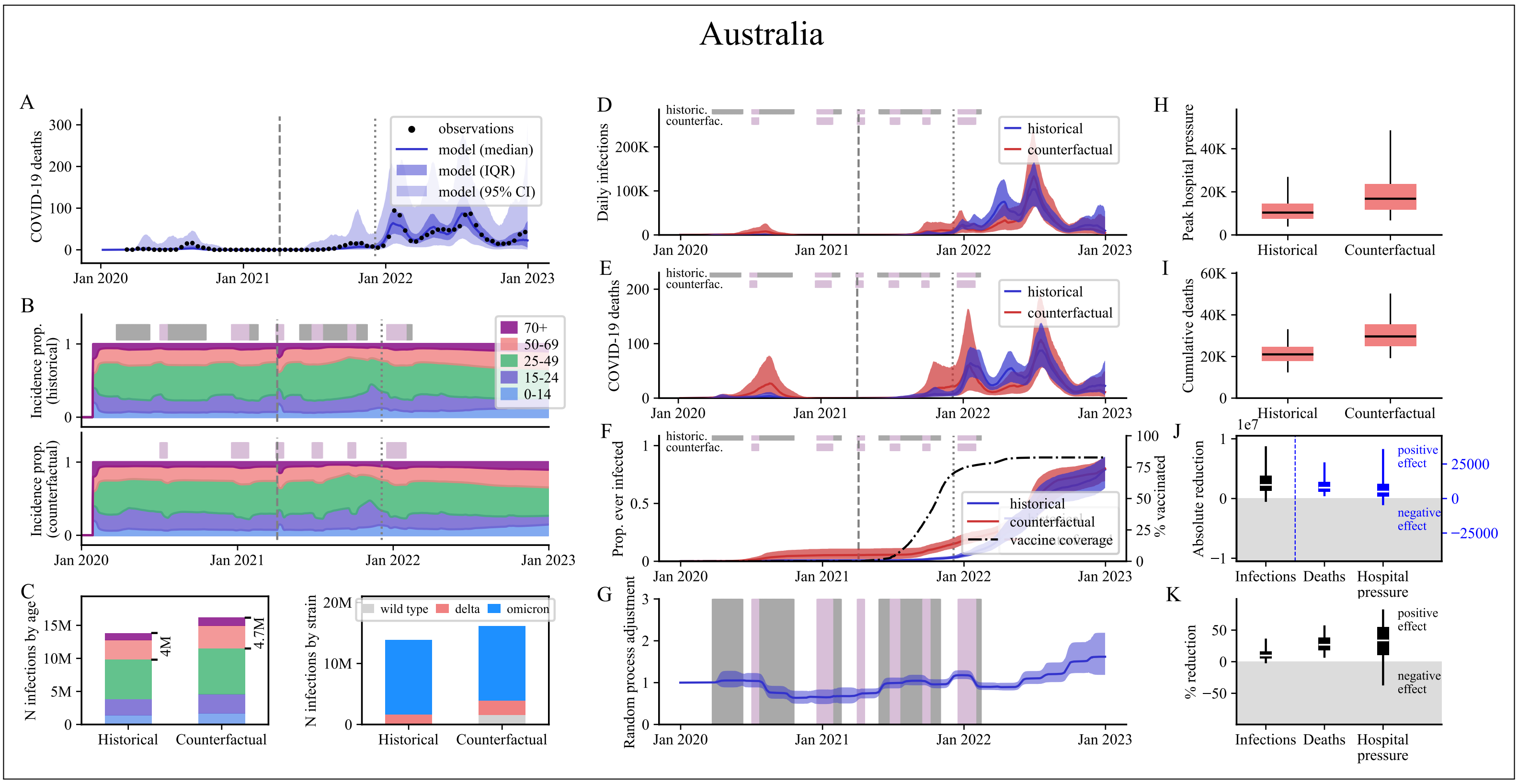

Australia
A
COVID-19 deaths
observations
model (median)
model (IQR)
model (95% CI)
B
Incidence prop. (historical)
Incidence prop. (counterfactual)
70+
50-69
25-49
15-24
0-14
C
N infections by age
4M
4.7M
N infections by strain
wild type
delta
omicron
Historical
Counterfactual
D
Daily infections
historic.
counterfac.
historical
counterfactual
E
COVID-19 deaths
F
Prop. ever infected
% vaccinated
vaccine coverage
G
Random process adjustment
H
Peak hospital pressure
I
Cumulative deaths
J
Absolute reduction
1e7
positive effect
negative effect
Infections
Deaths
Hospital pressure
K
% reduction

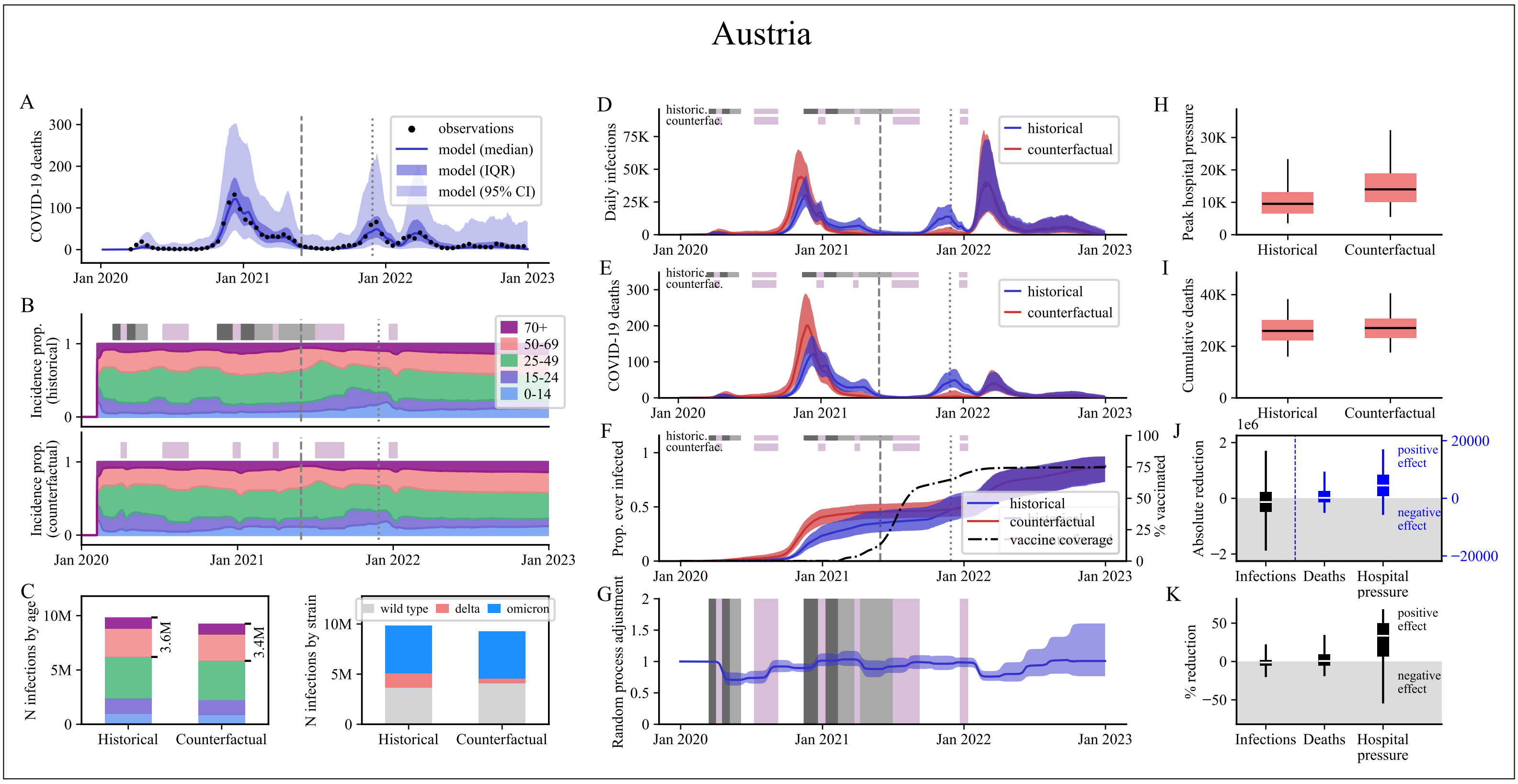
Austria
A
COVID-19 deaths
observations
model (median)
model (IQR)
model (95% CI)
B
Incidence prop. (historical)
Incidence prop. (counterfactual)
70+
50-69
25-49
15-24
0-14
C
N infections by age
3.6M
3.4M
Historical
Counterfactual
N infections by strain
wild type
delta
omicron
D
Daily infections
historic.
counterfac.
historical
counterfactual
E
COVID-19 deaths
F
Prop. ever infected
% vaccinated
vaccine coverage
G
Random process adjustment
H
Peak hospital pressure
I
Cumulative deaths
J
Absolute reduction
1e6
positive effect
negative effect
Infections
Deaths
Hospital pressure
K
% reduction
Jan 2020
Jan 2021
Jan 2022
Jan 2023

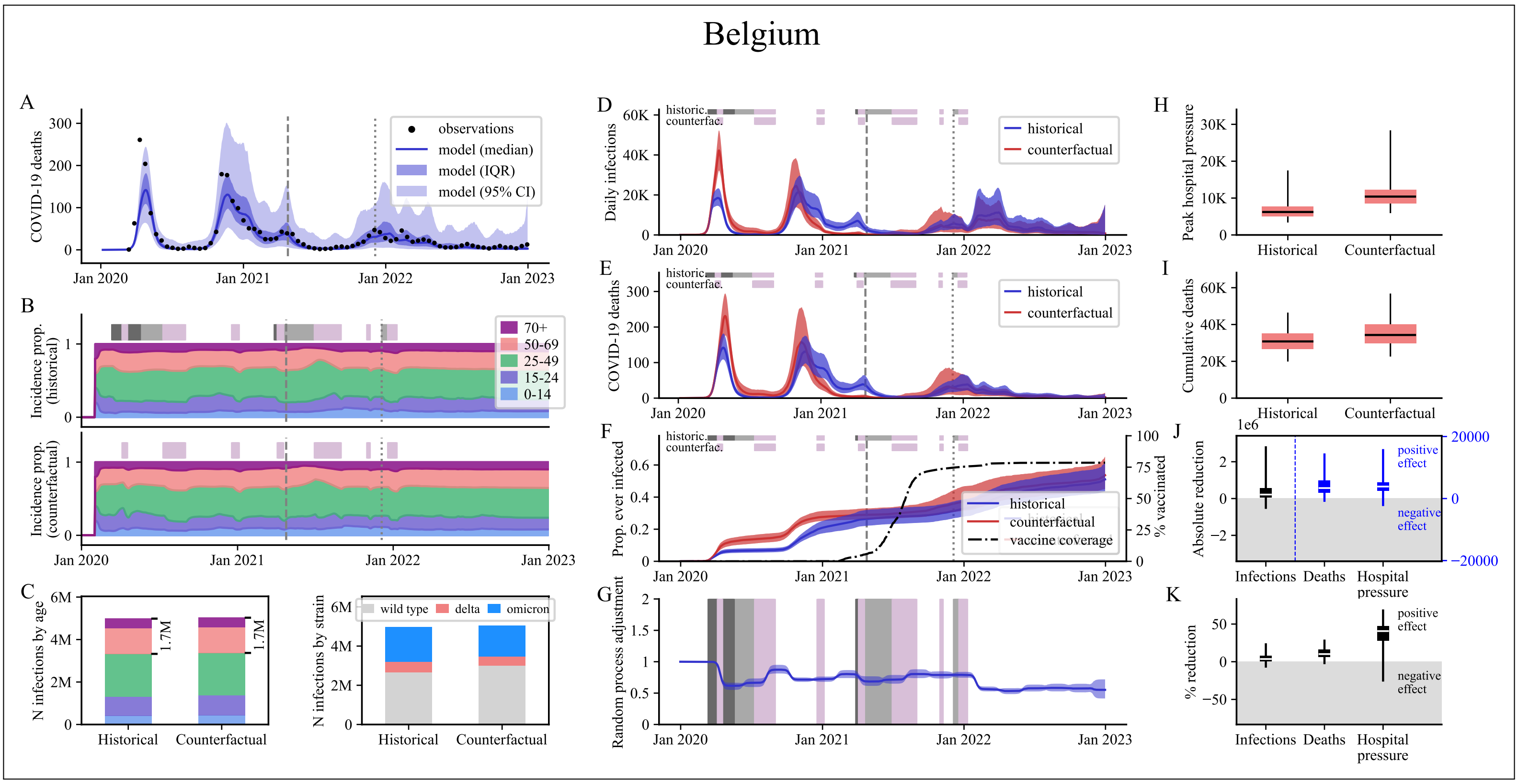
Belgium
A
COVID-19 deaths
observations
model (median)
model (IQR)
model (95% CI)
B
Incidence prop. (historical)
Incidence prop. (counterfactual)
70+
50-69
25-49
15-24
0-14
C
N infections by age
1.7M
1.7M
Historical
Counterfactual
N infections by strain
wild type
delta
omicron
D
Daily infections
historic.
counterfac.
historical
counterfactual
E
COVID-19 deaths
F
Prop. ever infected
% vaccinated
vaccine coverage
G
Random process adjustment
H
Peak hospital pressure
I
Cumulative deaths
J
Absolute reduction
1e6
positive effect
negative effect
Infections
Deaths
Hospital pressure
K
% reduction
Jan 2020
Jan 2021
Jan 2022
Jan 2023

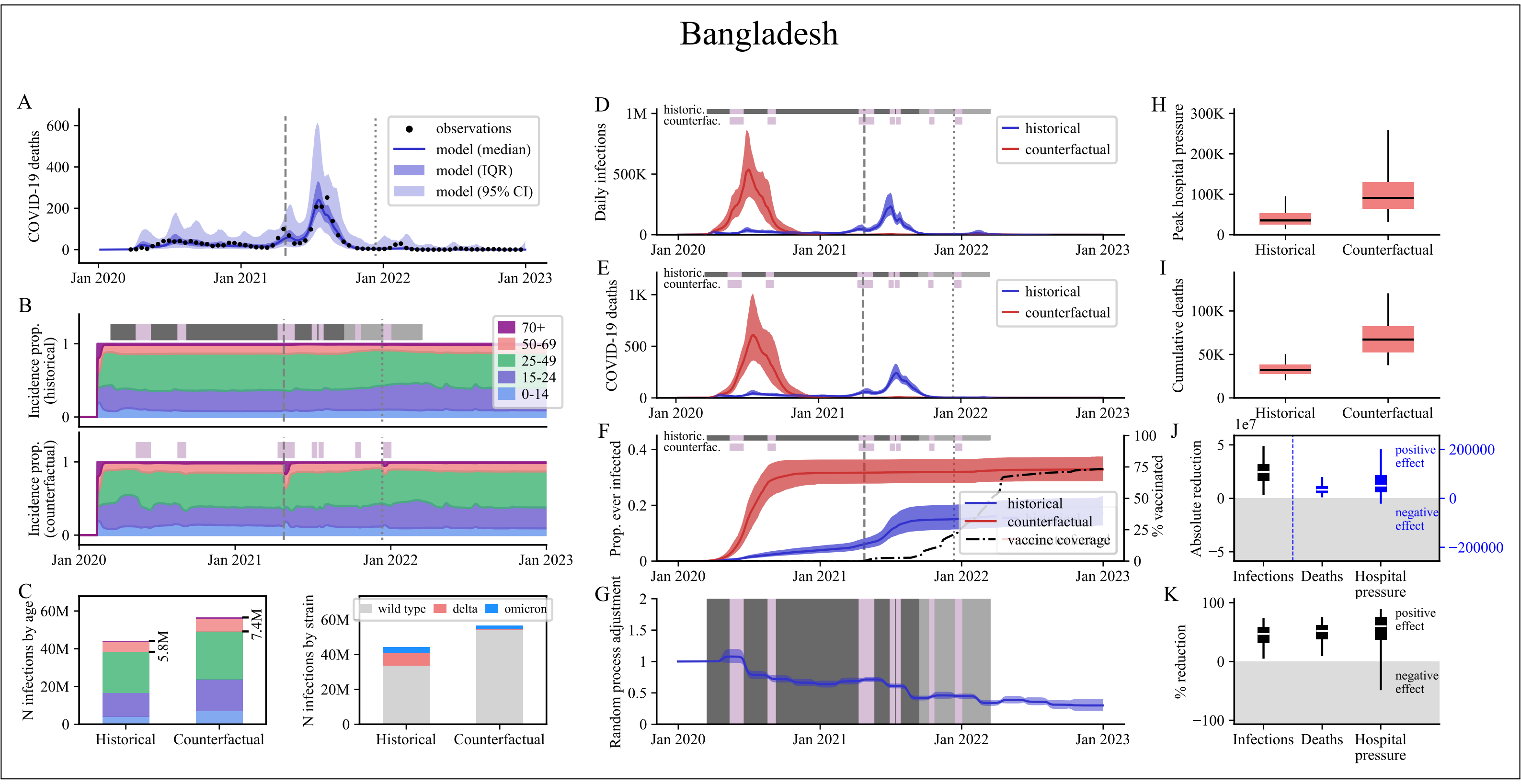
Bangladesh
A
COVID-19 deaths
observations
model (median)
model (IQR)
model (95% CI)
B
Incidence prop. (historical)
Incidence prop. (counterfactual)
70+
50-69
25-49
15-24
0-14
C
N infections by age
5.8M
7.4M
N infections by strain
wild type
delta
omicron
Historical
Counterfactual
D
Daily infections
historic.
counterfac.
historical
counterfactual
E
COVID-19 deaths
F
Prop. ever infected
% vaccinated
vaccine coverage
G
Random process adjustment
H
Peak hospital pressure
I
Cumulative deaths
J
Absolute reduction
positive effect
negative effect
Infections
Deaths
Hospital pressure
K
% reduction
Jan 2020
Jan 2021
Jan 2022
Jan 2023

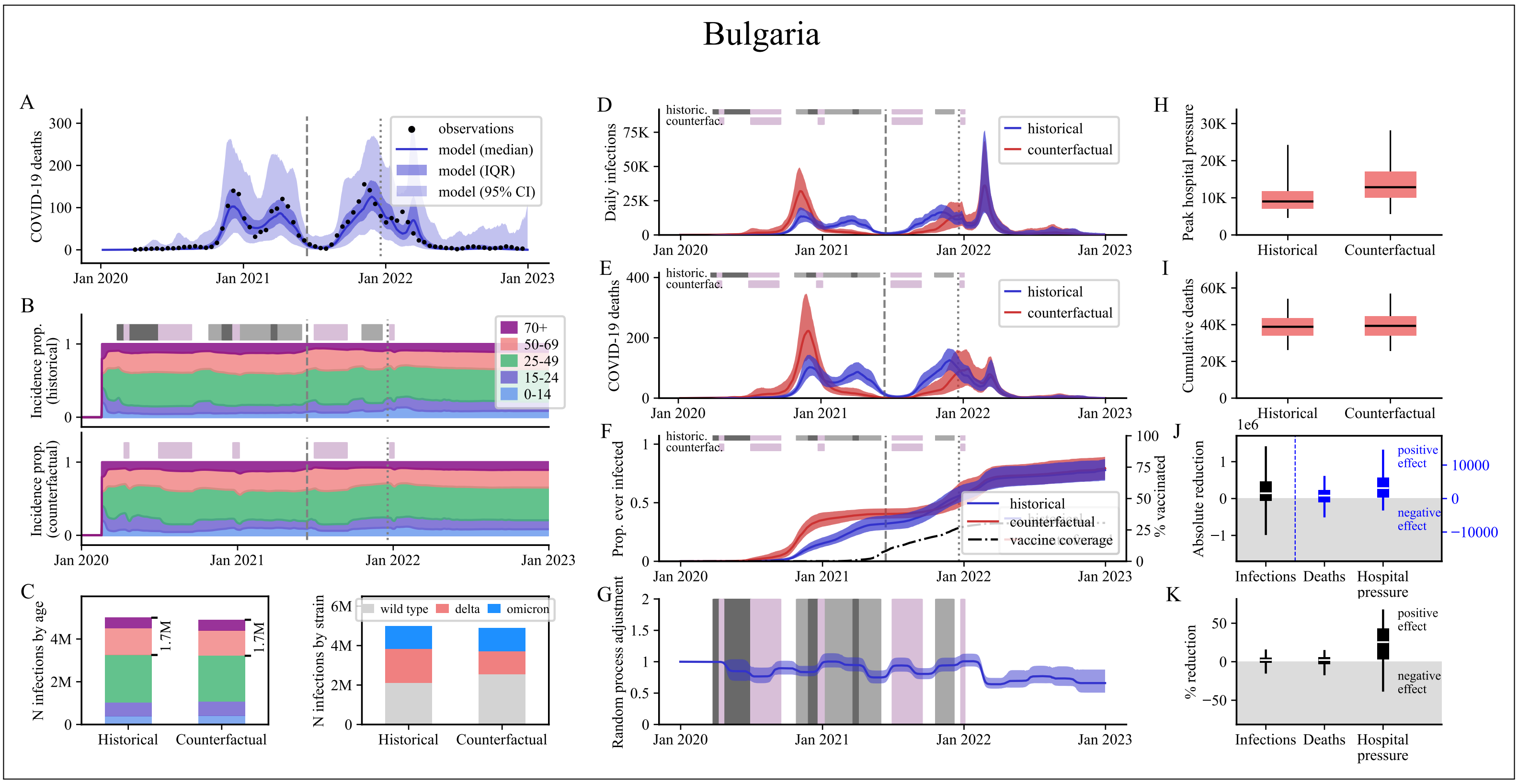
Bulgaria
A
COVID-19 deaths
observations
model (median)
model (IQR)
model (95% CI)
B
Incidence prop. (historical)
Incidence prop. (counterfactual)
70+
50-69
25-49
15-24
0-14
C
N infections by age
1.7M
1.7M
N infections by strain
wild type
delta
omicron
Historical
Counterfactual
D
Daily infections
historic.
counterfac.
historical
counterfactual
E
COVID-19 deaths
F
Prop. ever infected
% vaccinated
vaccine coverage
G
Random process adjustment
H
Peak hospital pressure
I
Cumulative deaths
J
Absolute reduction
positive effect
negative effect
Infections
Deaths
Hospital pressure
K
% reduction
Jan 2020
Jan 2021
Jan 2022
Jan 2023

Bosnia and Herzegovina

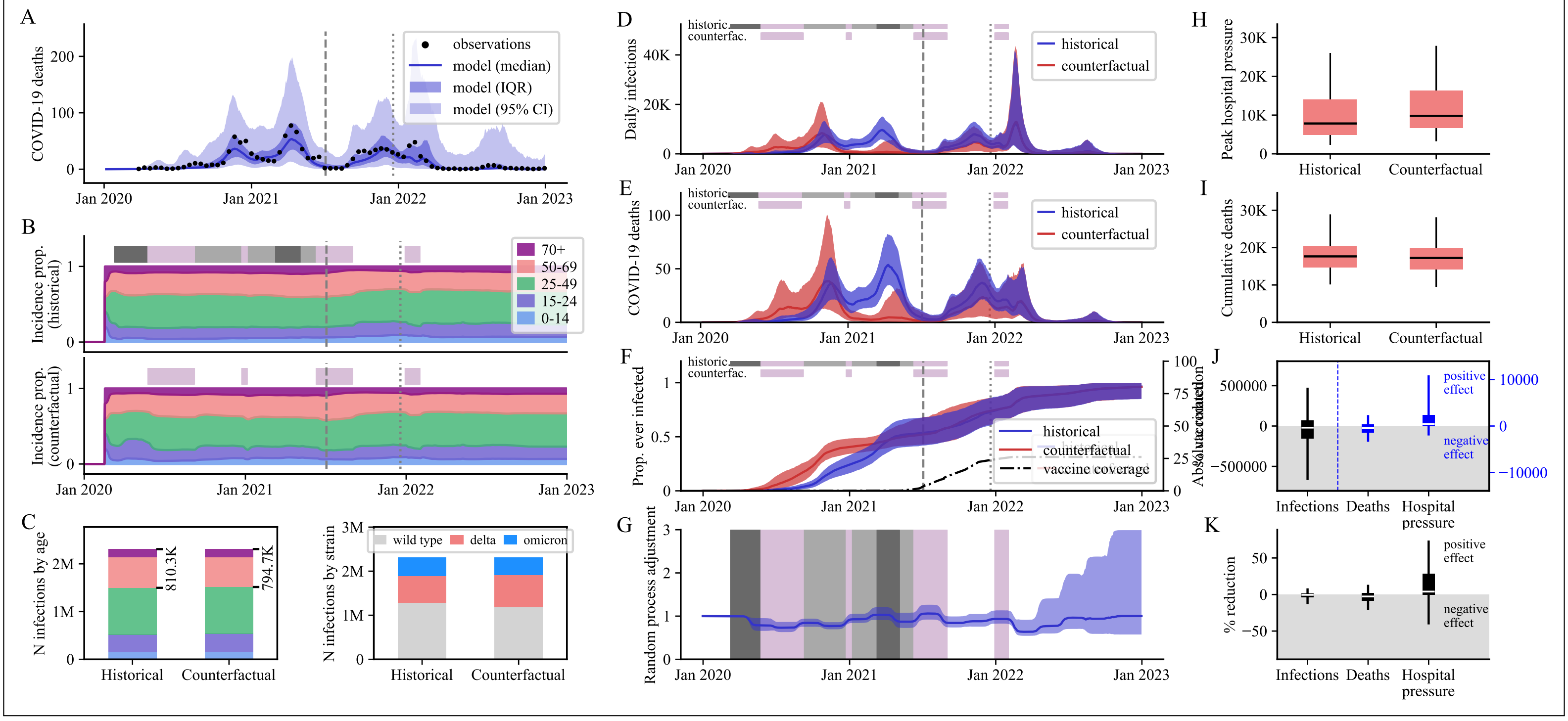

Bolivia Plurinational State of

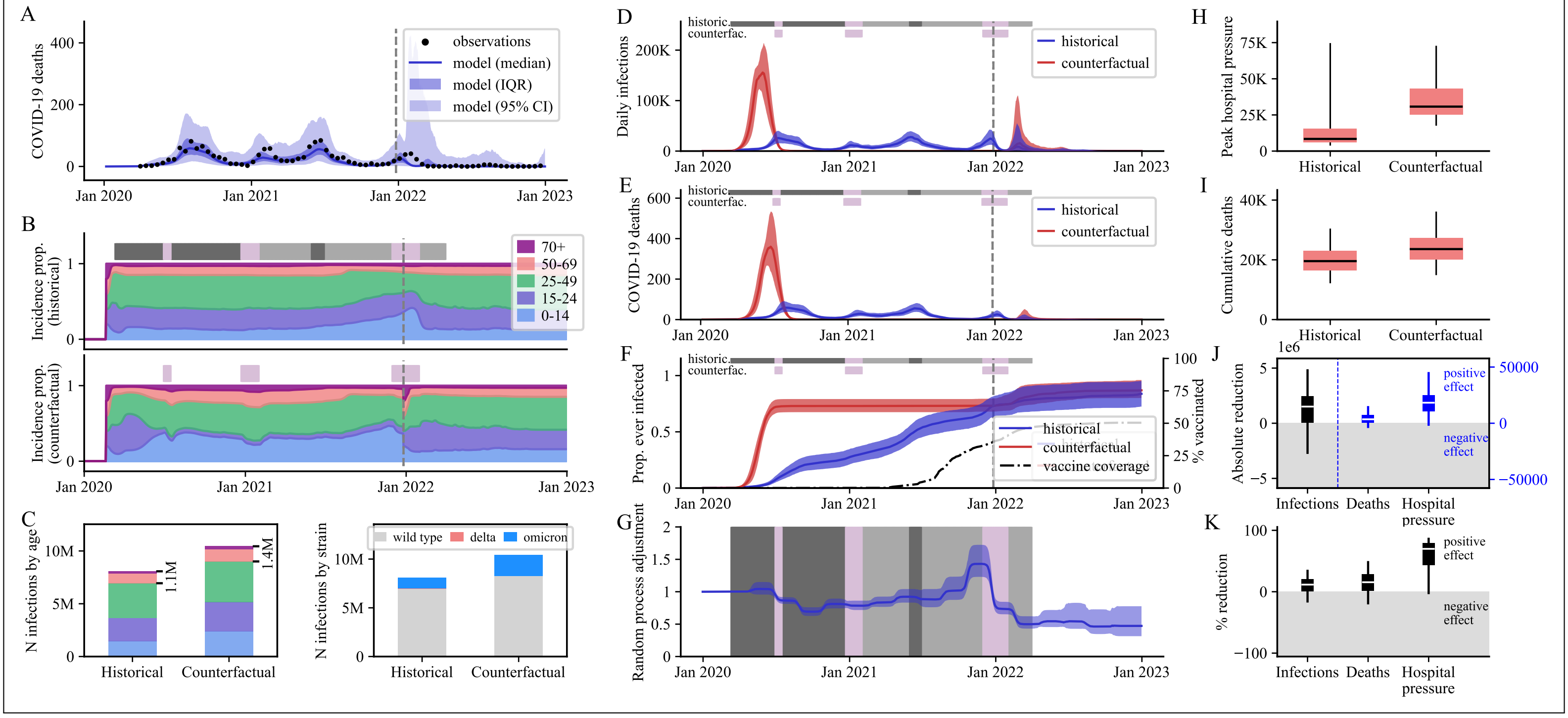

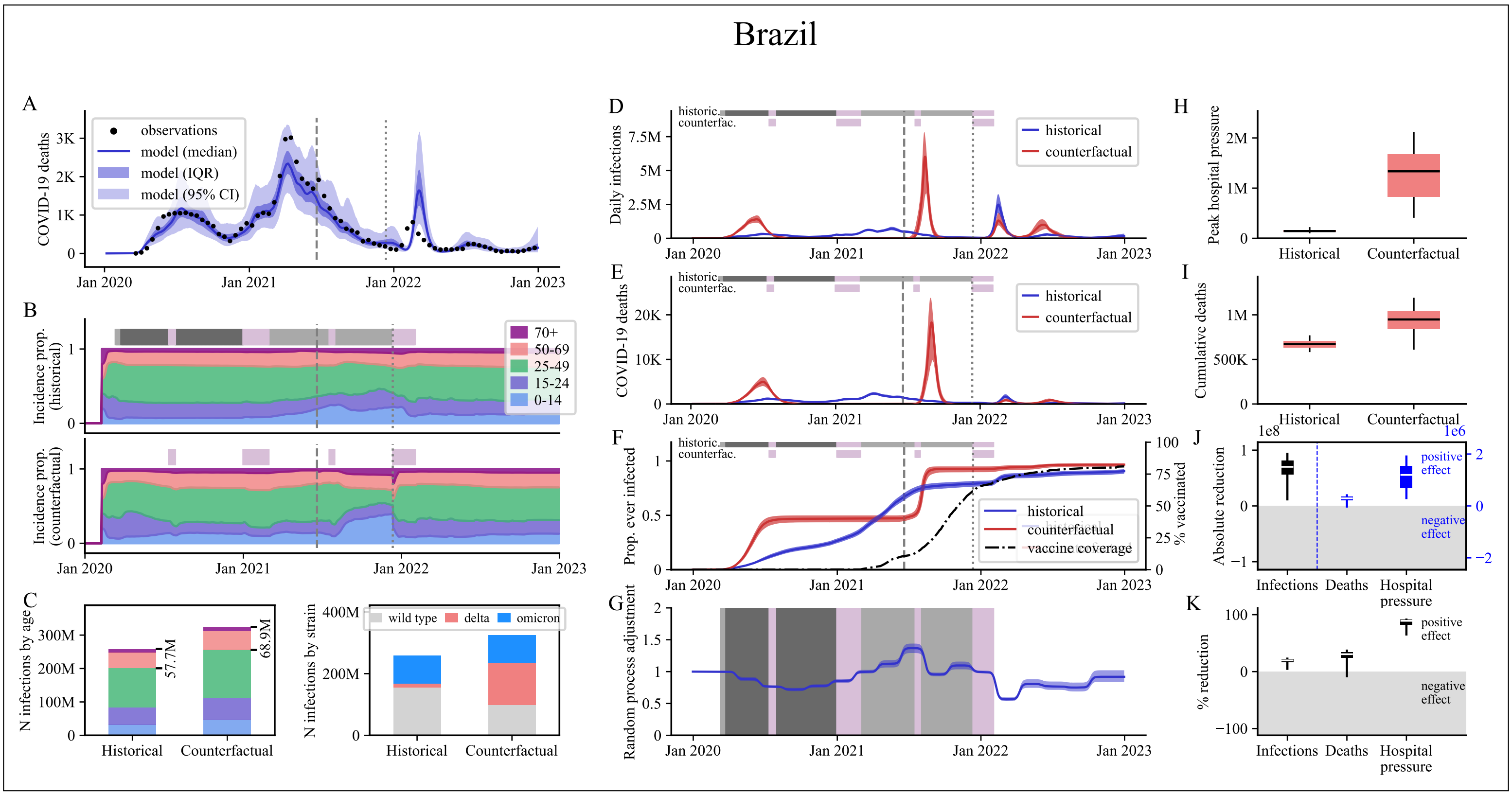

Brazil
A
COVID-19 deaths
observations
model (median)
model (IQR)
model (95% CI)
B
Incidence prop. (historical)
Incidence prop. (counterfactual)
70+
50-69
25-49
15-24
0-14
C
N infections by age
57.7M
68.9M
N infections by strain
wild type
delta
omicron
Historical
Counterfactual
D
Daily infections
historic.
counterfac.
historical
counterfactual
E
COVID-19 deaths
F
Prop. ever infected
% vaccinated
vaccine coverage
G
Random process adjustment
Jan 2020
Jan 2021
Jan 2022
Jan 2023
H
Peak hospital pressure
I
Cumulative deaths
J
Absolute reduction
1e8
1e6
positive effect
negative effect
K
% reduction
Infections
Deaths
Hospital pressure

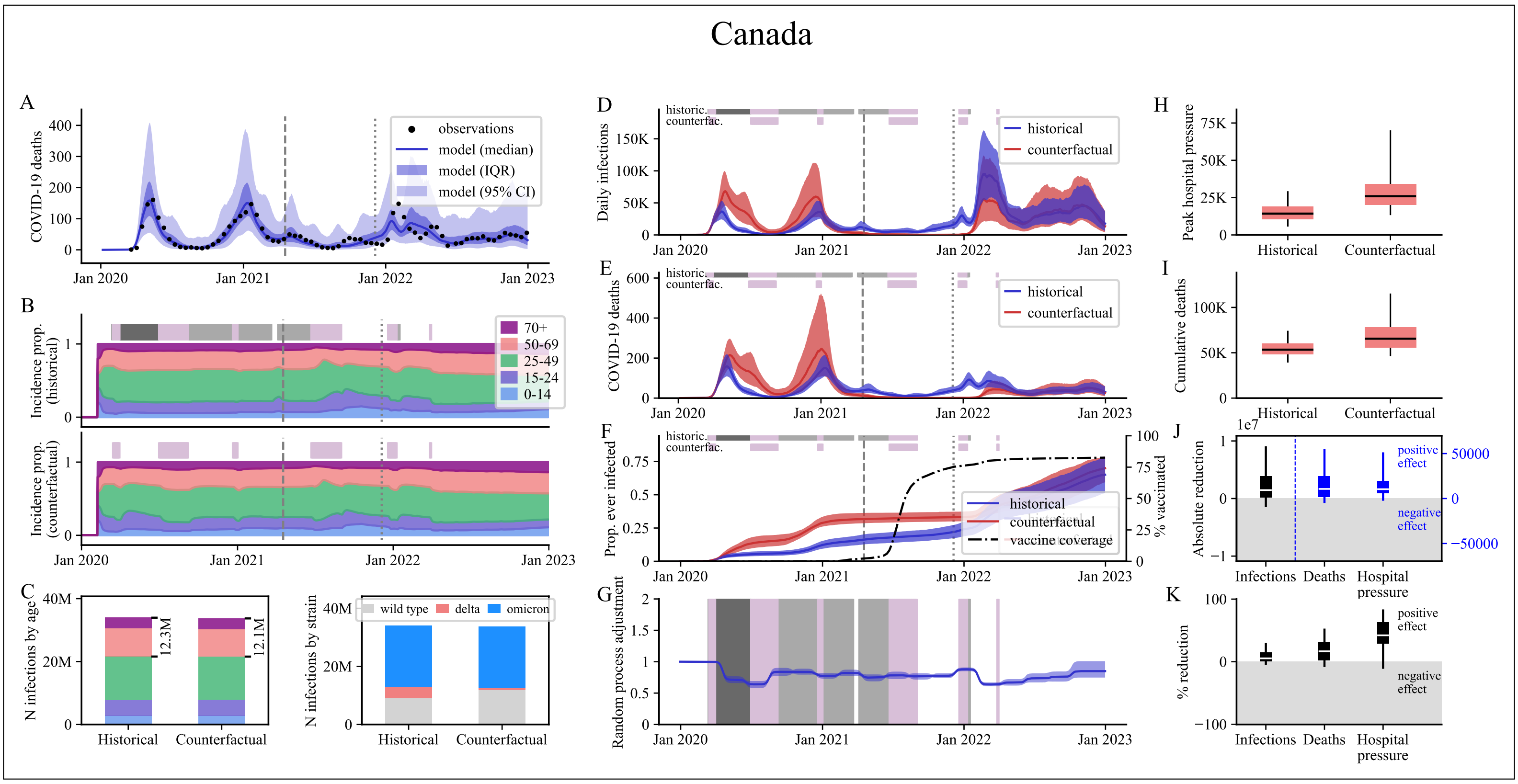

Canada
A
COVID-19 deaths
observations
model (median)
model (IQR)
model (95% CI)
B
Incidence prop. (historical)
Incidence prop. (counterfactual)
70+
50-69
25-49
15-24
0-14
C
N infections by age
12.3M
12.1M
N infections by strain
wild type
delta
omicron
Historical
Counterfactual
D
Daily infections
historic.
counterfac.
historical
counterfactual
E
COVID-19 deaths
F
Prop. ever infected
% vaccinated
vaccine coverage
G
Random process adjustment
Jan 2020
Jan 2021
Jan 2022
Jan 2023
H
Peak hospital pressure
I
Cumulative deaths
J
Absolute reduction
1e7
positive effect
negative effect
K
% reduction
Infections
Deaths
Hospital pressure

Chile

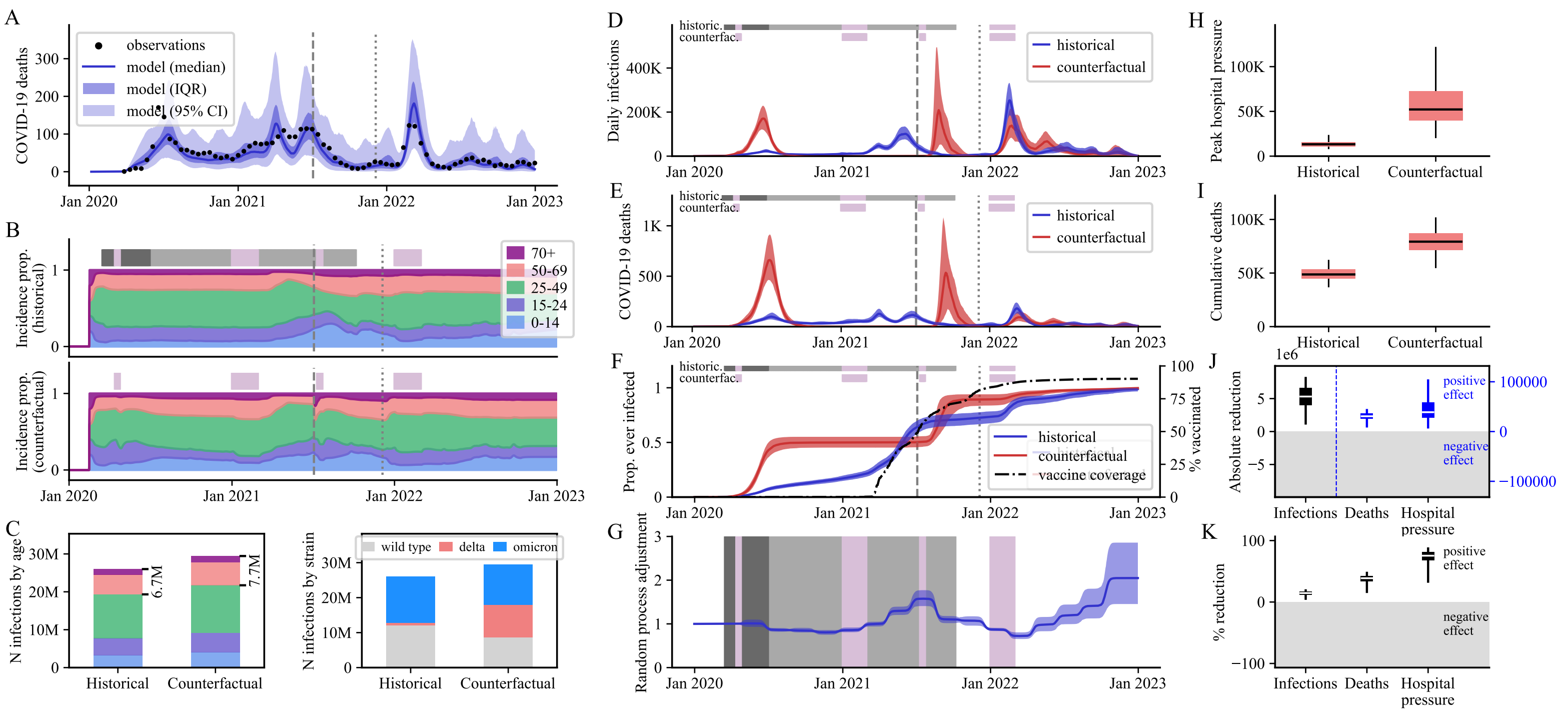

Colombia

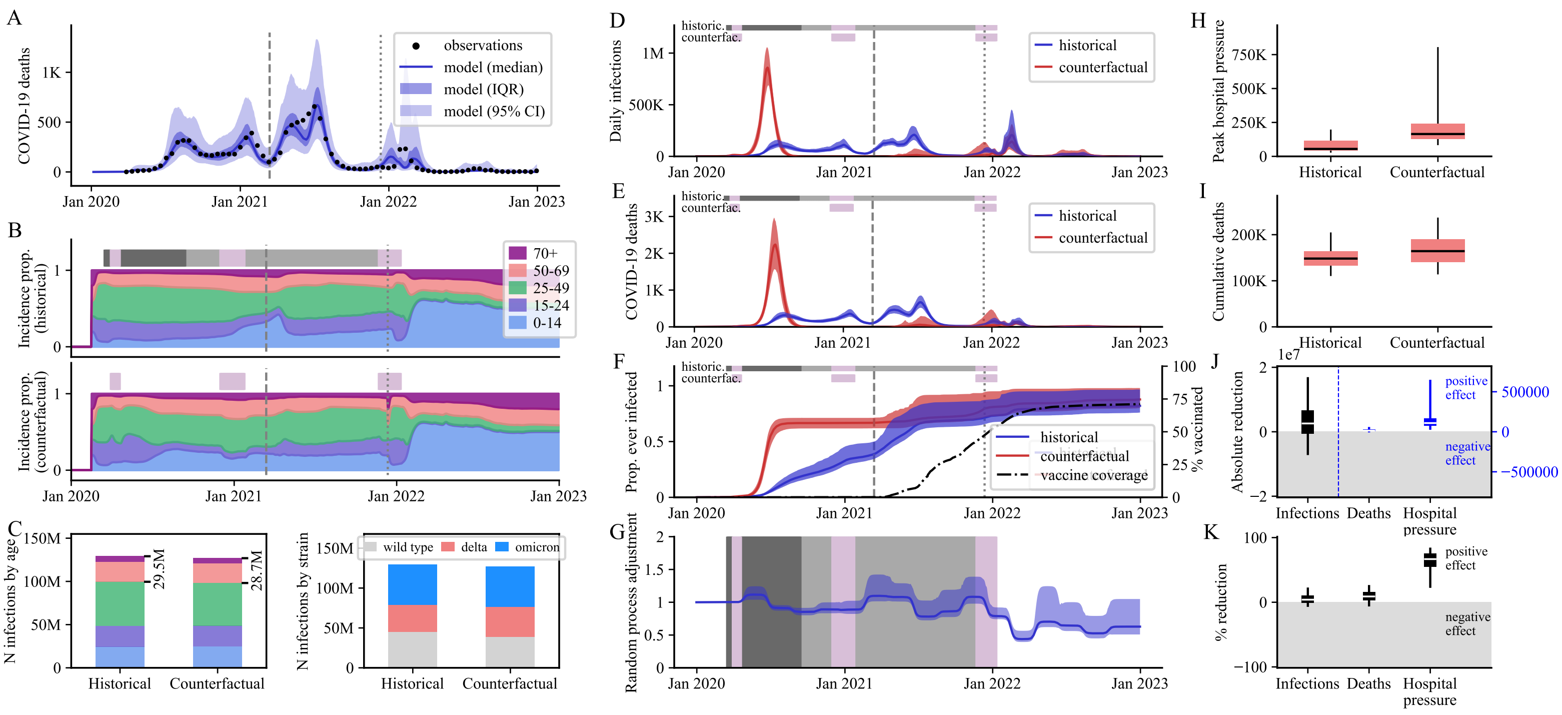

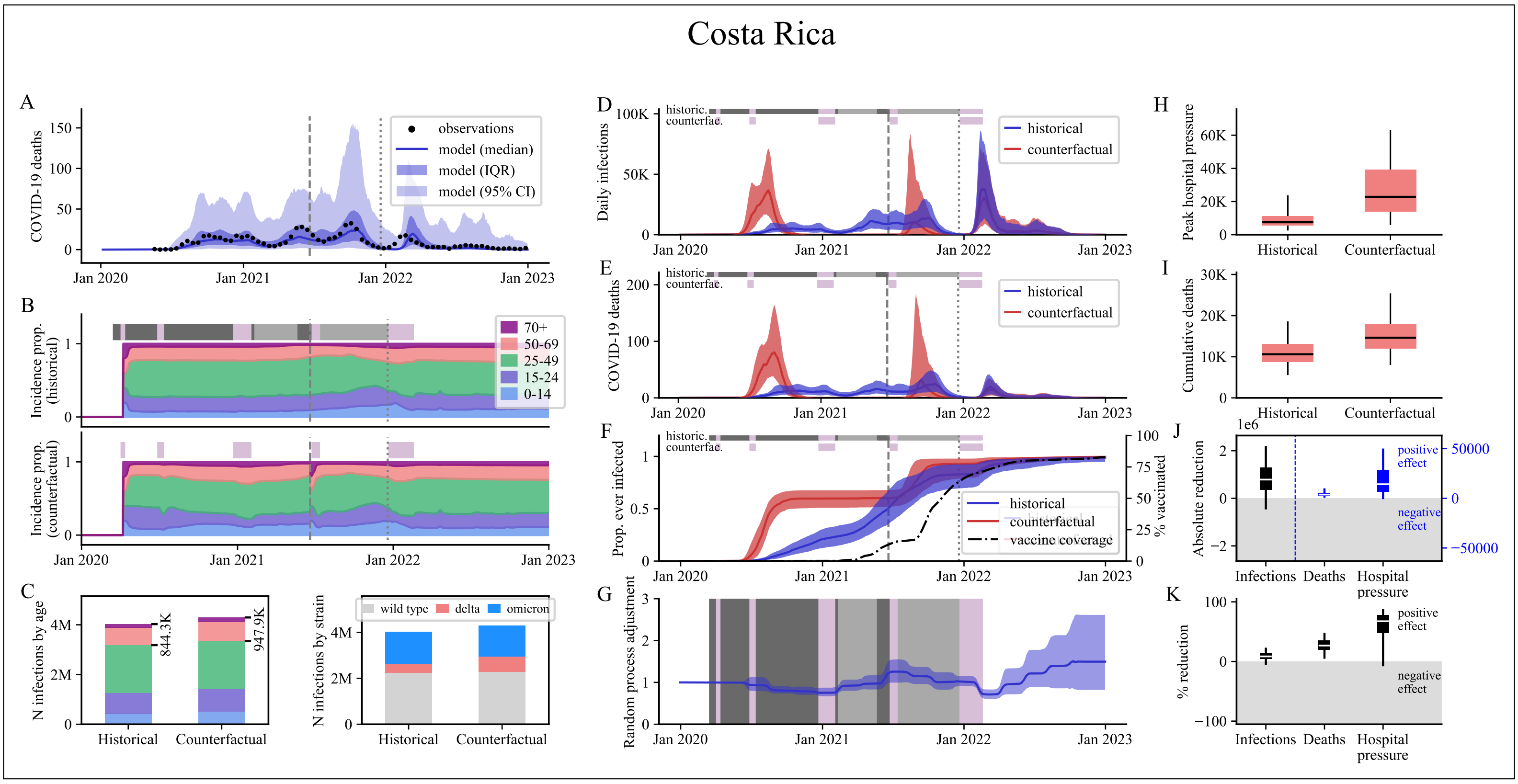

Costa Rica
A
COVID-19 deaths
observations
model (median)
model (IQR)
model (95% CI)
Jan 2020
Jan 2021
Jan 2022
Jan 2023
B
Incidence prop. (historical)
Incidence prop. (counterfactual)
70+
50-69
25-49
15-24
0-14
C
N infections by age
844.3K
947.9K
Historical
Counterfactual
N infections by strain
wild type
delta
omicron
D
Daily infections
historic.
counterfac.
historical
counterfactual
E
COVID-19 deaths
F
Prop. ever infected
% vaccinated
vaccine coverage
G
Random process adjustment
H
Peak hospital pressure
I
Cumulative deaths
J
Absolute reduction
1e6
positive effect
negative effect
Infections
Deaths
Hospital pressure
K
% reduction

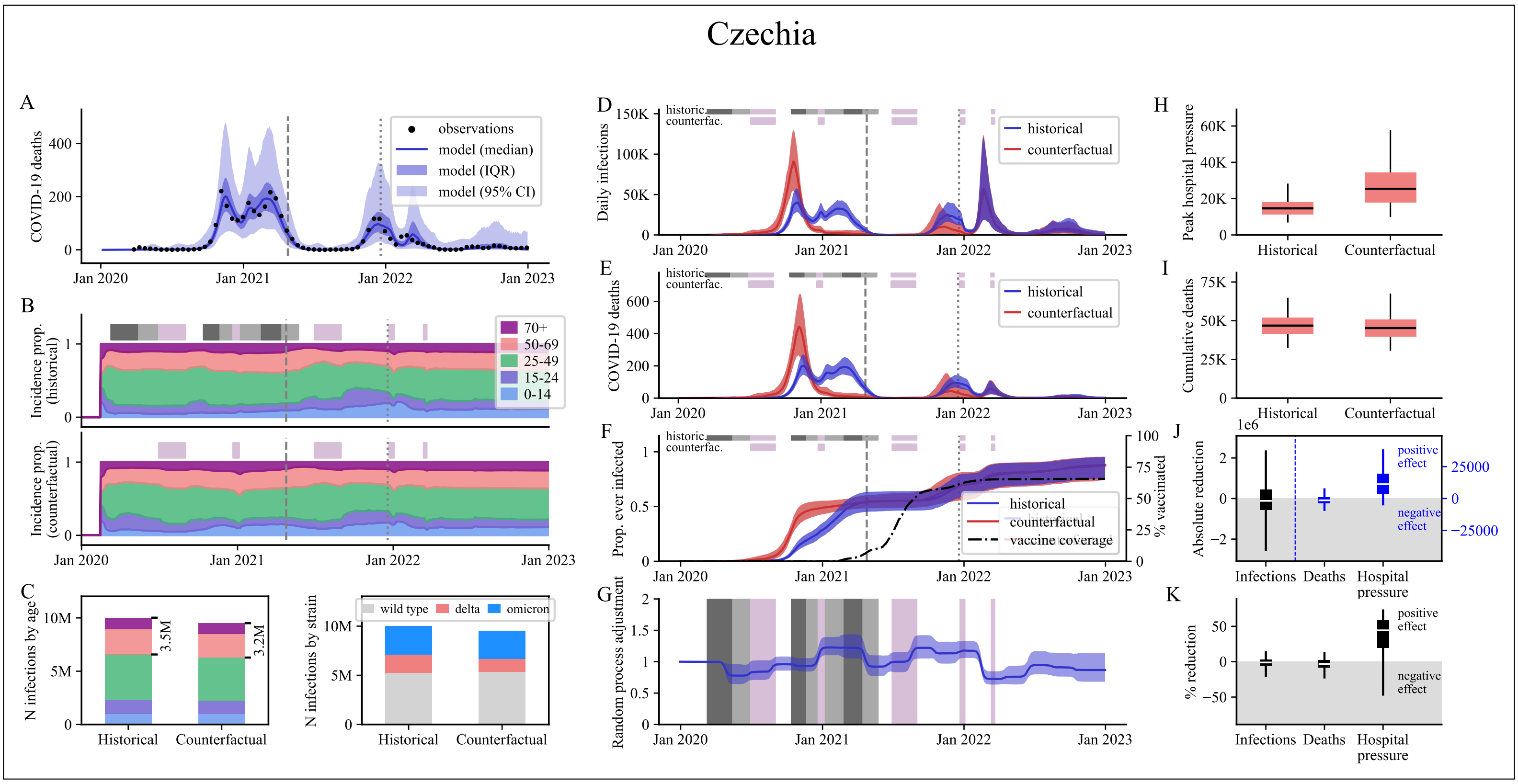

Czechia
A
COVID-19 deaths
observations
model (median)
model (IQR)
model (95% CI)
Jan 2020
Jan 2021
Jan 2022
Jan 2023
B
Incidence prop. (historical)
Incidence prop. (counterfactual)
70+
50-69
25-49
15-24
0-14
C
N infections by age
3.5M
3.2M
Historical
Counterfactual
N infections by strain
wild type
delta
omicron
D
Daily infections
historic.
counterfac.
historical
counterfactual
E
COVID-19 deaths
F
Prop. ever infected
% vaccinated
vaccine coverage
G
Random process adjustment
H
Peak hospital pressure
I
Cumulative deaths
J
Absolute reduction
1e6
positive effect
negative effect
Infections
Deaths
Hospital pressure
K
% reduction

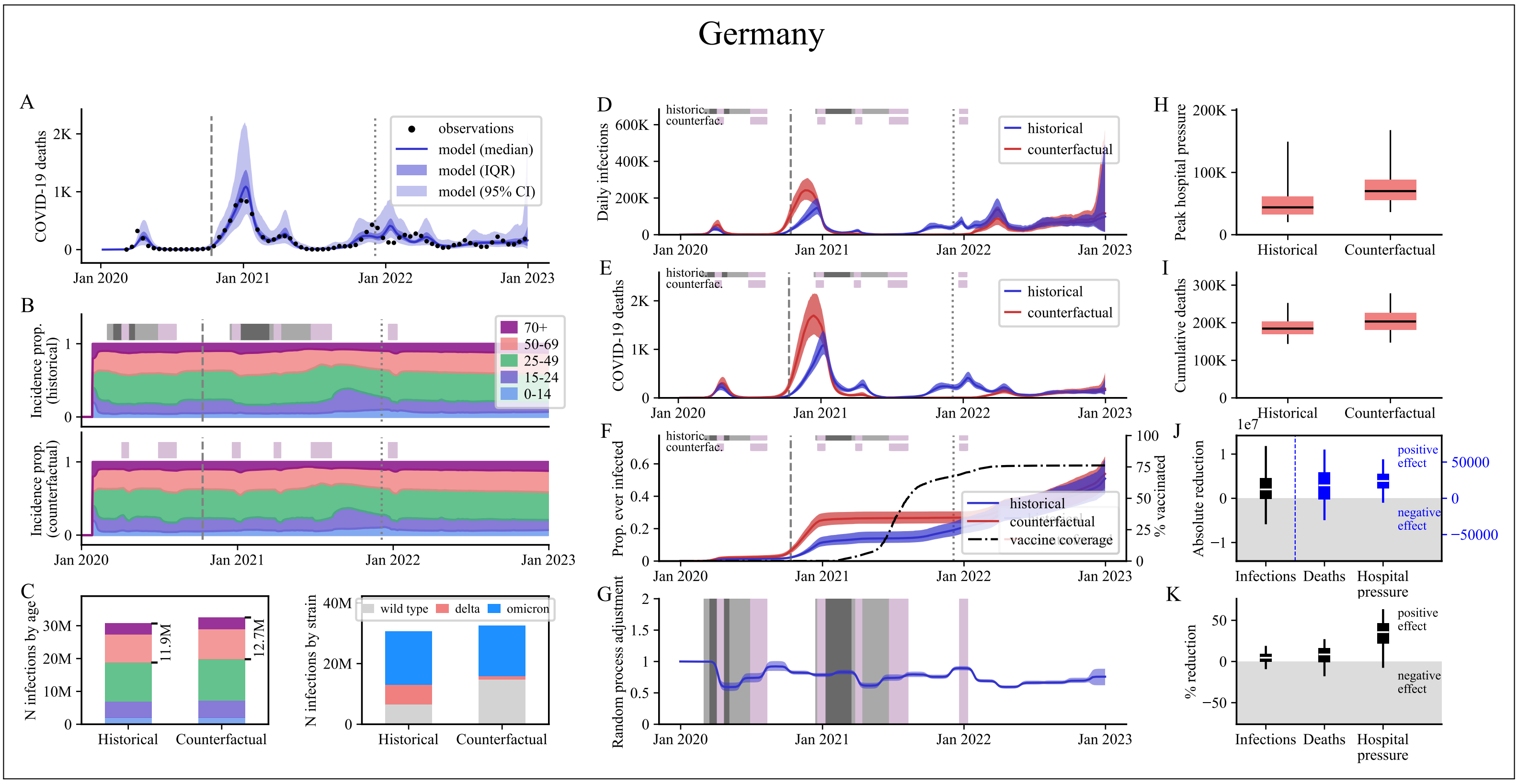
Germany
A
COVID-19 deaths
observations
model (median)
model (IQR)
model (95% CI)
Jan 2020
Jan 2021
Jan 2022
Jan 2023
B
Incidence prop. (historical)
Incidence prop. (counterfactual)
70+
50-69
25-49
15-24
0-14
C
N infections by age
11.9M
12.7M
N infections by strain
wild type
delta
omicron
Historical
Counterfactual
D
Daily infections
historic.
counterfac.
historical
counterfactual
E
COVID-19 deaths
F
Prop. ever infected
% vaccinated
vaccine coverage
G
Random process adjustment
H
Peak hospital pressure
I
Cumulative deaths
J
Absolute reduction
1e7
positive effect
negative effect
Infections
Deaths
Hospital pressure
K
% reduction

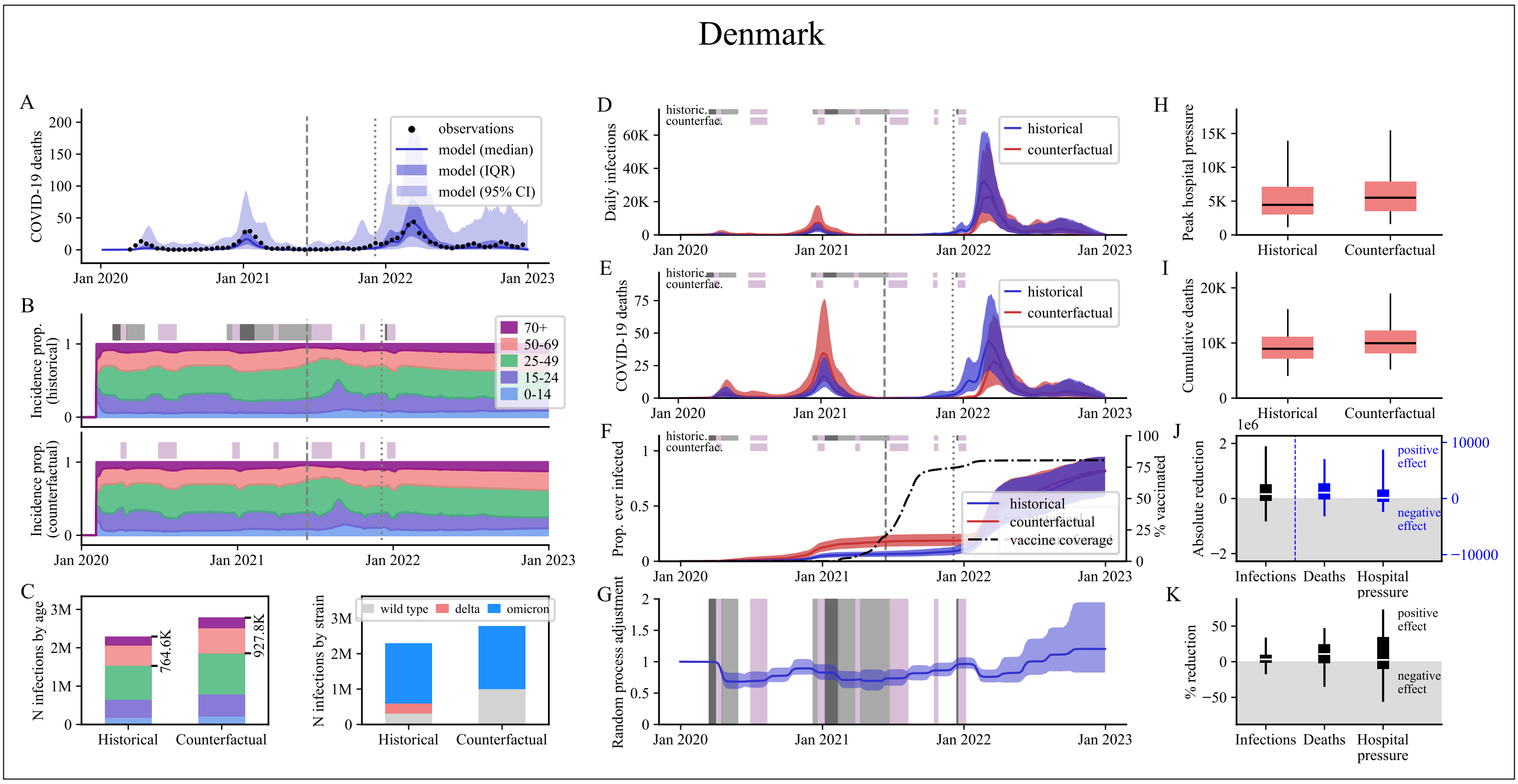
Denmark
A
COVID-19 deaths
observations
model (median)
model (IQR)
model (95% CI)
Jan 2020
Jan 2021
Jan 2022
Jan 2023
B
Incidence prop. (historical)
Incidence prop. (counterfactual)
70+
50-69
25-49
15-24
0-14
C
N infections by age
764.6K
927.8K
N infections by strain
wild type
delta
omicron
Historical
Counterfactual
D
Daily infections
historic.
counterfac.
historical
counterfactual
E
COVID-19 deaths
F
Prop. ever infected
% vaccinated
vaccine coverage
G
Random process adjustment
H
Peak hospital pressure
I
Cumulative deaths
J
Absolute reduction
1e6
positive effect
negative effect
Infections
Deaths
Hospital pressure
K
% reduction

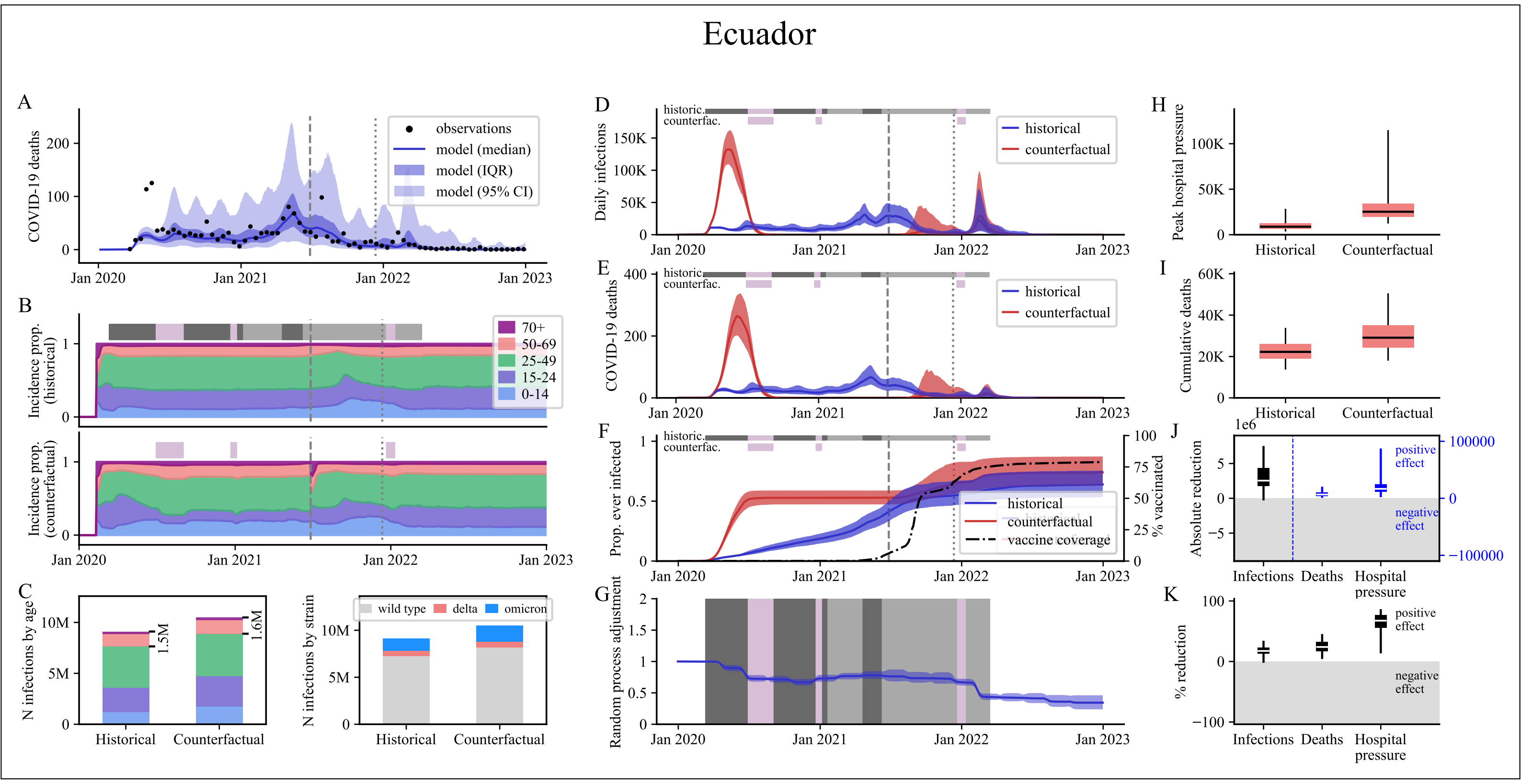

Ecuador
A
B
C
D
E
F
G
H
I
J
K
COVID-19 deaths
observations
model (median)
model (IQR)
model (95% CI)
Incidence prop. (historical)
Incidence prop. (counterfactual)
70+
50-69
25-49
15-24
0-14
N infections by age
1.5M
1.6M
N infections by strain
wild type
delta
omicron
Historical
Counterfactual
Daily infections
historic.
counterfac.
historical
counterfactual
Prop. ever infected
% vaccinated
vaccine coverage
Random process adjustment
Peak hospital pressure
Cumulative deaths
Absolute reduction
1e6
positive effect
negative effect
% reduction
Infections
Deaths
Hospital pressure
Jan 2020
Jan 2021
Jan 2022
Jan 2023

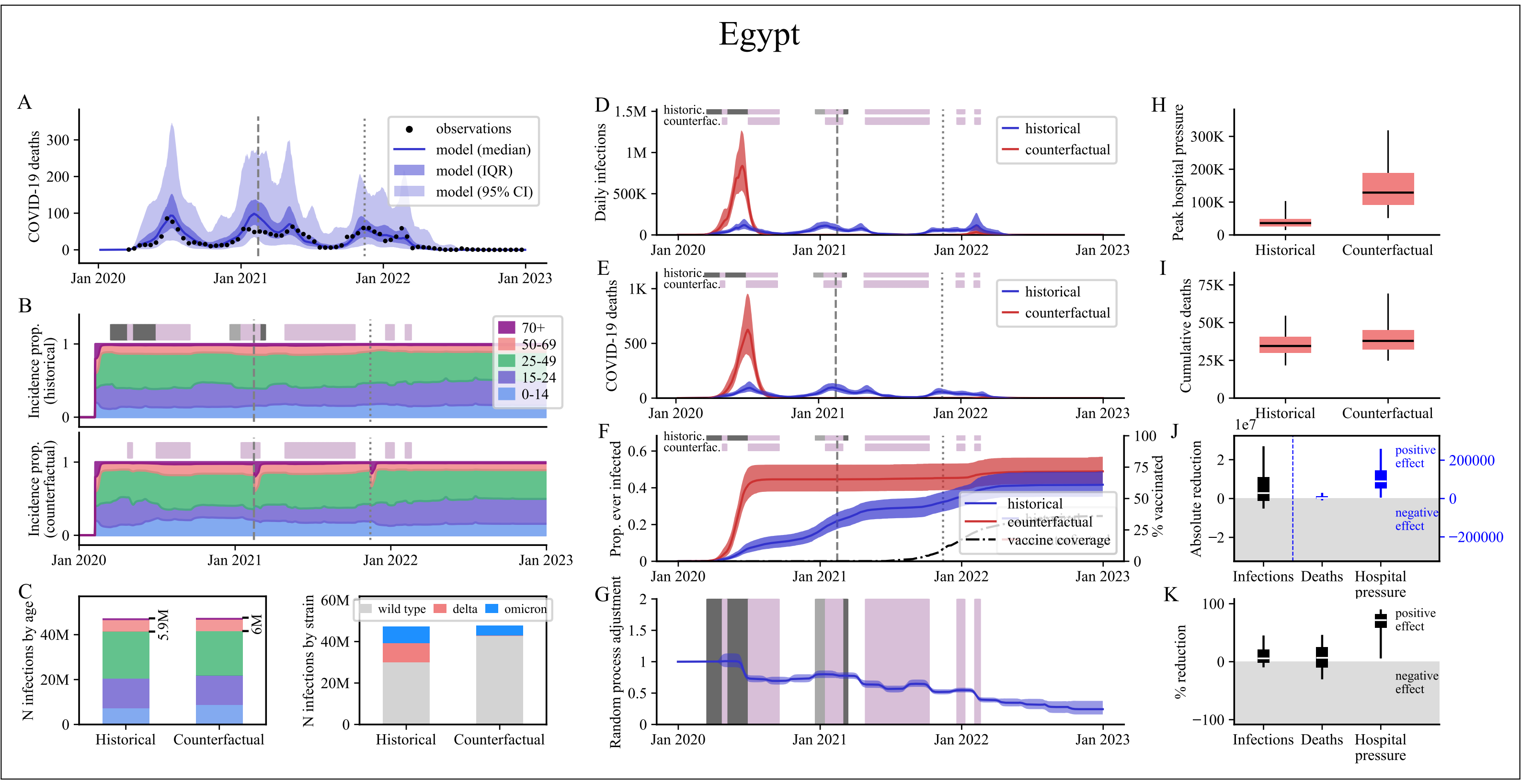

Egypt
A
B
C
D
E
F
G
H
I
J
K
COVID-19 deaths
observations
model (median)
model (IQR)
model (95% CI)
Incidence prop. (historical)
Incidence prop. (counterfactual)
70+
50-69
25-49
15-24
0-14
N infections by age
5.9M
6M
N infections by strain
wild type
delta
omicron
Historical
Counterfactual
Daily infections
historic.
counterfac.
historical
counterfactual
Prop. ever infected
% vaccinated
vaccine coverage
Random process adjustment
Peak hospital pressure
Cumulative deaths
Absolute reduction
1e7
positive effect
negative effect
% reduction
Infections
Deaths
Hospital pressure
Jan 2020
Jan 2021
Jan 2022
Jan 2023

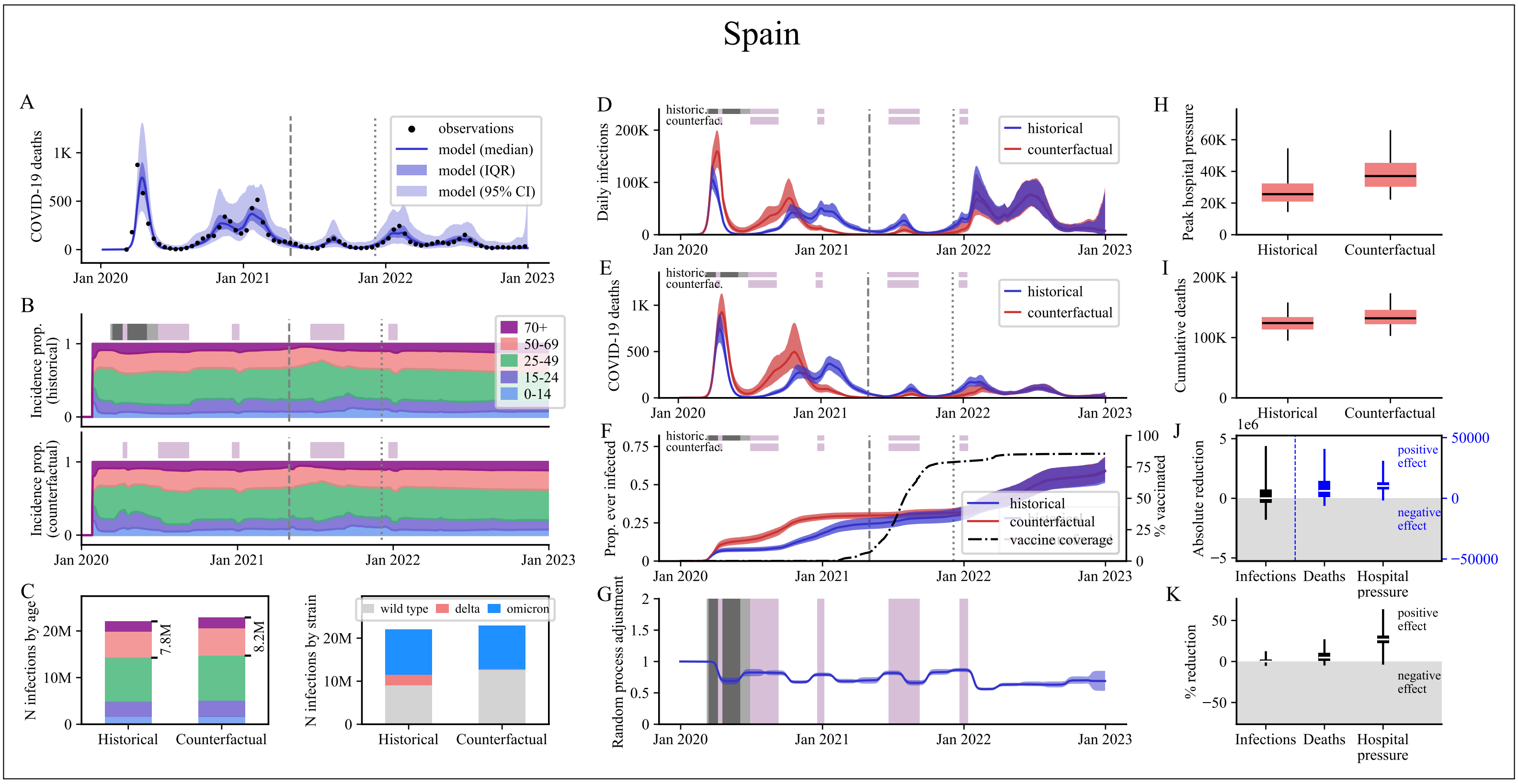

Spain
A
COVID-19 deaths
observations
model (median)
model (IQR)
model (95% CI)
Jan 2020
Jan 2021
Jan 2022
Jan 2023
B
Incidence prop. (historical)
Incidence prop. (counterfactual)
70+
50-69
25-49
15-24
0-14
C
N infections by age
7.8M
8.2M
Historical
Counterfactual
N infections by strain
wild type
delta
omicron
D
historic.
counterfac.
Daily infections
historical
counterfactual
E
COVID-19 deaths
F
Prop. ever infected
% vaccinated
vaccine coverage
G
Random process adjustment
H
Peak hospital pressure
I
Cumulative deaths
J
Absolute reduction
1e6
positive effect
negative effect
Infections
Deaths
Hospital pressure
K
% reduction

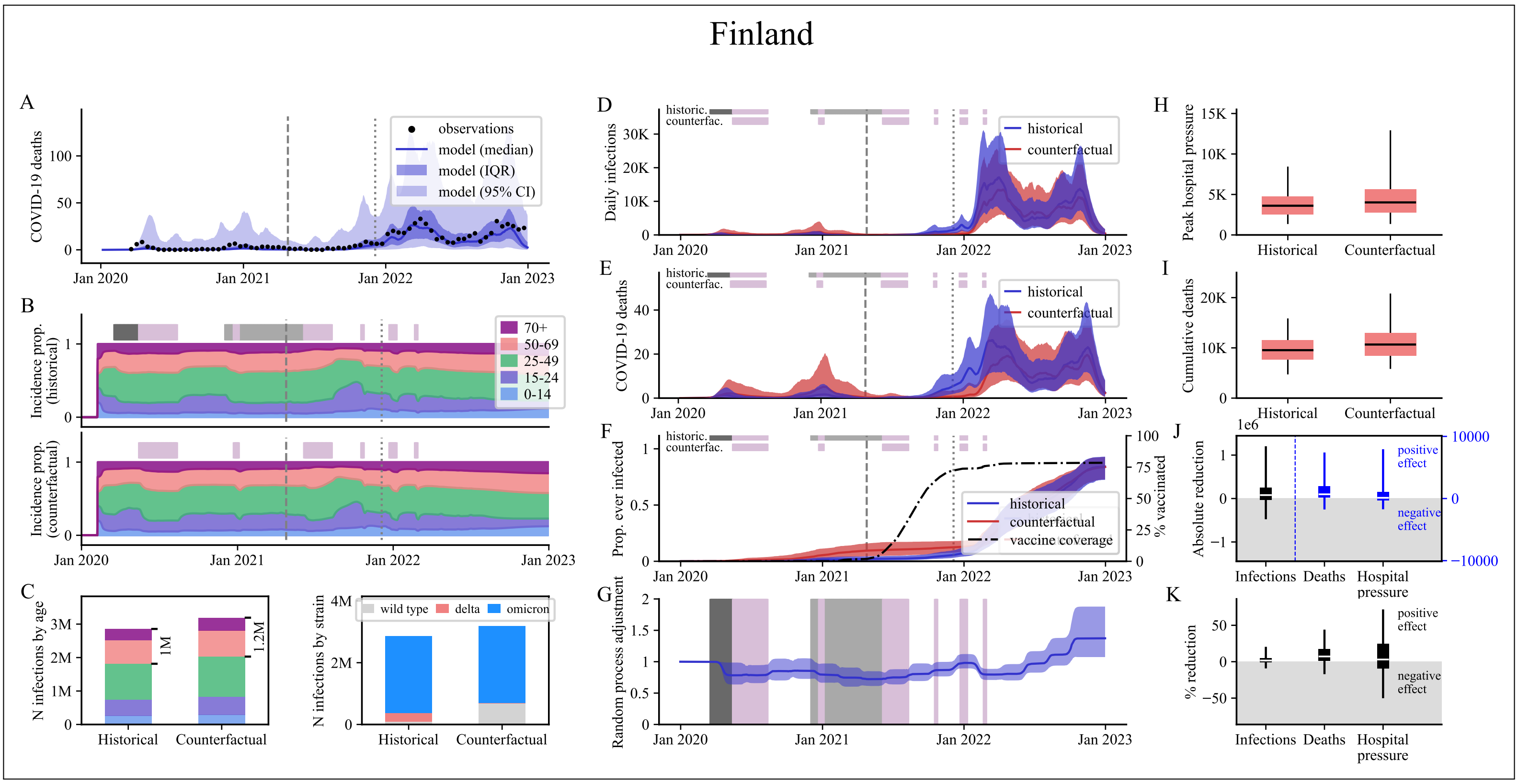

Finland
A
COVID-19 deaths
observations
model (median)
model (IQR)
model (95% CI)
Jan 2020
Jan 2021
Jan 2022
Jan 2023
B
Incidence prop. (historical)
Incidence prop. (counterfactual)
70+
50-69
25-49
15-24
0-14
C
N infections by age
1M
1.2M
Historical
Counterfactual
N infections by strain
wild type
delta
omicron
D
historic.
counterfac.
Daily infections
historical
counterfactual
E
COVID-19 deaths
F
Prop. ever infected
% vaccinated
vaccine coverage
G
Random process adjustment
H
Peak hospital pressure
I
Cumulative deaths
J
Absolute reduction
1e6
positive effect
negative effect
Infections
Deaths
Hospital pressure
K
% reduction

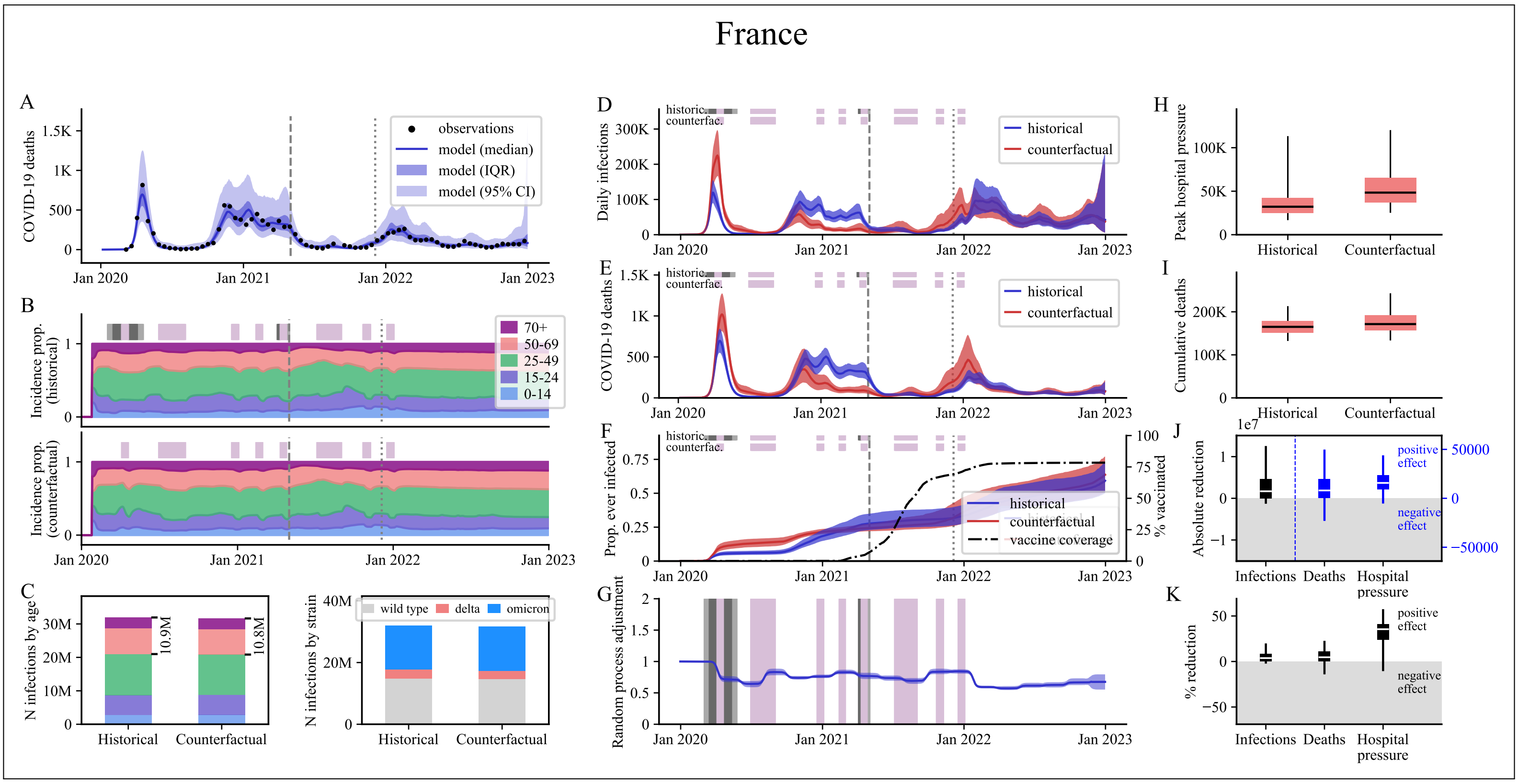
France
A
COVID-19 deaths
observations
model (median)
model (IQR)
model (95% CI)
Jan 2020
Jan 2021
Jan 2022
Jan 2023
B
Incidence prop. (historical)
Incidence prop. (counterfactual)
70+
50-69
25-49
15-24
0-14
C
N infections by age
10.9M
10.8M
Historical
Counterfactual
N infections by strain
wild type
delta
omicron
D
Daily infections
historical
counterfactual
E
COVID-19 deaths
F
Prop. ever infected
% vaccinated
vaccine coverage
G
Random process adjustment
H
Peak hospital pressure
I
Cumulative deaths
J
Absolute reduction
1e7
positive effect
negative effect
Infections
Deaths
Hospital pressure
K
% reduction

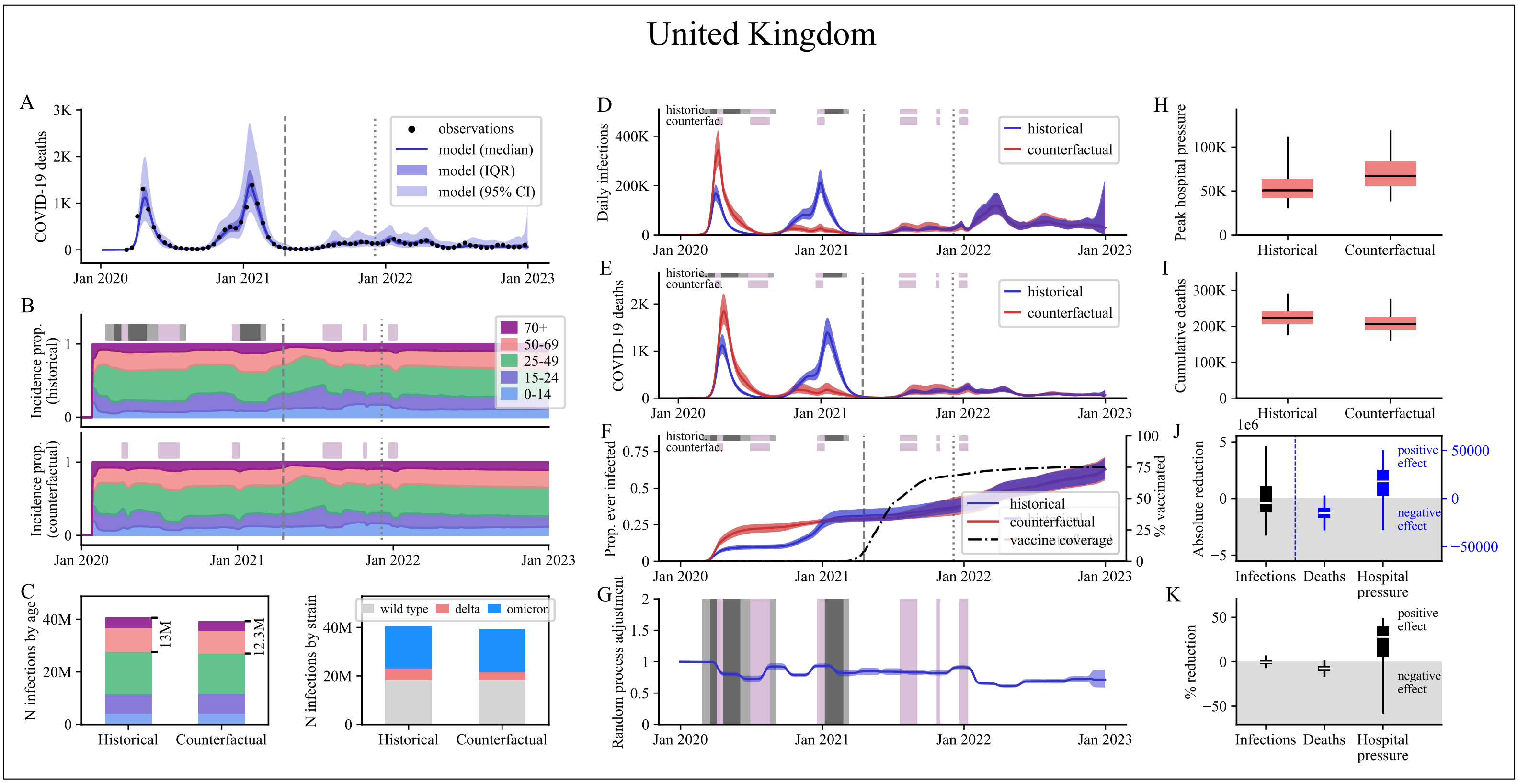
United Kingdom
A
COVID-19 deaths
observations
model (median)
model (IQR)
model (95% CI)
Jan 2020
Jan 2021
Jan 2022
Jan 2023
B
Incidence prop. (historical)
Incidence prop. (counterfactual)
70+
50-69
25-49
15-24
0-14
C
N infections by age
13M
12.3M
Historical
Counterfactual
N infections by strain
wild type
delta
omicron
D
Daily infections
historical
counterfactual
E
COVID-19 deaths
F
Prop. ever infected
% vaccinated
vaccine coverage
G
Random process adjustment
H
Peak hospital pressure
I
Cumulative deaths
J
Absolute reduction
1e6
positive effect
negative effect
Infections
Deaths
Hospital pressure
K
% reduction

Georgia

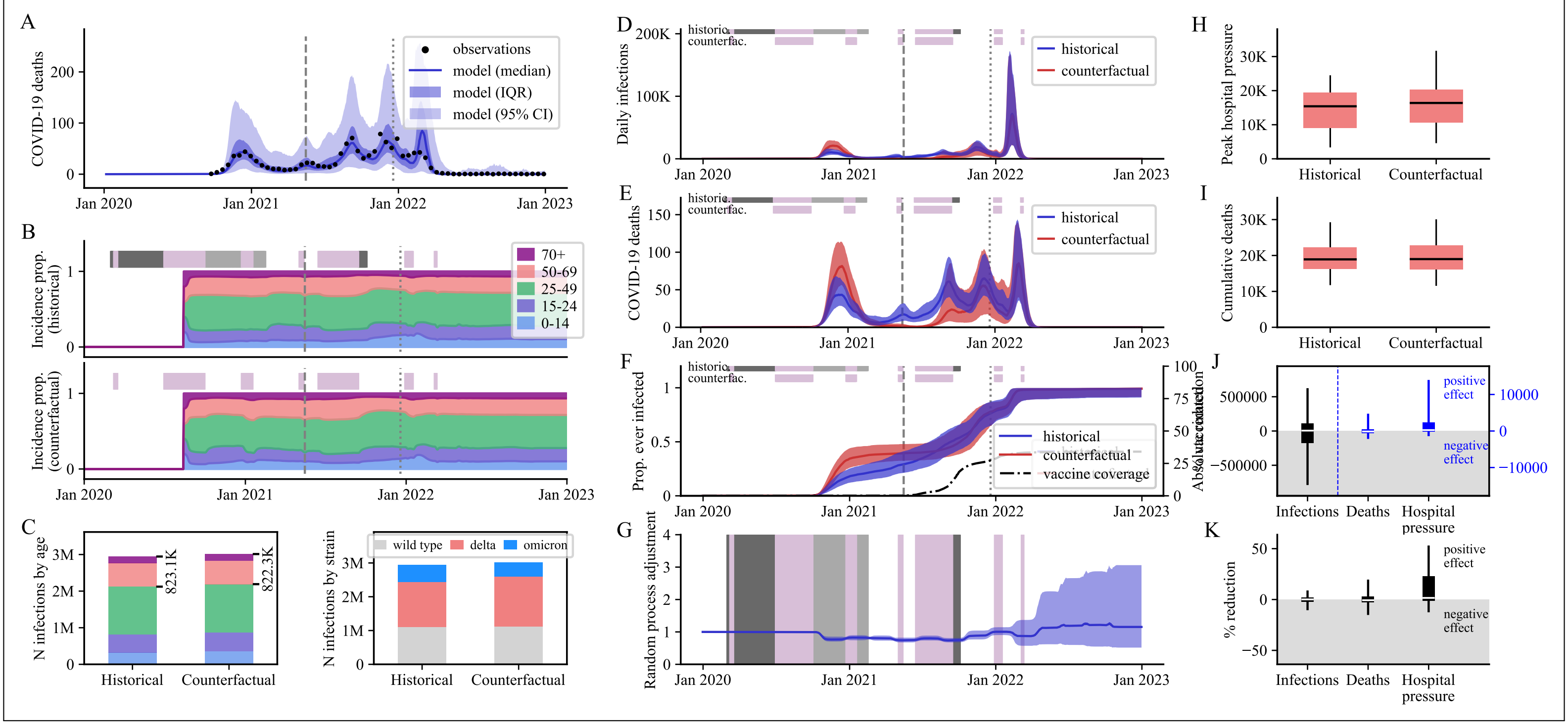

Greece

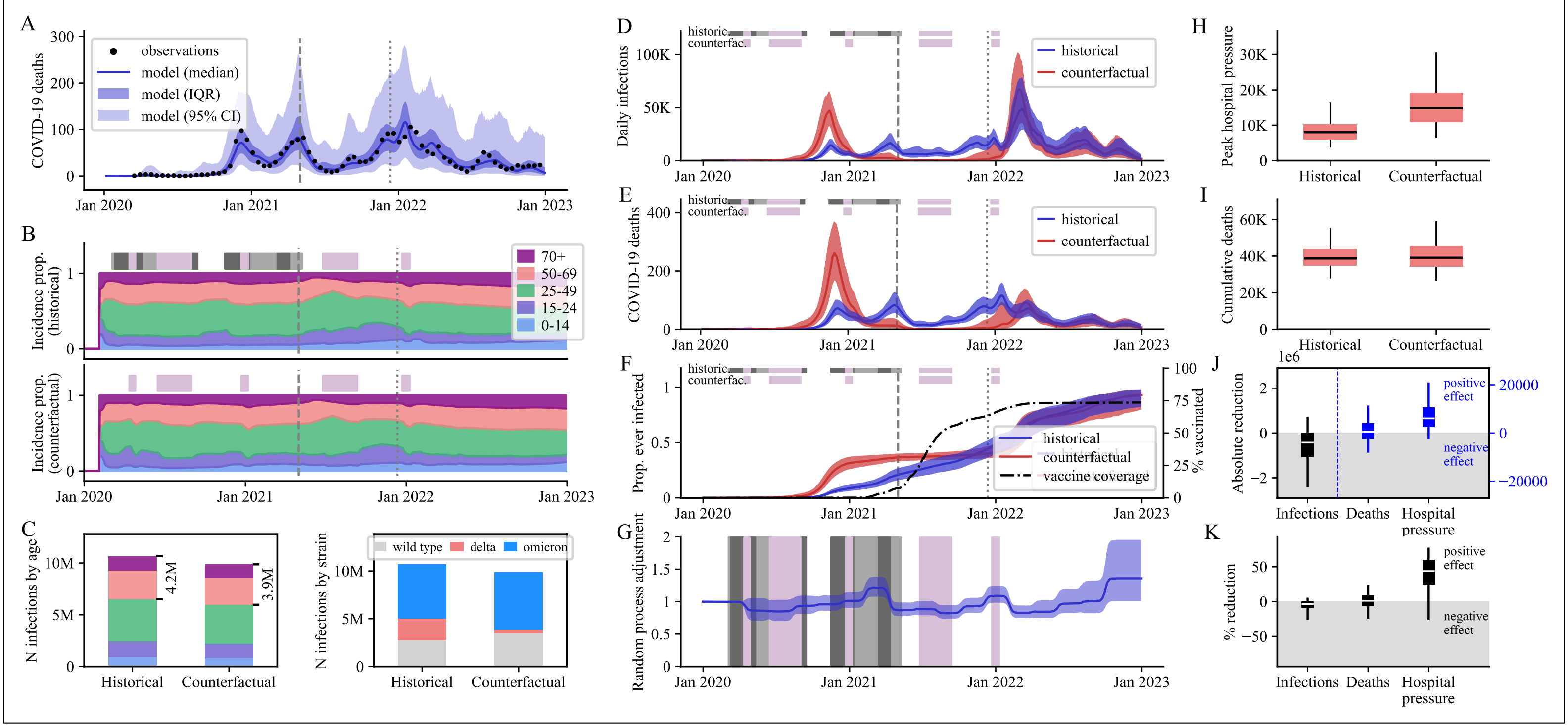

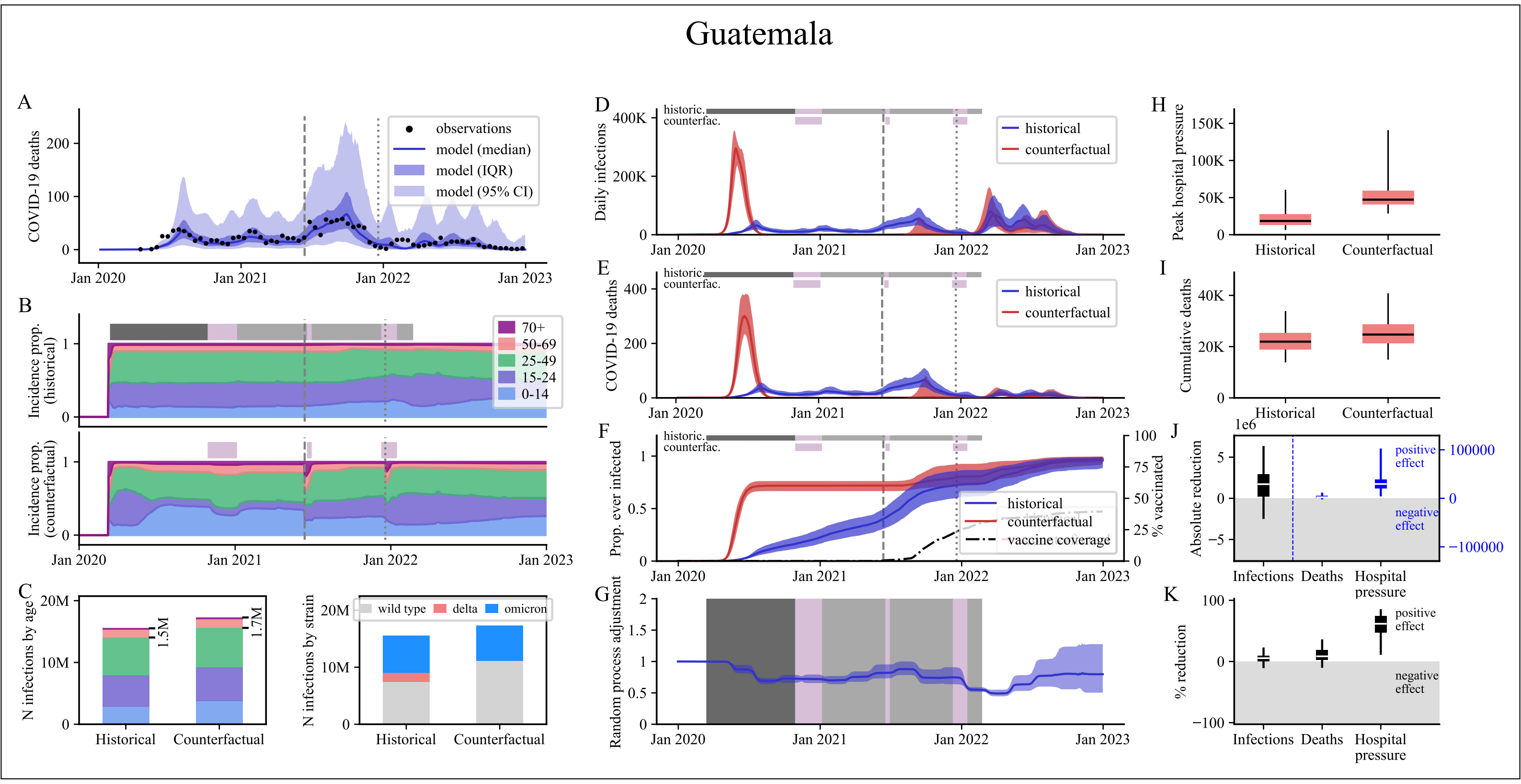
Guatemala
A
COVID-19 deaths
observations
model (median)
model (IQR)
model (95% CI)
B
Incidence prop. (historical)
Incidence prop. (counterfactual)
70+
50-69
25-49
15-24
0-14
C
N infections by age
1.5M
1.7M
N infections by strain
wild type
delta
omicron
Historical
Counterfactual
D
Daily infections
historic.
counterfac.
historical
counterfactual
E
COVID-19 deaths
F
Prop. ever infected
% vaccinated
vaccine coverage
G
Random process adjustment
H
Peak hospital pressure
I
Cumulative deaths
J
Absolute reduction
1e6
positive effect
negative effect
Infections
Deaths
Hospital pressure
K
% reduction
Jan 2020
Jan 2021
Jan 2022
Jan 2023

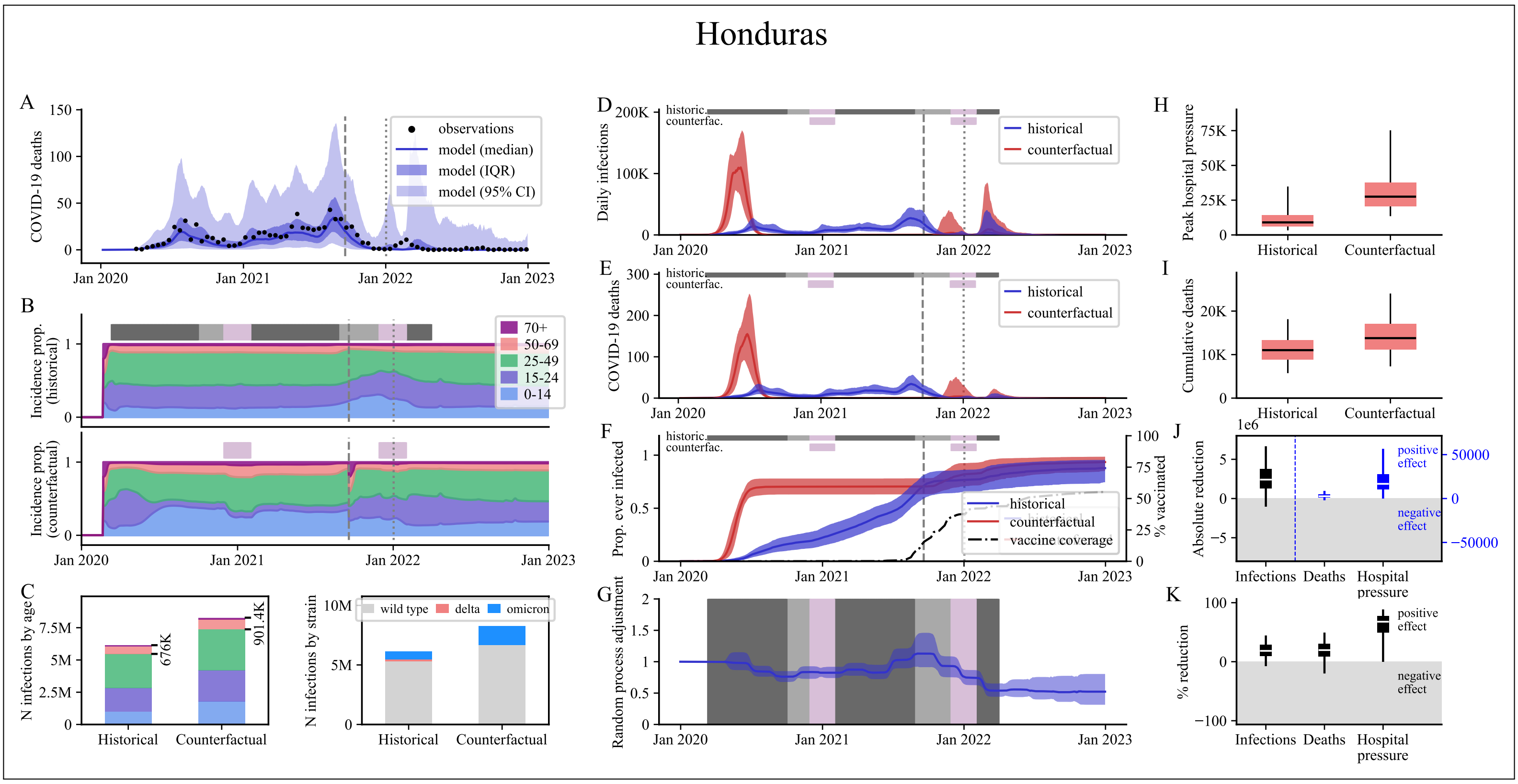
Honduras
A
COVID-19 deaths
observations
model (median)
model (IQR)
model (95% CI)
B
Incidence prop. (historical)
Incidence prop. (counterfactual)
70+
50-69
25-49
15-24
0-14
C
N infections by age
676K
901.4K
N infections by strain
wild type
delta
omicron
Historical
Counterfactual
D
Daily infections
historic.
counterfac.
historical
counterfactual
E
COVID-19 deaths
F
Prop. ever infected
% vaccinated
vaccine coverage
G
Random process adjustment
H
Peak hospital pressure
I
Cumulative deaths
J
Absolute reduction
1e6
positive effect
negative effect
Infections
Deaths
Hospital pressure
K
% reduction
Jan 2020
Jan 2021
Jan 2022
Jan 2023

Croatia

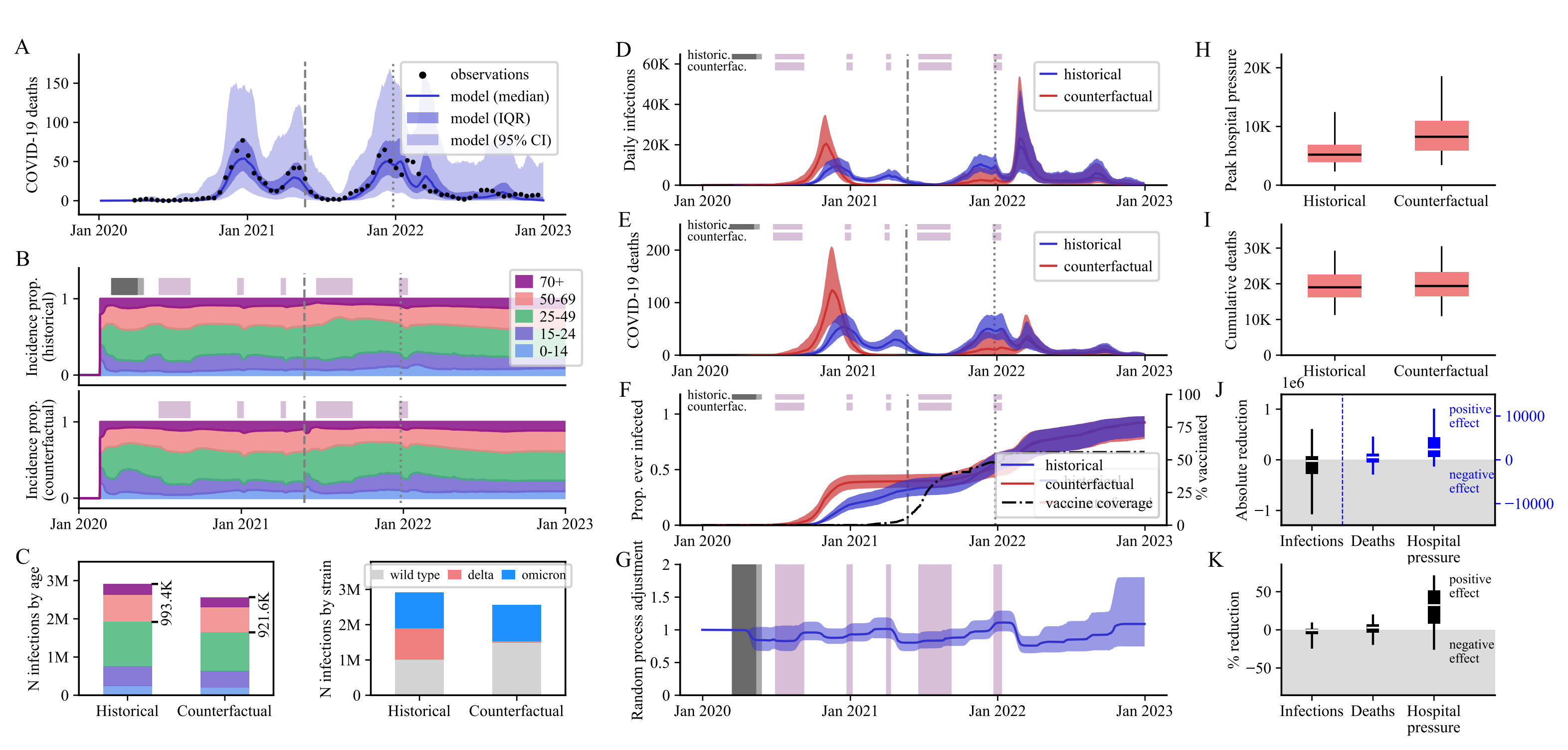

Hungary

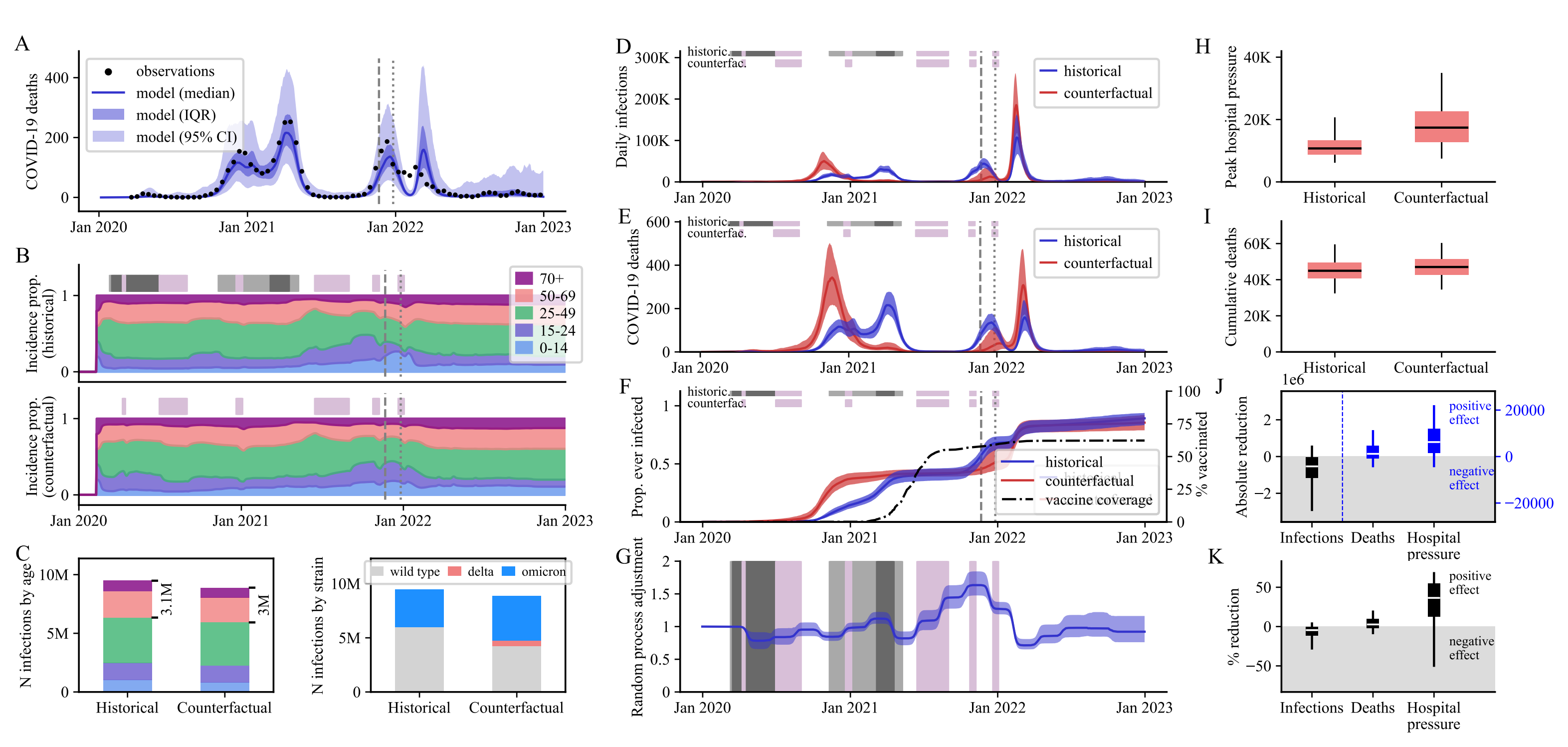

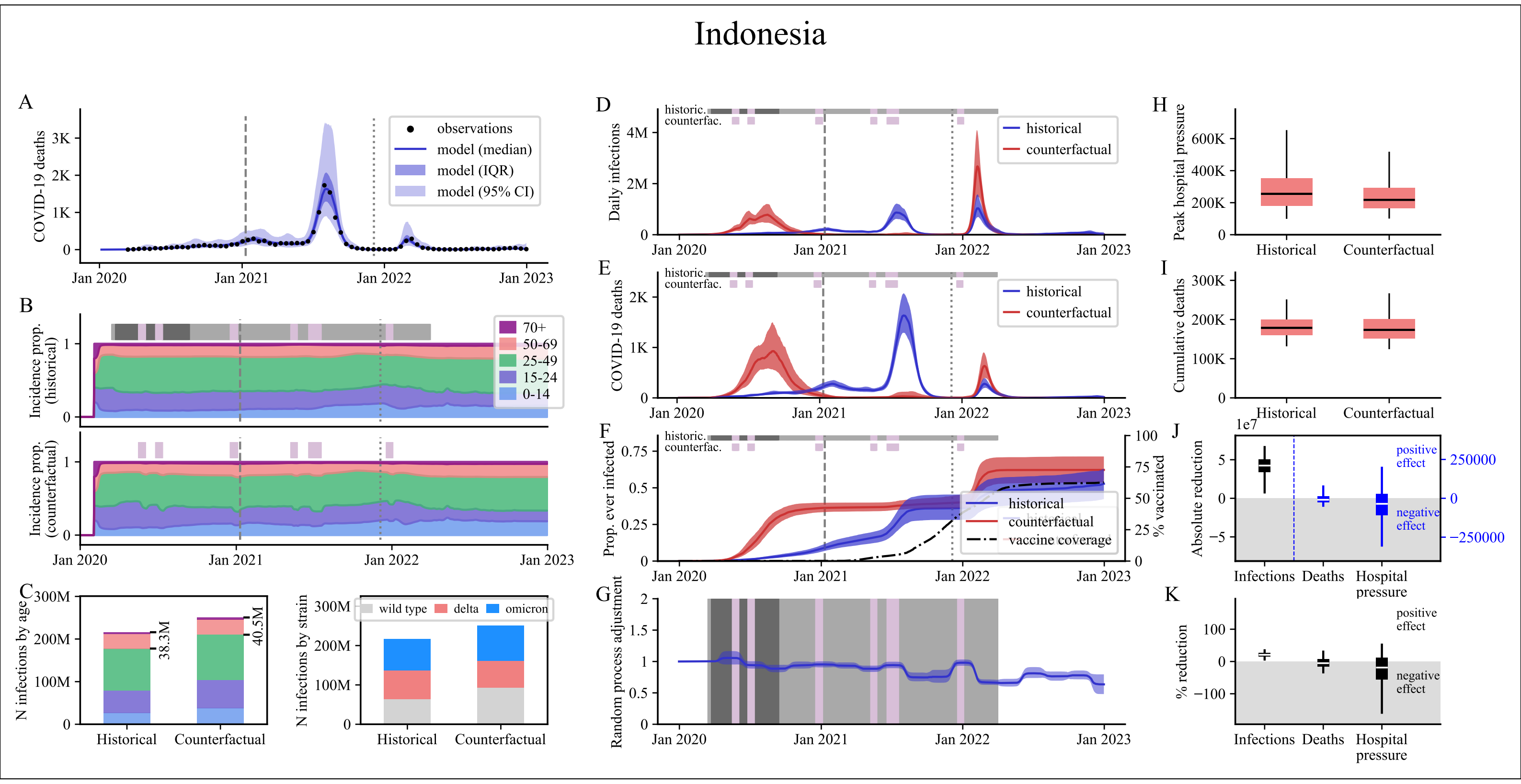
Indonesia
A
COVID-19 deaths
observations
model (median)
model (IQR)
model (95% CI)
B
Incidence prop. (historical)
Incidence prop. (counterfactual)
70+
50-69
25-49
15-24
0-14
C
N infections by age
38.3M
40.5M
N infections by strain
wild type
delta
omicron
Historical
Counterfactual
D
Daily infections
historical
counterfactual
E
COVID-19 deaths
F
Prop. ever infected
% vaccinated
vaccine coverage
G
Random process adjustment
H
Peak hospital pressure
I
Cumulative deaths
J
Absolute reduction
positive effect
negative effect
K
% reduction
Infections
Deaths
Hospital pressure

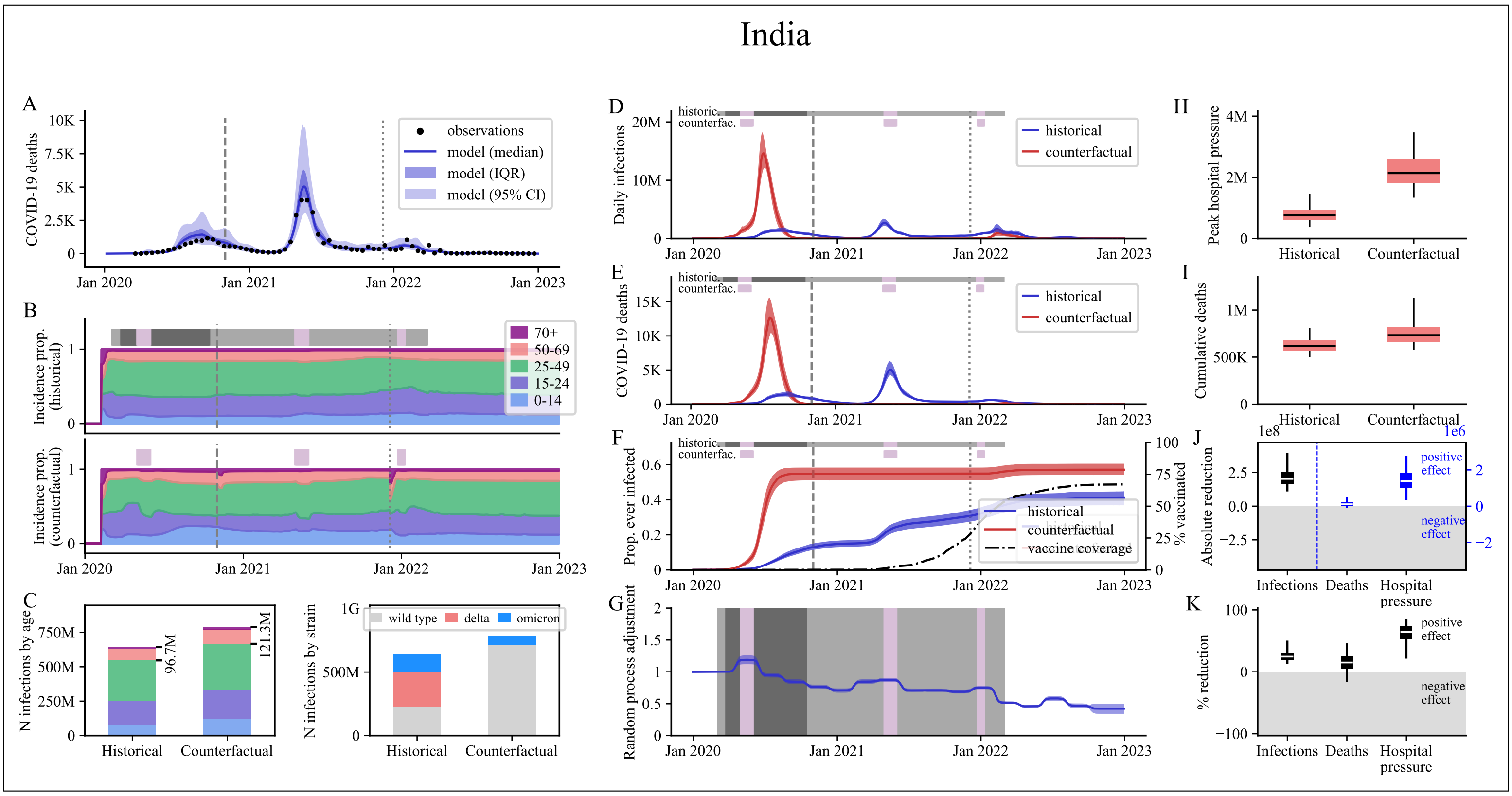
India
A
COVID-19 deaths
observations
model (median)
model (IQR)
model (95% CI)
B
Incidence prop. (historical)
Incidence prop. (counterfactual)
70+
50-69
25-49
15-24
0-14
C
N infections by age
96.7M
121.3M
N infections by strain
wild type
delta
omicron
Historical
Counterfactual
D
Daily infections
historical
counterfactual
E
COVID-19 deaths
F
Prop. ever infected
% vaccinated
vaccine coverage
G
Random process adjustment
H
Peak hospital pressure
I
Cumulative deaths
J
Absolute reduction
positive effect
negative effect
K
% reduction
Infections
Deaths
Hospital pressure

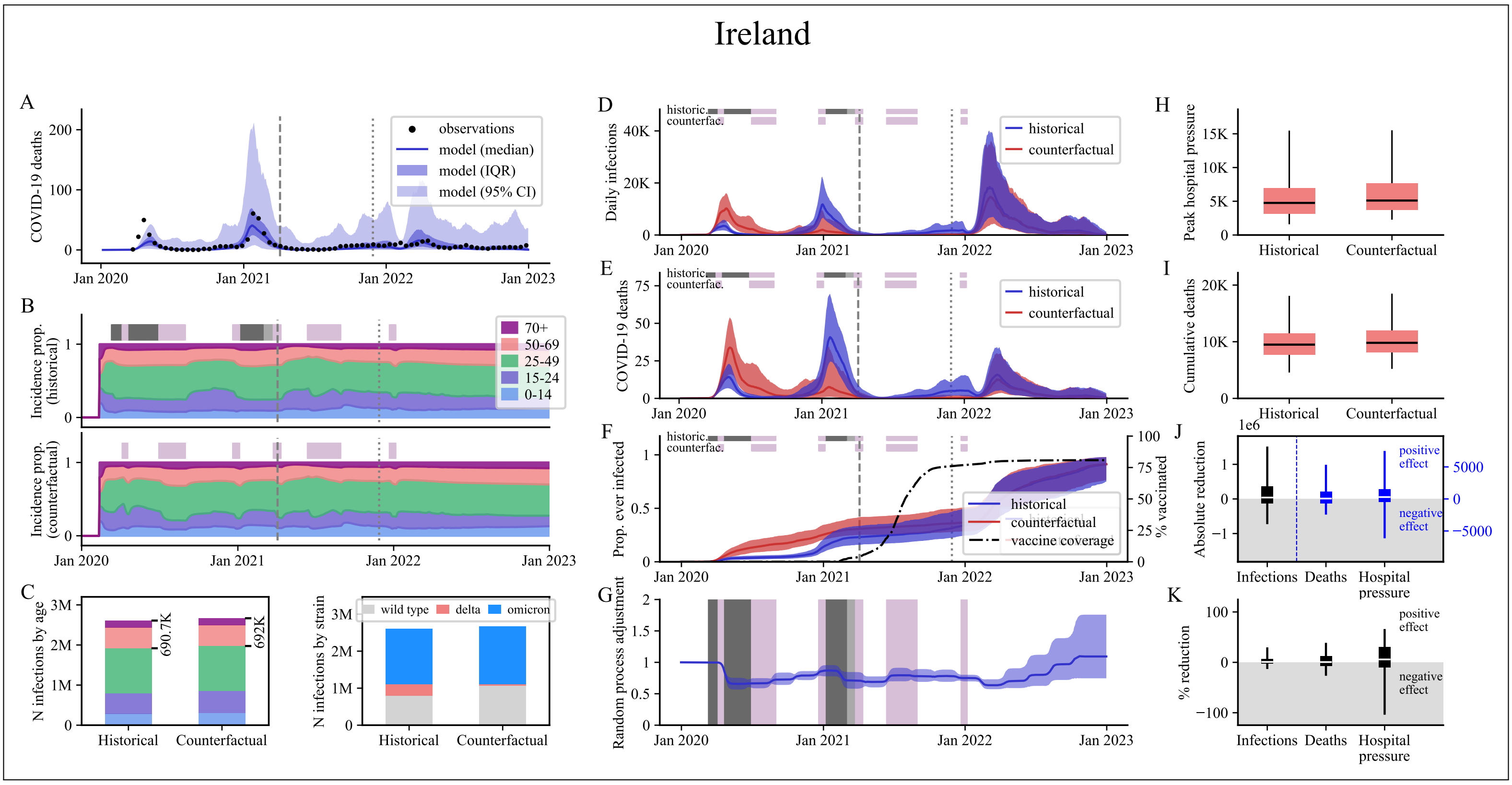
Ireland
A
COVID-19 deaths
observations
model (median)
model (IQR)
model (95% CI)
Jan 2020
Jan 2021
Jan 2022
Jan 2023
B
Incidence prop. (historical)
Incidence prop. (counterfactual)
70+
50-69
25-49
15-24
0-14
C
N infections by age
690.7K
692K
Historical
Counterfactual
N infections by strain
wild type
delta
omicron
D
Daily infections
historic.
counterfac.
historical
counterfactual
E
COVID-19 deaths
F
Prop. ever infected
% vaccinated
vaccine coverage
G
Random process adjustment
H
Peak hospital pressure
I
Cumulative deaths
J
Absolute reduction
1e6
positive effect
negative effect
Infections
Deaths
Hospital pressure
K
% reduction

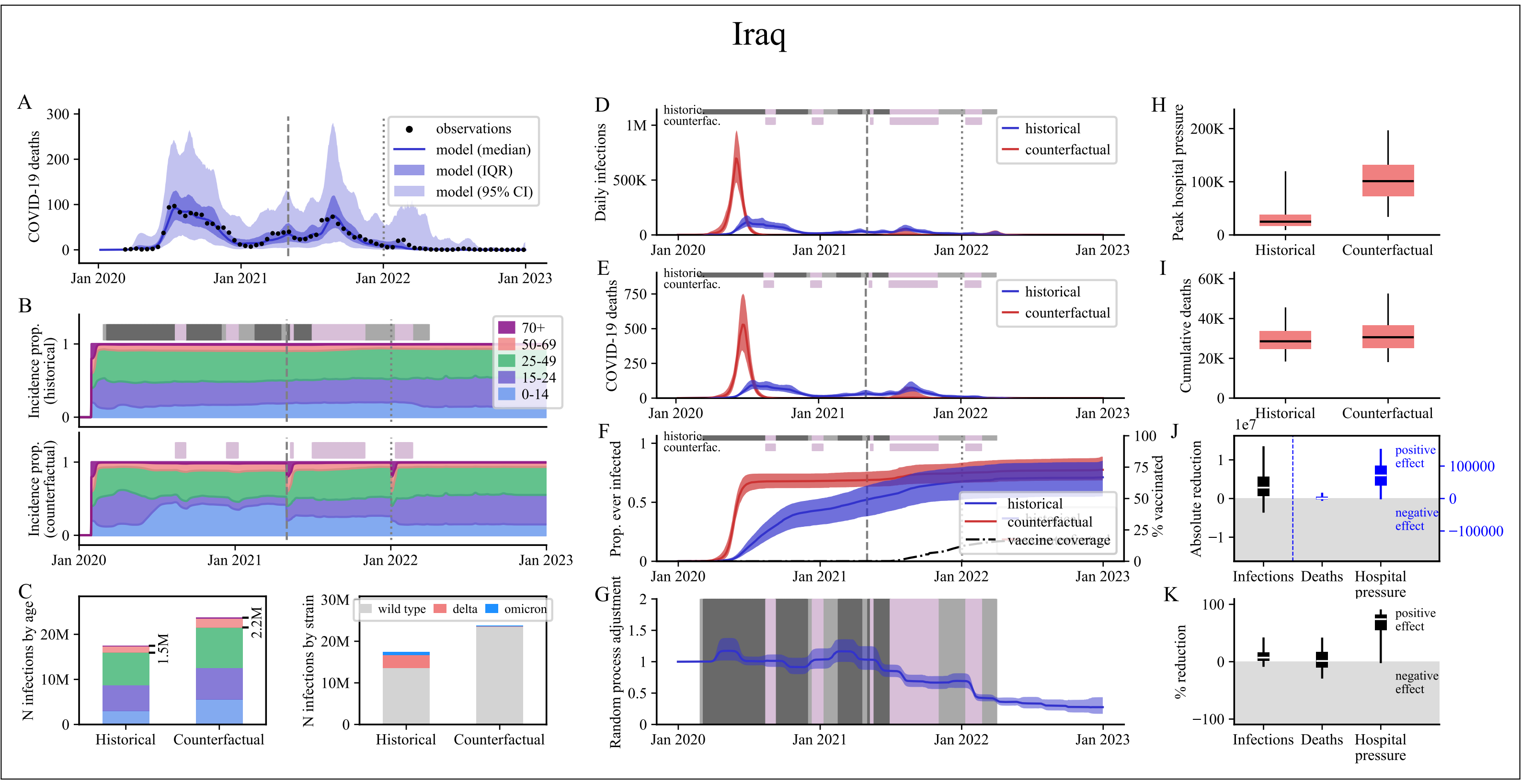
Iraq
A
COVID-19 deaths
observations
model (median)
model (IQR)
model (95% CI)
Jan 2020
Jan 2021
Jan 2022
Jan 2023
B
Incidence prop. (historical)
Incidence prop. (counterfactual)
70+
50-69
25-49
15-24
0-14
C
N infections by age
1.5M
2.2M
Historical
Counterfactual
N infections by strain
wild type
delta
omicron
D
Daily infections
historic.
counterfac.
historical
counterfactual
E
COVID-19 deaths
F
Prop. ever infected
% vaccinated
vaccine coverage
G
Random process adjustment
H
Peak hospital pressure
I
Cumulative deaths
J
Absolute reduction
1e7
positive effect
negative effect
Infections
Deaths
Hospital pressure
K
% reduction

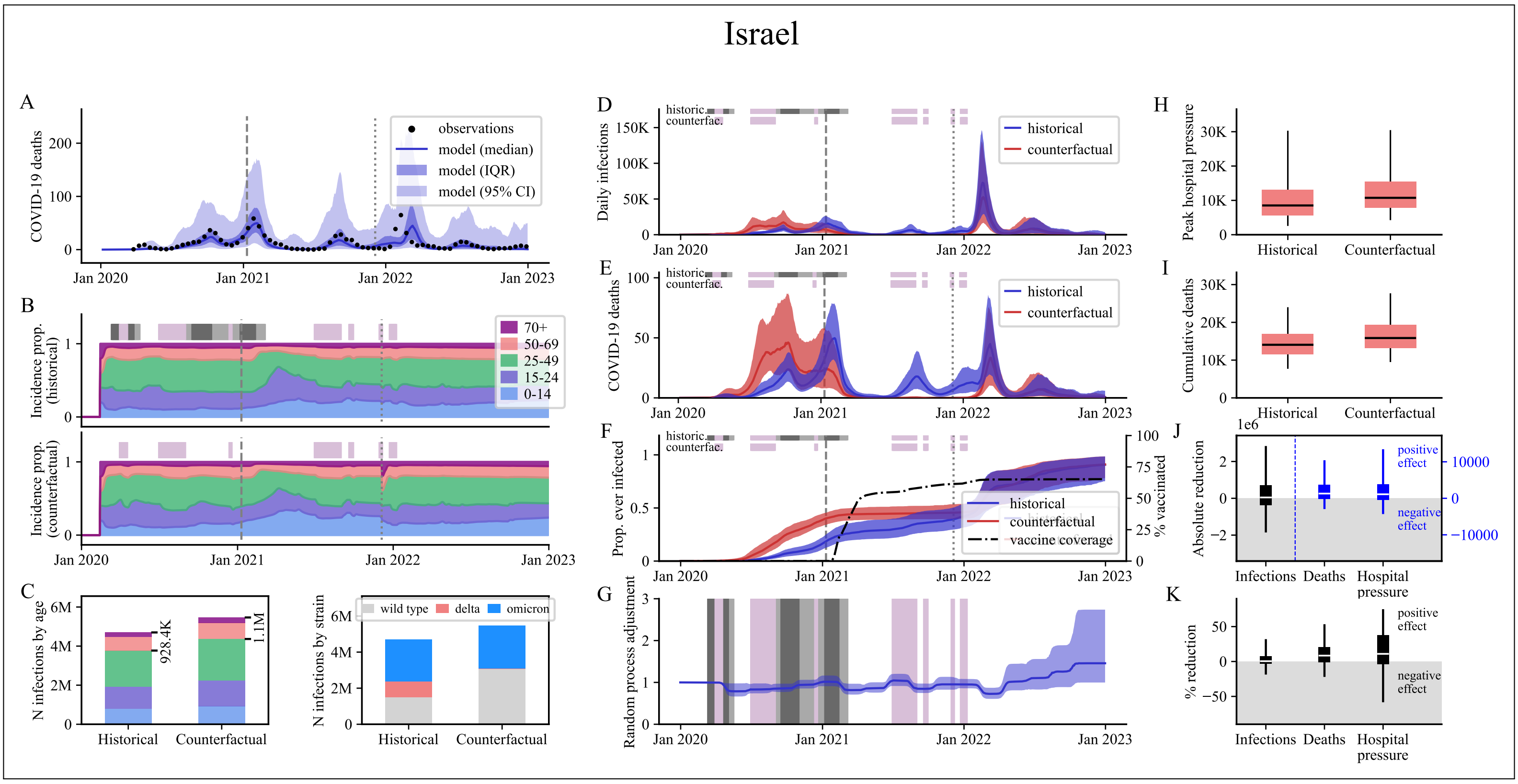
Israel
A
COVID-19 deaths
observations
model (median)
model (IQR)
model (95% CI)
Jan 2020
Jan 2021
Jan 2022
Jan 2023
B
Incidence prop. (historical)
Incidence prop. (counterfactual)
70+
50-69
25-49
15-24
0-14
C
N infections by age
928.4K
1.1M
Historical
Counterfactual
N infections by strain
wild type
delta
omicron
D
Daily infections
historic.
counterfac.
historical
counterfactual
E
COVID-19 deaths
F
Prop. ever infected
% vaccinated
vaccine coverage
G
Random process adjustment
H
Peak hospital pressure
I
Cumulative deaths
J
Absolute reduction
1e6
positive effect
negative effect
Infections
Deaths
Hospital pressure
K
% reduction

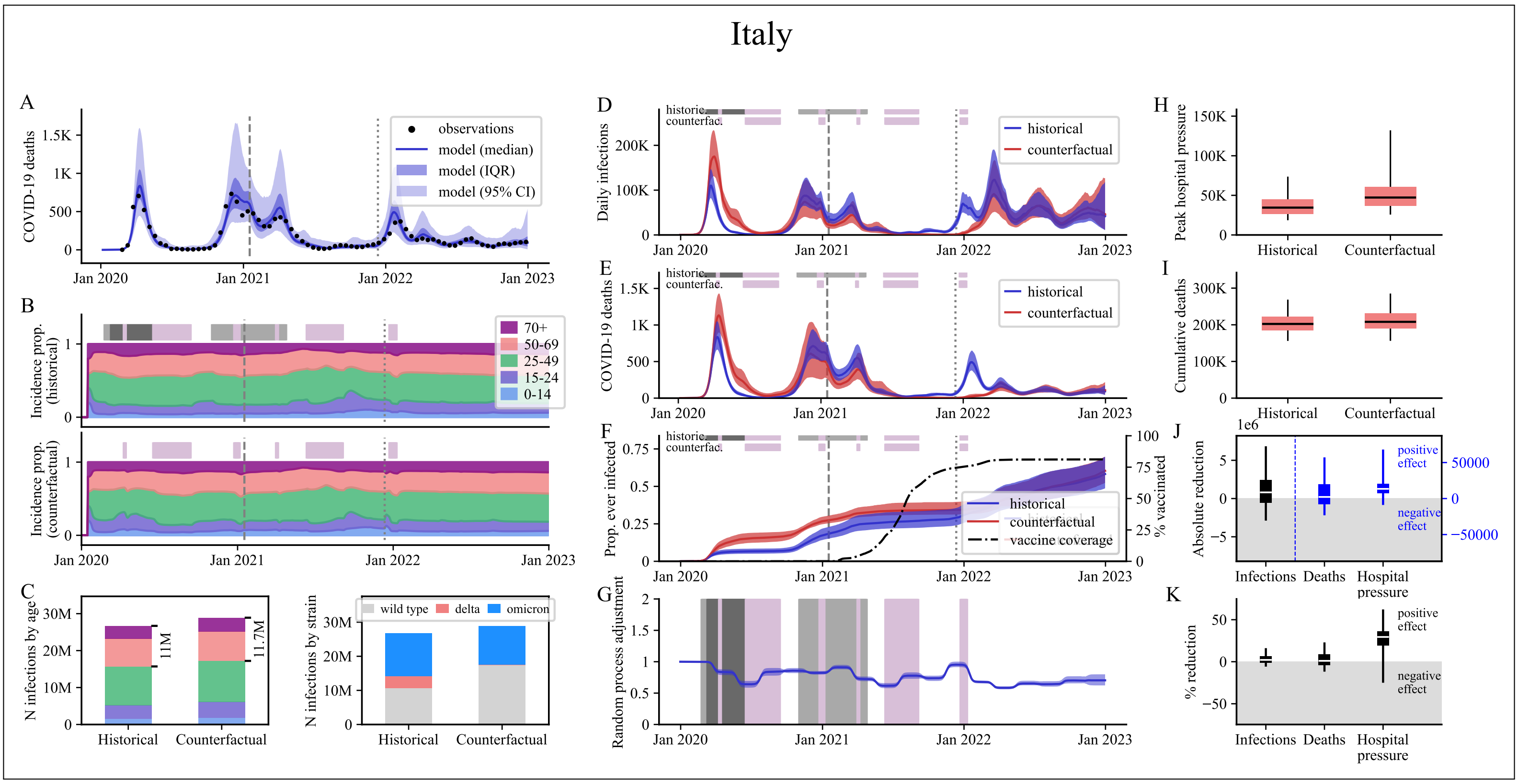
Italy
A
COVID-19 deaths
observations
model (median)
model (IQR)
model (95% CI)
Jan 2020
Jan 2021
Jan 2022
Jan 2023
B
Incidence prop. (historical)
Incidence prop. (counterfactual)
70+
50-69
25-49
15-24
0-14
C
N infections by age
11M
11.7M
Historical
Counterfactual
N infections by strain
wild type
delta
omicron
D
Daily infections
historic.
counterfac.
historical
counterfactual
E
COVID-19 deaths
F
Prop. ever infected
% vaccinated
vaccine coverage
G
Random process adjustment
H
Peak hospital pressure
I
Cumulative deaths
J
Absolute reduction
1e6
positive effect
negative effect
Infections
Deaths
Hospital pressure
K
% reduction

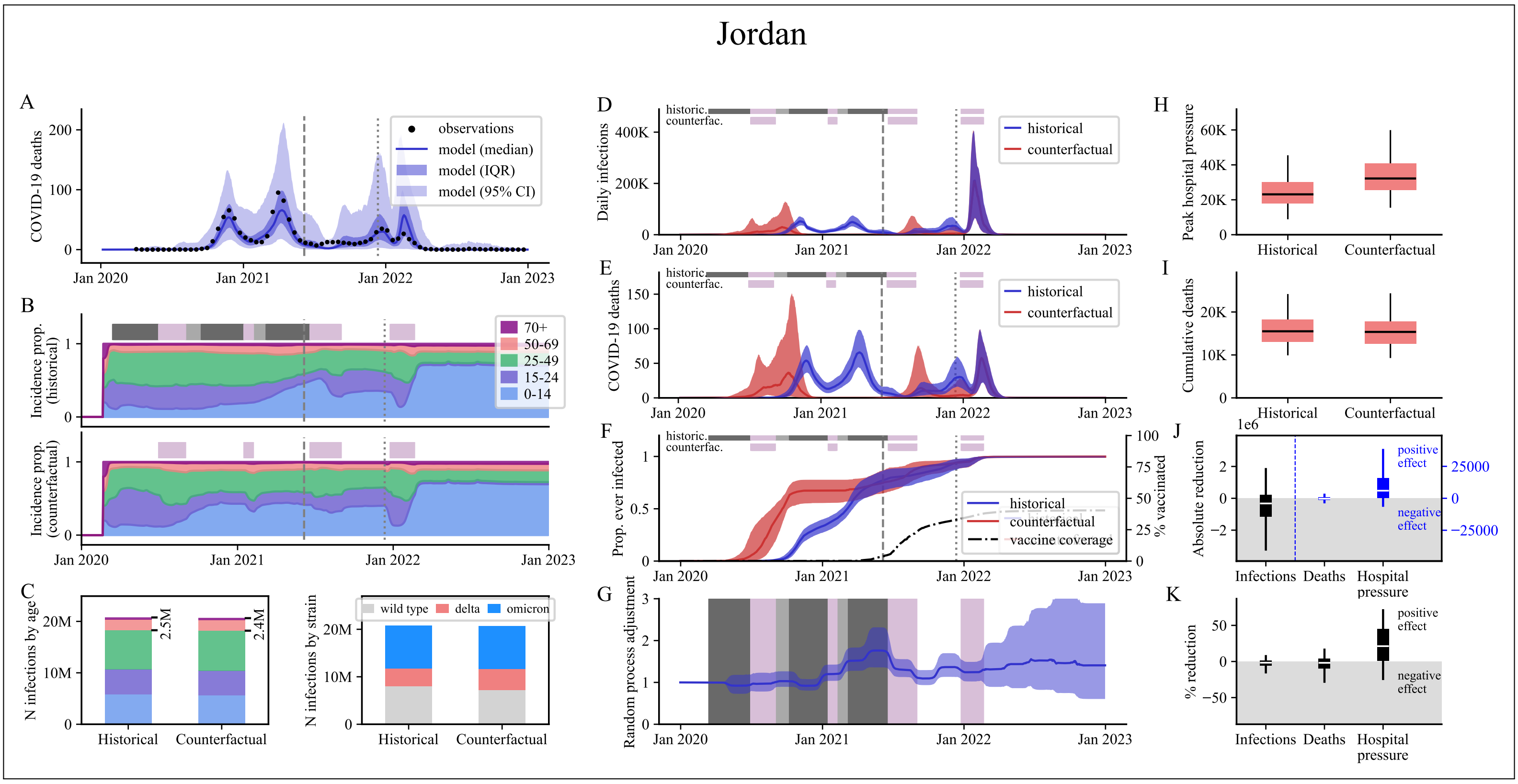
Jordan
A
COVID-19 deaths
observations
model (median)
model (IQR)
model (95% CI)
B
Incidence prop. (historical)
Incidence prop. (counterfactual)
70+
50-69
25-49
15-24
0-14
C
N infections by age
2.5M
2.4M
Historical
Counterfactual
N infections by strain
wild type
delta
omicron
D
Daily infections
historic.
counterfac.
historical
counterfactual
E
COVID-19 deaths
F
Prop. ever infected
% vaccinated
vaccine coverage
G
Random process adjustment
H
Peak hospital pressure
I
Cumulative deaths
J
Absolute reduction
1e6
positive effect
negative effect
Infections
Deaths
Hospital pressure
K
% reduction
Jan 2020
Jan 2021
Jan 2022
Jan 2023

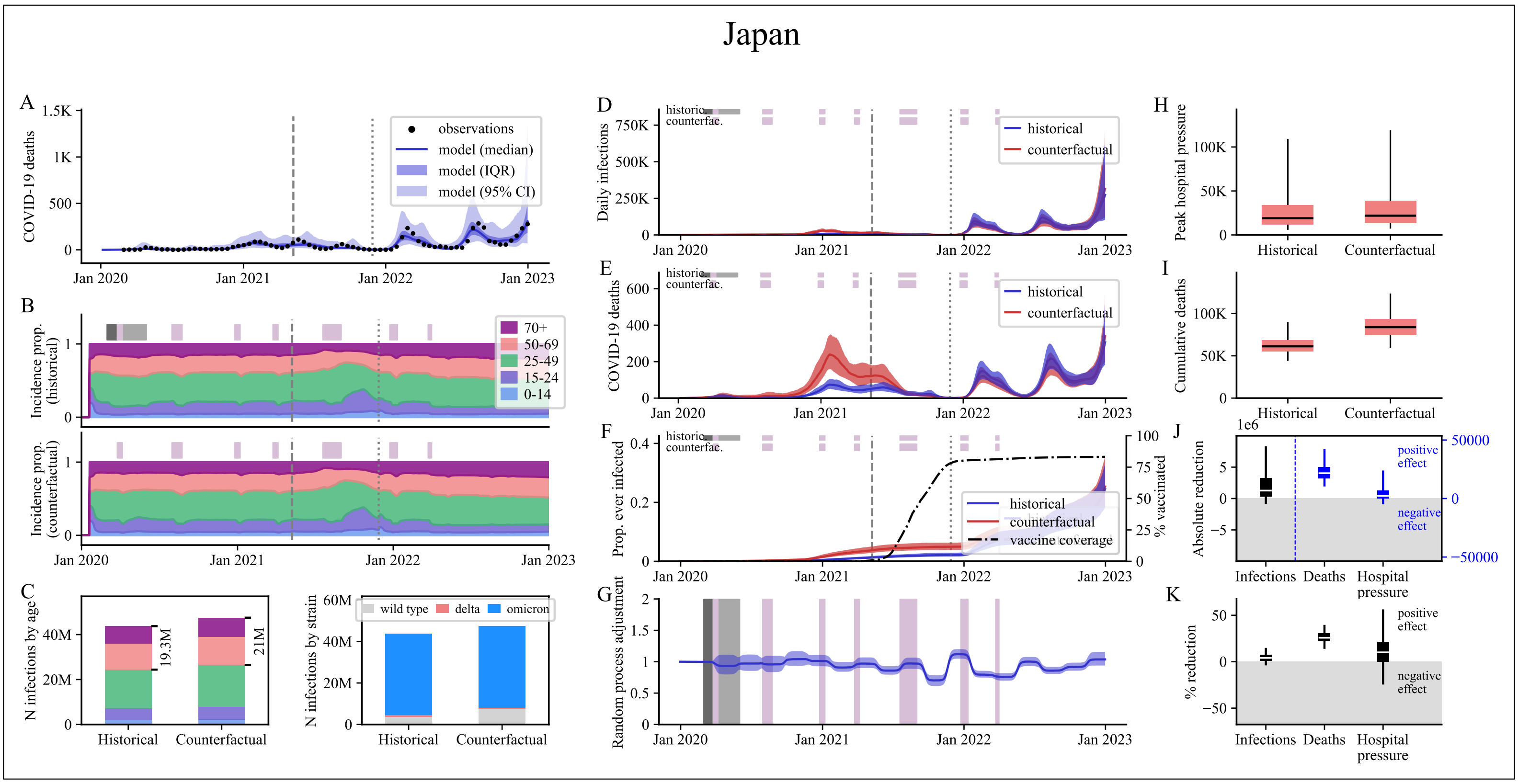
Japan
A
COVID-19 deaths
observations
model (median)
model (IQR)
model (95% CI)
B
Incidence prop. (historical)
Incidence prop. (counterfactual)
70+
50-69
25-49
15-24
0-14
C
N infections by age
19.3M
21M
Historical
Counterfactual
N infections by strain
wild type
delta
omicron
D
Daily infections
historic.
counterfac.
historical
counterfactual
E
COVID-19 deaths
F
Prop. ever infected
% vaccinated
vaccine coverage
G
Random process adjustment
H
Peak hospital pressure
I
Cumulative deaths
J
Absolute reduction
1e6
positive effect
negative effect
Infections
Deaths
Hospital pressure
K
% reduction
Jan 2020
Jan 2021
Jan 2022
Jan 2023

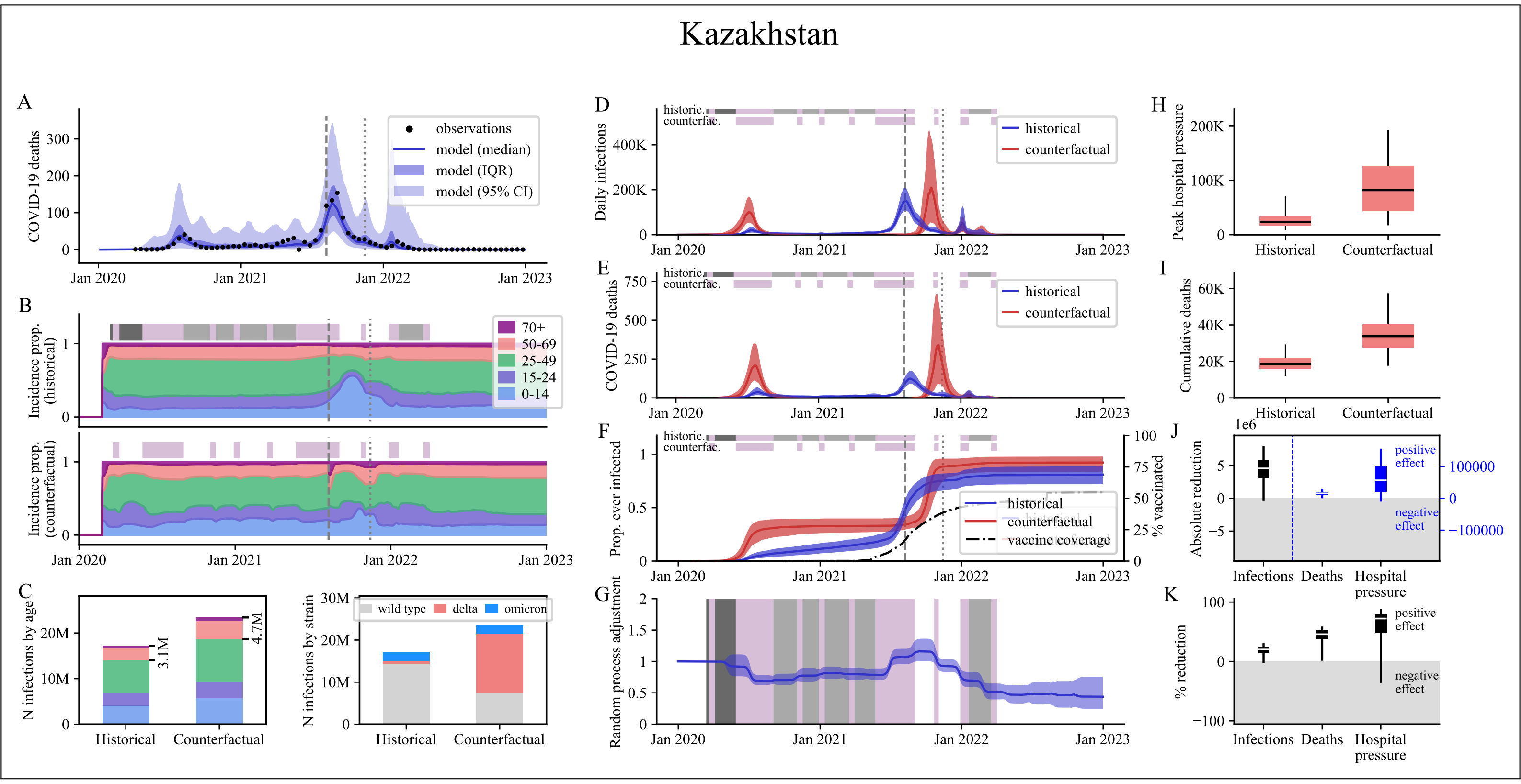
Kazakhstan
A
COVID-19 deaths
observations
model (median)
model (IQR)
model (95% CI)
B
Incidence prop. (historical)
Incidence prop. (counterfactual)
70+
50-69
25-49
15-24
0-14
C
N infections by age
3.1M
4.7M
N infections by strain
wild type
delta
omicron
Historical
Counterfactual
D
Daily infections
historic.
counterfac.
historical
counterfactual
E
COVID-19 deaths
F
Prop. ever infected
% vaccinated
vaccine coverage
G
Random process adjustment
H
Peak hospital pressure
I
Cumulative deaths
J
Absolute reduction
1e6
positive effect
negative effect
K
% reduction
Infections
Deaths
Hospital pressure
Jan 2020
Jan 2021
Jan 2022
Jan 2023

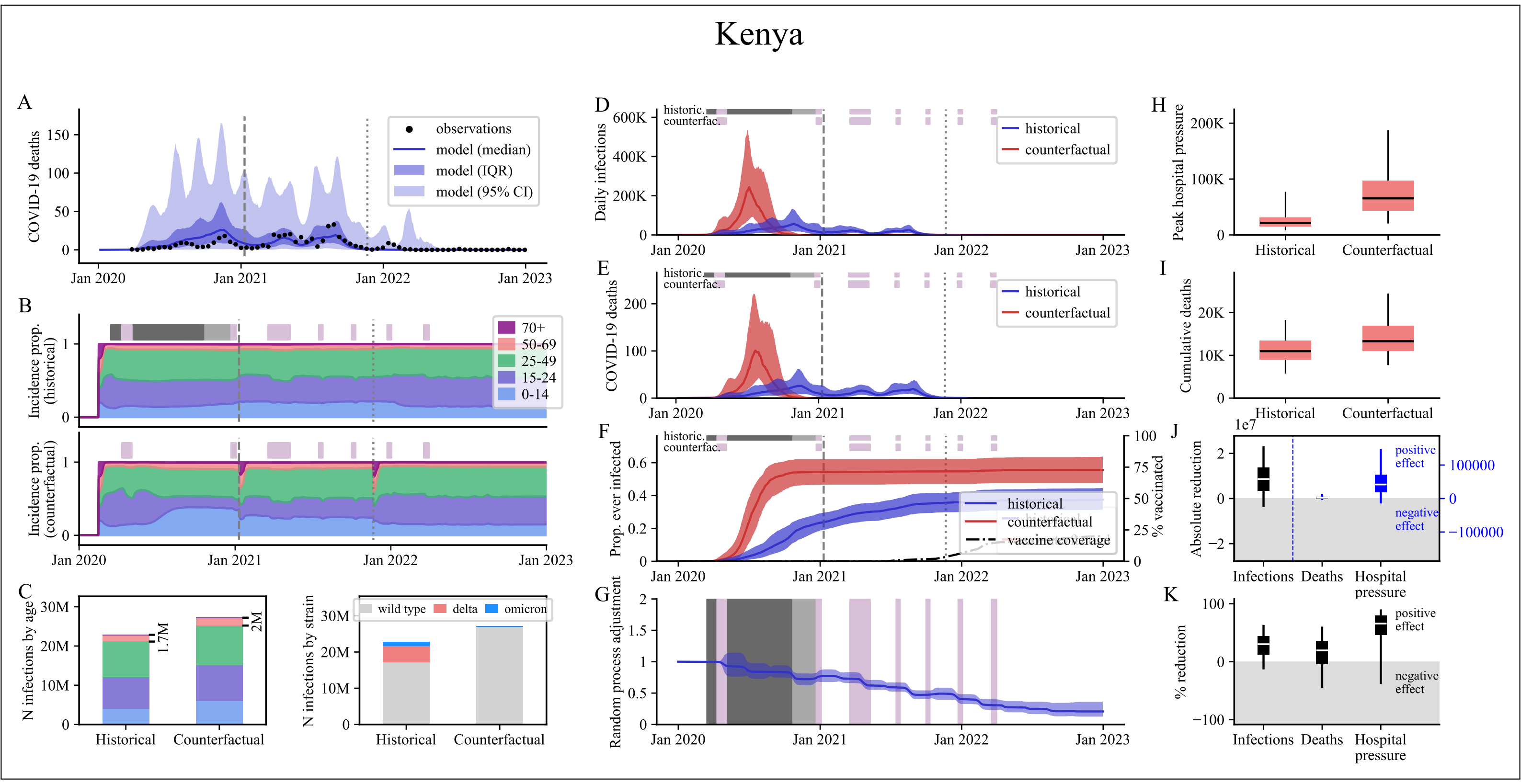
Kenya
A
COVID-19 deaths
observations
model (median)
model (IQR)
model (95% CI)
B
Incidence prop. (historical)
Incidence prop. (counterfactual)
70+
50-69
25-49
15-24
0-14
C
N infections by age
1.7M
2M
N infections by strain
wild type
delta
omicron
Historical
Counterfactual
D
Daily infections
historic.
counterfac.
historical
counterfactual
E
COVID-19 deaths
F
Prop. ever infected
% vaccinated
vaccine coverage
G
Random process adjustment
H
Peak hospital pressure
I
Cumulative deaths
J
Absolute reduction
1e7
positive effect
negative effect
K
% reduction
Infections
Deaths
Hospital pressure
Jan 2020
Jan 2021
Jan 2022
Jan 2023

Korea Republic of

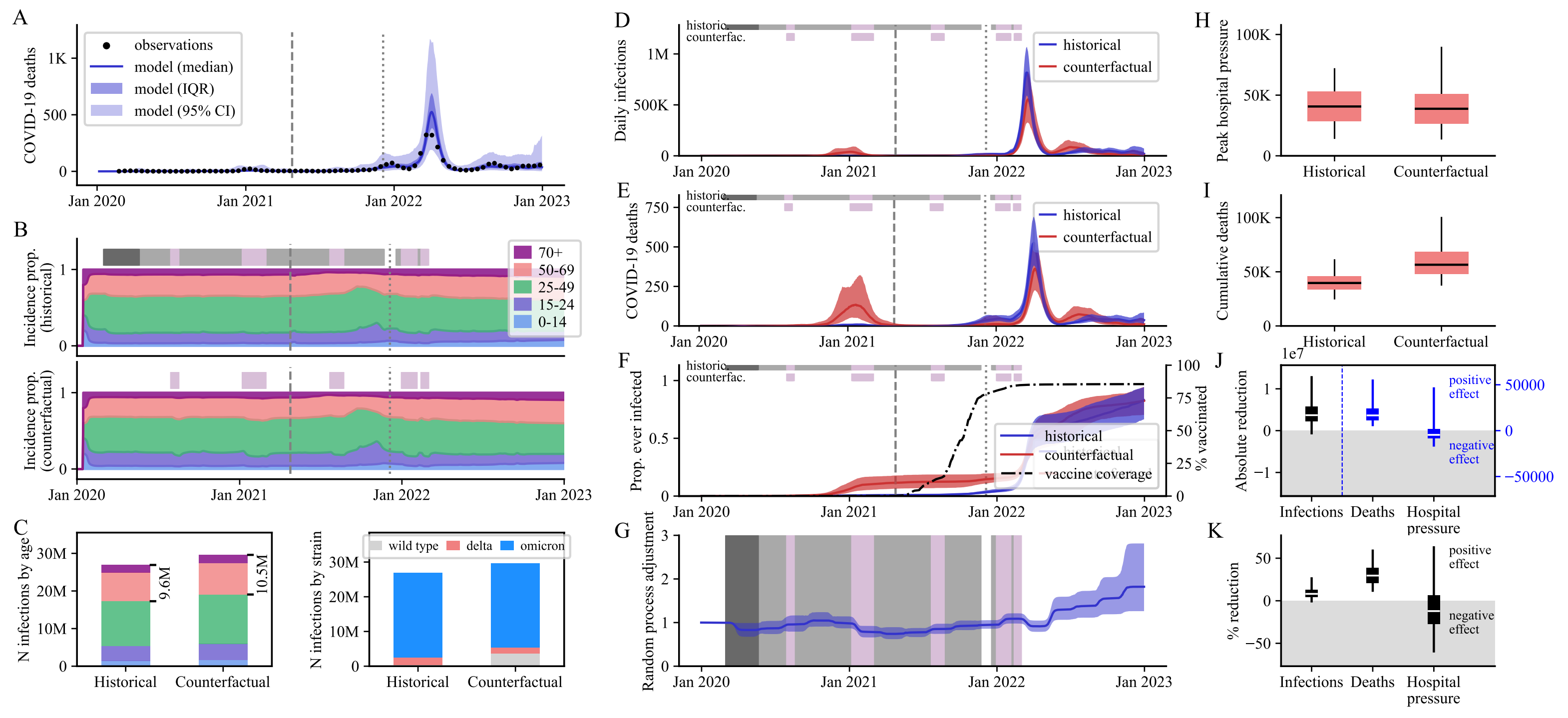

Lebanon

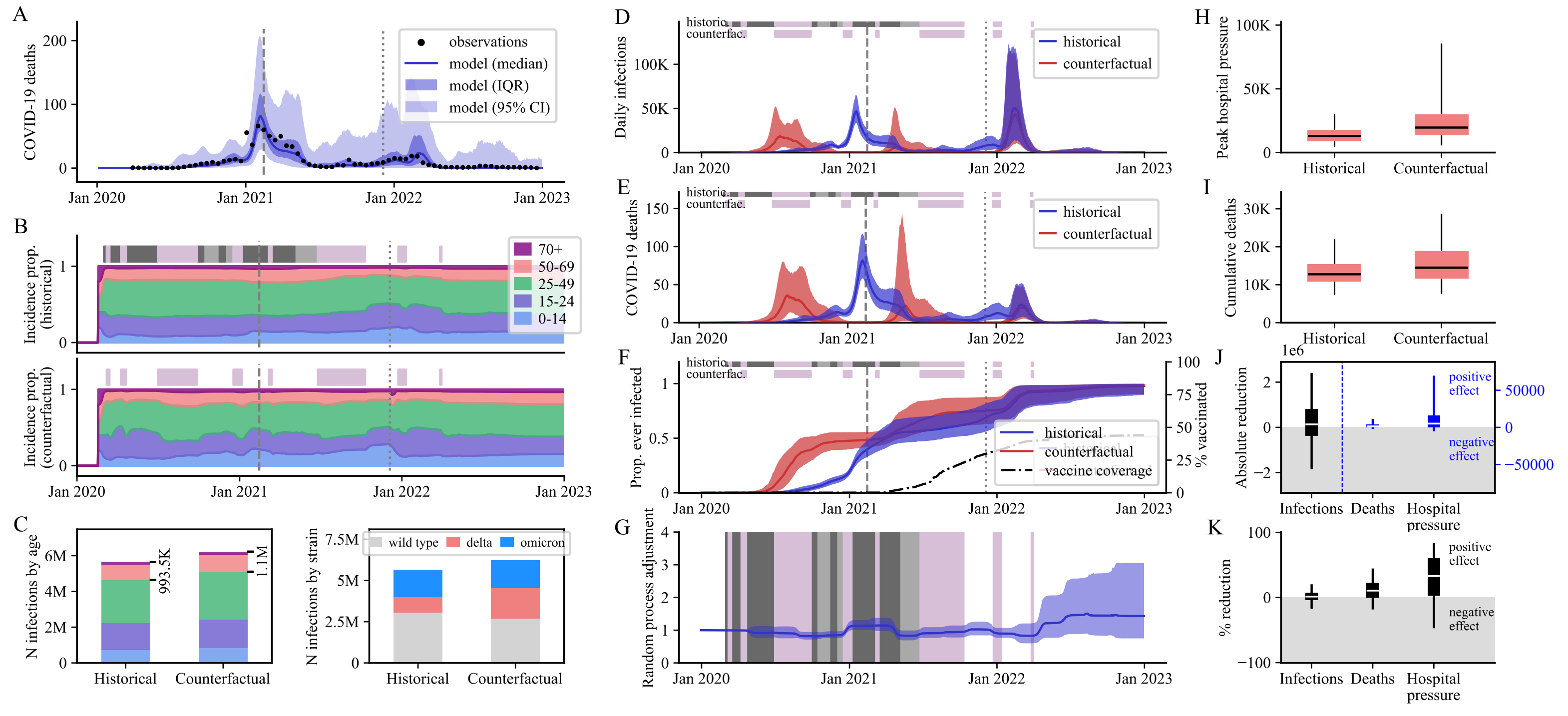

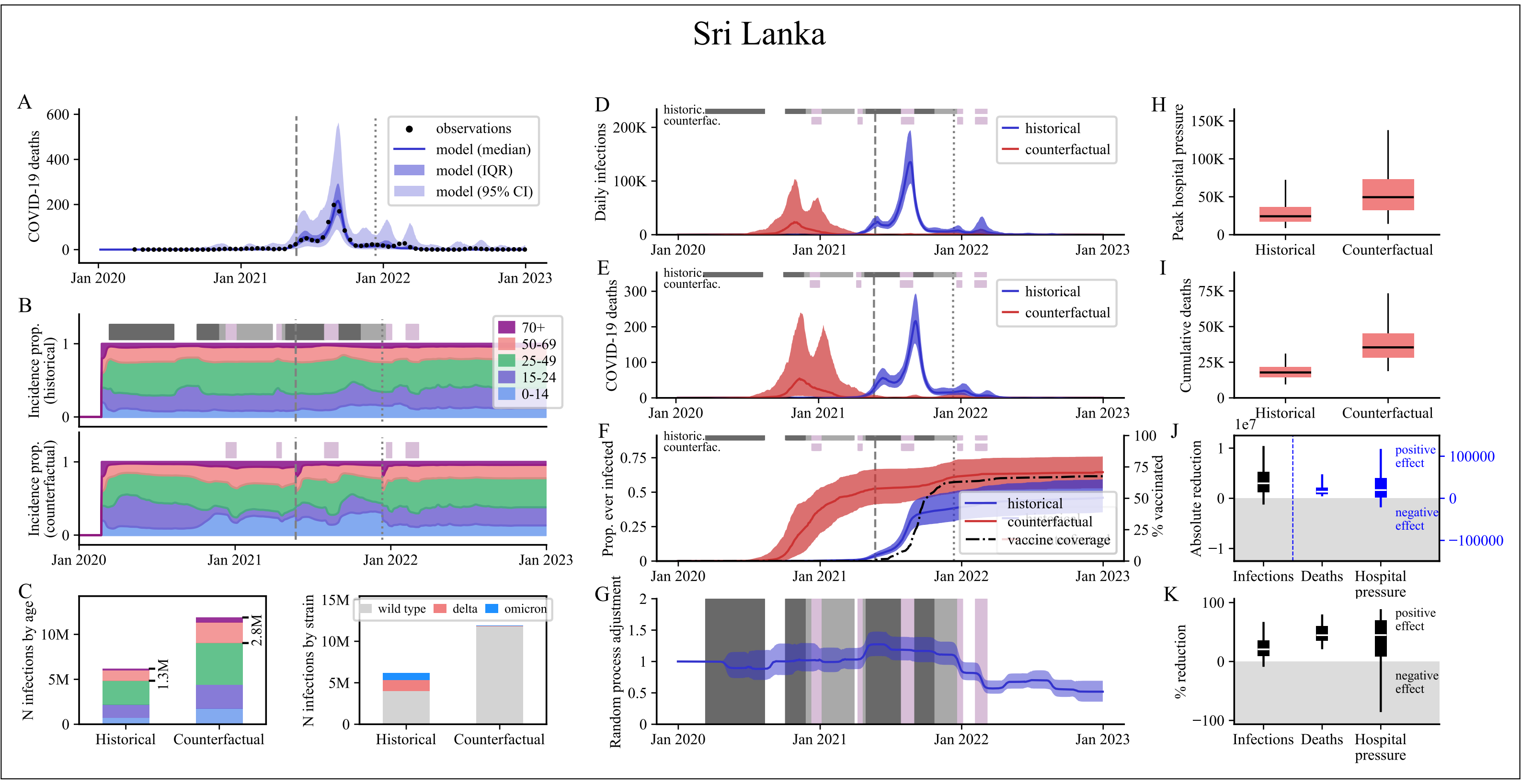

Sri Lanka
A
COVID-19 deaths
observations
model (median)
model (IQR)
model (95% CI)
B
Incidence prop. (historical)
Incidence prop. (counterfactual)
70+
50-69
25-49
15-24
0-14
C
N infections by age
1.3M
2.8M
N infections by strain
wild type
delta
omicron
Historical
Counterfactual
D
historic.
counterfac.
Daily infections
historical
counterfactual
E
COVID-19 deaths
F
Prop. ever infected
% vaccinated
vaccine coverage
G
Random process adjustment
Jan 2020
Jan 2021
Jan 2022
Jan 2023
H
Peak hospital pressure
I
Cumulative deaths
J
Absolute reduction
1e7
positive effect
negative effect
K
% reduction
Infections
Deaths
Hospital pressure

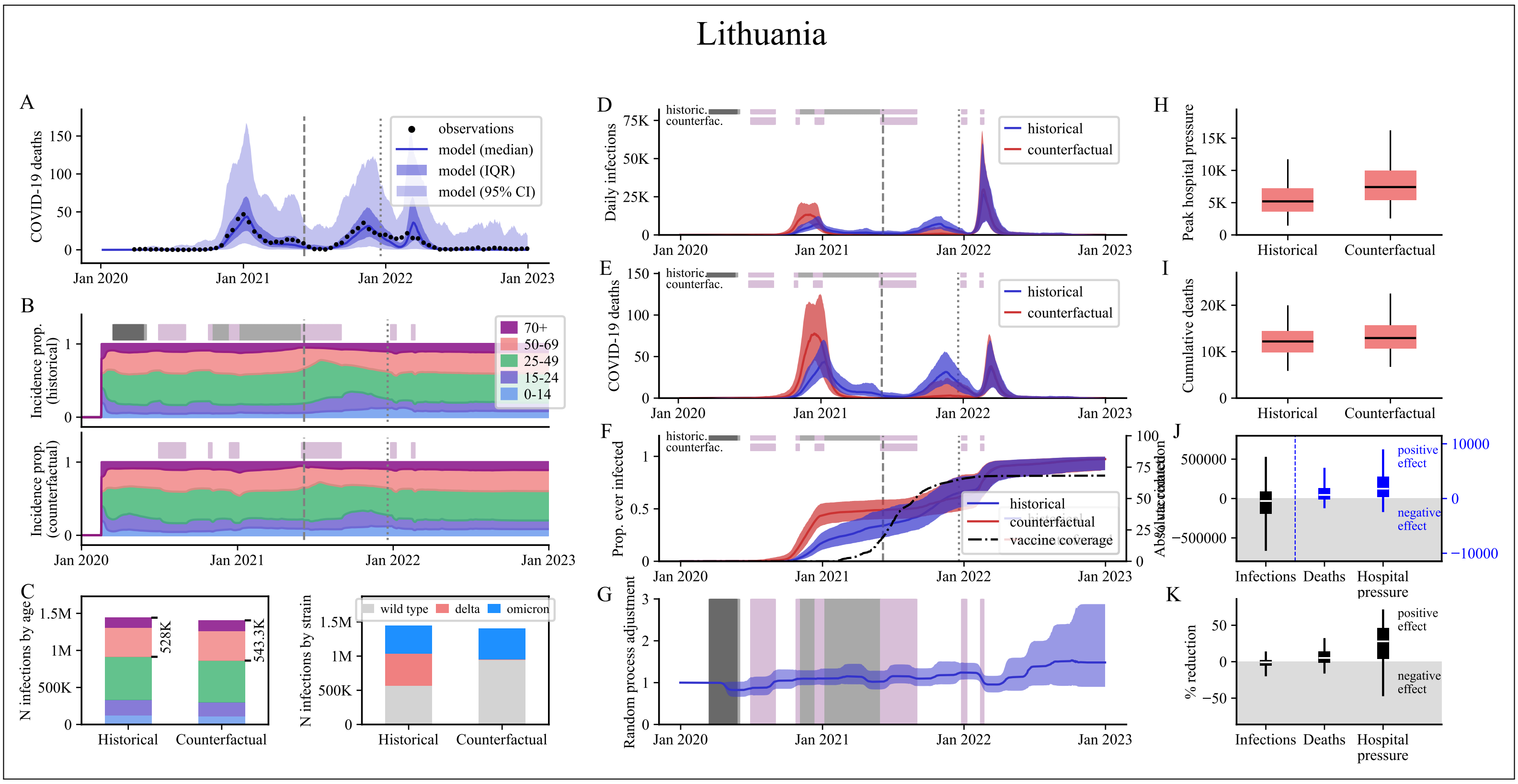

Lithuania
A
COVID-19 deaths
observations
model (median)
model (IQR)
model (95% CI)
B
Incidence prop. (historical)
Incidence prop. (counterfactual)
70+
50-69
25-49
15-24
0-14
C
N infections by age
528K
543.3K
N infections by strain
wild type
delta
omicron
Historical
Counterfactual
D
historic.
counterfac.
Daily infections
historical
counterfactual
E
COVID-19 deaths
F
Prop. ever infected
% vaccinated
vaccine coverage
G
Random process adjustment
Jan 2020
Jan 2021
Jan 2022
Jan 2023
H
Peak hospital pressure
I
Cumulative deaths
J
positive effect
negative effect
K
% reduction
Infections
Deaths
Hospital pressure

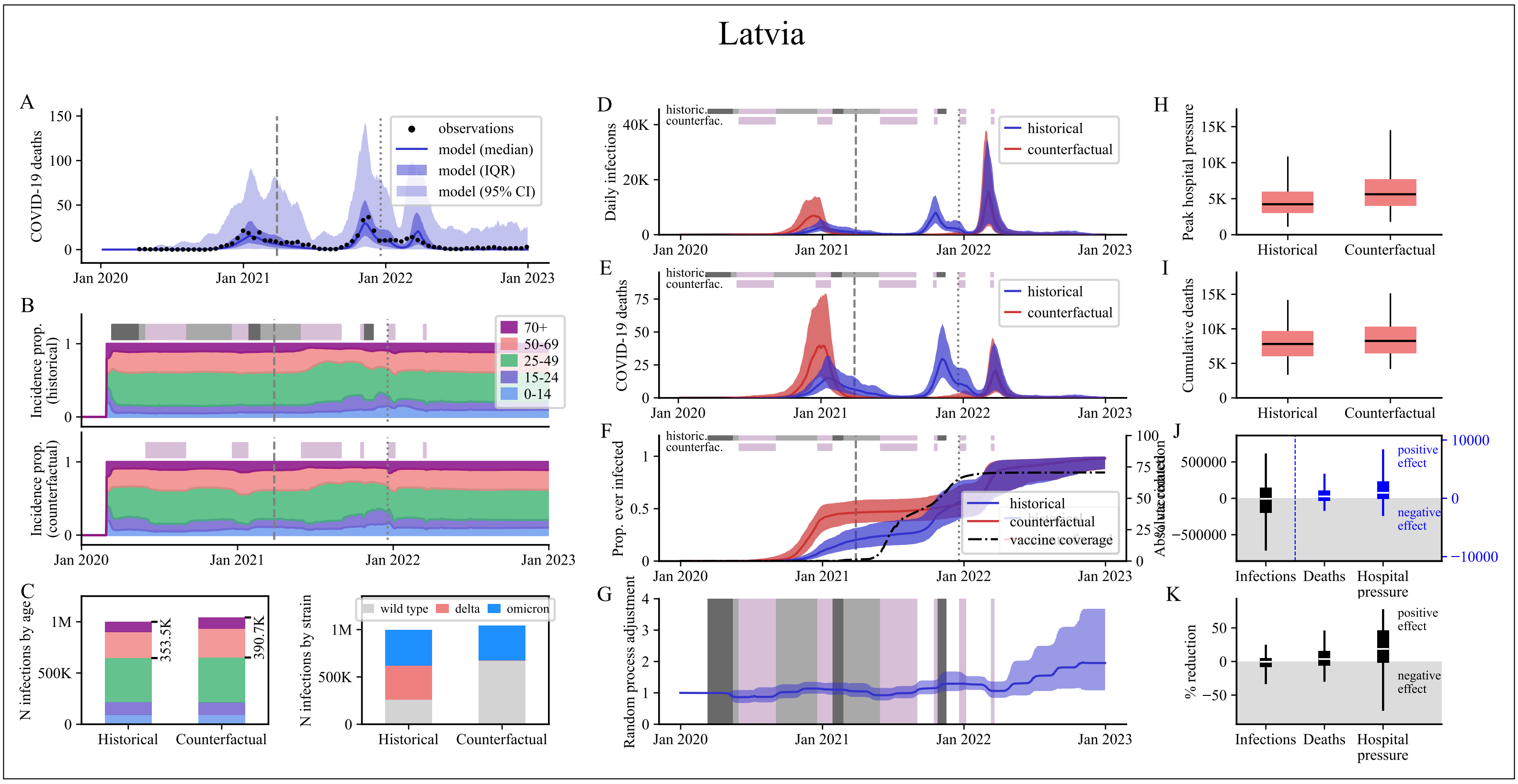
Latvia
A
COVID-19 deaths
observations
model (median)
model (IQR)
model (95% CI)
Jan 2020
Jan 2021
Jan 2022
Jan 2023
B
Incidence prop. (historical)
Incidence prop. (counterfactual)
70+
50-69
25-49
15-24
0-14
C
N infections by age
353.5K
390.7K
Historical
Counterfactual
N infections by strain
wild type
delta
omicron
D
Daily infections
historic.
counterfac.
historical
counterfactual
E
COVID-19 deaths
F
Prop. ever infected
Absolute reduction
vaccine coverage
G
Random process adjustment
H
Peak hospital pressure
I
Cumulative deaths
J
positive effect
negative effect
Infections
Deaths
Hospital pressure
K
% reduction

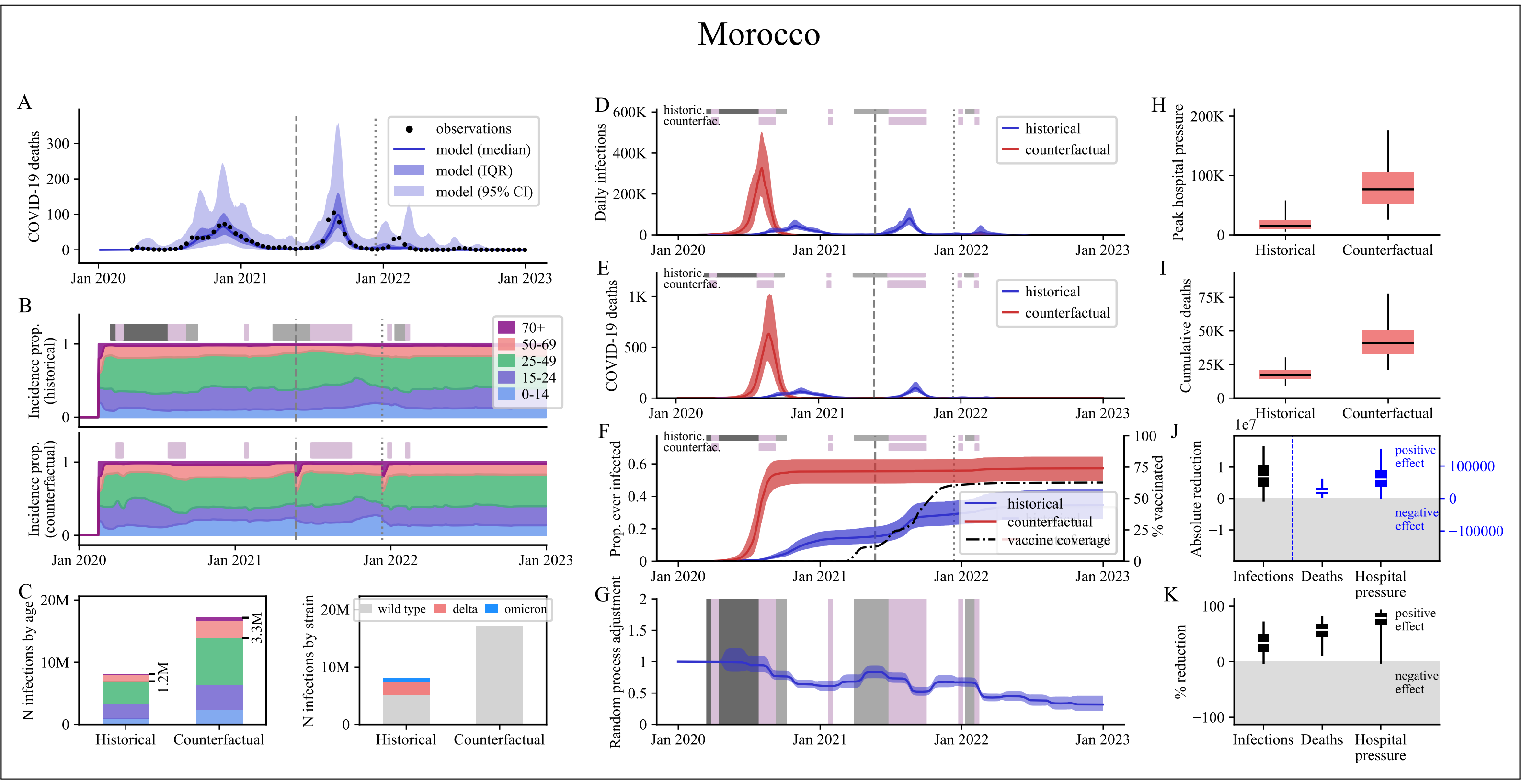
Morocco
A
COVID-19 deaths
observations
model (median)
model (IQR)
model (95% CI)
Jan 2020
Jan 2021
Jan 2022
Jan 2023
B
Incidence prop. (historical)
Incidence prop. (counterfactual)
70+
50-69
25-49
15-24
0-14
C
N infections by age
1.2M
3.3M
Historical
Counterfactual
N infections by strain
wild type
delta
omicron
D
Daily infections
historic.
counterfac.
historical
counterfactual
E
COVID-19 deaths
F
Prop. ever infected
% vaccinated
vaccine coverage
G
Random process adjustment
H
Peak hospital pressure
I
Cumulative deaths
J
Absolute reduction
1e7
positive effect
negative effect
Infections
Deaths
Hospital pressure
K
% reduction

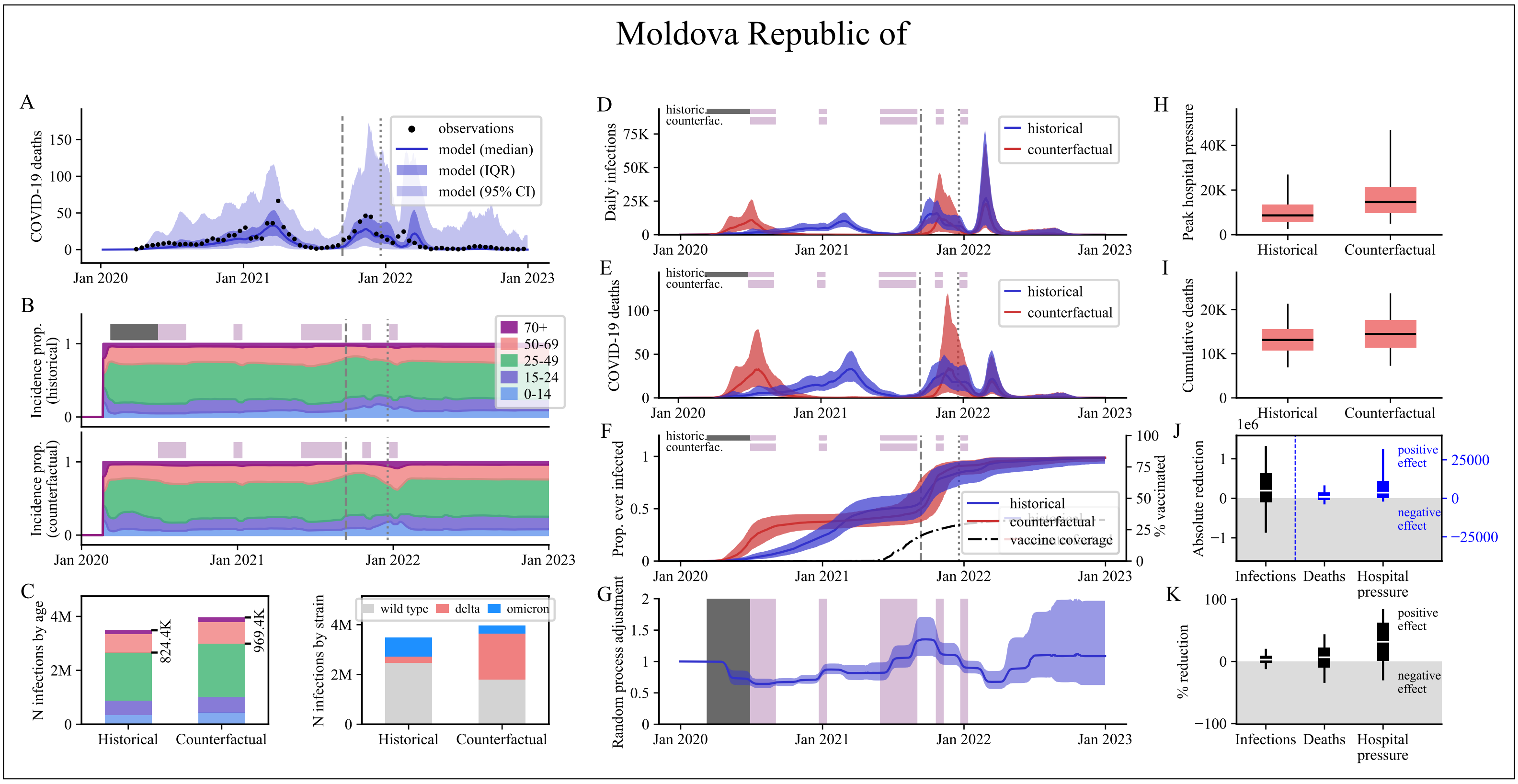
Moldova Republic of
A
COVID-19 deaths
observations
model (median)
model (IQR)
model (95% CI)
Jan 2020
Jan 2021
Jan 2022
Jan 2023
B
Incidence prop. (historical)
Incidence prop. (counterfactual)
70+
50-69
25-49
15-24
0-14
C
N infections by age
824.4K
969.4K
Historical
Counterfactual
N infections by strain
wild type
delta
omicron
D
historic.
counterfac.
Daily infections
historical
counterfactual
E
COVID-19 deaths
F
Prop. ever infected
% vaccinated
vaccine coverage
G
Random process adjustment
H
Peak hospital pressure
I
Cumulative deaths
J
1e6
Absolute reduction
positive effect
negative effect
Infections
Deaths
Hospital pressure
K
% reduction

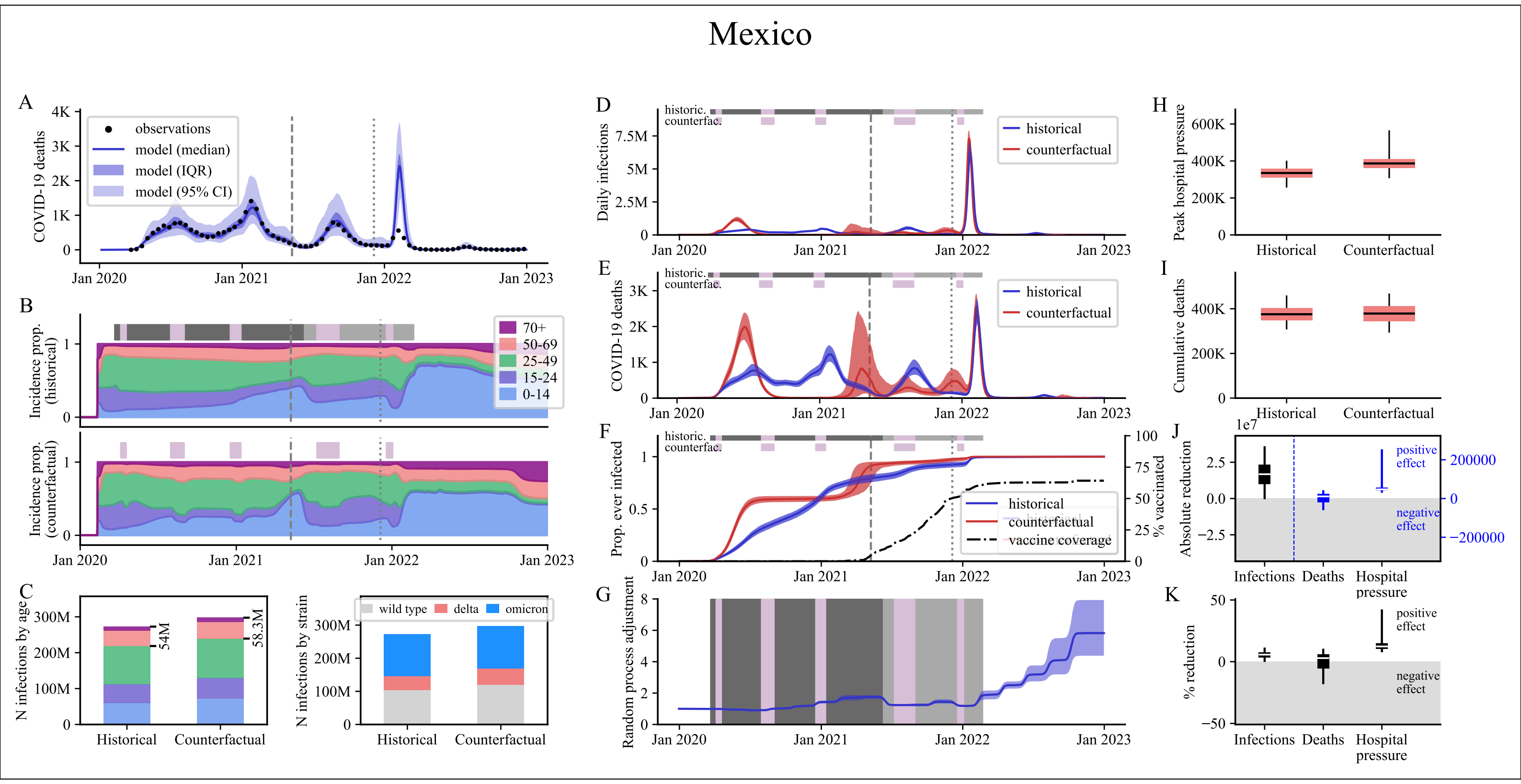
Mexico
A
COVID-19 deaths
observations
model (median)
model (IQR)
model (95% CI)
Jan 2020
Jan 2021
Jan 2022
Jan 2023
B
Incidence prop. (historical)
Incidence prop. (counterfactual)
70+
50-69
25-49
15-24
0-14
C
N infections by age
54M
58.3M
Historical
Counterfactual
N infections by strain
wild type
delta
omicron
D
historic.
counterfac.
Daily infections
historical
counterfactual
E
COVID-19 deaths
F
Prop. ever infected
% vaccinated
vaccine coverage
G
Random process adjustment
H
Peak hospital pressure
I
Cumulative deaths
J
1e7
Absolute reduction
positive effect
negative effect
Infections
Deaths
Hospital pressure
K
% reduction

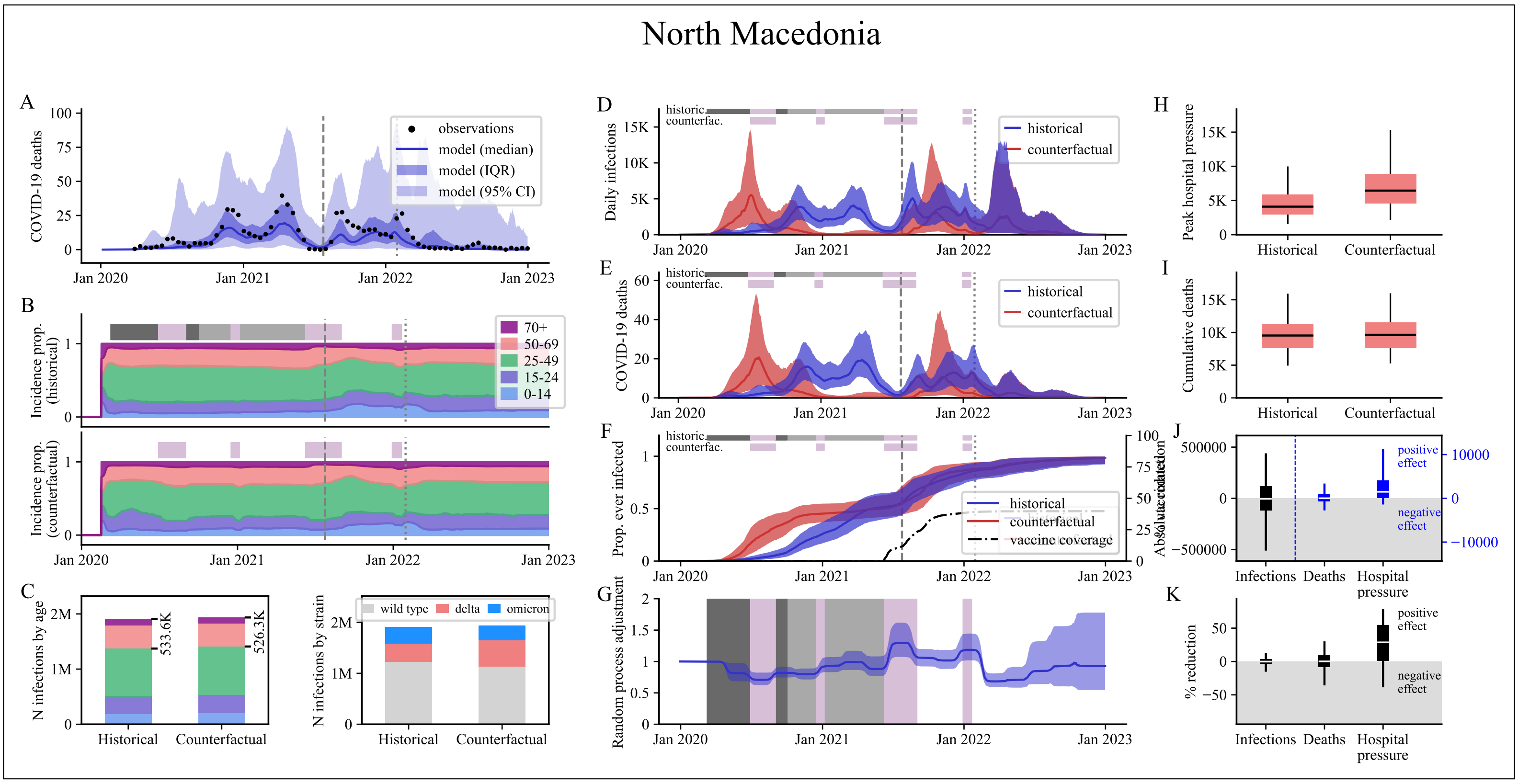
North Macedonia
A
COVID-19 deaths
observations
model (median)
model (IQR)
model (95% CI)
Jan 2020
Jan 2021
Jan 2022
Jan 2023
B
Incidence prop. (historical)
Incidence prop. (counterfactual)
70+
50-69
25-49
15-24
0-14
C
N infections by age
533.6K
526.3K
Historical
Counterfactual
N infections by strain
wild type
delta
omicron
D
Daily infections
historic.
counterfac.
historical
counterfactual
E
COVID-19 deaths
F
Prop. ever infected
vaccine coverage
G
Random process adjustment
H
Peak hospital pressure
I
Cumulative deaths
J
Absolute reduction
positive effect
negative effect
Infections
Deaths
Hospital pressure
K
% reduction

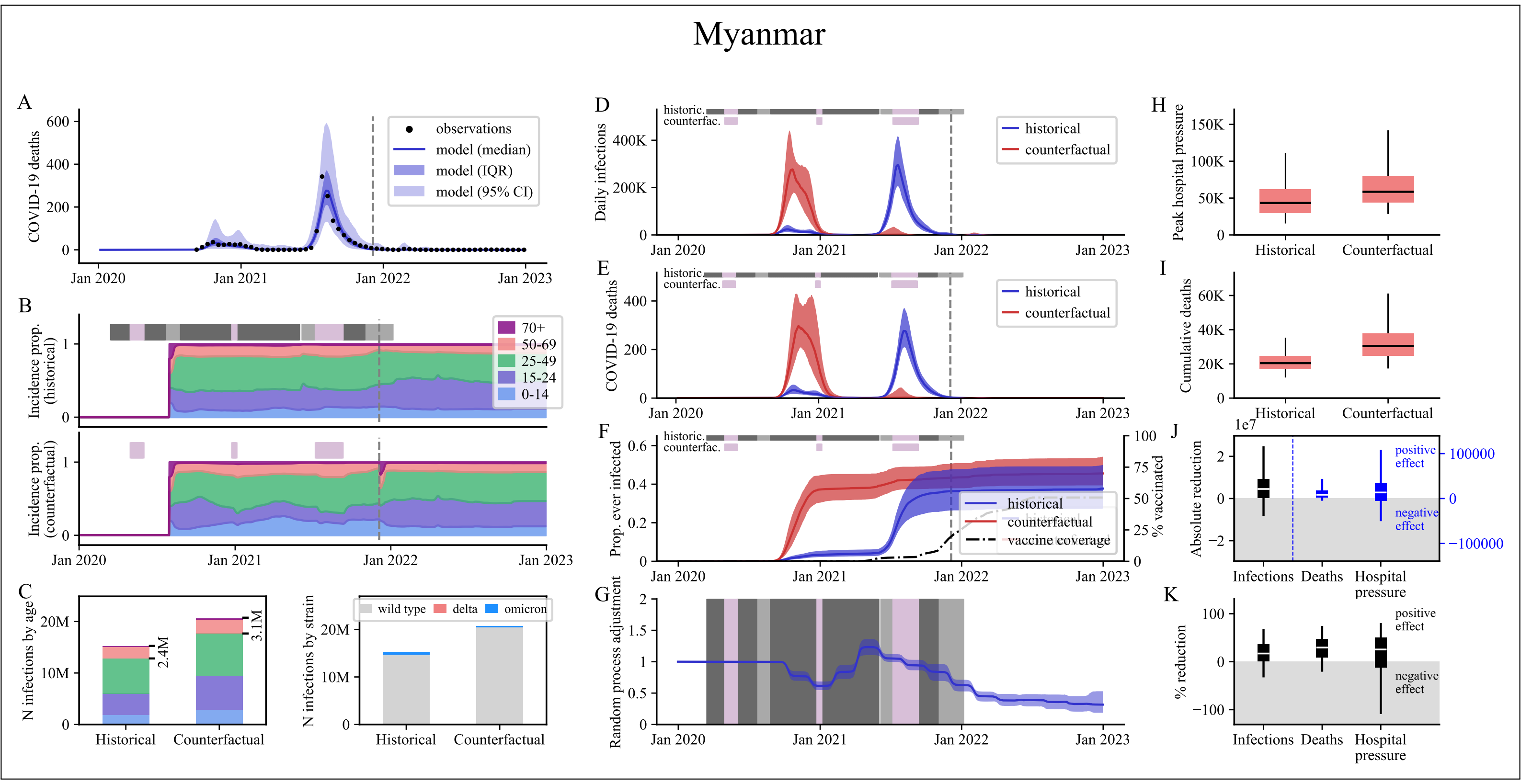
Myanmar
A
COVID-19 deaths
observations
model (median)
model (IQR)
model (95% CI)
Jan 2020
Jan 2021
Jan 2022
Jan 2023
B
Incidence prop. (historical)
Incidence prop. (counterfactual)
70+
50-69
25-49
15-24
0-14
C
N infections by age
2.4M
3.1M
Historical
Counterfactual
N infections by strain
wild type
delta
omicron
D
Daily infections
historic.
counterfac.
historical
counterfactual
E
COVID-19 deaths
F
Prop. ever infected
% vaccinated
vaccine coverage
G
Random process adjustment
H
Peak hospital pressure
I
Cumulative deaths
J
Absolute reduction
1e7
positive effect
negative effect
Infections
Deaths
Hospital pressure
K
% reduction

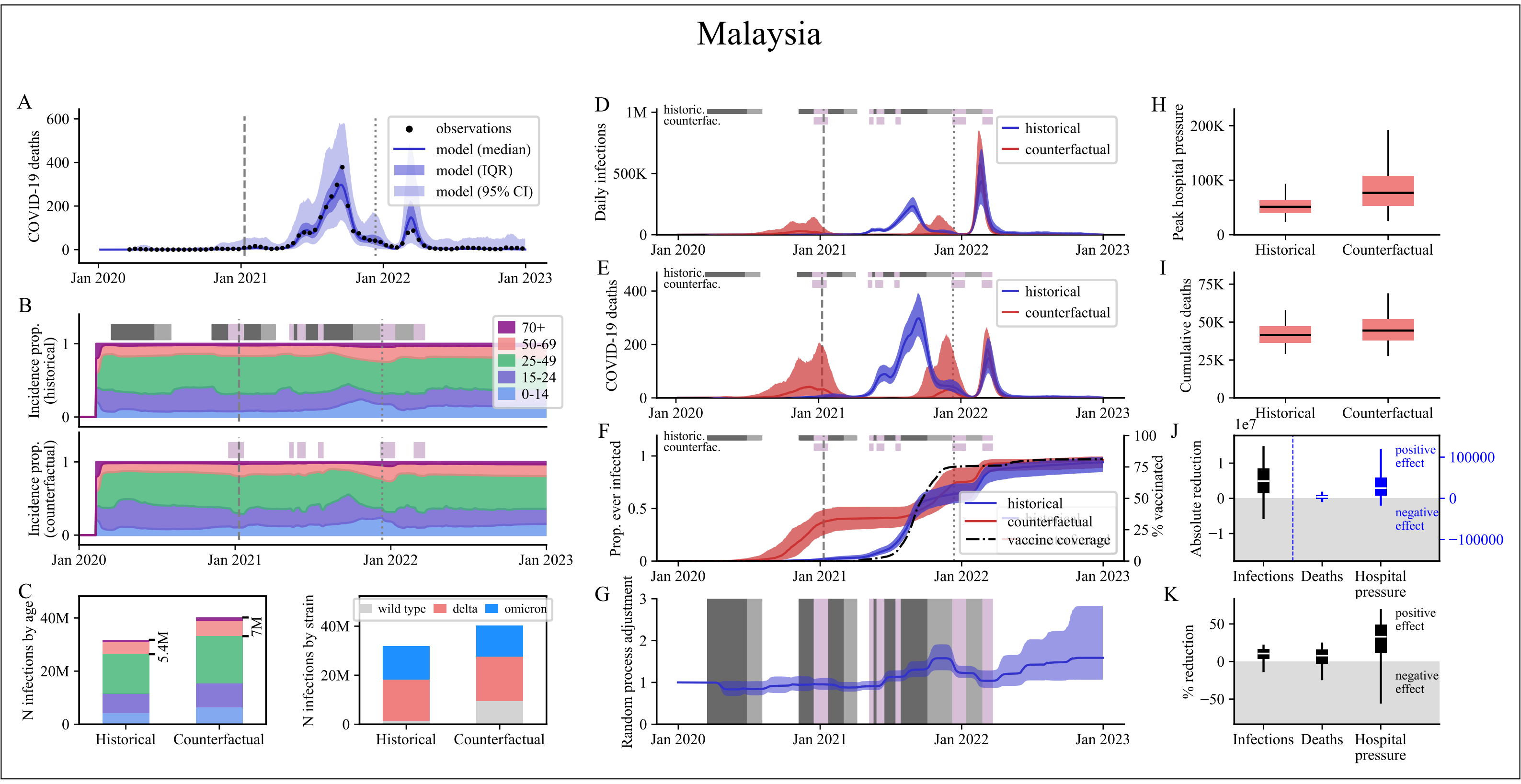

Malaysia
A
COVID-19 deaths
observations
model (median)
model (IQR)
model (95% CI)
Jan 2020
Jan 2021
Jan 2022
Jan 2023
B
Incidence prop. (historical)
Incidence prop. (counterfactual)
70+
50-69
25-49
15-24
0-14
C
N infections by age
5.4M
7M
Historical
Counterfactual
N infections by strain
wild type
delta
omicron
D
Daily infections
historic.
counterfac.
historical
counterfactual
E
COVID-19 deaths
F
Prop. ever infected
% vaccinated
vaccine coverage
G
Random process adjustment
H
Peak hospital pressure
I
Cumulative deaths
J
Absolute reduction
1e7
positive effect
negative effect
Infections
Deaths
Hospital pressure
K
% reduction

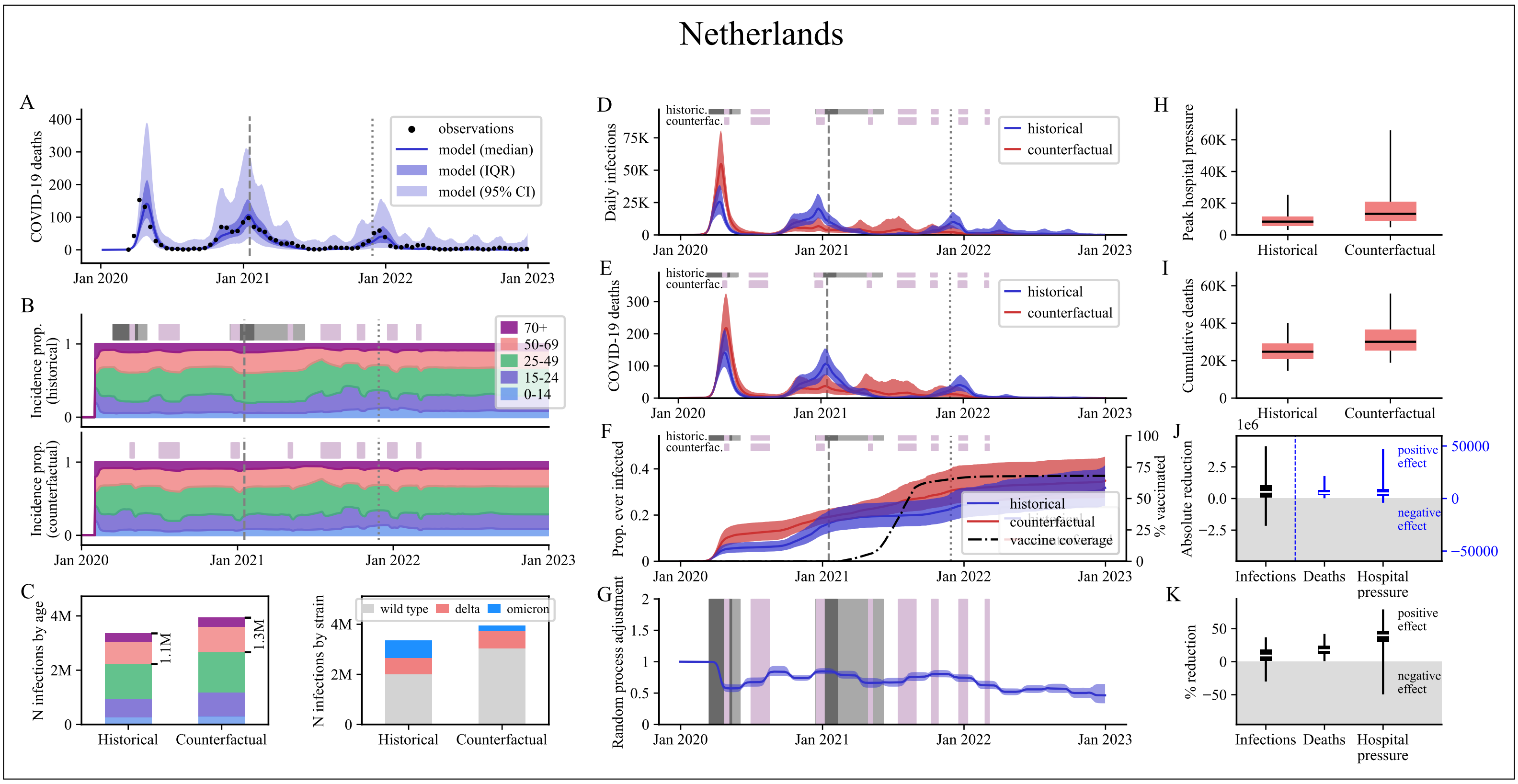

Netherlands
A
COVID-19 deaths
observations
model (median)
model (IQR)
model (95% CI)
Jan 2020
Jan 2021
Jan 2022
Jan 2023
B
Incidence prop. (historical)
Incidence prop. (counterfactual)
70+
50-69
25-49
15-24
0-14
C
N infections by age
1.1M
1.3M
Historical
Counterfactual
N infections by strain
wild type
delta
omicron
D
Daily infections
historic.
counterfac.
historical
counterfactual
E
COVID-19 deaths
F
Prop. ever infected
% vaccinated
vaccine coverage
G
Random process adjustment
H
Peak hospital pressure
I
Cumulative deaths
J
Absolute reduction
1e6
positive effect
negative effect
Infections
Deaths
Hospital pressure
K
% reduction

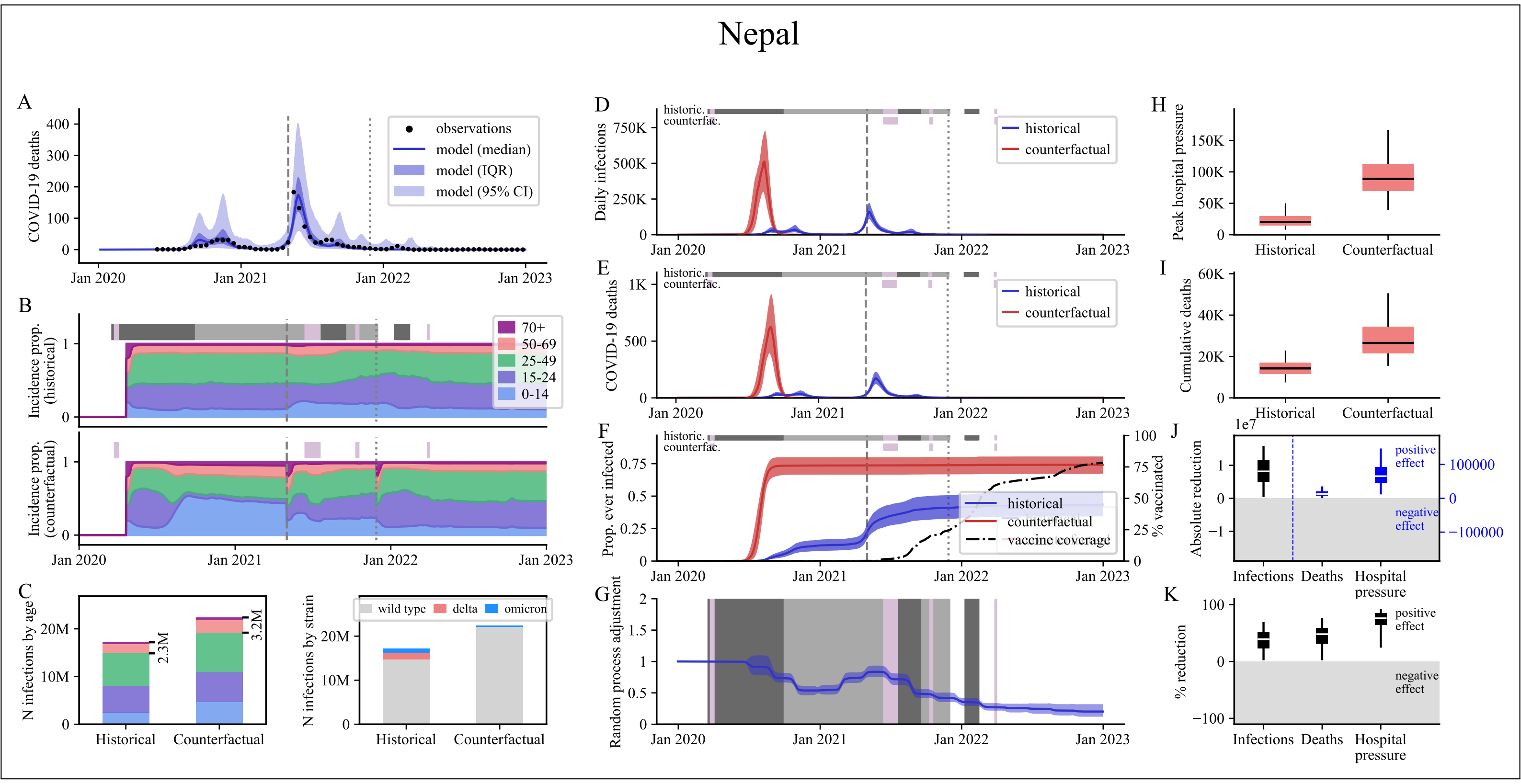

Nepal
A
COVID-19 deaths
observations
model (median)
model (IQR)
model (95% CI)
Jan 2020
Jan 2021
Jan 2022
Jan 2023
B
Incidence prop. (historical)
Incidence prop. (counterfactual)
70+
50-69
25-49
15-24
0-14
C
N infections by age
2.3M
3.2M
N infections by strain
wild type
delta
omicron
Historical
Counterfactual
D
Daily infections
historic.
counterfac.
historical
counterfactual
E
COVID-19 deaths
F
Prop. ever infected
% vaccinated
vaccine coverage
G
Random process adjustment
H
Peak hospital pressure
I
Cumulative deaths
J
Absolute reduction
1e7
positive effect
negative effect
Infections
Deaths
Hospital pressure
K
% reduction

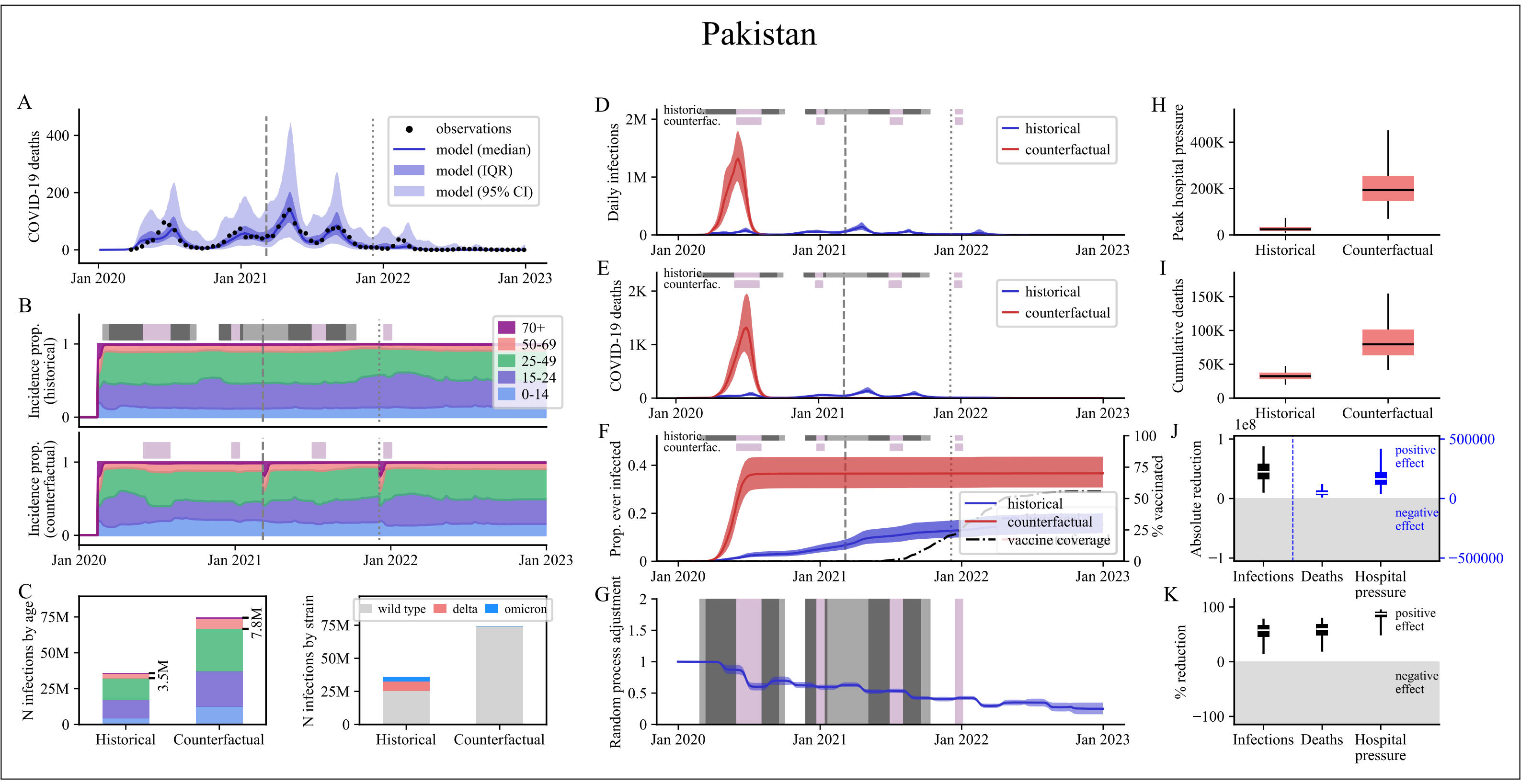

Pakistan
A
COVID-19 deaths
observations
model (median)
model (IQR)
model (95% CI)
Jan 2020
Jan 2021
Jan 2022
Jan 2023
B
Incidence prop. (historical)
Incidence prop. (counterfactual)
70+
50-69
25-49
15-24
0-14
C
N infections by age
3.5M
7.8M
N infections by strain
wild type
delta
omicron
Historical
Counterfactual
D
Daily infections
historic.
counterfac.
historical
counterfactual
E
COVID-19 deaths
F
Prop. ever infected
% vaccinated
vaccine coverage
G
Random process adjustment
H
Peak hospital pressure
I
Cumulative deaths
J
Absolute reduction
1e8
positive effect
negative effect
Infections
Deaths
Hospital pressure
K
% reduction

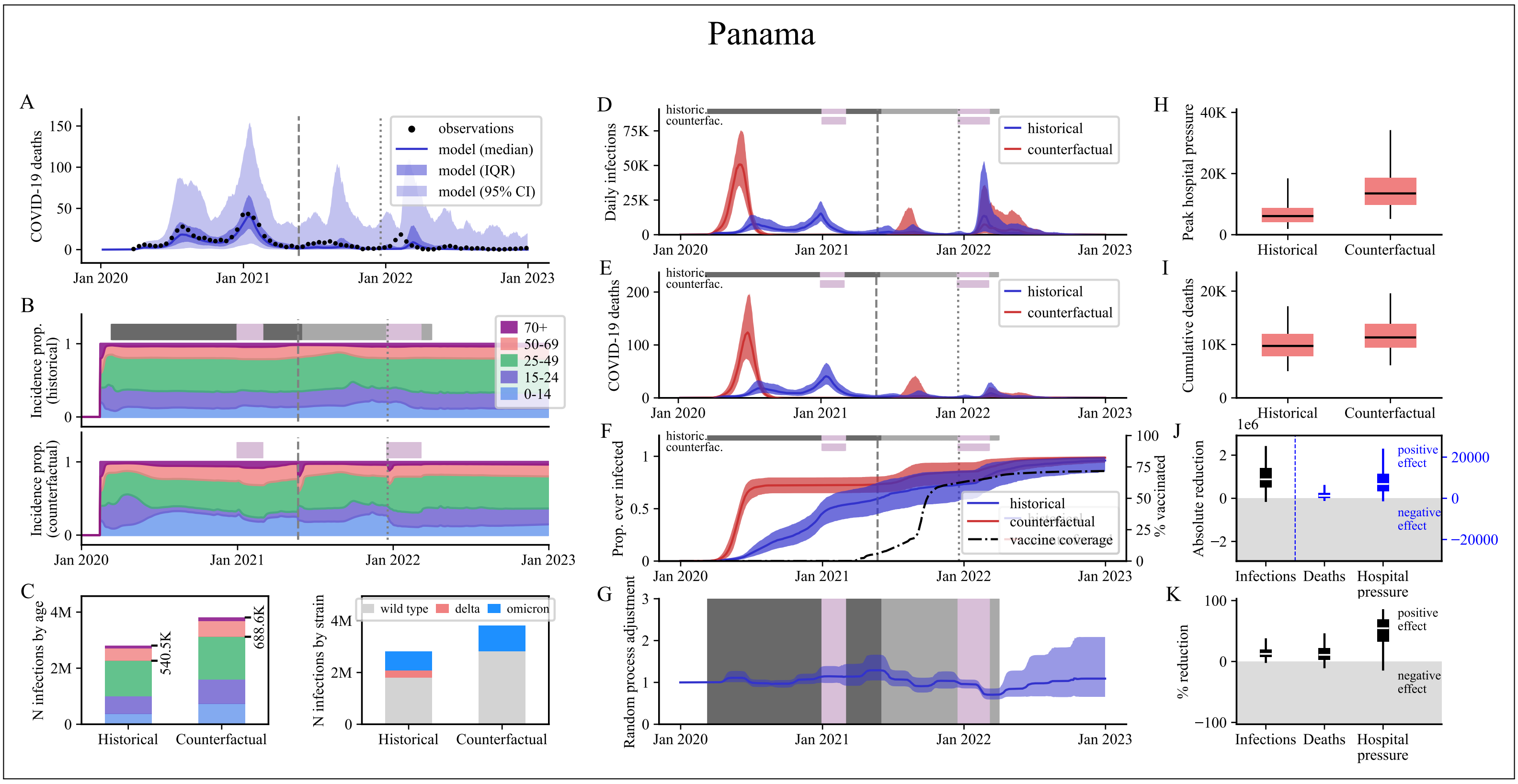
Panama
A
COVID-19 deaths
observations
model (median)
model (IQR)
model (95% CI)
Jan 2020
Jan 2021
Jan 2022
Jan 2023
B
Incidence prop. (historical)
Incidence prop. (counterfactual)
70+
50-69
25-49
15-24
0-14
C
N infections by age
540.5K
688.6K
Historical
Counterfactual
N infections by strain
wild type
delta
omicron
D
historic.
counterfac.
Daily infections
historical
counterfactual
E
COVID-19 deaths
F
Prop. ever infected
% vaccinated
vaccine coverage
G
Random process adjustment
H
Peak hospital pressure
I
Cumulative deaths
J
1e6
Absolute reduction
positive effect
negative effect
Infections
Deaths
Hospital pressure
K
% reduction

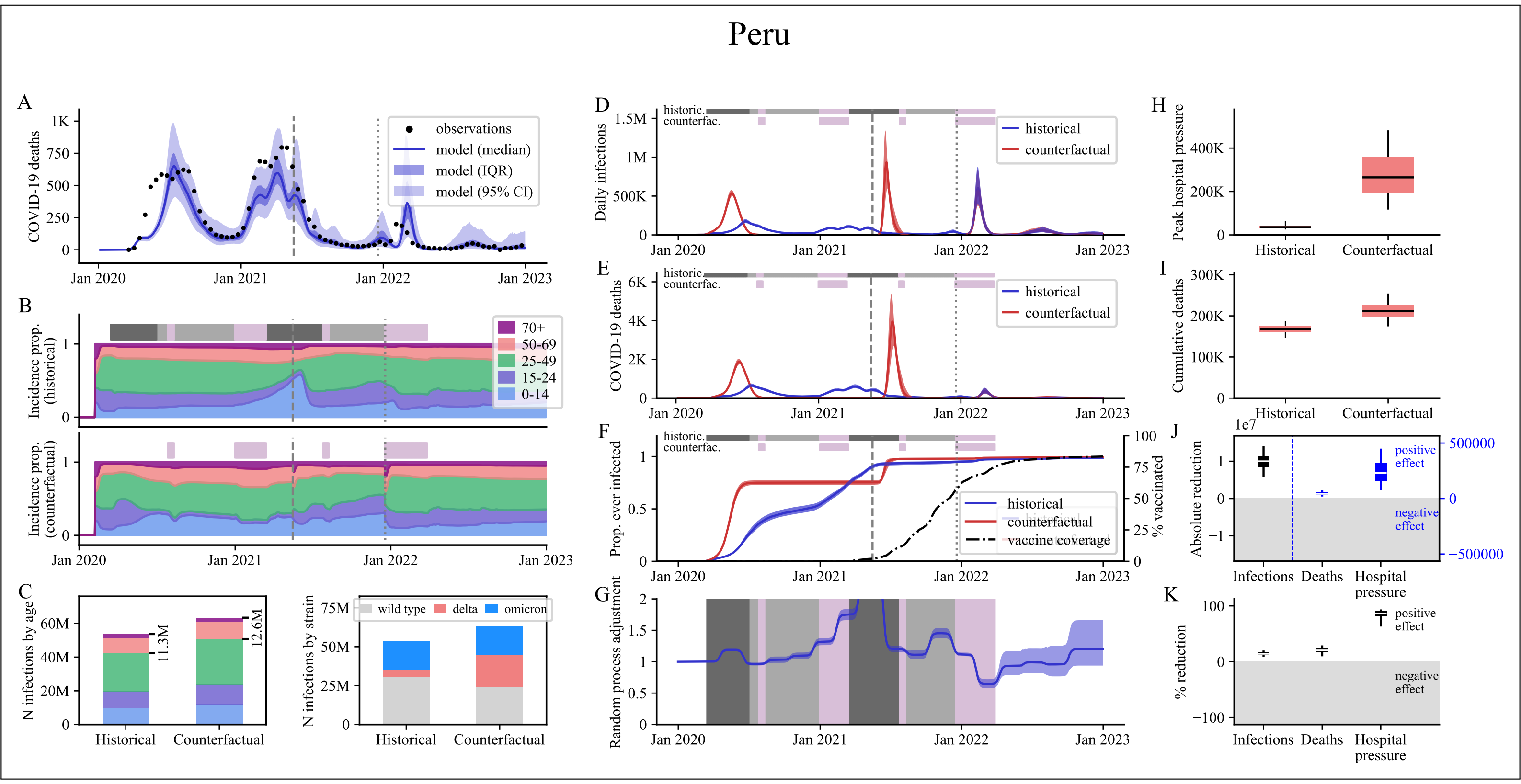
Peru
A
COVID-19 deaths
observations
model (median)
model (IQR)
model (95% CI)
Jan 2020
Jan 2021
Jan 2022
Jan 2023
B
Incidence prop. (historical)
Incidence prop. (counterfactual)
70+
50-69
25-49
15-24
0-14
C
N infections by age
11.3M
12.6M
Historical
Counterfactual
N infections by strain
wild type
delta
omicron
D
historic.
counterfac.
Daily infections
historical
counterfactual
E
COVID-19 deaths
F
Prop. ever infected
% vaccinated
vaccine coverage
G
Random process adjustment
H
Peak hospital pressure
I
Cumulative deaths
J
1e7
Absolute reduction
positive effect
negative effect
Infections
Deaths
Hospital pressure
K
% reduction

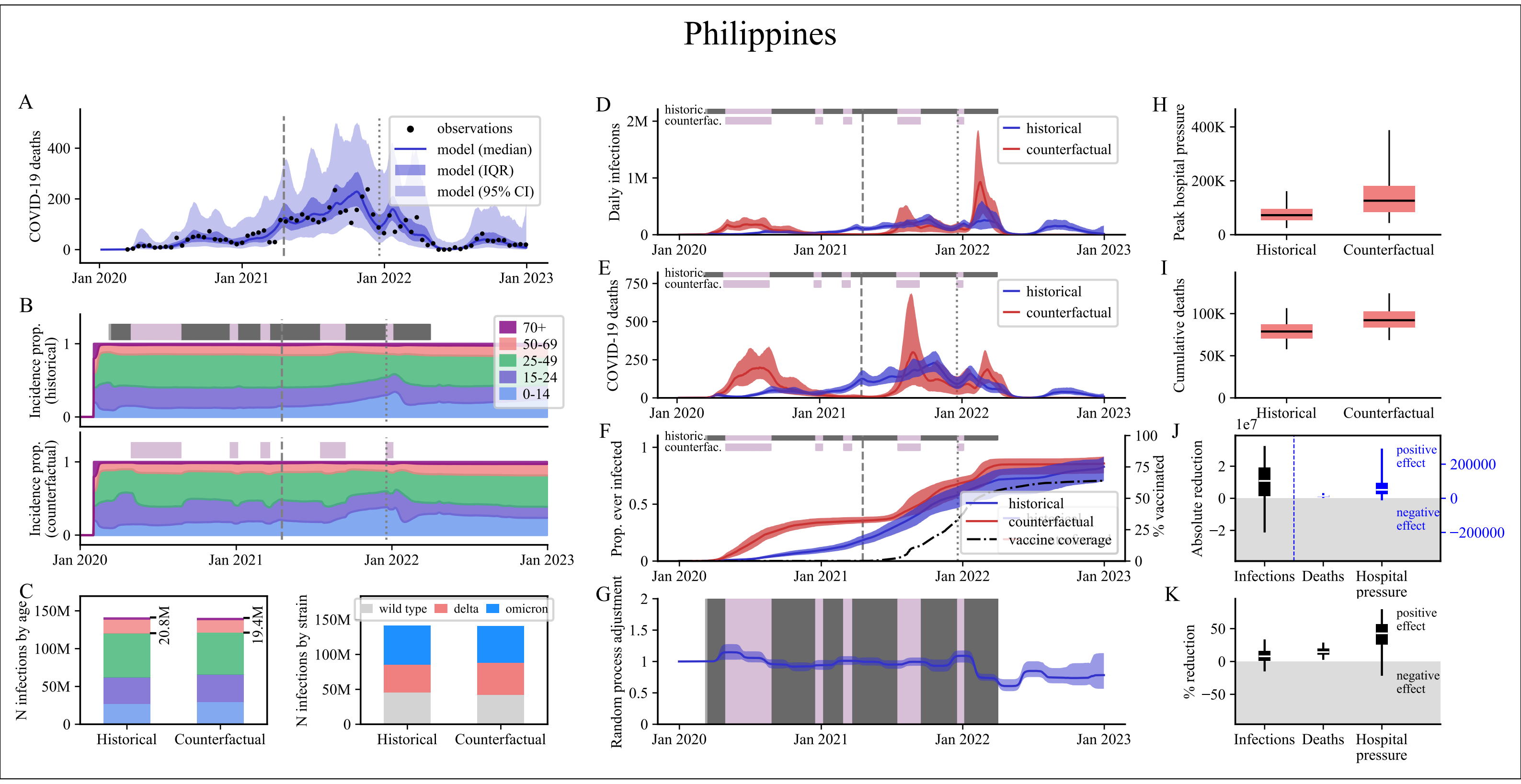
Philippines
A
COVID-19 deaths
observations
model (median)
model (IQR)
model (95% CI)
Jan 2020
Jan 2021
Jan 2022
Jan 2023
B
Incidence prop. (historical)
Incidence prop. (counterfactual)
70+
50-69
25-49
15-24
0-14
C
N infections by age
20.8M
19.4M
Historical
Counterfactual
N infections by strain
wild type
delta
omicron
D
Daily infections
historic.
counterfac.
historical
counterfactual
E
COVID-19 deaths
F
Prop. ever infected
% vaccinated
vaccine coverage
G
Random process adjustment
H
Peak hospital pressure
I
Cumulative deaths
J
Absolute reduction
1e7
positive effect
negative effect
Infections
Deaths
Hospital pressure
K
% reduction

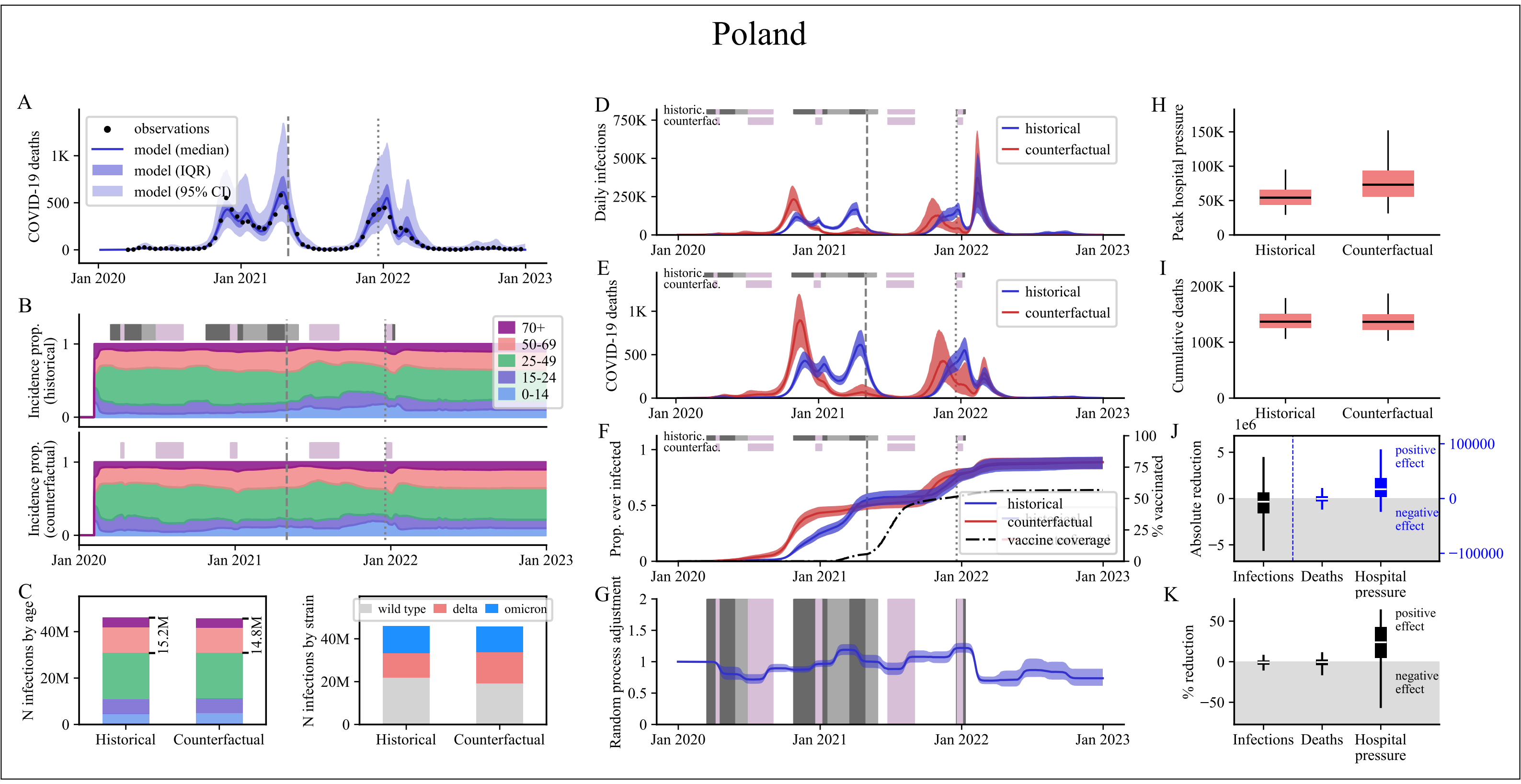
Poland
A
COVID-19 deaths
observations
model (median)
model (IQR)
model (95% CI)
Jan 2020
Jan 2021
Jan 2022
Jan 2023
B
Incidence prop. (historical)
Incidence prop. (counterfactual)
70+
50-69
25-49
15-24
0-14
C
N infections by age
15.2M
14.8M
Historical
Counterfactual
N infections by strain
wild type
delta
omicron
D
Daily infections
historic.
counterfac.
historical
counterfactual
E
COVID-19 deaths
F
Prop. ever infected
% vaccinated
vaccine coverage
G
Random process adjustment
H
Peak hospital pressure
I
Cumulative deaths
J
Absolute reduction
1e6
positive effect
negative effect
Infections
Deaths
Hospital pressure
K
% reduction

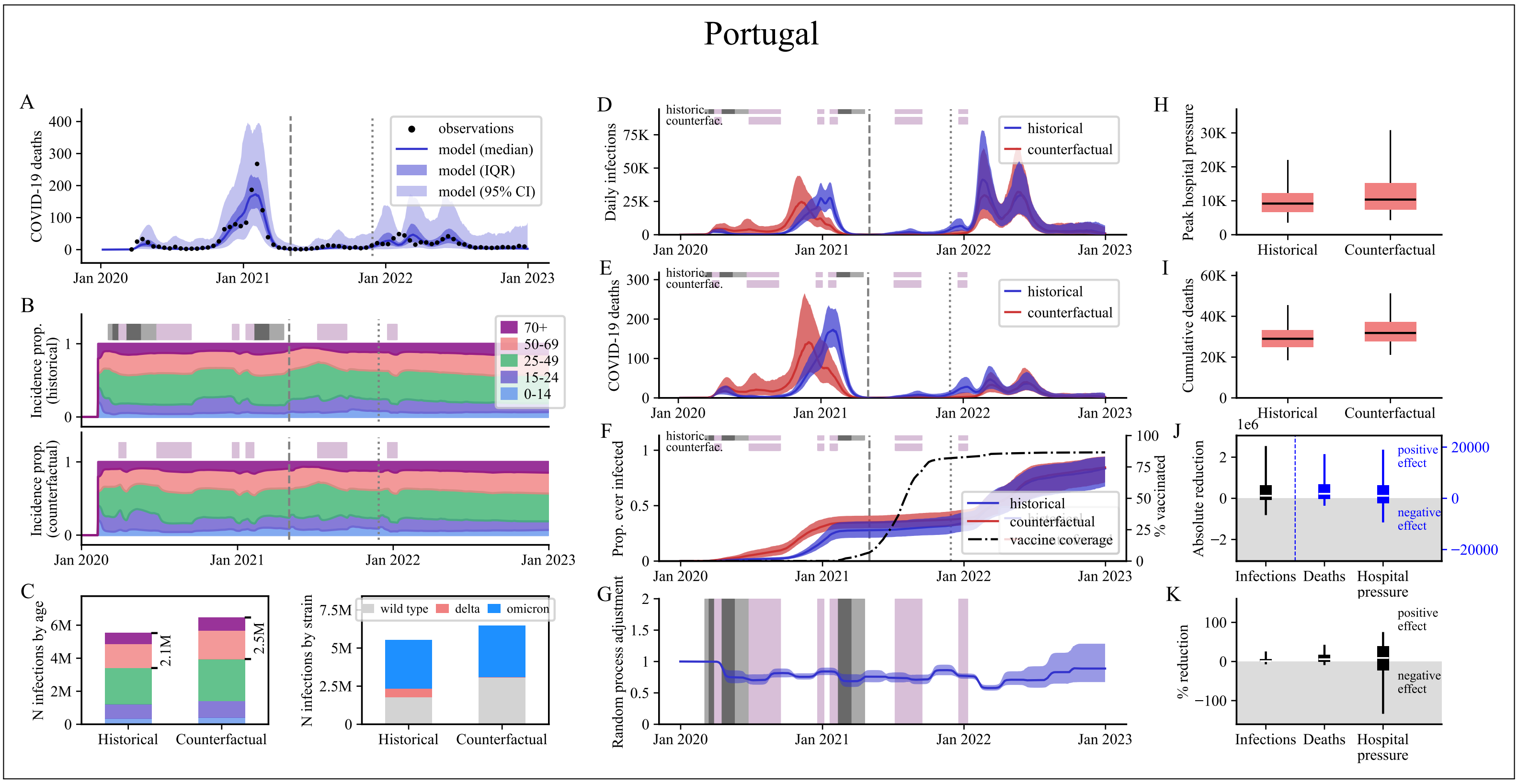
Portugal
A
COVID-19 deaths
observations
model (median)
model (IQR)
model (95% CI)
Jan 2020
Jan 2021
Jan 2022
Jan 2023
B
Incidence prop. (historical)
Incidence prop. (counterfactual)
70+
50-69
25-49
15-24
0-14
C
N infections by age
2.1M
2.5M
Historical
Counterfactual
N infections by strain
wild type
delta
omicron
D
historic.
counterfac.
Daily infections
historical
counterfactual
E
COVID-19 deaths
F
Prop. ever infected
% vaccinated
vaccine coverage
G
Random process adjustment
H
Peak hospital pressure
I
Cumulative deaths
J
1e6
Absolute reduction
positive effect
negative effect
Infections
Deaths
Hospital pressure
K
% reduction

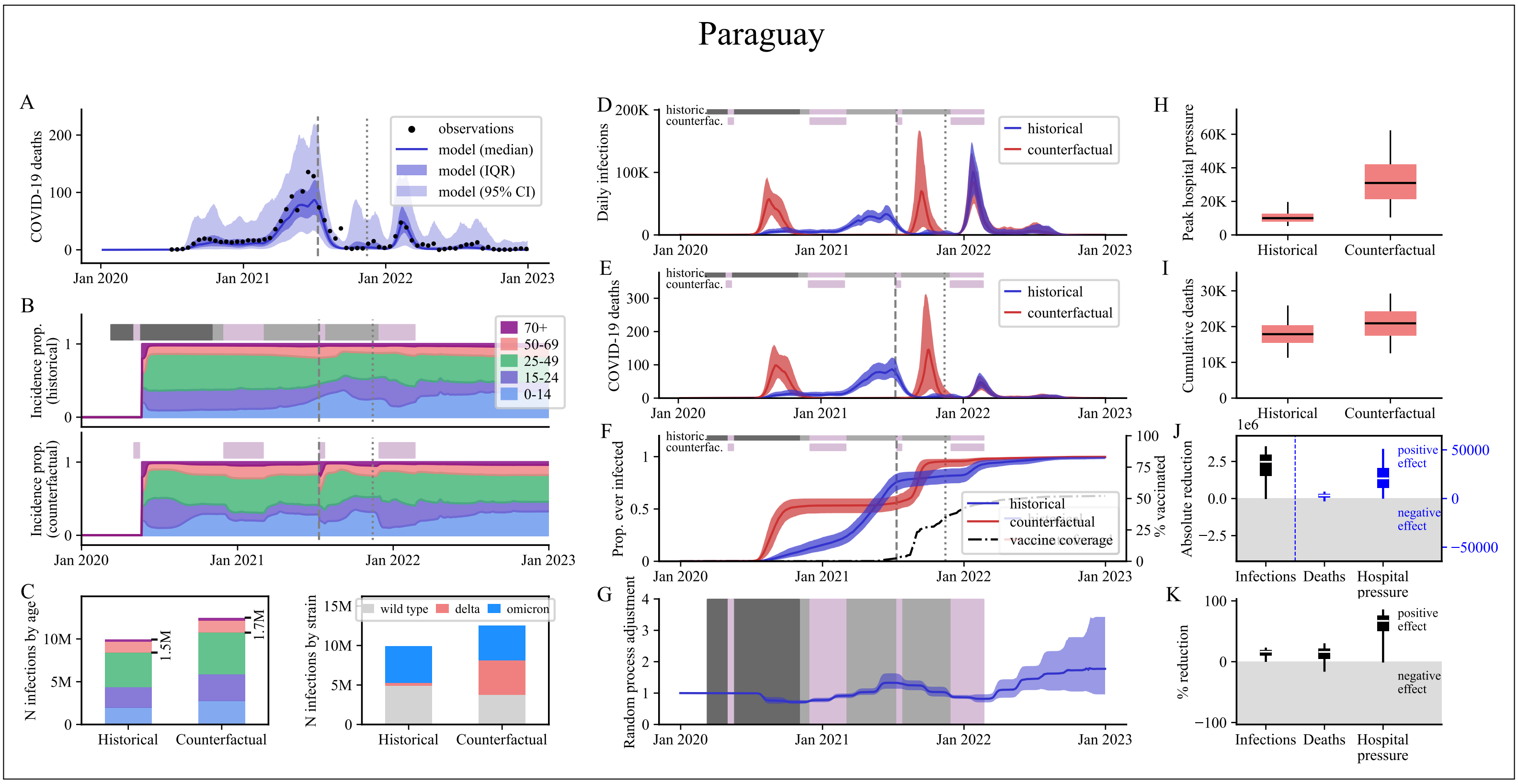
Paraguay
A
COVID-19 deaths
observations
model (median)
model (IQR)
model (95% CI)
Jan 2020
Jan 2021
Jan 2022
Jan 2023
B
Incidence prop. (historical)
Incidence prop. (counterfactual)
70+
50-69
25-49
15-24
0-14
C
N infections by age
1.5M
1.7M
Historical
Counterfactual
N infections by strain
wild type
delta
omicron
D
historic.
counterfac.
Daily infections
historical
counterfactual
E
COVID-19 deaths
F
Prop. ever infected
% vaccinated
vaccine coverage
G
Random process adjustment
H
Peak hospital pressure
I
Cumulative deaths
J
1e6
Absolute reduction
positive effect
negative effect
Infections
Deaths
Hospital pressure
K
% reduction

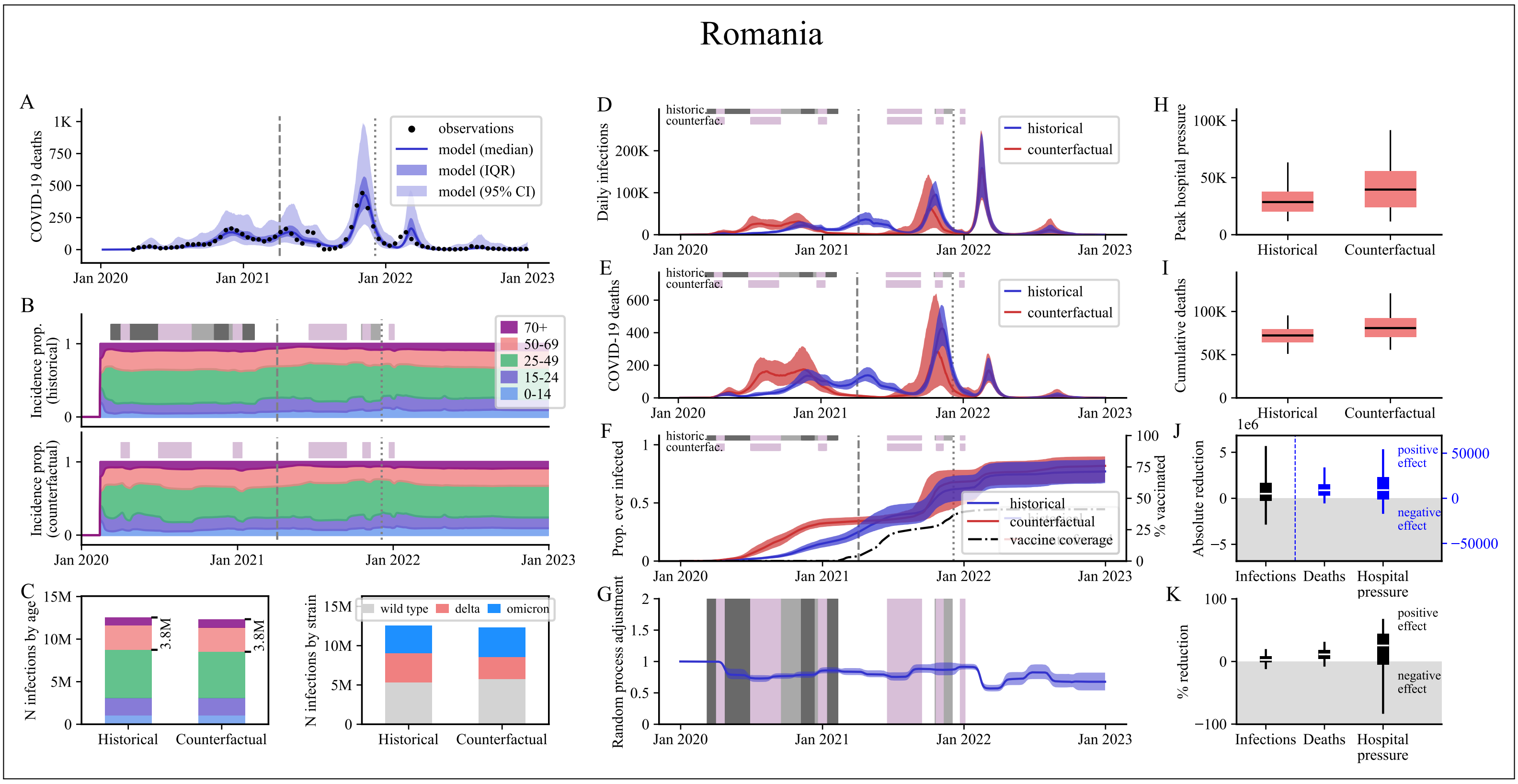
Romania
A
COVID-19 deaths
observations
model (median)
model (IQR)
model (95% CI)
B
Incidence prop. (historical)
Incidence prop. (counterfactual)
70+
50-69
25-49
15-24
0-14
C
N infections by age
3.8M
3.8M
N infections by strain
wild type
delta
omicron
Historical
Counterfactual
D
Daily infections
historic.
counterfac.
historical
counterfactual
E
COVID-19 deaths
F
Prop. ever infected
% vaccinated
vaccine coverage
G
Random process adjustment
H
Peak hospital pressure
I
Cumulative deaths
J
Absolute reduction
positive effect
negative effect
K
% reduction
Infections
Deaths
Hospital pressure
Jan 2020
Jan 2021
Jan 2022
Jan 2023

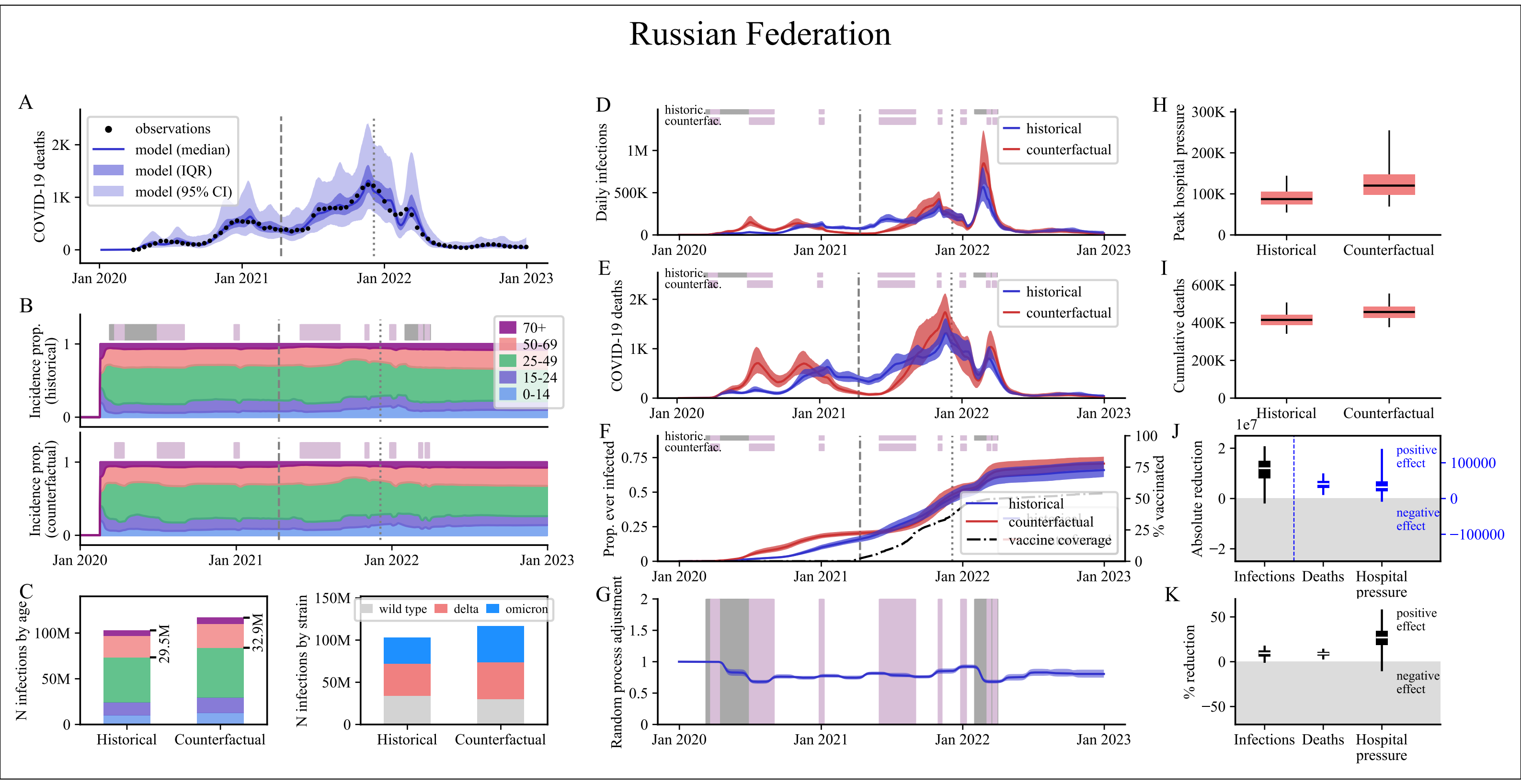
Russian Federation
A
COVID-19 deaths
observations
model (median)
model (IQR)
model (95% CI)
B
Incidence prop. (historical)
Incidence prop. (counterfactual)
70+
50-69
25-49
15-24
0-14
C
N infections by age
29.5M
32.9M
N infections by strain
wild type
delta
omicron
Historical
Counterfactual
D
Daily infections
historic.
counterfac.
historical
counterfactual
E
COVID-19 deaths
F
Prop. ever infected
% vaccinated
vaccine coverage
G
Random process adjustment
H
Peak hospital pressure
I
Cumulative deaths
J
Absolute reduction
positive effect
negative effect
K
% reduction
Infections
Deaths
Hospital pressure
Jan 2020
Jan 2021
Jan 2022
Jan 2023

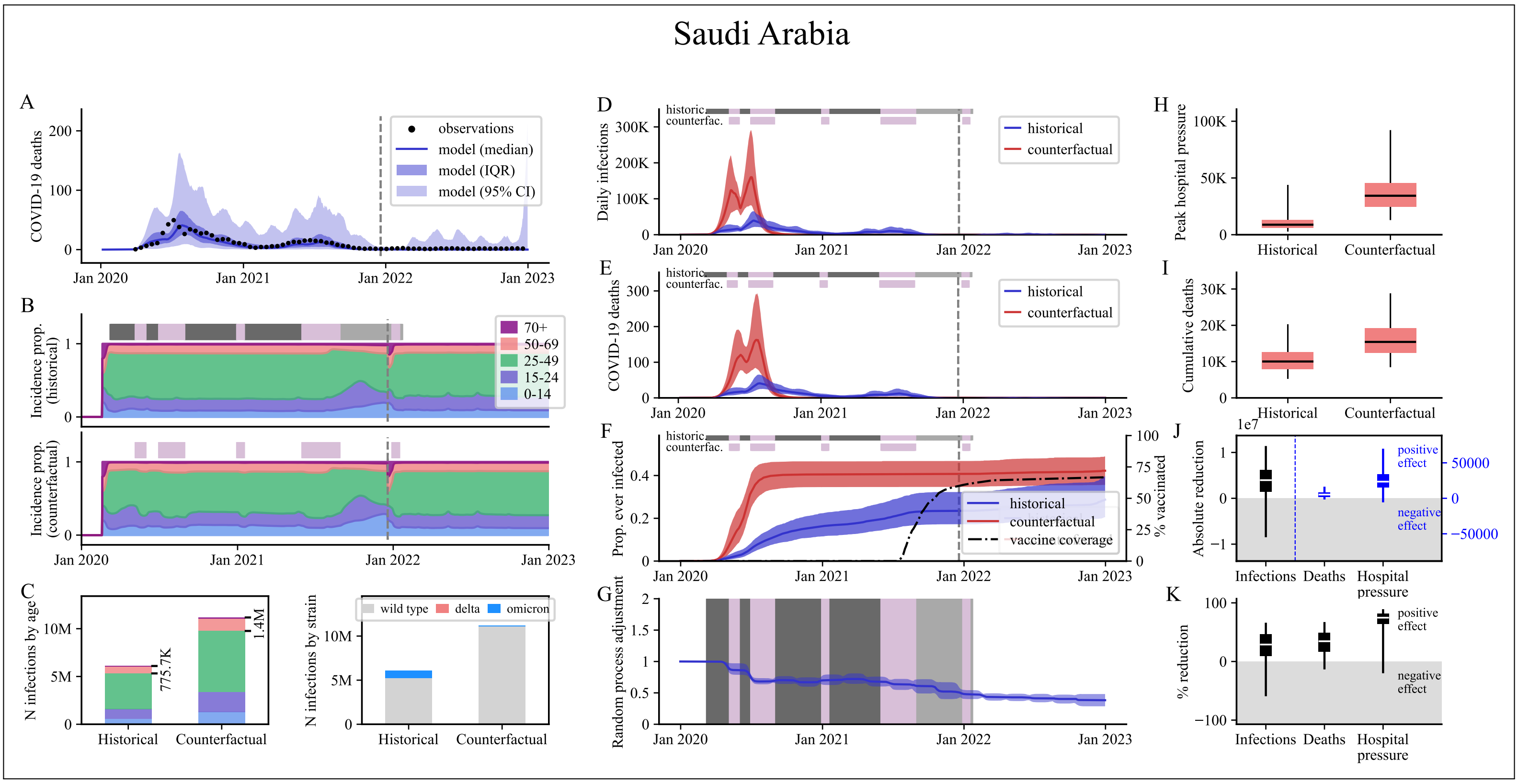
Saudi Arabia
A
COVID-19 deaths
observations
model (median)
model (IQR)
model (95% CI)
Jan 2020
Jan 2021
Jan 2022
Jan 2023
B
Incidence prop. (historical)
Incidence prop. (counterfactual)
70+
50-69
25-49
15-24
0-14
C
N infections by age
775.7K
1.4M
Historical
Counterfactual
N infections by strain
wild type
delta
omicron
D
historic.
counterfac.
Daily infections
historical
counterfactual
E
COVID-19 deaths
F
Prop. ever infected
% vaccinated
vaccine coverage
G
Random process adjustment
H
Peak hospital pressure
I
Cumulative deaths
J
1e7
Absolute reduction
positive effect
negative effect
Infections
Deaths
Hospital pressure
K
% reduction

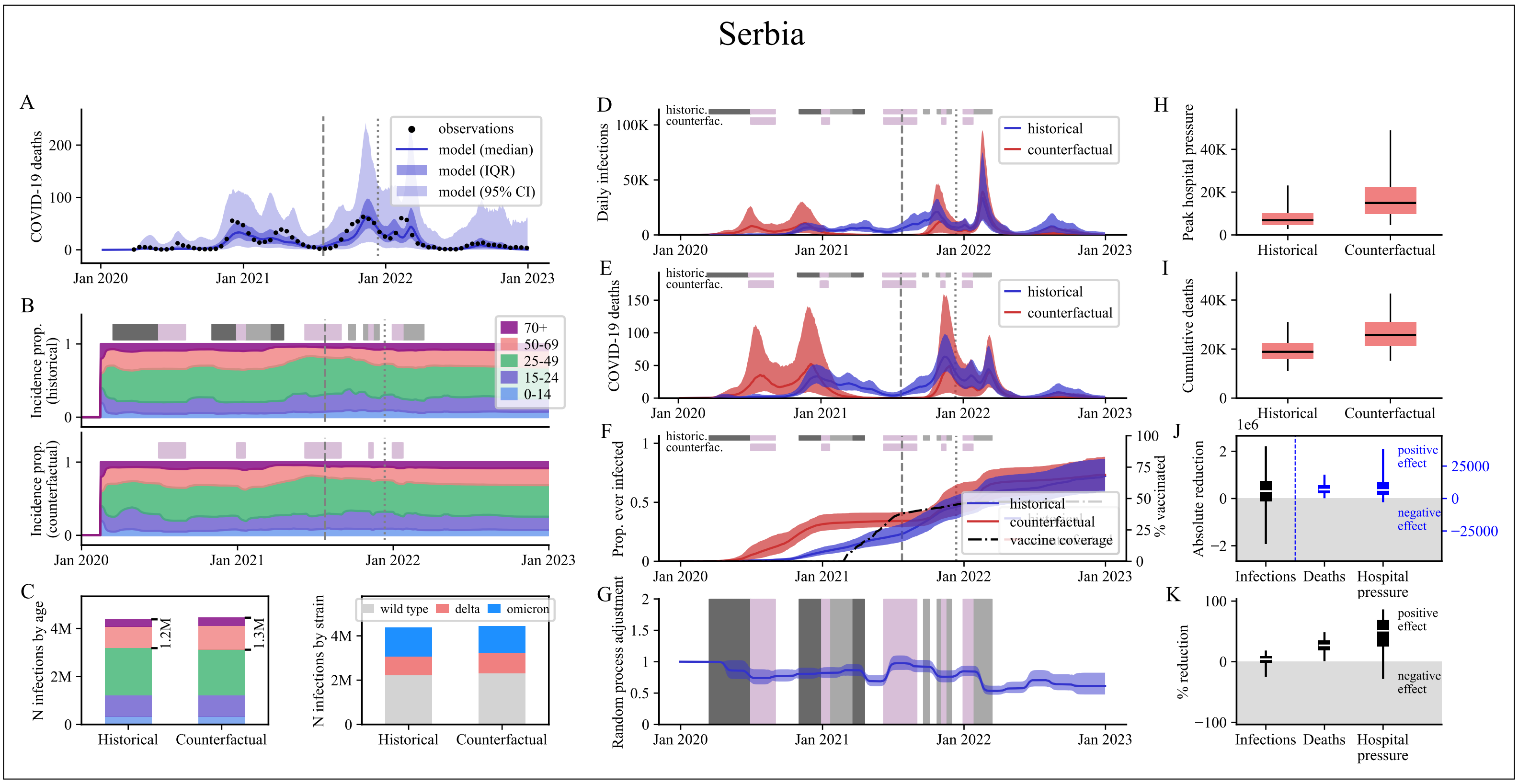
Serbia
A
COVID-19 deaths
observations
model (median)
model (IQR)
model (95% CI)
Jan 2020
Jan 2021
Jan 2022
Jan 2023
B
Incidence prop. (historical)
Incidence prop. (counterfactual)
70+
50-69
25-49
15-24
0-14
C
N infections by age
1.2M
1.3M
Historical
Counterfactual
N infections by strain
wild type
delta
omicron
D
historic.
counterfac.
Daily infections
historical
counterfactual
E
COVID-19 deaths
F
Prop. ever infected
% vaccinated
vaccine coverage
G
Random process adjustment
H
Peak hospital pressure
I
Cumulative deaths
J
1e6
Absolute reduction
positive effect
negative effect
Infections
Deaths
Hospital pressure
K
% reduction

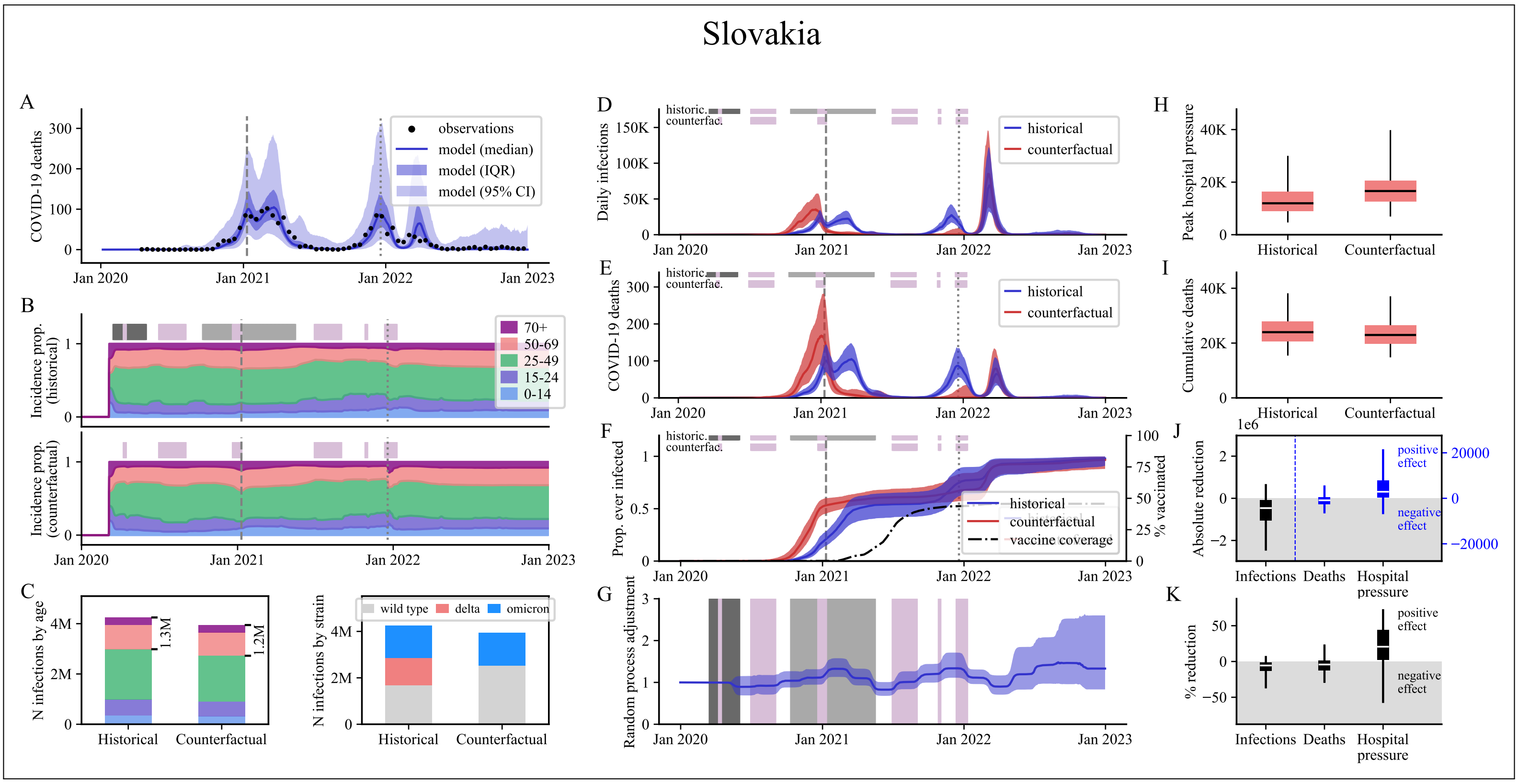
Slovakia
A
COVID-19 deaths
observations
model (median)
model (IQR)
model (95% CI)
Jan 2020
Jan 2021
Jan 2022
Jan 2023
B
Incidence prop. (historical)
Incidence prop. (counterfactual)
70+
50-69
25-49
15-24
0-14
C
N infections by age
1.3M
1.2M
Historical
Counterfactual
N infections by strain
wild type
delta
omicron
D
historic.
counterfac.
Daily infections
historical
counterfactual
E
COVID-19 deaths
F
Prop. ever infected
% vaccinated
vaccine coverage
G
Random process adjustment
H
Peak hospital pressure
I
Cumulative deaths
J
1e6
Absolute reduction
positive effect
negative effect
Infections
Deaths
Hospital pressure
K
% reduction

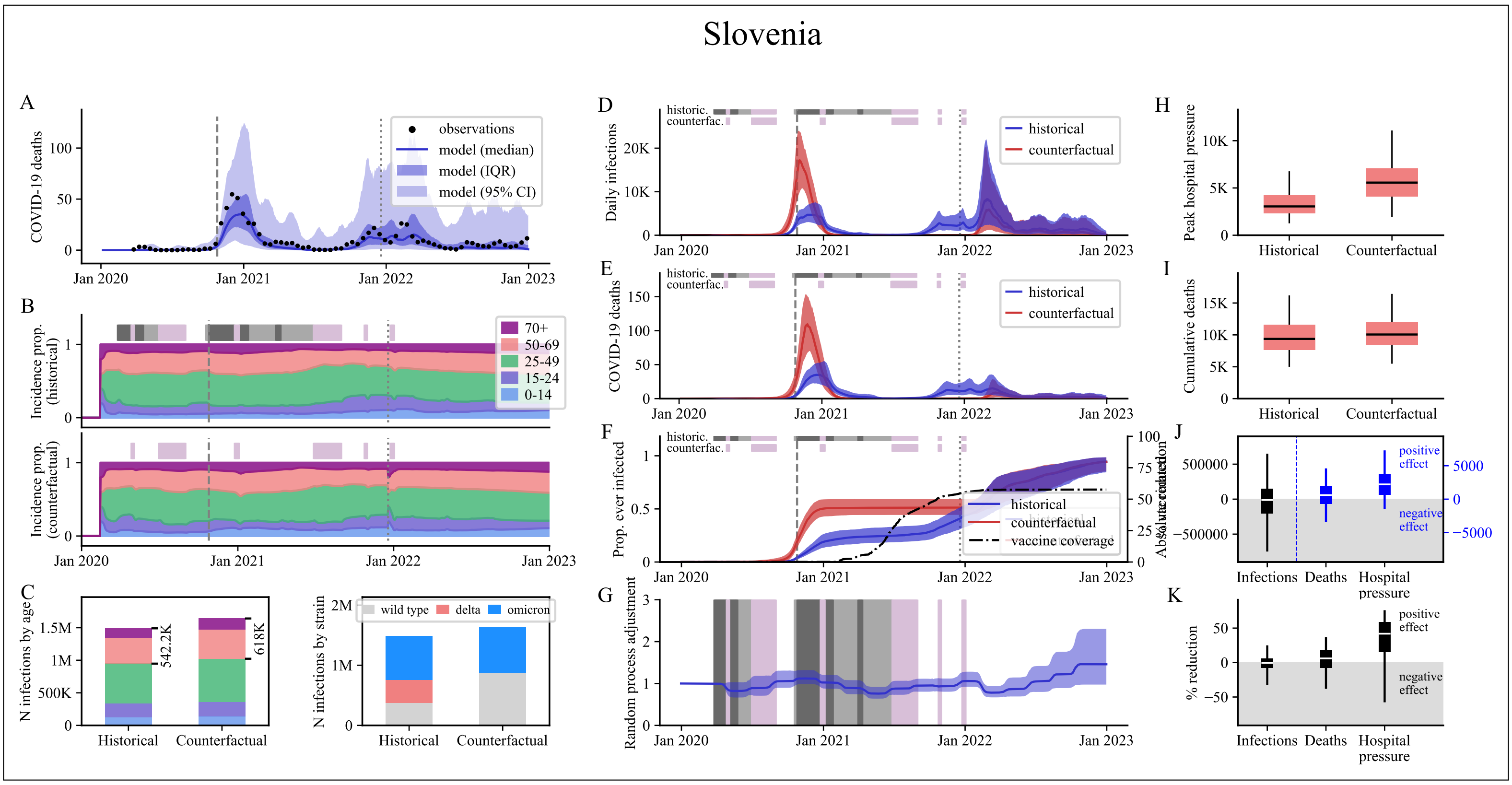
Slovenia
A
COVID-19 deaths
observations
model (median)
model (IQR)
model (95% CI)
Jan 2020
Jan 2021
Jan 2022
Jan 2023
B
Incidence prop. (historical)
Incidence prop. (counterfactual)
70+
50-69
25-49
15-24
0-14
C
N infections by age
542.2K
618K
Historical
Counterfactual
N infections by strain
wild type
delta
omicron
D
historic.
counterfac.
Daily infections
historical
counterfactual
E
COVID-19 deaths
F
Prop. ever infected
% vaccinated
vaccine coverage
G
Random process adjustment
H
Peak hospital pressure
I
Cumulative deaths
J
Absolute reduction
positive effect
negative effect
Infections
Deaths
Hospital pressure
K
% reduction

Sweden

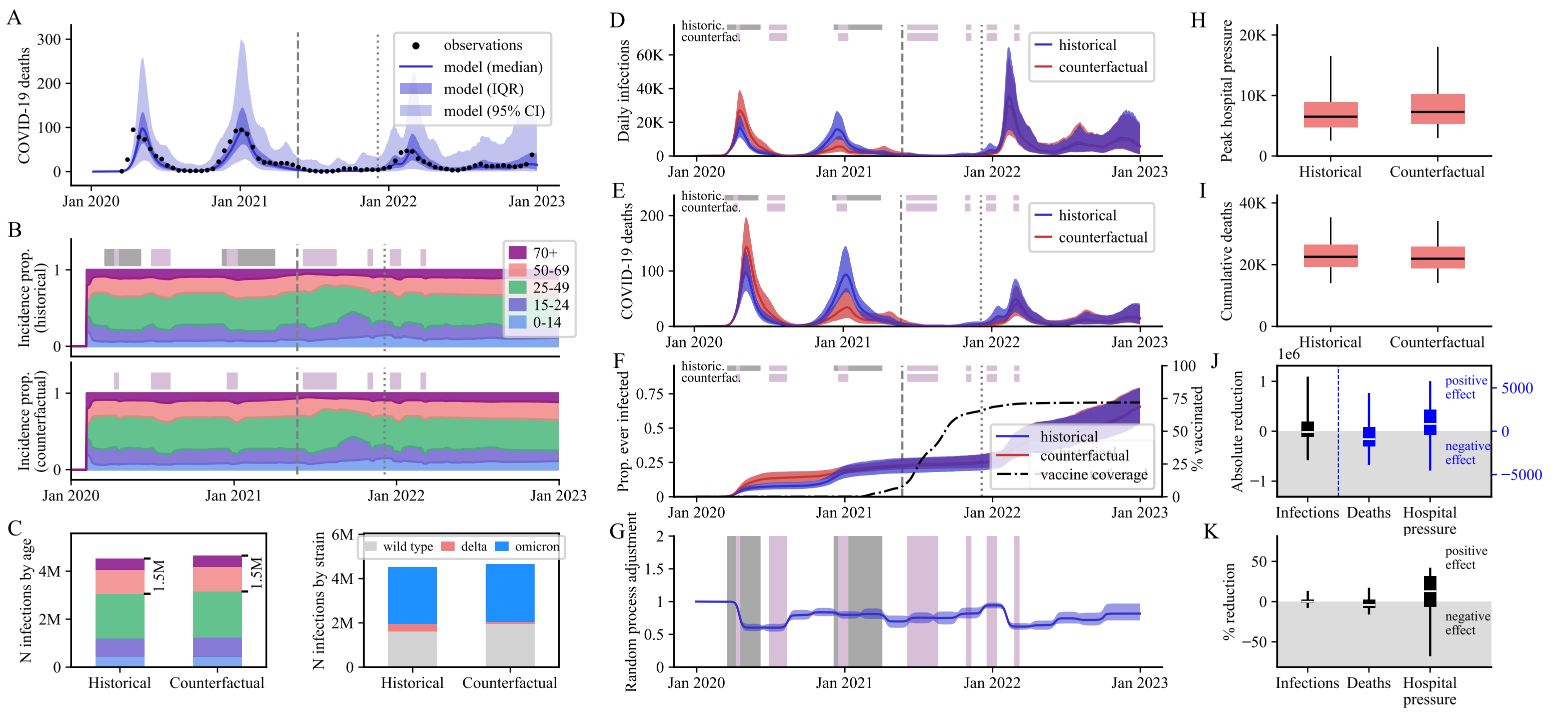

Thailand

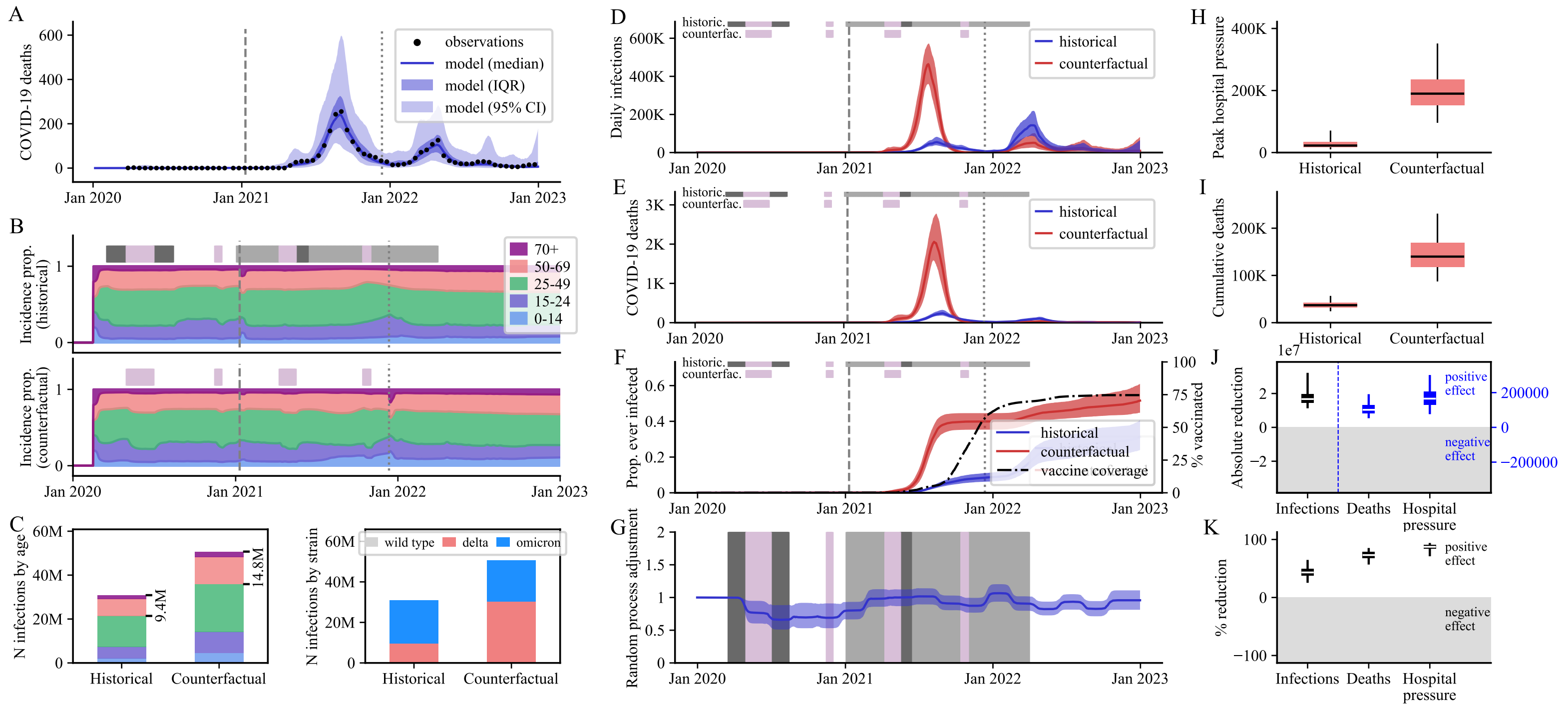

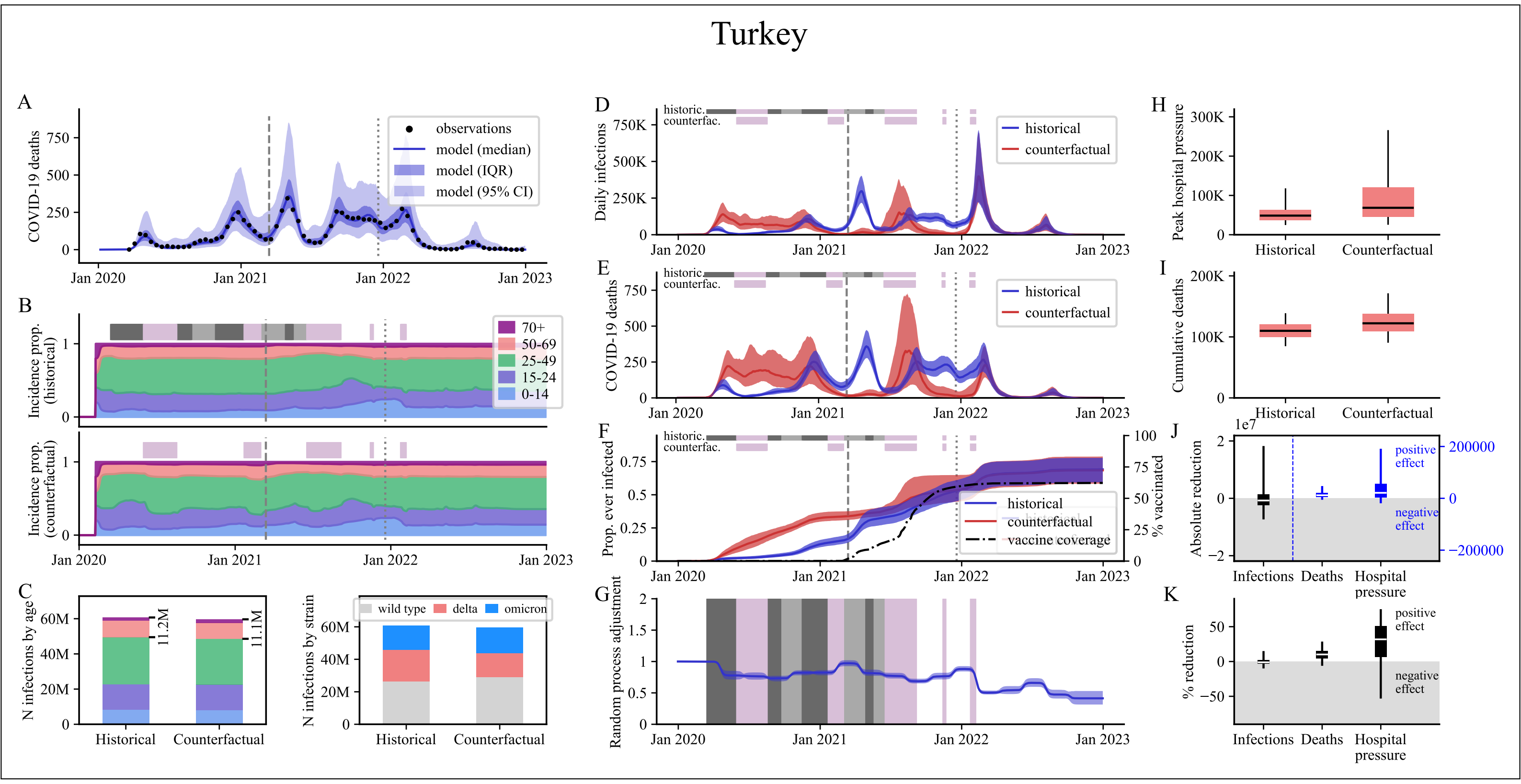
Turkey
A
COVID-19 deaths
observations
model (median)
model (IQR)
model (95% CI)
Jan 2020
Jan 2021
Jan 2022
Jan 2023
B
Incidence prop. (historical)
Incidence prop. (counterfactual)
70+
50-69
25-49
15-24
0-14
C
N infections by age
11.2M
11.1M
Historical
Counterfactual
N infections by strain
wild type
delta
omicron
D
historic.
counterfac.
Daily infections
historical
counterfactual
E
COVID-19 deaths
F
Prop. ever infected
% vaccinated
vaccine coverage
G
Random process adjustment
H
Peak hospital pressure
I
Cumulative deaths
J
1e7
Absolute reduction
positive effect
negative effect
Infections
Deaths
Hospital pressure
K
% reduction

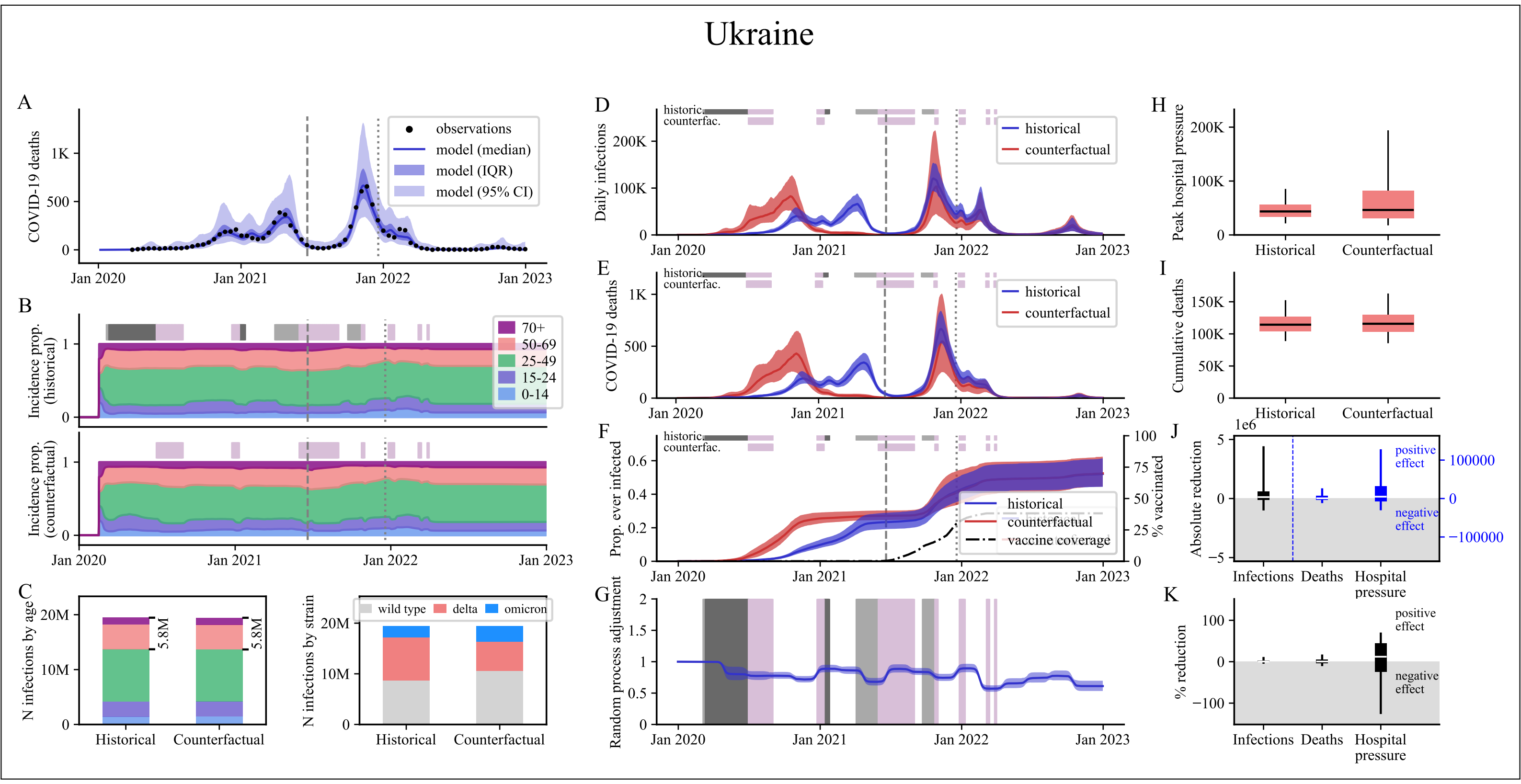
Ukraine
A
COVID-19 deaths
observations
model (median)
model (IQR)
model (95% CI)
Jan 2020
Jan 2021
Jan 2022
Jan 2023
B
Incidence prop. (historical)
Incidence prop. (counterfactual)
70+
50-69
25-49
15-24
0-14
C
N infections by age
5.8M
5.8M
Historical
Counterfactual
N infections by strain
wild type
delta
omicron
D
historic.
counterfac.
Daily infections
historical
counterfactual
E
COVID-19 deaths
F
Prop. ever infected
% vaccinated
vaccine coverage
G
Random process adjustment
H
Peak hospital pressure
I
Cumulative deaths
J
1e6
Absolute reduction
positive effect
negative effect
Infections
Deaths
Hospital pressure
K
% reduction

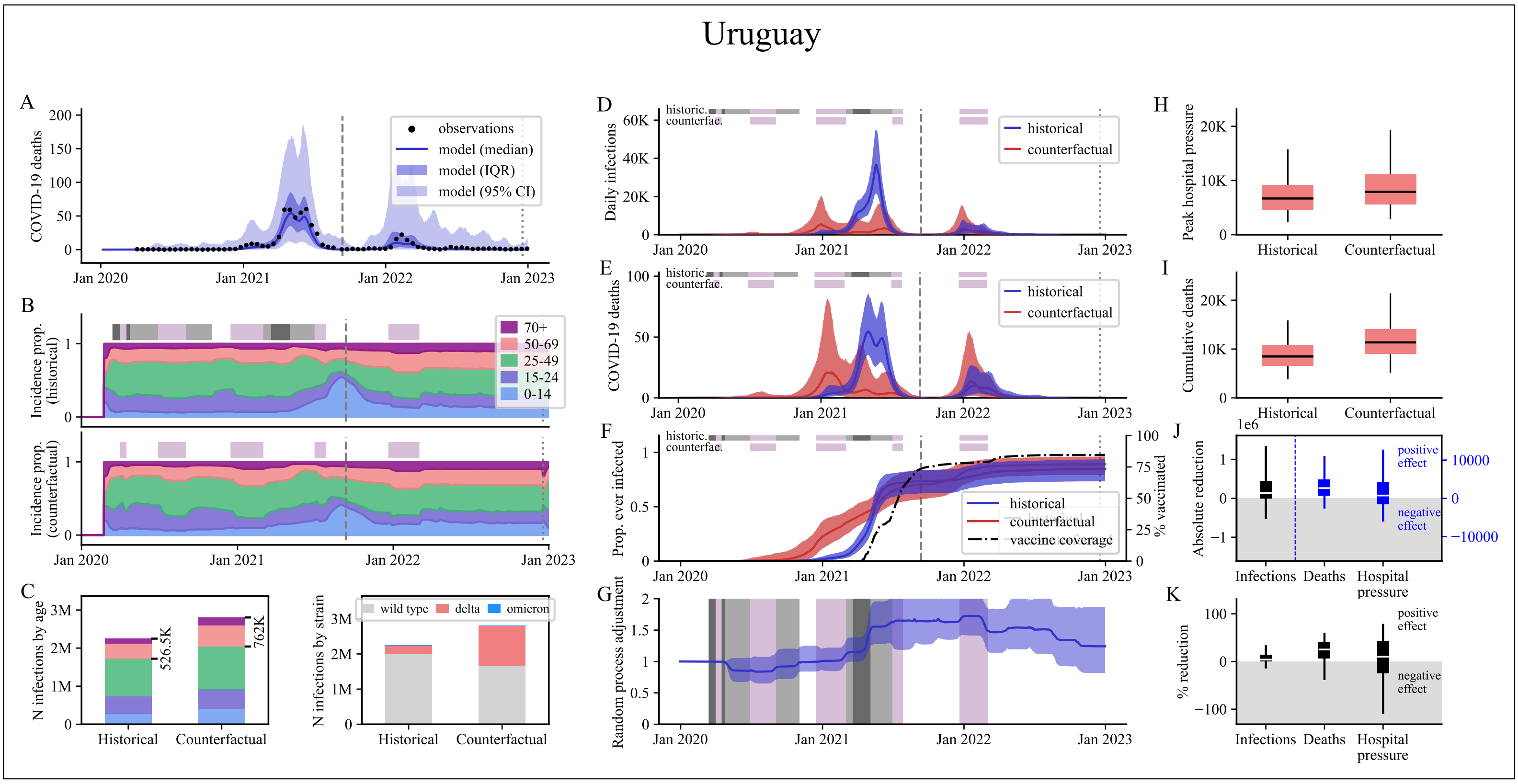
Uruguay
A
COVID-19 deaths
observations
model (median)
model (IQR)
model (95% CI)
Jan 2020
Jan 2021
Jan 2022
Jan 2023
B
Incidence prop. (historical)
Incidence prop. (counterfactual)
70+
50-69
25-49
15-24
0-14
C
N infections by age
526.5K
762K
Historical
Counterfactual
N infections by strain
wild type
delta
omicron
D
historic.
counterfac.
Daily infections
historical
counterfactual
E
COVID-19 deaths
F
Prop. ever infected
% vaccinated
vaccine coverage
G
Random process adjustment
H
Peak hospital pressure
I
Cumulative deaths
J
1e6
Absolute reduction
positive effect
negative effect
Infections
Deaths
Hospital pressure
K
% reduction

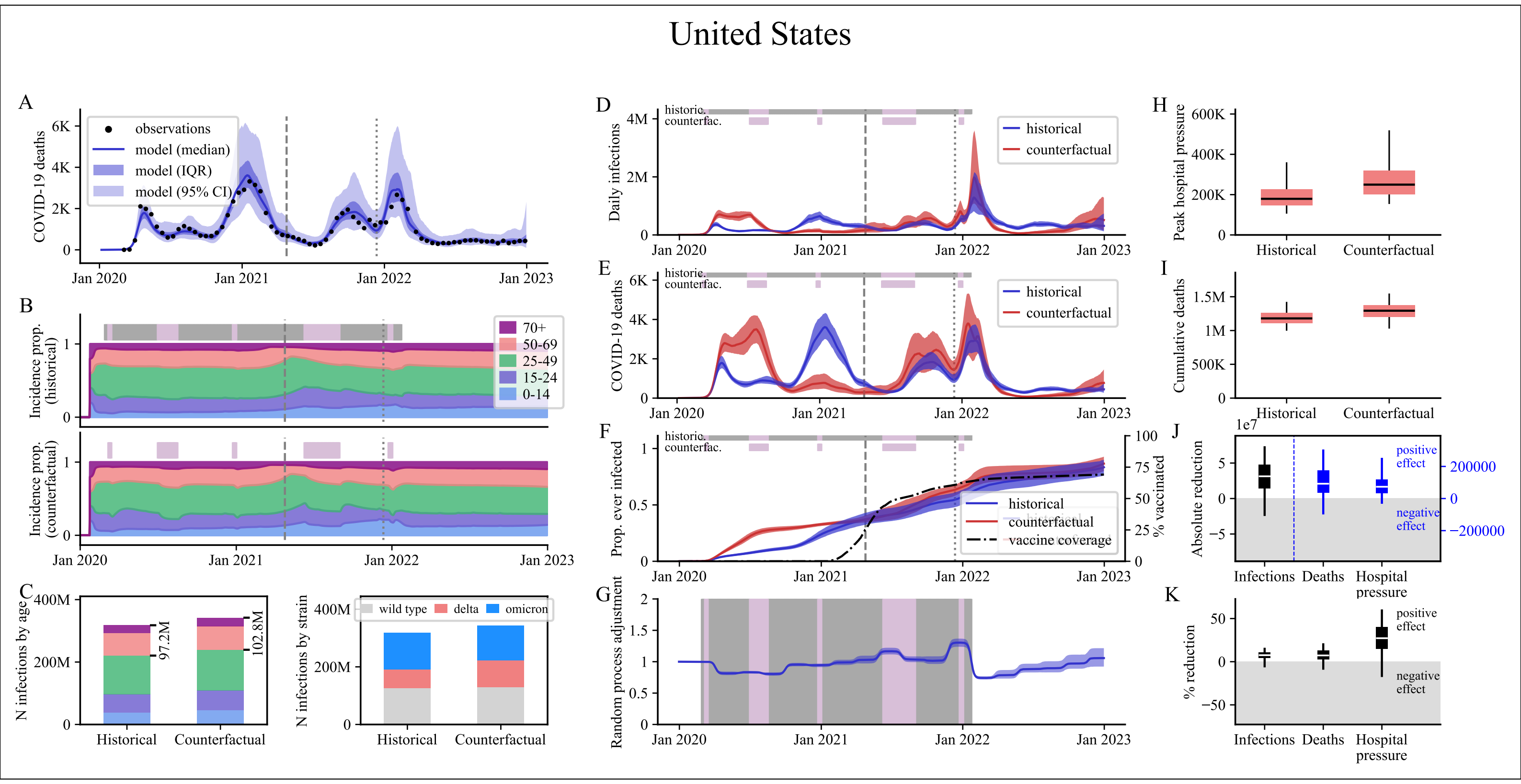
United States
A
COVID-19 deaths
observations
model (median)
model (IQR)
model (95% CI)
Jan 2020
Jan 2021
Jan 2022
Jan 2023
B
Incidence prop. (historical)
Incidence prop. (counterfactual)
70+
50-69
25-49
15-24
0-14
C
N infections by age
97.2M
102.8M
Historical
Counterfactual
N infections by strain
wild type
delta
omicron
D
historic.
counterfac.
Daily infections
historical
counterfactual
E
COVID-19 deaths
F
Prop. ever infected
% vaccinated
vaccine coverage
G
Random process adjustment
H
Peak hospital pressure
I
Cumulative deaths
J
1e7
Absolute reduction
positive effect
negative effect
Infections
Deaths
Hospital pressure
K
% reduction

Venezuela Bolivarian Republic of

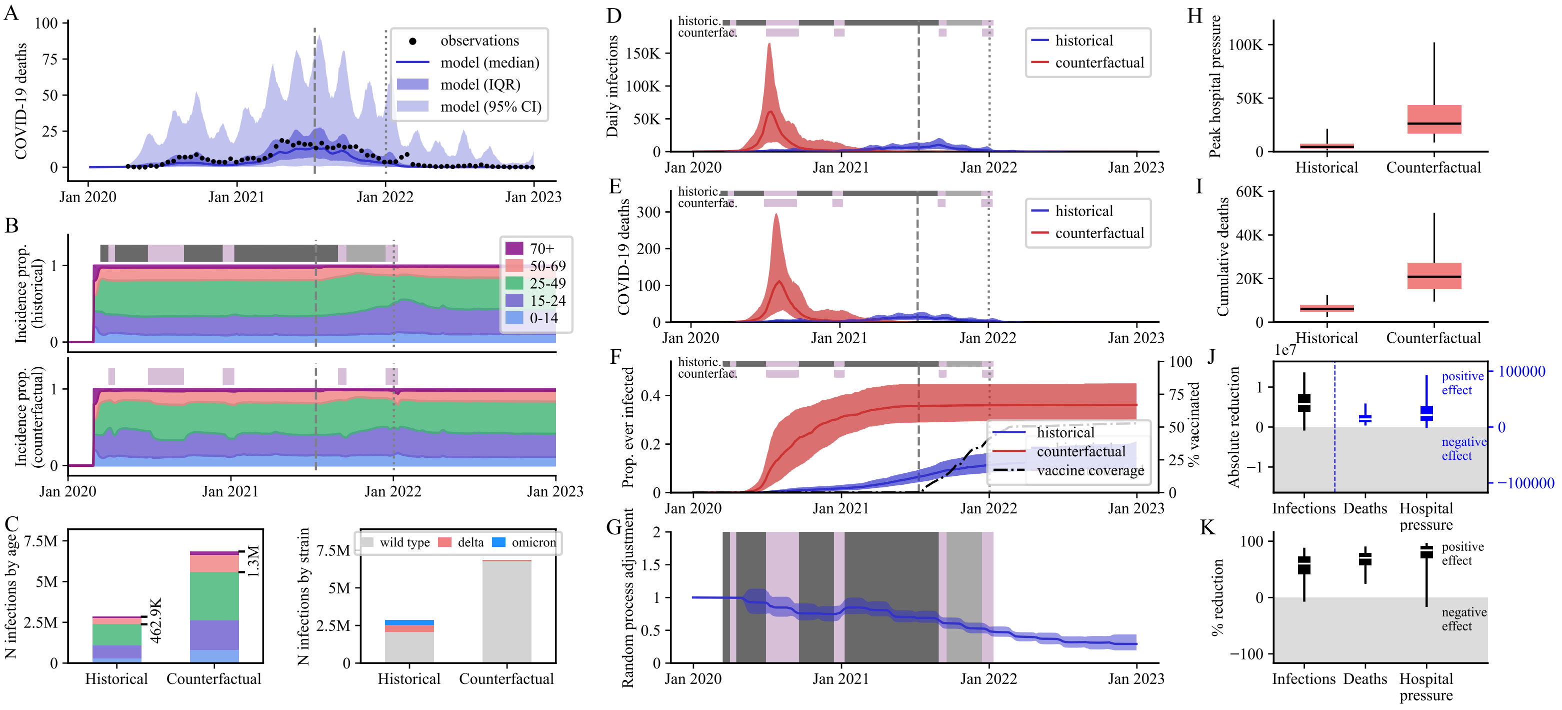

Viet Nam

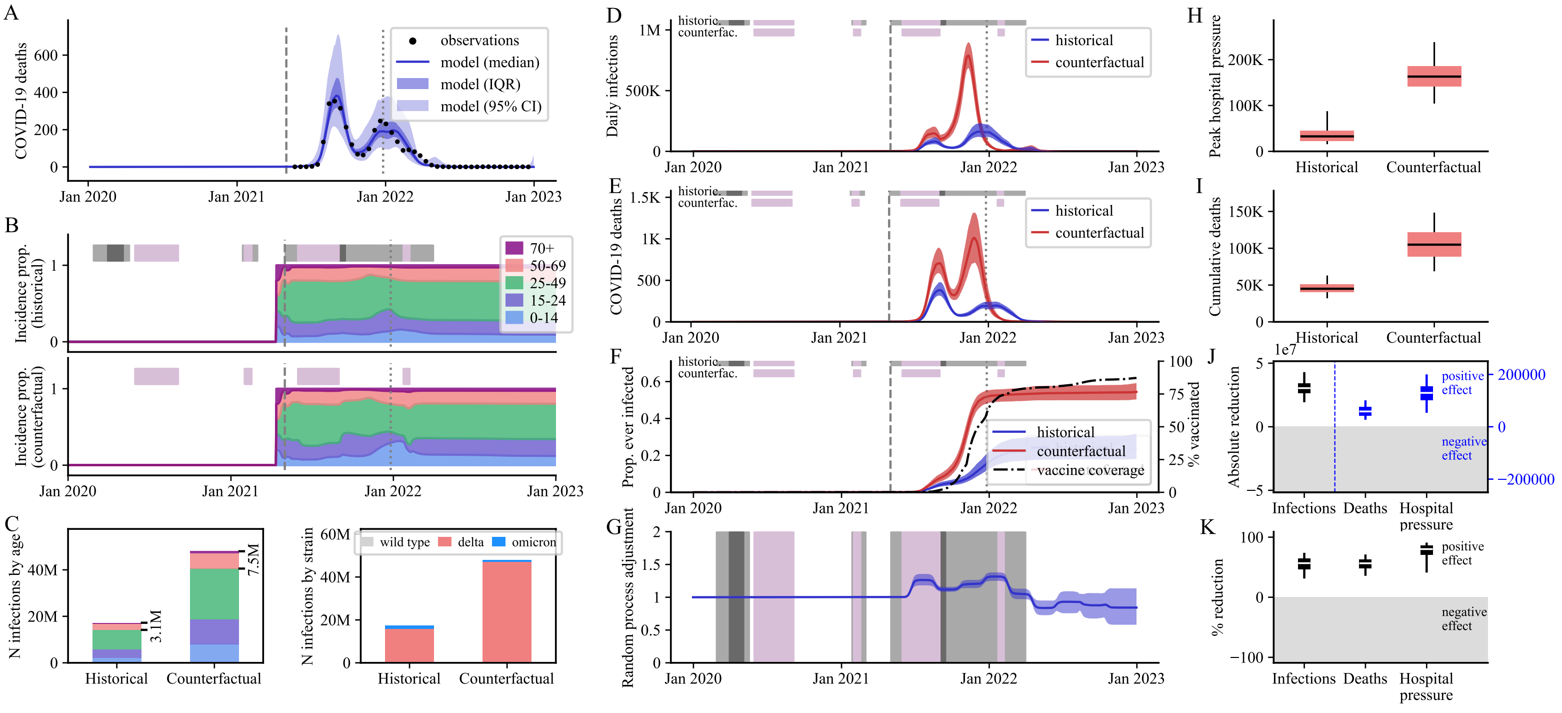

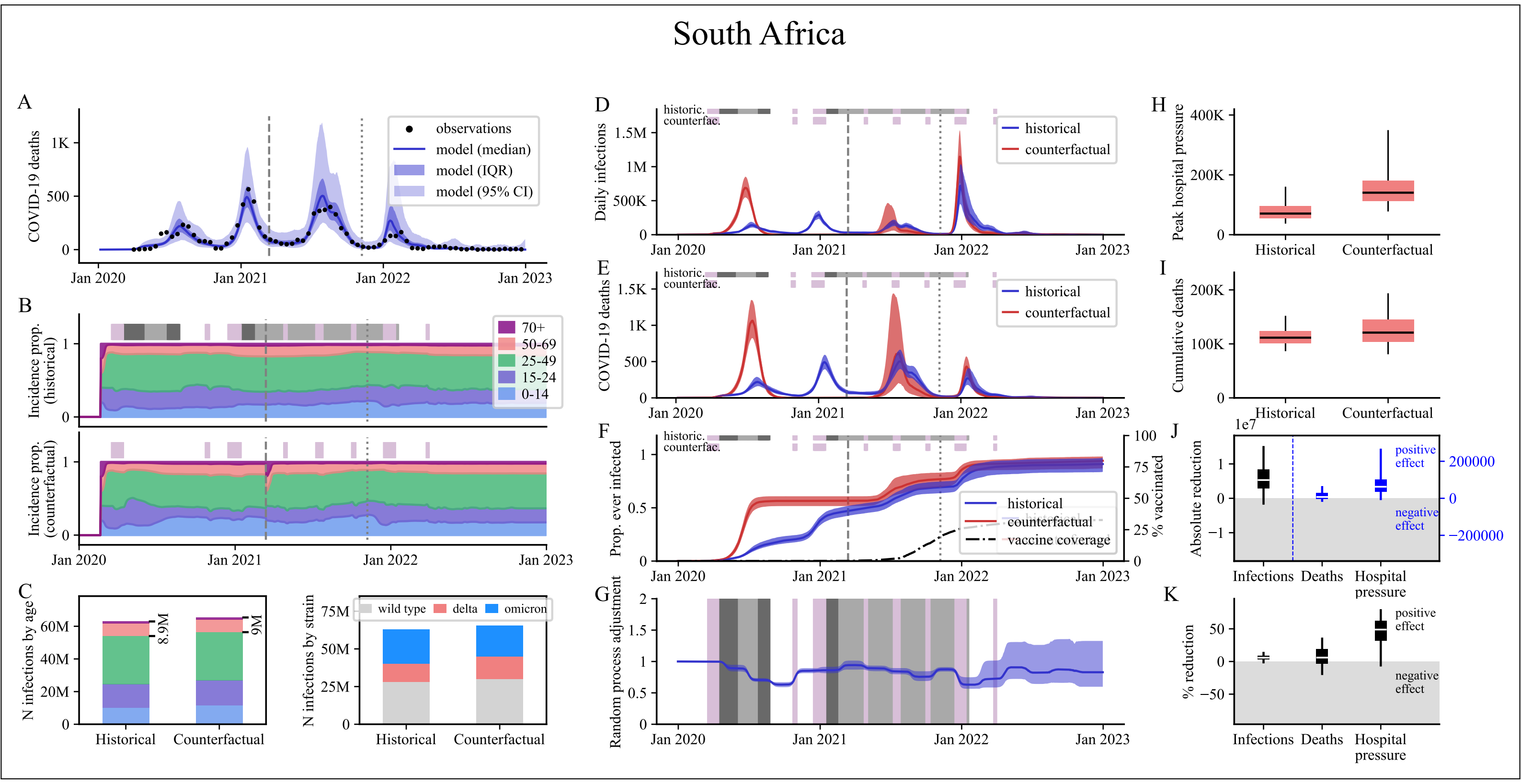
South Africa
A
COVID-19 deaths
observations
model (median)
model (IQR)
model (95% CI)
B
Incidence prop. (historical)
Incidence prop. (counterfactual)
70+
50-69
25-49
15-24
0-14
C
N infections by age
8.9M
9M
N infections by strain
wild type
delta
omicron
Historical
Counterfactual
D
Daily infections
historic.
counterfac.
historical
counterfactual
E
COVID-19 deaths
F
Prop. ever infected
% vaccinated
vaccine coverage
G
Random process adjustment
H
Peak hospital pressure
I
Cumulative deaths
J
Absolute reduction
1e7
positive effect
negative effect
K
% reduction
Infections
Deaths
Hospital pressure
Jan 2020
Jan 2021
Jan 2022
Jan 2023

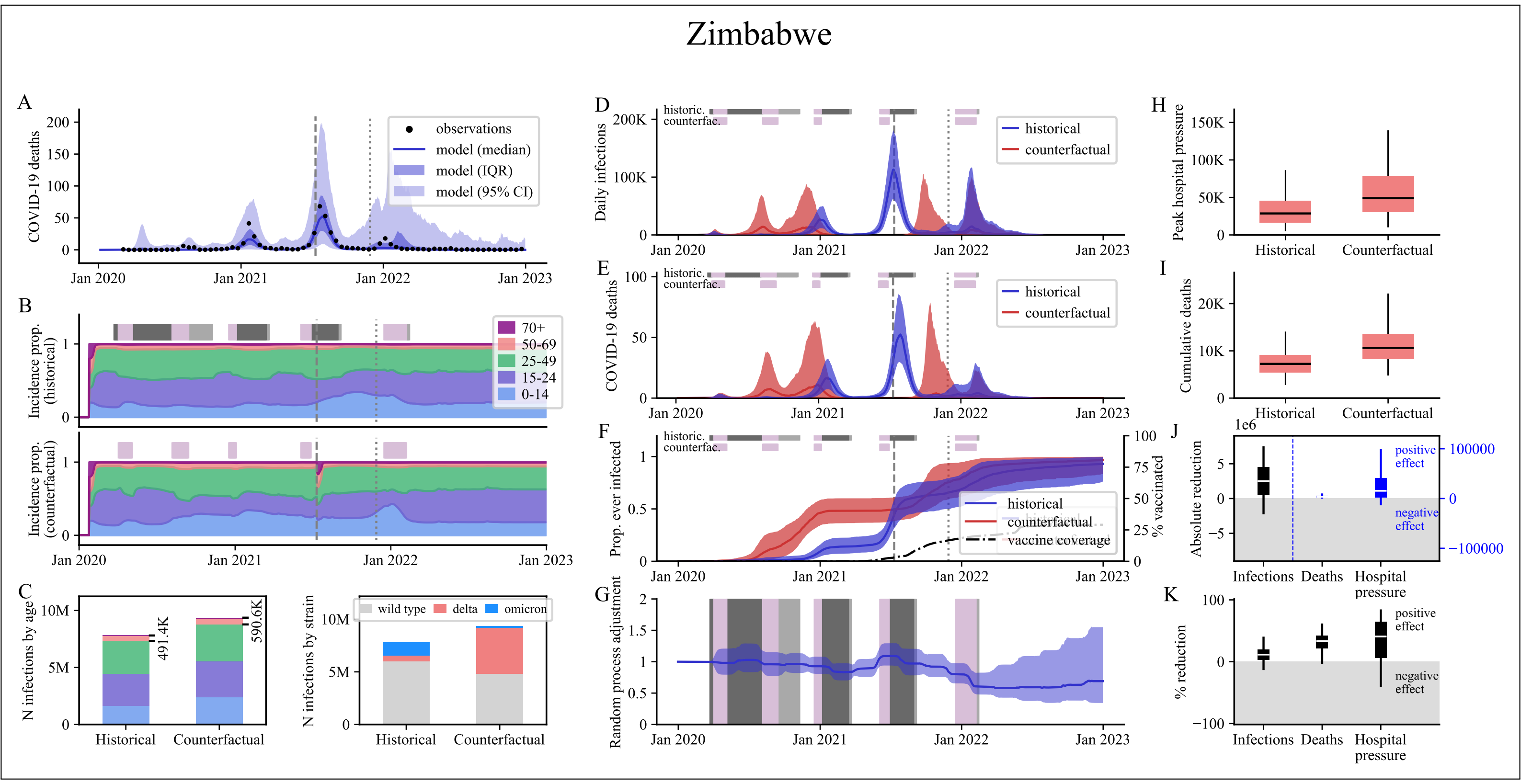
Zimbabwe
A
COVID-19 deaths
observations
model (median)
model (IQR)
model (95% CI)
B
Incidence prop. (historical)
Incidence prop. (counterfactual)
70+
50-69
25-49
15-24
0-14
C
N infections by age
491.4K
590.6K
N infections by strain
wild type
delta
omicron
Historical
Counterfactual
D
Daily infections
historic.
counterfac.
historical
counterfactual
E
COVID-19 deaths
F
Prop. ever infected
% vaccinated
vaccine coverage
G
Random process adjustment
H
Peak hospital pressure
I
Cumulative deaths
J
Absolute reduction
1e6
positive effect
negative effect
K
% reduction
Infections
Deaths
Hospital pressure
Jan 2020
Jan 2021
Jan 2022
Jan 2023
