# Supplementary material for: Estimating the impact of school closures on the COVID-19 dynamics in 74 countries: A modelling analysis
Source: PLoS Med. 2025 Jan 21;22(1):e1004512. doi: 10.1371/journal.pmed.1004512 (PMC11793732; doi:10.1371/journal.pmed.1004512)
Supplement: Attachment — Submitted filename: Comments.docx [file pmed.1004512.s002.docx]

**Responses to reviewers’ comments**

18^th^ September 2024

Dear Editor,

Many thanks for the opportunity to revise our manuscript.

We believe that the changes made in this revision have improved the analysis significantly and we thank you and the reviewers for the careful review and constructive comments.

Please see below for our response to the reviewers’ comments and descriptions of the changes made since the first submission.

Yours sincerely,

Dr Romain Ragonnet

Corresponding author

**Summary of main changes made to the manuscript in response to the Editor’s and Reviewers’ comments:**

- We repeated the entire analysis using alternative contact structures for the 25 countries for which matrices were available, as suggested by Reviewer #3.
- We present our estimates for the adjustment to the risk of transmission over time, which is a critical component of our model as highlighted by Reviewer #2, and compare it to the school closure timelines.
- We performed additional sensitivity analyses considering alternative model structures following a concern raised by Reviewer #4.
- We performed a statistical analysis of our main modelling estimates to explain the heterogeneities observed in the estimated impact of school closures on SARS-CoV-2 infections and COVID-19 deaths.
- We modified some of the main text’s figure captions to make them easier to understand and incorporated estimates of absolute magnitudes of the effects, as suggested by the Editor.
- We edited the main text and supplement to address Reviewers concerns regarding some of our wording and discussion.

Please find our point-by-point responses to comments in the following pages.

**Comments from the Academic Editor**

It is a strong manuscript, with some interesting findings. The comments are helpful, and it seems that the manuscript has potential to be an important addition to the scientific literature about a contentious and under researched issue (effect of school closures).

We thank the Academic Editor for the careful review of our manuscript and the positive comments.

A couple of suggestions:

E.C1) Some figures, particularly 3, 4 and 5, would benefit from simplification and avoidance of trying to present too much data at once.

We have reduced the amount of information presented in Figures 3, 4 and 5 to make them easier for the reader to understand, while retaining the necessary data to communicate and discuss our main findings. The full sets of model outputs are still available in the Supplement for all countries.

E.C2) More emphasis in the discussion on the lack of data from Africa. Having lived and worked in Africa during the COVID-19 pandemic, I am acutely aware that many countries implemented long school closures based on what was happening elsewhere in the world, and probably to the considerable detriment of children's well-being and education. It is a shame that more African countries did not have data to include.

We share the Editor’s frustration regarding the limited number of African countries included in the analysis. In an attempt to increase the representation of the African region, we performed full analyses for five additional countries—Nigeria, Uganda, Cameroon, Angola, and Mozambique—after manually collecting the required data on the timing of variant emergence from alternative sources. Unfortunately, the model failed to produce reliable results for these countries, primarily due to the relatively low number of reported COVID-19 deaths over time, which is the main indicator used for model calibration. This issue, coupled with the lack of seroprevalence data, made it impossible for our model to generate reliable projections. Therefore, we decided not to include these results in the manuscript. Below is an illustration of the results obtained for Cameroon, highlighting the difficulty in achieving a satisfactory fit to the data (panel A) and the enormous amount of uncertainty in the main estimates (e.g., panels H-K).

We have added the following text to the Discussion section:
Lines 415-21 (clean version): “While we aimed to include a comprehensive range of countries in our analysis, certain regions were underrepresented due to data limitations. The small number of included African countries was primarily explained by the lack of data on variants emergence times from the GISAID database. We attempted to include more African countries after manually collecting variants data from alternative sources. Unfortunately, the model did not produce interpretable results due to the relatively low number of reported COVID-19 deaths over time, which was the main indicator used for model calibration, and the lack of seroprevalence data.”

E.C3) I also note that there seemed to be no data available for China to support inclusion – good if the authors can comment on this.

We added the following sentence after the text mentioned in the previous comment:

Lines 421-2 (clean version): “Additionally, we could not provide estimates for China due to the unavailability of Google mobility data.”

E.C4) Although the presentation of relative risks is helpful, I would probably have liked to see absolute numerical magnitudes of the effects on outcomes under counterfactual scenarios. This would be very helpful for public health planning.

We have included the estimates of absolute effects of school closures on infections, deaths and peak hospitalisations in Figures 3, 4 and 5, as well as in all country-specific figures presented in the S1 Appendix.

**Comments from Reviewer #1:**

R1.C0) I commend the authors for such a well-written, novel, and well-conceived manuscript. The topic will be of interest to readers. While other researchers have attempted to clarify the effect of school closures on COVID-19 outcomes, doing so remains of importance and this paper offers a novel approach to this question via 1) inclusion of a remarkable 74 countries in the analysis, and 2) exploration of the effect of school closures over a longer period of time, 2020 to 2022. Additionally, the paper demonstrates some novel findings, that are presented in an intuitive way. First, the authors find that, in some countries, the avoidance of a surge in the wildtype strain from school closures led to lower immunity and thus higher cases and more hospitalizations during the delta wave. Second, the authors demonstrate that school closures in some cases shifted the age distribution of infections to older individuals, resulting in an overall increase in deaths.

Methodologically, the work is sound. The authors have previously published this model in other settings and are highly transparent about model functionality through a well-written supplement and sharing of the code in a public GitHub repo. They add additional components to this model beyond their previously published work via a semi-mechanistic component and model calibration with seroprevalence data and death data.

There are, as to be expected, some limitations of this work. Notably, there is substantial uncertainty surrounding childhood behavior that cannot be captured by the UNESCO database. In the US, where this reviewer is from, the "partial reopening" period was clustered by geographies (e.g., some states opened far sooner than others), and in the "fully reopen" period, many districts offered the choice to parents to continue to keep their children home for remote instruction. Adherence to social distancing was highly heterogeneous at the sub-national level. However, the authors note both these limitations in the discussion and account well for uncertainty, including through sensitivity analyses. Other methodological limitations I observed include the assumption that individuals cannot be infected twice with the same strain, imperfect immunity seems to be constant rather than reflect waning, vaccination patterns (saturating the elderly before moving to younger age groups) may be unrealistic, and hospital pressure is based off values from the Netherlands (although adjusted by country for death rate). Nevertheless, I find these to be reasonable assumptions to make within an already sophisticated and nuanced model framework.

In sum, it was a delight to review this manuscript and am pleased to have no substantial comments on this work. I can only offer small suggestions and observations:

We thank Reviewer #1 for the extremely positive and relevant feedback.

R1.C1)	Because the time period of the study was only introduced once in the intro, it would be helpful to add within results and figure legends that the estimated relative reductions are over the period January 1, 2020 to December 31, 2022.

We now specify the exact period over which the relative reductions are calculated in the opening sentence of the Results section and in all figure legends.

Lines 227-8 (clean version): “Based on our median estimates of intervention effects over the period 1 January 2020 to 31 December 2022, school closures were associated with a beneficial effect on […]”

R1.C2)	I noted that the Github links on page 15 of the supplement did not work for me. I was able to access what I believe are the correct links, with the difference in url's being "master" vs. "main"

Thank you for identifying this issue. We have now updated the Github URLs.

R1.C3)	I spent a lot of time trying to interpret the vaccination-related terms in A.5 equations in the supplement. First, I could not find the definition of phi because it came after the equations. Then, I saw the definition and understood phi, but continued to have trouble with its interpretation, including for the subscript v=0, as in S_v=0. Perhaps it may help readers to have phi introduced in the text above the equations with an additional sentence surrounding the product of phi with w(t) and the state variable conditioned on v=0.

We recognise that the previous approach to describing the vaccination transitions was confusing and we now describe the notation $\Phi_{v}$ clearly before introducing the ordinary differential equations:

**Comments from Reviewer #2:**

R2.C0) Authors of this paper analyze a mass of data collected during the COVID-19 pandemic describing the epidemic dynamics, vaccination coverage, dynamics of variants, mobility, timing and nature of school closures in 71 countries to ascertain the impact of school closure (as measured by the proportion of cases, hospitalizations and deaths averted thanks to the intervention) during the pandemic. They find that in most countries school closure had a beneficial impact but that in a few countries, counterfactual scenarios without school closure led to smaller epidemic burden.

I find that this is an interesting attempt to quantify the impact of school closure, looking at data from a large number of countries. However, I have major comments about the methods, the results, their interpretation and validity.

We thank Reviewer #2 for the careful review and relevant comments that contributed to improving the quality of our manuscript.

R2.C1) Is school closure impact inferred from the data or is it mostly a byproduct of modelling assumptions and model structure? In general, in studies that aim to evaluate the impact of interventions on transmission, the transmission rate is modelled as a function of the interventions being implemented at a given time, for example the modelled transmission rate is reduced by a factor beta_school when schools are closed. The impact of school closure is then measured by estimating from the data parameter beta_school. What puzzles me with this work is that there does not seem to be parameters that are estimated from the data to quantify the impact of school closure. The only two I could find are the "uncertainty multiplier for school contact" (between 0.8 and 1.2 so always close to 1) and the "proportion of students on site during partial opening". As a result, it appears that estimates of school closure impact are largely driven by modelling assumptions, specifically equation A.3 in the Supplement describing the structure of the contact matrix that includes a matrix for schools.

So, I expect that assumptions about the contact matrices and how they changed over time during the pandemic are driving the results.

a. I'm not familiar with the package conmat that was used to generate these matrices, put presumably for a relatively large number of countries, no contact data are available and the contact matrix is reconstructed from data available in countries with similar profiles, which might be particularly problematic in countries with atypical demographic and cultural structures.

b. Properly characterizing how these contact matrices changed over time during the pandemic is a major challenge and there is a serious risk of model misspecification here. I didn't find it very clear how authors performed this task. I understand this was done through google mobility; but authors need to provide more details. An important risk with the approach used is that results may be wrong if the model is misspecified (which is a serious possibility). The problem is exacerbated by the fact that there is currently no way to ascertain model adequacy (see next point). I think that this represents an important limitation of the work. For readers, it is essential to clarify what is being done here, i.e. it is not about estimating the instantaneous impact of school closure on transmission, rather, under a setting of assumptions about contact matrices and the instantaneous impact of school closure on transmission, it is to derive overall impact of school closure. Discussion needs to be extended to reflect the fact that results are highly dependent on model assumptions, in particular the modelling of the contact matrices and the important risk of misspecification, particularly in the pandemic context.

We appreciate Reviewer #2's insightful comments and would like to address the concerns raised about the assumptions and limitations of our model, particularly regarding our approach to modelling school closures.

Approach to capturing school closures:

As noted by Reviewer #2, we capture school closures by removing the school components from the contact matrices (Equation A.3). We believe this mechanistic approach is appropriate for several reasons:

- it provides a natural and intuitive way to model school closures,

- it facilitates sensitivity analyses to test alternative social mixing assumptions,

- it allows for the incorporation of uncertainty around key aspects of modelled social mixing, as illustrated by the uncertainty multiplier for school contacts and the variable proportion of students on-site during partial openings,

- it is consistent with multiple COVID-19 intervention modelling studies, allowing for comparison across studies.

The approach suggested by Reviewer #2 consists in calibrating transmission reductions during school closures using data. However, our approach similarly involves the calibration of a time-variant transmission adjustment (referred to as “random process”) which we expect to capture any transmission changes not already covered by the mechanisms explicitly included in our model. As such, if our matrix-based approach failed to adequately capture the effects of school closures (model mis-specification), we would expect to observe a significant step change in the calibrated random process around the times of school closures or reopening. We have now undertaken further analysis and did not observe such phenomenon, which suggests that our approach adequately captured transmission changes induced by school closures.

We acknowledge that the concern raised by Reviewer #2 is of significant importance and that more clarity was required around these methodological considerations. We now present the estimated random process profile for each analysed country alongside the school closure timelines to offer the reader the opportunity to explore the model dynamics in more details (e.g. Figures 3-5). We also added the following paragraph in the Results section:

Lines 274-82 (clean version): “The estimated transmission adjustment (random process) over time is presented for the three highlighted countries in Panel D of Figures 3-5, and for all analysed countries in S1 Appendix. Our model consistently captured local epidemic dynamics without requiring abrupt changes in the random process. Notably, we found no systematic shifts coinciding with school closure or reopening times, suggesting that the transmission changes induced by school closures were adequately captured by our approach based on mixing matrix modifications. However, we observed a gradual decline in the random process in some countries (e.g. Morocco, Figure 3), suggesting the presence of factors not explicitly captured by the model that may have contributed to reduced transmission over time (e.g. increased awareness, improved hygiene).”

Finally, we have further clarified our approach to capturing school closures and how it differs from previous works in the Discussion section:

Lines 364-75: “Previous studies investigating the epidemiological impact of school closures on COVID-19 mostly focused on the instantaneous effects on transmission through estimations of reproduction number or growth rate. […] Our approach differs fundamentally from previous works from at least two aspects. First, instead of trying to estimate the changes in reproduction number induced by school closures, we captured transmission changes explicitly by modifying the school component of country-specific mixing matrices over time. Second, we considered the comprehensive impact of school closures over prolonged periods spanning multiple epidemic waves. […]”

Decision to use extrapolated contact matrices and not only contact survey data:

Reviewer #2 is correct in pointing out that our approach relies on contact matrix assumptions, which are at the core of our model. It is also correct that social contact data are unavailable for most countries, and we used extrapolated matrices based on available data. While we acknowledge the challenges this presents, we aimed to include as many countries as possible to provide a global perspective on the effects of school closures. We believe restricting the analysis to countries with available contact data would have reduced the broader applicability of our findings, and excluded countries that have historically lacked modelling-based epidemiological insights.

Decision to use of the *conmat* package:

We selected the *conmat* package for our contact matrix estimates as it offers extensive country coverage and employs a simple, transparent, and fully reproducible statistical approach for extrapolation. To the best of our knowledge, the only available alternatives are the matrices from Prem et al. (PLoS Comput Biol 2021) and Mistry et al. (Nat Commun 2021). We previously identified significant errors in Prem et al.'s methodology, such that we do not consider them to be valid (see [*Review of methods used to create synthetic matrices (Prem et al.)*, Ragonnet et al., Figshare 2022](https://figshare.com/articles/online_resource/Review_of_methods_used_to_create_synthetic_matrices_Prem_et_al_/21052918?file=37361773)). Mistry et al.'s approach, while thorough, relies on numerous assumptions that are difficult to verify and provides more limited coverage compared to *conmat*. In this revision, we performed a sensitivity analysis comparing Mistry et al.'s estimates with our base case results using *conmat* and identified no significant variation in the findings (Sensitivity Analysis SA3).

R2.C2) Model adequacy: When a simple mechanistic model (for example with 1 transmission rate when schools are open and 1 when schools are closed) presents a good fit to the epidemic time series, this offers assurances that the school closure model captures key features in the data. Here, although fits to country data are almost systematically good, such good fits are not necessarily indicative that the impact of school closures is well captured by the model. This is because other interventions are modeled with a very flexible autocorrelated process. With this approach, any mismatch between the school closure model and the data will be compensated by the autocorrelated process so that the overall fit will be good. This is problematic because it means we have no way to assess if modelling assumptions about school closure are supported by the data.

The model is only calibrated to death counts and serological data. Overall death are very aggregated data that are unlikely to provide the granular insight necessary to capture the impact of an intervention such as school closure targeting kids.

It seems important that authors manage to identify a signature in the data for the impact of school closure (e.g. a change in the age structure of cases, a slowing down of growth in children) and that they show that their model can reproduce this signature and that a model without school closure could not reproduce this signature. I think this is difficult to achieve in a context where a lot of other interventions were implemented.

It would also be useful to be able to compare impact derived on instantaneous R in different countries to assess consistency between estimate and see if these estimates make sense.

We agree with the point raised by Reviewer #2 and we believe it has been addressed in our response to the previous comment (R2.C1). In particular, the potential compensation phenomenon mentioned by Reviewer #2 was not observed at the times of school closure or reopening, as we have now illustrated. We thank the reviewer for this comment, which highlighted the importance of providing more detailed information about the calibrated time-variant random process. This has now been done and we believe the additional results provide confidence about the robustness of our assumptions.

We chose to use COVID-19 deaths as calibration data because we consider it a more reliable and consistent indicator compared to reported cases. Reported cases can be heavily influenced by case detection policies, which have varied significantly over time. We agree with Reviewer #2 that using age-specific case data may have the potential to further refine estimates of school closure effects. However, such data is unavailable for most countries, making it challenging to include in an analysis that we wanted to be as systematic as possible. Furthermore, we tracked and reported the estimated age-distribution of infections over time for all analysed countries (see Supplement, Section B.3). Although we observed changes in the age profiles of infections during school closures, these changes are unlikely to be large enough to improve parameter inference. Moreover, the variability in case detection and the likely differential detection rates by age would further complicate the calibration process, making it unfeasible without introducing significant additional assumptions.

Finally, although we did not track the instantaneous reproduction number (R), the relative stability of the estimated random process during school closures indicates that changes in the reproduction number were effectively captured through adjustments to the contact matrix. This approach was consistently applied across all included countries.

R2.C3) Interpretation of results: Authors provide for each country the estimated number of cases, deaths and hospitalizations at the peak averted thanks to school closure. For example number of cases averted due to school closure go from more than 50% in Venezuela to slightly negative value for Great Britain. Currently, there is no much mechanistic insight about why we get these differences. Authors explain a bit why in some cases the impact might have been negative (because of an absence of build up of immunity in children); but this does not explain the huge heterogeneity observed between countries (and why this phenomenon only occurred in a very small subset of countries). Without good understanding of the drivers of this heterogeneity, it is very hard to ascertain whether the results across the different countries are consistent with each other or simply reflect a model that is overparametrized and produce inconsistent results from one country to the next. It is therefore essential that authors manage to better explain what drives heterogeneity in country specific estimates:

a. The duration of closure is a first natural driver.

b. Most importantly, I would hypothesize that the impact of school closure may largely depend on the intensity of other interventions. For example, in a country where the rest of the population is in lockdown, closing schools might have only limited additional impact. In contrast, if mixing patterns in other environments are close to normal, school closure may have a more important contribution to parameter.

c. It might also be interesting to explore how other types of school based interventions might affect results. For example, in some countries, school closure was limited but there were a lot of other school based interventions, e.g. masks wearing, repeated testing etc. Do school closures remain relevant in this context?

d. Could the population structure affect impact? For example with bigger impact in younger populations?

We thank Reviewer #2 for the excellent suggestions provided in this comment. We have now performed a post hoc statistical analysis of our main findings to investigate potential sources of heterogeneity. This analysis is fully detailed in the Supplement (Section B.1: Exploring the findings heterogeneity) and its main results are reported in the main text as follows:

Results section, Lines 301-8: “To investigate potential sources of heterogeneity in the effects of school closures on COVID-19 dynamics, we conducted a post-hoc analysis examining the relationships between our findings and key population characteristics (see S1 Appendix Section B.1). This analysis revealed a negative association between the proportion of individuals aged 70 and older and the effectiveness of school closures in reducing infections and deaths. Additionally, the analysis suggested that school closures may have been more beneficial in settings where less stringent PHSMs other than school closures were in place during the closure periods, although this association did not reach statistical significance.”

R2.C4) Emphasis on the negative impact of school closure: There is currently an important emphasis in the abstract, results and discussion on the potentially negative impact of school closure. However, I don't find it convincing at all. Just looking at figure 1, there is a first set of countries where school closure clearly had a positive impact; and then a second set where impact was negligeable. There are just 1-3 countries out of 71 where estimates suggest a slightly borderline negative effect so I would really question the strength of evidence on this negative effect. Yet, this weak finding is strongly emphasized and takes a big part of abstract/results/discussion.

We have revised the manuscript throughout to address this issue, ensuring that the text now more accurately reflects the estimates.

As indicated in the previous point, it would be much better to attempt to explain heterogeneity in estimates rather than spending a large part of paper explaining patterns in 1-3 countrie out of 71.

Please see response to comment R2.C3.

Other comments:

R2.C5) Line 69 : "We introduced differential levels of transmissibility, hospitalisation risk and death risk by strain. We assumed that pre-existing immunity (both infection- and vaccine-induced) was only partially protective against the Delta and Omicron strains." Please comment on assumption made and data supporting them (a reference to Supplement is fine).

We have added the following sentence to the Methods section:

Lines 137-8: “The assumptions and sources of evidence used to inform strain-specific parameters are detailed in the Supplementary appendix (Section A.1.4.6).”

R2.C6) High autocorrelation in random effect to "avoid unrealistic changes over a short period of time". But in a lot of countries, very important changes occurred over short periods of time, for example at the start of lockdowns in the first wave in 2020. The autocorrelation might lead to underestimating the impact of the lockdown and overestimating that of school closure (for which sudden nature of change is presumably well captured by model.

We expect shifts in transmission associated with significant COVID-19 policy changes to be reflected in the Google Mobility trends, which directly inform our model by time-variant scaling of the different mixing matrix components. Therefore, we do not rely solely on the random process to capture such abrupt changes.

Notably, we conducted a sensitivity analysis (SA2) in which we removed the contribution of Google Mobility data from the modelled social mixing. In this analysis, we found that the time-variant random process, which accounts for transmission changes not explicitly captured by model inputs, required less variability when mobility data was incorporated. This suggests that integrating Google Mobility data improved the model's ability to capture the impact of abrupt policy changes, such as lockdowns.

Results section Lines 287-94: “The two configurations in which Google Mobility data were used to inform the modelled dynamics (Base-case and SA1) were associated with higher a posteriori likelihood values in most countries compared to the configuration where these data were excluded (SA2), suggesting that incorporating Google Mobility data helped achieve more realistic model fits (Supplementary Figure S23). In particular, we found that the time-variant random process, which reflects transmission changes not explicitly captured by the model inputs, required less variability under the configurations where mobility data was incorporated in multiple countries (Supplementary Figure S24).”

**Comments from Reviewer #3:**

R3.C0) This manuscript presents a mathematical model used to evaluate the impact of school closure policies on the dynamics of the COVID-19 pandemic in 2020-2022 in 74 countries. The authors claim that school closures generally reduced the COVID-19 burden but had a negative impact in some countries (USA and Europe), probably attributable to a shift in the age distribution of cases.

The article is well written and organized in general, and I appreciate that the authors made the code and data used for their analyses available on Github.

The topic is interesting as the role of school closures in contrasting an epidemic is still not completely clear and deserves thorough investigation through a modeling approach; advances in the knowledge of the advantages and drawbacks of this non-pharmaceutical policy would surely have an impact on public health decisions.

However, I have some perplexity on the presented modeling choices, and I also believe that the findings of this work would not represent a considerable contribution to the topic. This considered, I would not recommend this work for publication into PLOS Medicine; I would suggest considering PLOS ONE after revision of this manuscript.

We thank Reviewer #3 for the thorough review and the constructive comments. We have addressed the methodological concerns in the responses that follow.

Major comments

R3.C1) The transmission model implemented by the authors is quite complex and to a certain extent unconventional:

- two exposed compartments are partially infectious, but they are not considered as infectious compartments

- the random perturbation of the transmission risk, W(t), is non-standard

- the nomenclature used for the compartments is unusual ("actively diseased" instead of "infectious")

The authors should provide additional information to justify their modeling choices and support them by showing in the Supplementary material their appropriateness and the improvements achieved as compared to a standard compartmental model. Lacking this, it is difficult to understand whether these results would be confirmed by a standard, well-established model.

The two exposed compartments that are partially infectious are modelled as contributing to the force of infection. This property is described in Supplementary Section A.1.2.1 and in the force of infection equation (Supplement, Equation A.6). This assumption is now explained in more detail for clarity in the Methods section, along with supporting evidence:

Lines 123-8 (clean version): “We used four Exposed compartments and four Active Disease compartments arranged in series to achieve realistic time distributions for the periods spent in each of these states, with alternative assumptions regarding the number of serial compartments explored in sensitivity analyses (S1 Appendix Section B.1.6). All Active Disease compartments were assumed to be infectious, while the last two Exposed compartments were considered only partially infectious [17, 18].”

Our choice to refer to the “I” compartments as “Active Disease” compartments rather than “Infectious” compartment was a direct consequence of the fact that two of the exposed compartments are also infectious, such that we adapted our terminology in order to avoid any confusion.

In this revision, we performed additional analyses considering alternative assumptions to model both the exposed and the active disease periods. These explorations revealed no noticeable changes in the conclusions, providing confidence in the robustness of our modelling approach. We added the following text to the Results section:

Lines 297-300: “Similarly, varying the number of compartments used to represent the incubation and active disease periods produced model outputs that were virtually identical to those of the base-case analyses (Supplementary Figures S19-21).”

The random perturbation of the transmission rate similar to our random process W(t) is commonly employed in semi-mechanistic modelling frameworks. Notable examples include reference works from Camacho et al. (PLoS Curr. 2015, doi: 10.1371/currents.outbreaks.406ae55e83ec0b5193e30856b9235ed2), Funk et al. (Epidemics 2018, doi.org/10.1016/j.epidem.2016.11.003), and Flaxman et al. (Nature 2020, doi.org/10.1038/s41586-020-2405-7)

PMCID: PMC4339317). The inclusion of the random process W(t) was also inspired by a COVID-19 modelling study from Watson et al. investigating the impact of vaccination around the world (Lancet ID 2022, doi.org/10.1016/S1473-3099(22)00320-6).

In our revised manuscript, we present the estimated time-variant random process for all countries analysed to provide more details about the internal dynamics of our model.

Results section Lines 274-5: “The estimated transmission adjustment (random process) over time is presented for the three highlighted countries in Panel D of Figures 3-5, and for all analysed countries in S1 Appendix.”

R3.C2) The authors state "We further assume that the infectious exposed compartments (E3 and E4) are half as infectious as the active disease compartments" without motivating this choice. Is there a supporting reference?

We thank Reviewer #3 for identifying the issue and we have now included supporting references both in the main text and in the Supplementary Appendix, as well as adding a section describing the evidence in the Supplementary Appendix:

R3.C3) Active disease compartments are considered all equally infectious, but infectivity is not constant throughout the infectious period of an individual.

We acknowledge that infectiousness is almost certainly not uniform over an individual’s infectious period. This has been particularly demonstrated from studies of infectious viral shedding in infected individuals (e.g. Hakki et al., Lancet Respir Med 2022). However, it is challenging to directly correlate the distribution of viral shedding with epidemiological measures of infectiousness, such as attack rates. To maintain model simplicity, we assumed a constant average level of infectiousness throughout the active disease period.

Importantly, our newly added sensitivity analysis, which explored alternative assumptions for both the incubation and active disease periods (in response to R3.C1), showed no significant impact on our model outputs. Therefore, we believe this issue does not affect our overall findings.

R3.C4) Since the authors report different impact of school closures on infections/hospitalizations/deaths for different areas of the world, I believe that this study would benefit from the inclusion of details on the possible socio-demographic factors that may be involved: correlation with age structure of the population, fraction of active individuals (i.e. students or belonging to the workforce), etc.

We thank Reviewer #3 for this highly relevant comment, which highlight a similar concern as in one of Reviewer #2’s comments (R2.C3). We have now performed a post hoc statistical analysis of our main findings to investigate potential sources of heterogeneity. This analysis is fully detailed in the Supplement (Section B.1: Exploring the findings heterogeneity) and its main results are reported in the main text as follows:

Results section Lines 301-8: “To investigate potential sources of heterogeneity in the effects of school closures on COVID-19 dynamics, we conducted a post-hoc analysis examining the relationships between our findings and key population characteristics (see S1 Appendix Section B.1). This analysis revealed a negative association between the proportion of individuals aged 70 and older and the effectiveness of school closures in reducing infections and deaths. Additionally, the analysis suggested that school closures may have been more beneficial in settings where less stringent PHSMs other than school closures were in place during the closure periods, although this association did not reach statistical significance.”

R3.C5) The authors observe that, for some countries, school closure may have caused an overall worsening of COVID-19 mortality by contributing to the emergence of the Delta wave. It would be interesting to include a second counterfactual scenario implementing a reactive school closure policy where closure is based on real-time monitoring of some epidemiological indicators (e.g. hospital occupancy, or case incidence in the country). I would expect reactive closure to be more effective in preventing large waves and more feasible in terms of cumulative policy duration. I would at least recommend including a comment on the possible better outcome of reactive policies.

This is an important point, and we agree that it warrants further discussion in our manuscript. One of the key challenges with reactive strategies is determining the appropriate triggers for policy implementation, which can vary greatly depending on the setting. As Reviewer #3 suggested, hospital occupancy could serve as a useful indicator for such policies. However, defining a threshold at which the policy should be activated would depend heavily on local hospital capacity and other contextual factors. While conducting an analysis comparing multiple reactive policy scenarios would offer valuable insights, we believe it would be more suitable to focus on specific settings or a few countries where we have a more detailed understanding of local capacities. We have added the following text to the Discussion section:

Lines 355-60: “In many instances, governments have employed school closures as a reactive measure in response to escalating epidemics or localised outbreaks. However, there remains considerable uncertainty regarding the optimal trigger for implementing such policies, whether based on hospital capacity thresholds, case numbers, or other indicators. While we did not evaluate specific triggers, future research could explore various reactive strategies, comparing different indicators and threshold levels to better inform decision-making.”

R3C6) Synthetic contact matrices specifically derived from highly detailed, country-specific data for many of the countries covered by this study exist (see for instance Mistry et al. 2021). As the matrices contained in the conmat package, as far as I understand, are not directly generated for the single countries, I am wondering whether results would be different when using contact patterns directly obtained from the socio-demographic structure of the population considered, rather than using generalized contact patterns.

We thank Reviewer #3 for this comment and agree that it is important to explore alternative social mixing assumptions. We selected the *conmat* package for our contact matrix estimates as it offers extensive country coverage and employs a simple, transparent, and fully reproducible statistical approach for extrapolation. To the best of our knowledge, the only available alternatives are the matrices from Prem et al. (PLoS Comput Biol 2021) and Mistry et al. (Nat Commun 2021). We previously identified significant errors in Prem et al.'s methodology, which leads us to believe the resulting estimates should no longer be used ([see *Review of methods used to create synthetic matrices (Prem et al.)*, Ragonnet et al., Figshare 2022](https://figshare.com/articles/online_resource/Review_of_methods_used_to_create_synthetic_matrices_Prem_et_al_/21052918?file=37361773)). Mistry et al.'s approach, while thorough, relies on numerous assumptions that are difficult to verify and provides more limited coverage compared to *conmat*. In this revision, we performed a sensitivity analysis comparing Mistry et al.'s estimates with our base case results using *conmat* and identified no significant variation in the findings (Sensitivity Analysis SA3).

The following text was also added to the main text:

Methods section Lines 220-2: “Finally, our sensitivity analyses considering alternative contact matrices sourced from Mistry et al. (termed SA3) further explored the sensitivity of our findings to changes to social mixing parameterisation.”

Results section Lines 295-7: “Using an alternative source of contact matrix estimates (SA3) did not lead to significant changes in the estimated school closure effects in the 25 countries where we were able to conduct this sensitivity analysis (Supplementary Figures S18-21).”

Discussion section Lines 404-6: “We also tested alternative contact matrices in sensitivity analyses, which resulted in only marginal changes to our estimates.”

Minor comments

R3C7) It would be interesting to expand the discussion on the finding that longer cumulative closure duration did not necessarily lead to greater reduction in COVID-19 mortality: for instance, reactive school closure probably would have performed better, and I believe this should be at least acknowledged, if not investigated with the model.

We added the following sentence in the Discussion section:

Lines 360-3: “Our finding that longer school closure durations did not consistently lead to better epidemiological outcomes suggests that carefully designed strategic policy approaches may potentially achieve more favourable results with less stringent restrictions.”

R3C8) I think it would be important to offer the reader a wider perspective on the existing literature on school closure effectiveness by including more references to previous research. There are many modeling works especially on pandemic influenza but also on COVID-19 that could make a good starting point: see for instance Cauchemez et al. 2008, Wu et al. 2010, Fumanelli et al. 2012, Di Domenico et al. 2021, Rozhnova et al. 2021 only to mention some of them.

We have expanded our discussion of previous works including more references and clarified how they differ from our present study.

Discussion section Lines 364-75: “Previous studies investigating the epidemiological impact of school closures on COVID-19 mostly focused on the instantaneous effects on transmission through estimations of reproduction number or growth rate. Most studies focused on specific countries such as Canada [26], France [27], Germany [25], and China [28], or applied conceptual models to theoretical settings [29]. Despite the variation in scope and settings, the collective evidence indicates that school closures tend to reduce SARS-CoV-2 transmission [27, 28, 30-32], which is consistent with our findings regarding the short-term effects of the intervention. Our approach differs fundamentally from previous works from at least two aspects. First, instead of trying to estimate the changes in reproduction number induced by school closures, we captured transmission changes explicitly by modifying the school component of country-specific mixing matrices over time. Second, we considered the comprehensive impact of school closures over prolonged periods spanning multiple epidemic waves.”

**Comments from Reviewer #4:**

R4.C1) The semi-mechanistic compartmental model used to simulate COVID-19 transmission is well-structured, with appropriate compartment types. The stratification by age, vaccination status, and virus strain is a robust approach for capturing the dynamics of the pandemic. However, it would be beneficial to consider whether additional stratifications, such as by geographic region within countries, could further enhance the model's granularity.

We thank Reviewer #4 for the positive feedback and thorough review of our manuscript. As the reviewer noted, our model already incorporates several key stratifications, though it does not include geographical stratification within countries. Implementing such a feature would require subnational data across multiple factors, including timelines of school closures and the data used for model calibration. Unfortunately, this level of granularity is not publicly available for most countries, and manually requesting such data from a large number of nations would be impractical. To ensure the inclusivity of our study and maintain a consistent methodological approach across all countries, we opted to work at the national level.

However, we recognise that enhancing geographic granularity may have had benefits for this analysis and we included the following text in the Discussion section:

Lines 425–29 (clean version): “Subnational analyses would offer more granularity, considering that school closures often occurred non-uniformly within countries. However, the lack of publicly available subnational data for key factors, such as school closure timelines and model calibration inputs, made it unfeasible to incorporate this level of detail in our analysis across numerous countries.”

R4.C2) The compartmental model is stratified by age and vaccination status, but it is important to assess whether the chosen age group stratification is appropriate and whether the assumptions regarding vaccination and pre-existing immunity are reasonable. Additionally, are the differential transmissibility and risks associated with each viral variant (wild-type, Delta, Omicron) sufficiently justified?

Our assumptions around the relative transmissibility, immune escape properties and relative risk of severe outcomes for the modelled viral variants is documented extensively in section A.1.4 of the Supplemental Appendix. We have now added a sentence in the main text’s Methods section to point to the relevant section:

Lines 137-8: “The assumptions and sources of evidence used to inform strain-specific parameters are detailed in the S1 Appendix (Section A.1.4.6).”

Regarding the choice of modelled age groups, we extended our literature review and we did not identify any further evidence of epidemiological important factors that differed across the age groups that were not modelled.

R4.C3) The authors simulate a counterfactual scenario in which schools remained open. It would be helpful if the authors provide detailed information on the assumptions underlying this counterfactual scenario and clearly explained the differences between historical and counterfactual scenarios.

We have edited the Methods section to provide more details about our approach:

Lines 184-9: “Following calibration under the historical scenario, the model simulated a “counterfactual scenario” in which schools would have remained fully open throughout the pandemic. This involved modifying the dynamic adjustment of population mixing such that rates of contact at schools are maintained at baseline levels during periods in which school closures occurred. The modifications applied to the mixing matrices to capture school closures are presented in detail in Supplementary Section A.1.2.5.”

R4.C4) Regarding the sensitivity analyses, it is worth evaluating whether these analyses are comprehensive enough to address the potential variability in the model's assumptions. Are there any additional sensitivity analyses, such as varying the number of days schools were closed or the impact of variations in vaccine efficacy, that could strengthen the findings?

In this revision, we have performed additional sensitivity analyses around key aspects of our model. First, we explored alternative model structures considering both a larger and a smaller number of serialised compartments to capture both incubation and active disease period. Second, we reran analyses using an alternative source of mixing matrices to explore the sensitivity of our model outputs to social mixing assumptions. These analyses are in addition to two other sensitivity analyses that we already considered in the previous submission around key assumptions of our model. We have now run over 250 analyses across 74 countries in total. We believe that adding any further sensitivity analyses would make the manuscript more difficult to read and the findings difficult to summarise. We believe the current range of analyses sufficiently addresses the potential variability in our model and supports the robustness of our findings.

R4.C5) The decision to use four sequential compartments for both exposed and active states to achieve an Erlang distribution is technically sound, as it avoids the unrealistic assumptions of an exponential distribution for disease progression. However, the authors should clarify whether the number of compartments used is adequately justified. Could fewer or more compartments have yielded similar or better results?

We thank Reviewer #4 for this suggestion. We have now performed additional analyses considering alternative numbers of sequential compartments (n=2 and n=6, compared to n=4 in the main analysis). These explorations revealed no noticeable changes in the conclusions, providing confidence in the robustness of our modelling approach. The analyses are presented in detail in Supplementary Appendix Section B.2.6 and we added the following text to the Results section:

Lines 297-300: Similarly, varying the number of compartments used to represent the incubation and active disease periods produced model outputs that were virtually identical to those of the base-case analyses (Supplementary Figures S19-21)”

Illustration of all the assumptions now considered:

R4.C6) The authors employed a Bayesian approach for model calibration, which is a solid choice for managing the complex uncertainties in the model parameters. However, the manuscript should provide more detail on the convergence diagnostics of their chains and include information on how the robustness of their parameter estimates was ensured.

We recognise that we had not described the way algorithm convergence was assessed in the original version of the manuscript. We have now expanded the description of our calibration approach in the Methods section:

Lines 177-82: “Model calibration was achieved through a Bayesian approach to account for uncertainty around key epidemiological parameters and the data used for model fitting. We employed an Adaptive Differential Evolution Metropolis algorithm to facilitate efficient sampling of the parameter space. Sampling convergence was assessed through trace visual inspection and the Gelman-Rubin statistic.”

We also added the following sentence in Section A.3 of the Supplementary Appendix:

“Sampling convergence was assessed by analysing all parameter traces visually and by requiring that the

Gelman-Rubin statistic was below 1.05.”

R4.C7) The authors selected uniform prior distributions for the primary parameters during calibration. It would be beneficial for the manuscript to justify this choice of priors. Were other distribution types considered, and if so, why were uniform priors ultimately chosen?

The choice of uniform prior distributions for the primary parameters was made because we had no substantive prior knowledge regarding their values. By using uniform priors, we allowed the process of fitting to data to drive the estimation of these parameters without imposing any preconceived constraints. Using uniform priors is a common practice in such cases where prior knowledge is lacking, as it provides a neutral starting point that allows the data to be the primary source of information for parameter estimation.

R4.C8) The authors mentioned the use of random processes to account for unexplained variations in transmission risk. It is important to clarify whether the implementation of random walks and Gaussian updates is appropriate and to provide sufficient reasoning for the frequency of these updates.

The random walk with Gaussian update is commonly employed in semi-mechanistic models of infectious disease transmission as it allows consideration of the different directions of change in a balanced way, and it requires a single hyper-parameter (the distribution standard deviation). See for example reference work from Funk et al. (Epidemics 2018, doi.org/10.1016/j.epidem.2016.11.003)

The choice of the frequency of the random process updates is now discussed in Section A.1.2.6 of the Appendix:

R4.C9) The model captures changes in social interactions over time using dynamic age-specific mixing matrices, with particular attention to school closures. The manuscript should expand on whether the assumptions around the modification of contact rates, such as the effect of partial school closures on contact rates, are well-supported by empirical data.

As noted by Reviewer #4, social mixing is modelled using age-specific mixing matrices and we capture school closures by removing the school components from the contact matrices (Equation A.3). For a detailed justification of why we believe this mechanistic approach is appropriate, please refer to our response to Reviewer #2’s comment (R2.C1).

R4.C10) The use of a convolution process to estimate hospitalisations and deaths from the model's outputs is a practical approach. However, the manuscript should consider whether the convolution process has been adequately validated against empirical data from the analysed countries. Are the assumptions regarding the time distributions for hospitalisations and deaths appropriate and consistent with the literature?

We believe our convolution-based method offers a significant improvement over the traditional approach of modelling hospitalisations and deaths as exit transitions from an "active disease" compartment. Indeed, the classic modelling approach is constrained by the assumption that the time to hospitalisation or death follows an exponential distribution, which may not accurately reflect the observed duration due to its mode being zero and its heavy-tailed nature. In contrast, our convolution approach provides greater flexibility by allowing us to select statistical distributions that better capture these durations. We found that gamma distributions, in particular, are well-suited to represent the duration spread observed in the literature.

R4.C11) The adjustment of infection fatality rates using country-specific multipliers is a necessary step given the variability in health system responses. The method for calibrating these multipliers should be clearly explained and justified in the manuscript.

We thank Reviewer #4 for detecting the absence of justification for this modelling choice. We added the following text to the Methods section:

Lines 162-8: “The infection fatality rate (IFR) was allowed to vary by country to account for differences in COVID-19 death definition and reporting standards. In particular, age-specific death rates were multiplied by a single scaling factor calibrated independently for each country. This approach maintains a consistent IFR age-profile across countries, allowing for meaningful comparisons while accommodating country-specific variations in overall mortality rates. It also reduces analysis complexity by requiring only one mortality-related calibrated parameter per country.”

R4.C12) I suggest that the authors include additional validations of their model outputs against independent datasets, such as sub-national data or external validation using datasets not employed in the model calibration.

The counterfactual nature of the scenario that we modelled implies that there is no data available to inform what would have happened in the absence of school closure. For this reason, we could not directly validate our estimates using data. However, we undertook a literature review to identify previous modelling studies investigating school closures for COVID-19 with the aim to compare our results with previous estimates.

We identified 17 published studies estimating the impacts of school closures on any COVID-19 epidemiological indicator. None of these studies estimated the impact over extended periods of time covering multiple epidemic waves. Studies mostly focused on specific regions—England, Finland, France, Germany, Iran, United Kingdom, United States, Ontario (Canada), Toronto (Canada), and Shanghai (China)—or considered a theoretical framework without any specific setting. Several studies considered only the effect of school closures in combination with other interventions (e.g. complete lockdown). Most studies focused on quantifying the effect on SARS-CoV-2 transmission by estimating changes to the reproduction number or growth rate, including two that combined data from multiple countries and territories to estimate an aggregated effect. The collective evidence suggests that school closures generally reduce SARS-CoV-2 transmission, which is consistent with our findings. However, we could not identify previous estimates that could be compared with ours, due to differences in study objectives, design, and periods investigated.

We have now edited the Discussion section to include some of this information:

Lines 364-70: “Previous studies investigating the epidemiological impact of school closures on COVID-19 mostly focused on the instantaneous effects on transmission through estimations of reproduction number or growth rate. Most studies focused on specific countries such as Canada [26], France [27], Germany [25], and China [28], or applied conceptual models to theoretical settings [29]. Despite the variation in scope and settings, the collective evidence indicates that school closures tend to reduce SARS-CoV-2 transmission [27, 28, 30-32], which is consistent with our findings regarding the short-term effects of the intervention. […]”

R4.C13) I propose that the authors include more real-world scenarios in their sensitivity analyses, such as varying the effectiveness of non-pharmaceutical interventions or testing the impact of different vaccination rollout speeds.

In our current analyses, we have already varied the direct effect of school closures by adjusting two key parameters: the relative contribution of school contacts to overall mixing, and the fraction of students onsite during periods of school closures. Additionally, we have performed sensitivity analyses that consider alternative assumptions about how school closures may influence social interactions within household settings. Finally, Sensitivity Analysis SA2 considered alternative assumptions regarding the effectiveness of non-pharmaceutical interventions as it removed the contribution of the Google mobility data from the modelled social mixing, thus exclusively relying on the random transmission adjustment to capture mobility changes in locations other than households and schools.

Evaluating different vaccination rollout scenarios would provide meaningful insights. However, we believe such an analysis would require changing the scope of the current study dramatically. Our focus here is on the role of school closures specifically, and incorporating vaccination scenarios would shift the focus toward a broader pandemic response strategy, which we believe is outside the current study's objectives.

Finally, as previously mentioned in response to comment R4.C4, we are concerned that including more sensitivity analyses would make the manuscript very challenging to read and the findings difficult to interpret.

R4.C14) The manuscript should clearly articulate the limitations of the model. For instance, the authors could discuss the potential impact of unmodeled factors such as behavioural changes not captured by mobility data or the effect of partial immunity in populations with mixed infection and vaccination histories.

We added the following paragraph in the Discussion section:

Lines 407-14: “Despite our efforts to incorporate numerous factors relevant to SARS-CoV-2 transmission, our model did not account for every mechanism influencing disease spread and evolution over time. Specifically, changes in individual behaviours, such as mask-wearing and social distancing practices, were not explicitly modelled, beyond what could be inferred from mobility data. However, the use of an automatically calibrated random transmission adjustment helped to account for unmodeled factors. Importantly, we did not observe abrupt changes in this random adjustment, suggesting that the factors explicitly captured by our model were sufficient to explain most of the observed variations in COVID-19 dynamics.”
