# Supplementary material for: Estimating the impact of school closures on the COVID-19 dynamics in 74 countries: A modelling analysis
Source: PLoS Med. 2025 Jan 21;22(1):e1004512. doi: 10.1371/journal.pmed.1004512 (PMC11793732; doi:10.1371/journal.pmed.1004512)
Supplement: Attachment — Submitted filename: Comments.docx [file pmed.1004512.s003.docx]

**Responses to reviewers’ comments**

11^th^ November 2024

Dear Editor,

Many thanks for the opportunity to further revise our manuscript. We thank you and the reviewers for the detailed and highly relevant comments. Please see below for our response to the comments.

Yours sincerely,

Dr Romain Ragonnet

Corresponding author

**Comments from the Academic Editor**

My overall impression is that the authors have done an excellent job of responding to the reviewers' comments, and that the manuscript is now strong, and appropriately nuanced in its discussion of results.

We thank the Academic Editor for the thorough review of our manuscript and this positive comment.

**Comments from the Editorial Team**

Please note that we always require a point-by-point response to not only reviewer comments, but all editorial comments, including general editorial requests. Please be sure to provide such a document that addresses all editorial comments of the previous decision letter.

We apologise for not providing a point-by-point response to the General Editorial Requests in the previous revision. We have now incorporated this at the end of this document.

**Comments from Reviewer #2:**

The authors have satisfyingly addressed my comments. I would like to congratulate the authors for this interesting study. 2 final minor comments:
R1.C1) Abstract : « We estimate that school closures achieved moderate to significant burden reductions in most settings over the period 2020-2022. They reduced peak hospital occupancy in nearly all countries, with 72 out of 74 countries showing a positive median estimated effect. However, we identified countries, including multiple European nations and Indonesia, where school closures may have increased overall COVID 19 mortality. » Authors give a proportion of countries (72/74) in which impact is positive on peak hospital occupancy. It would be good to provide a similar statistics in the second sentence discussing the risk of increasing overall mortality.

We modified the abstract to incorporate Reviewer #1’s suggestion:

Lines 28-31: “They reduced peak hospital occupancy in nearly all countries, with 72 out of 74 countries (97%) showing a positive median estimated effect. However, we identified nine countries (12%), including several European nations and Indonesia, where school closures may have increased overall COVID-19 mortality (based on median estimates).”

R1.C2) Methods: "Following calibration under the historical scenario, the model simulated a  "counterfactual scenario" in which schools would have remained fully open throughout the pandemic." Please clarify what is considered for school holidays? In the counterfactual scenario, was it assumed that schools remain open during school holidays? If the counterfactual scenario is really "schools always open even during holiday periods", the difference in terms of what should be expected for a pattern of standard school year (i.e. when schools are closed during standard holiday terms) should at least be discussed.

We recognise that this aspect required clarification. We have included a specific description of the counterfactual “schools open” scenario:

Lines 199-201: “Following calibration under the historical scenario, the model simulated a “counterfactual scenario” in which schools would have remained fully open throughout the pandemic (outside of the usual academic break periods).”

The social mixing assumptions applied during academic breaks are also describes in S1 Appendix Section A.1.2.5.2.

**Comments from Reviewer #3:**

I thank the authors for their extensive reply to the questions raised by all the reviewers.
I acknowledge the great effort made by the authors in providing more supplementary analyses and comments to justify their modeling choices and improve clarity. I also appreciate that the negative impact of school closure for few countries is less emphasized in the current text than in its previous version.
Overall, I believe that the quality of the manuscript has improved.

R3.C1) I appreciate the post-hoc statistical analysis on the potential drivers of heterogeneity: it highlights that "young" countries can benefit more from generalized school closures.

We thank Reviewer #3 for this positive comment.

R3.C2) The post-hoc analysis also underlines that, although longer closures are not always associated with a larger number of averted infections and deaths, short closures are rarely highly beneficial. Looking at this and at the results for all the single countries in S2 Appendix, it seems that the timing, duration and type of single school closure events with respect to epidemic peaks may have an impact on the cumulative proportion of cases as well as on deaths. In fact, full closure of schools was not implemented during the Delta phase of the pandemic in many countries where a minor overall impact can be observed. Another counterfactual scenario with more stringent features for school closure would be very interesting to understand this aspect; however, I recognize that there are already many sensitivity analyses. Therefore, I suggest either implementing this counterfactual scenario at least for a representative country (e.g. Indonesia), and commenting on the possible importance of timing and duration on the outcome of school closure interventions.

We agree with the interesting point made by Reviewer #3 and we have now conducted the suggested analysis applied to Indonesia. Namely, we considered an additional counterfactual scenario where schools would have been fully closed during the Delta phase of the pandemic.

We included the following text in the Results section:

Lines 284-8:“We explored whether more stringent school closures during the Delta wave could have reduced COVID-19 mortality in Indonesia. With schools only partially closed at the time, we considered a counterfactual scenario with full closures throughout 2021. Our estimates suggest that this intervention alone would have been insufficient to reverse the trajectory of Indonesia’s Delta wave, resulting in only a relatively small reduction in COVID-19 burden (S1 Appendix Section B.2.7).”

The analysis is described in detail in the S1 Appendix, including the following figure:

R3.C3) I would also add a comment on accurate testing and contact tracing procedures that may be employed to perform more effective reactive closures at school (or even class) level, in addition to what the authors have already added in the Discussion.

We included the following sentence in the Discussion section:

Lines 382-4: “Accurate testing and contact tracing procedures may also enable more effective, targeted closures at the school or even class level”.

R3.C4) I would suggest, as future work, to perform further analysis restricted to a small subset of countries (even a single one) for which the pattern of school closures at regional/local level is known, as this would provide more precise insight into the feasibility and expected outcome of this measure.

We added the following text to the Discussion section:

Lines 437-40: “Future work may expand on our analysis by considering a more limited set of countries or regions with detailed information on individual behaviours and specific control policies, providing more precise insights into the effects of school closures.”

R3.C5) Closing schools has generally the effect to reduce to some extent also contacts in other locations, e.g. public transportation: can this be observed in the Google Mobility data?

We agree with Reviewer #3 that school closures could possibly have this effect. However, even if Google Mobility data show mobility reductions during periods of school closures, it is impossible to determine whether these reductions can be attributed to school closures specifically or to other policies often implemented alongside them.

R3.C6) I found some misprints.
Line 300: Figures S19-21 -> Figures S25-28.
Caption of Figure S27: India -> Indonesia
Supp A.1.2.5.2: "between 10 and 30% of students attended on-site learning" -> "between 10 and 50%...."

We thank Reviewer #3 for identifying these misprints. They have now been corrected.

**Comments from Reviewer #4:**

R4.C1) The authors mention the use of a Bayesian approach with an Adaptive Differential Evolution Metropolis algorithm to calibrate the model. However, to enhance understanding of the calibration process, it would be beneficial to provide more comprehensive details. This should include the priors chosen for all key parameters, the criteria used to determine convergence, and the length of the burn-in period. Additionally, summarising the posterior distributions obtained and discussing the sensitivity of the results to different prior choices would offer readers a clearer picture of how model parameters were estimated and validated.

We have enhanced our description of model calibration in the main text:

Lines 188-95: “Model calibration was achieved through a Bayesian approach to account for uncertainty around key epidemiological parameters and the data used for model fitting. We employed an adaptive differential evolution Metropolis algorithm to facilitate efficient sampling of the parameter space. For each analysis, we used eight Metropolis chains, each running for 35,000 iterations. We discarded the first 25,000 iterations of each chain as burn-in before collecting samples for analysis. Sampling convergence was assessed through trace visual inspection and the Gelman-Rubin statistic ($\hat{R}<1.1$)”

We also indicate where more details about the calibration approach can be found in the S1 Appendix, including about prior distributions and likelihood calculation:

Lines 195-7: “S1 Appendix Section A.3 describes our calibration approach in details, along with the prior distributions used for all calibrated parameters and the model likelihood calculation.”

We have made every effort to provide a clear and transparent description of our calibration approach, including in the main text, appendix, and GitHub repository. We also presented posterior estimates for the time-variant random process for each country analysed. In addition to this, we now provide plots of posterior distributions and pairwise comparison for the three countries highlighted in the main text. Below is an example for Morocco (S1 Appendix Figure S31):

R4.C2) The random perturbation, denoted as W(t), plays a critical role in capturing unobserved variability in transmission. A more thorough explanation of how this random process interacts with other model variables and how it represents interventions not captured by explicit covariates—such as changes in behaviour—would greatly enhance transparency. Furthermore, providing the mathematical form of W(t) and detailing any assumptions regarding its distribution and correlation structure would add clarity and allow for better understanding of how this stochastic element affects the model.

We have modified the description of the random process to include more details about what mechanisms it is expected to capture:

Lines 156-62: “We further adjusted the risk of SARS-CoV-2 transmission using a time-variant random process, making the model semi-mechanistic. This random process reflected additional variations in transmission risk beyond those explicitly captured by model inputs, such as vaccination, dynamic mobility, and new variant emergence not captured by strain stratification. The process is expected to capture changes in individual behaviours such as physical distancing or improved hygiene, as well as changes in social contact patterns due to interventions not already captured by Google mobility data and school closures.”

The mathematical form of the random perturbation W(t) is provided in S1 Appendix Section A.1.2.6. We have now included the following text in the Methods section to specify how the random perturbation W(t) was modelled and to refer to the Appendix section which describes the process in details:

Lines 165-6: “We used a random walk with Gaussian update to model this random process, as described in S1 Appendix Section A.1.2.6.”

Finally, the calibrated random process profile is presented for all analyses and described in the Results section:

Lines 296-304: “The estimated transmission adjustment (random process) over time is presented for the three highlighted countries in Panel D of Figures 3-5, and for all analysed countries in S1 Appendix. Our model consistently captured local epidemic dynamics without requiring abrupt changes in the random process. Notably, we found no systematic shifts coinciding with school closure or reopening times, suggesting that the transmission changes induced by school closures were adequately captured by our approach based on mixing matrix modifications. However, we observed a gradual decline in the random process in some countries (e.g. Morocco, Figure 3), suggesting the presence of factors not explicitly captured by the model that may have contributed to reduced transmission over time (e.g. increased awareness, improved hygiene).”

R4.C3) Since the model relies on multiple data sources, such as death counts, seroprevalence, and mobility data, it is essential to elaborate on how any missing data or discrepancies between sources were managed. If imputation techniques were employed or if weighting was based on data reliability, these methods should be clearly outlined. A thorough explanation of the data-handling process would provide readers with insight into the model's robustness and reliability.

We thank Reviewer #4 for this insightful comment and appreciate the opportunity to clarify how we handled the data for our model.

We would like to emphasise that we used a single source of data for each type of data (e.g. death counts, seroprevalence, mobility data…), mitigating the potential for discrepancies between sources. We also ensured that all data used were from reputable, publicly available sources, and we applied stringent inclusion criteria described in the manuscript to ensure the reliability and consistency of the data.

Regarding missing data and imputation techniques, we made every effort to avoid the need for imputation. We ensured that only fully available data points were used for model calibration, meaning no missing data imputation was required. Instead, we focused on thoroughly exploring and verifying the input data for any inconsistencies or implausible trends. For example:

- We visually inspected the data for each country individually to check for issues such as the emergence of variants of concern occurring before their global emergence date or a decrease in vaccination coverage.

- We defined strain emergence using the criterion outlined in Section A.1.2.4 of the S1 Appendix, where a strain (Delta or Omicron) was considered to have emerged when at least two cases of that strain were reported in a single week in GISAID, accounting for at least 1% of all reported cases during that week.

- For COVID-19 deaths, as detailed in Section A.3.2 of S1 Appendix, we applied a 7-day moving average to the daily death counts reported by the WHO to smooth any fluctuations.

- We carefully considered the inclusion criteria for seroprevalence data to ensure its robustness and consistency (S1 Appendix Section A.3.2).

We have now modified the Methods section to clarify our approach to data handling. We renamed the opening section of the Methods from “Country inclusion” to “Country inclusion and data sources”, and we included the following paragraph:

Lines 115-20: “To ensure the robustness and reliability of the analyses, we applied strict inclusion criteria and used only available data points for model calibration, such that imputation for missing data was not required. Additionally, we visually inspected the data for each country to identify any unrealistic features, such as the emergence of variants of concern prior to their global recognition or a decrease in vaccination coverage over time.”

R4.C4) The paper reports various sensitivity analyses, but there is a need for greater detail regarding the framework used. Specifically, it would be helpful to describe the criteria for selecting parameters included in these analyses. Additionally, explaining how the ranges for sensitivity analyses were chosen—whether based on literature, observed data variability, or other factors—would offer context. Addressing whether and how parameter correlations were accounted for during sensitivity analyses is also crucial to provide a complete picture of the approach to assessing model sensitivity.

We performed sensitivity analyses by focusing on parameters and assumptions that we anticipated to be the most critical for addressing the research question. The selection of parameters was based on their relevance to the model's output and their potential to influence the outcomes of the study significantly. In particular, we explored multiple alternative assumptions related to modelled social mixing and effect of school closures on parameters.

The ranges used for sensitivity analyses were determined by considering plausible values derived from literature and expert opinion from authors RR, JMT, ESM and AEH. In instances where data was limited, we chose ranges that reflected reasonable uncertainty or variations that could realistically occur in the system under study.

We have now edited the Methods section to include the following text:

Lines 219-23: “In addition to the uncertainty in our input parameters, we considered sensitivity analyses of parameters and assumptions most relevant to the effects of school closures, selected for their direct impact on model outcomes and based on the expert judgment of authors RR, JMT, ESM and AEH. Specifically, we focused on assumptions regarding social interactions or the effect of school closures on contact matrices.”

R4.C5) To improve the statistical rigor of the analysis, it is recommended to elaborate on how parameter identifiability was assessed. Were any parameters found to be highly correlated or unidentifiable? Describing such aspects would highlight the challenges encountered during the modelling process. Additionally, outlining how uncertainty in parameter estimates was propagated to model outcomes would help readers assess the robustness and reliability of the model's conclusions. Detailing these approaches ensures transparency and strengthens the overall confidence in the study's findings.

Assessing identifiability across all analyses was not feasible due to the model’s application across 74 countries and multiple sensitivity analyses. Given the study's scale and complexity, a comprehensive identifiability assessment for each case would have required computational resources beyond the scope of this manuscript.

To minimise identifiability issues, we intentionally included only the smallest possible number of parameters in the Metropolis sampling, focusing on parameters that are either the least known or the most crucial to this analysis. This approach reduces the risk of parameter overlap and ensures that only the most essential parameters drive the model’s behaviour.

We now include pairwise comparison plots of posterior distributions for the three countries highlighted in the main text (S1 Appendix, Section B.3; see response to comment R4C1). These plots reveal no clear correlations between parameters, suggesting that identifiability issues are unlikely to affect our main findings.

Finally, we provide comparisons between prior and posterior distributions for the three highlighted countries to illustrate how the data used for calibration have influenced the posterior beliefs in each analysis (S1 Appendix, Section B.3). This also serves to illustrate how uncertainty in parameter estimates propagates through the model.

**General Editorial Requests from previous revision**

(Note: not all will apply to your paper, but please check each item carefully)

GER1) We ask every co-author listed on the manuscript to fill in a contributing author statement, making sure to declare all competing interests. If any of the co-authors have not filled in the statement, we will remind them to do so when the paper is revised. If all statements are not completed in a timely fashion this could hold up the re-review process. If new competing interests are declared later in the revision process, this may also hold up the submission. Should there be a problem getting one of your co-authors to fill in a statement we will be in contact. Please do not add or remove authors without first discussing this with the handling editor. You can see our competing interests policy here: <http://journals.plos.org/plosmedicine/s/competing-interests>.

All co-authors have now returned their forms.

GER2) Please upload any figures associated with your paper as individual TIF or EPS files with 300dpi resolution at resubmission; please read our figure guidelines for more information on our requirements: <http://journals.plos.org/plosmedicine/s/figures>. While revising your submission, please upload your figure files to the PACE digital diagnostic tool, <https://pacev2.apexcovantage.com/>. PACE helps ensure that figures meet PLOS requirements. To use PACE, you must first register as a user. Then, login and navigate to the UPLOAD tab, where you will find detailed instructions on how to use the tool. If you encounter any issues or have any questions when using PACE, please email us at [PLOSMedicine@plos.org](mailto:PLOSMedicine@plos.org).

We used the PACE digital diagnostic tool to ensure that all figures meet PLOS requirements, and upload them as individual TIF files with 300dpi resolution.

GER3) Please ensure that the paper adheres to the PLOS Data Availability Policy (see <http://journals.plos.org/plosmedicine/s/data-availability>), which requires that all data underlying the study's findings be provided in a repository or as Supporting Information. For data residing with a third party, authors are required to provide instructions with contact information (web or email address) for obtaining the data. Please note that a study author cannot be the contact person for the data. PLOS journals do not allow statements supported by "data not shown" or "unpublished results." For such statements, authors must provide supporting data or cite public sources that include it.

The data and code associated with this study are available via a GitHub repository as indicated in the Methods section, along with the repository’s URL.

GER4) We expect all researchers with submissions to PLOS in which author-generated code underpins the findings in the manuscript to make all author-generated code available without restrictions upon publication of the work. In cases where code is central to the manuscript, we may require the code to be made available as a condition of publication. Authors are responsible for ensuring that the code is reusable and well documented. Please make any custom code available, either as part of your data deposition or as a supplementary file. Please add a sentence to your data availability statement regarding any code used in the study, e.g. "The code used in the analysis is available from Github [URL] and archived in Zenodo [DOI link]" Please review our guidelines at <https://journals.plos.org/plosmedicine/s/materials-software-and-code-sharing> and ensure that your code is shared in a way that follows best practice and facilitates reproducibility and reuse. Because Github depositions can be readily changed or deleted, we encourage you to make a permanent DOI'd copy (e.g. in Zenodo) and provide the URL.

The code used in the analysis is available from GitHub and we included the following statement in the *Data Sharing* section: “All data and codes used and generated by this analysis are available in a GitHub repository (github.com/monash-emu/covid19_school_closures).”

GER5) All authors must declare their relevant competing interests per the PLOS policy, which can be seen here:
<https://journals.plos.org/plosmedicine/s/competing-interests>
For authors with ties to industry, please indicate whether any of the interests has a financial stake in the results of the current study.

All authors declared they have no conflict of interest.

FORMATTING - GENERAL
GER6) Abstract: Please structure your abstract using the PLOS Medicine headings (Background, Methods and Findings, Conclusions). Please combine the Methods and Findings sections into one section.

We have edited the Abstract using the required formatting.

GER7) At this stage, we ask that you include a short, non-technical Author Summary of your research to make findings accessible to a wide audience that includes both scientists and non-scientists. The Author Summary should immediately follow the Abstract in your revised manuscript. This text is subject to editorial change and should be distinct from the scientific abstract. Ideally each sub-heading should contain 2-3 single sentence, concise bullet points containing the most salient points from your study. In the final bullet point of 'What Do These Findings Mean?', please include the main limitations of the study in non-technical language. Please see our author guidelines for more information: <https://journals.plos.org/plosmedicine/s/revising-your-manuscript#loc-author-summary>.

We included an Author Summary in the revised manuscript.

GER8) Please express the main results with 95% CIs as well as p values. When reporting p values please report as p<0.001 and where higher as the exact p value p=0.002, for example. Throughout, suggest reporting statistical information as follows to improve clarity for the reader "22% (95% CI [13%,28%]; p</=)". Please be sure to define all numerical values at first use.

Not Applicable

GER9) Please include page numbers and line numbers in the manuscript file. Use continuous line numbers (do not restart the numbering on each page).

We have modified the manuscript formatting to include continuous line and page numbers.

GER10) Please cite the reference numbers in square brackets. Citations should precede punctuation.

We cited reference numbers using the required formatting.

FIGURES AND TABLES
GER11) Please provide titles and legends for all figures and tables (including those in Supporting Information files).

We provided titles and legends for all figures and tables.

GER12) Please define all abbreviations used in each figure/table (including those in Supporting Information files).

We defined all abbreviations used in each figures and tables.

GER13) Please consider avoiding the use of red and green in order to make your figure more accessible to those with color blindness.

We tried to avoid the use of red and green as much as possible, although using these colours was sometimes necessary.

SUPPLEMENTARY MATERIAL
GER14) Please note that supplementary material will be posted as supplied by the authors. Therefore, please amend it according to the relevant comments outlined here.

The supplementary material was amended to address the comments.

GER15) Please cite your Supporting Information as outlined here: <https://journals.plos.org/plosmedicine/s/supporting-information>

The supporting information is now cited using the required formatting.

REFERENCES
GER16) PLOS uses the numbered citation (citation-sequence) method and first six authors, et al.

We used the required citation format throughout the manuscript.

GER17) Please ensure that journal name abbreviations match those found in the National Center for Biotechnology Information (NCBI) databases (<http://www.ncbi.nlm.nih.gov/nlmcatalog/journals>), and are appropriately formatted and capitalised.

Journal name abbreviations match those found in the NCBI database.

GER18) Where website addresses are cited, please include the complete URL and specify the date of access (e.g. [accessed: 12/06/2023]).

We included URL and date of access in website citations.

GER19) Please also see <https://journals.plos.org/plosmedicine/s/submission-guidelines#loc-references> for further details on reference formatting.

We read the reference formatting guidelines mentioned above and ensured that our manuscript complies with them.

STUDY TYPE-SPECIFIC REQUESTS - MODELLING STUDIES

The following list is derived from Geoffrey P Garnett, Simon Cousens, Timothy B Hallett, Richard Steketee, Neff Walker. Mathematical models in the evaluation of health programmes. (2011) Lancet DOI:10.1016/S0140-6736(10)61505-X:   

GER20) If pertinent, please provide a diagram that shows the model structure, including how the natural history of the disease is represented, the process and determinants of disease acquisition, and how the putative intervention could affect the system.

Model structure diagrams are provided in the technical description of the model.

GER21) Please provide a complete list of model parameters, including clear and precise descriptions of the meaning of each parameter, together with the values or ranges for each, with justification or the primary source cited and important caveats about the use of these values noted.

Model parameters are clearly described along with their values (or prior distributions where relevant). We also provided detailed justifications for these values and distributions, using clearly acknowledged sources (e.g. S1 Appendix Section A.1.4).

GER22) Please provide a clear statement about how the model was fitted to the data, including goodness-of-fit measure, the numerical algorithm used, which parameter varied, constraints imposed on parameter values, and starting conditions.

We included the information above as follows:

- Methods, Lines 184-5: “For each included country, we first fitted the model to local observations of reported COVID-19 deaths over time and SARS-CoV-2 seroprevalence estimates (where available).”
- Methods, Lines 187-98: “Model calibration was achieved through a Bayesian approach to account for uncertainty around key epidemiological parameters and the data used for model fitting. We employed an adaptive differential evolution Metropolis algorithm to facilitate efficient sampling of the parameter space. For each analysis, we used eight Metropolis chains, each running for 35,000 iterations. We discarded the first 25,000 iterations of each chain as burn-in before collecting samples for analysis. Sampling convergence was assessed through trace visual inspection and the Gelman-Rubin statistic ($\bar{R}<1.1$). S1 Appendix Section A.3 describes our calibration approach in details, along with the prior distributions used for all calibrated parameters and the model likelihood calculation. Our calibration approach resulted in a comprehensive exploration of the parameter space and identification of numerous configurations capable of replicating real-world observations.”
- S1 Appendix sections:
  - A.3.1: Parameters varied during calibration
  - A.3.2: Calibration targets
  - A.3.3: Likelihood definition
  - Technical details about starting conditions, numerical algorithm provided in beginning of Section A.3

GER23) For uncertainty analyses, please state the sources of uncertainties quantified and not quantified [can include parameter, data, and model structure].

Uncertainty was included as part of our calibration approach, which is described in detail in the main text and S1 Appendix. Our response to comment GER22 provides details about where the relevant information can be found in the manuscript.

GER24) Please provide sensitivity analyses to identify which parameter values are most important in the model. Uncertainty estimates seek to derive a range of credible results on the basis of an exploration of the range of reasonable parameter values. The choice of method should be presented and justified.

We performed extensive sensitivity analyses to explore how the model outputs are affected by changes in key model parameters and assumptions. These include:

- An exploration of alternative model structures considering both a larger and a smaller number of serialised compartments to capture both incubation and active disease period.

- Using an alternative source of mixing matrices to explore the sensitivity of our model outputs to social mixing assumptions.

- Considering a different assumption regarding the impact of school closures on people’s mobility.

- Running all analyses without the contribution of Google Mobility data.

GER25) Please discuss the scientific rationale for the choice of model structure and identify points where this choice could influence conclusions drawn. Please also describe the strength of the scientific basis underlying the key model assumptions.

The rationale for the choice of model structure is described in the main text (e.g. Methods 🡪 Model Description Lines 114-68) and S1 Appendix:

- A.1.2.1 Compartment types and sequence

- A.1.2.2 Model stratification by age

- A.1.2.3 Capturing the effects of vaccination

- A.1.2.4 Modelling multiple viral strains

- A.1.2.5 Dynamic social mixing

We also demonstrated the robustness of our model structure assumptions by conduction sensitivity analyses considering alternative structural assumptions (S1 Appendix Sections B.2.6 and B.2.3).

GER26) For studies that develop a prediction model or evaluate its performance, please ensure that the study is reported according to the TRIPOD statement (<https://www.equator-network.org/reporting-guidelines/tripod-statement>) and include the completed checklist as Supporting Information. Please add the following statement, or similar, to the Methods: "This study is reported as per the Transparent Reporting of a Multivariable Prediction Model for Individual Prognosis Or Diagnosis (TRIPOD) statement (S1 Checklist)." For studies using machine learning, please use the TRIPOD-AI checklist. When completing the checklist, please use section and paragraph numbers, rather than page numbers.

Not Applicable.
